# Et_3_SiH + KO^*t*^Bu provide multiple reactive intermediates that compete in the reactions and rearrangements of benzylnitriles and indolenines†

**DOI:** 10.1039/d0sc04244g

**Published:** 2020-10-21

**Authors:** Andrew J. Smith, Daniela Dimitrova, Jude N. Arokianathar, Kenneth F. Clark, Darren L. Poole, Stuart G. Leach, John A. Murphy

**Affiliations:** Department of Pure and Applied Chemistry Thomas Graham Building, 295 Cathedral Street Glasgow G1 1XL UK john.murphy@strath.ac.uk; GlaxoSmithKline Medicines Research Centre Gunnels Wood Road Stevenage Hertfordshire SG1 2NY UK

## Abstract

The combination of potassium *tert*-butoxide and triethylsilane is unusual because it generates multiple different types of reactive intermediates simultaneously that provide access to (i) silyl radical reactions, (ii) hydrogen atom transfer reactions to closed shell molecules and to radicals, (iii) electron transfer reductions and (iv) hydride ion chemistry, giving scope for unprecedented outcomes. Until now, reactions with this reagent pair have generally been explained by reference to one of the intermediates, but we now highlight the interplay and competition between them.

## Abstract 2

The combination of potassium *tert*-butoxide and triethylsilane provides simultaneous access to multiple reactive intermediates, radicals, H-atom donors, hydride donors and electron donors, giving scope for unprecedented reaction outcomes.

## Graphical abstract

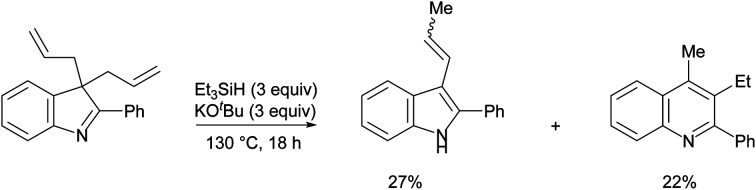

## Introduction

A novel reducing system, consisting of the reagent-pair, triethylsilane and potassium *tert*-butoxide was reported by Stoltz, Grubbs *et al.* in 2013.^1^ The combination of the two reagents has since been investigated by a number of research groups^2–15^ and provides a range of distinctive reaction types, arising through an unprecedented menu of reactive intermediates formed in the reaction, including triethylsilyl radicals **1**, silanates **2** as hydrogen atom donors to both closed shell molecules and to radicals, and as potential hydride ion donors, and *tert*-butoxytriethylsilyl radical anions **3** as a very powerful electron donor. Exposing substrates simultaneously to multiple reactive intermediates is not routinely encountered in organic chemistry, other than in modelling of prebiotic conditions,^16^ and so the variety of reactive intermediates produced by this reagent pair provides opportunities to witness unusual outcomes.

**Scheme 1:**
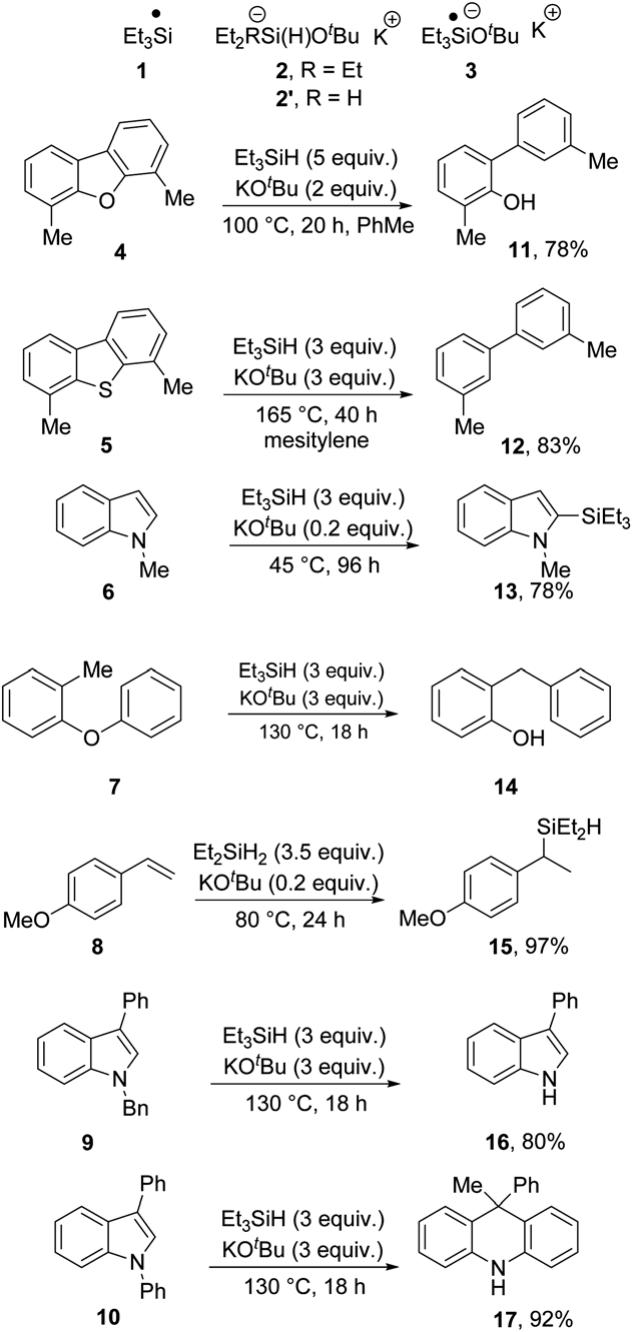


Thus, triethysilyl radicals **1** are candidates for the conversions of substrates **4–7** (ref. 2–6, 8 and 13) to their products **11–14**. (Note that silylation reactions, as in formation of **13** usually occur at lower temperatures, here 45 °C). On the other hand, Jeon has established^9^ that silanate complex **2′** (and less efficiently **2**) conducts a potassium ion-dependent H-atom transfer to afford hydrosilylation products **15** from styrenes such as **8** at 80 °C. Tuttle, Murphy *et al.* have reported that *N*-benzylindoles **9** are deprotected by electron transfer reactions with **3** acting as electron donor.^7^ In each of the above cases, the products can be attributed to one of the reactive intermediates. Most recently, a more complex rearrangement of *N*-aryl indoles (*e.g.***10**) to dihydroacridines (in this case, **17**) features sequential electron transfer from **3** and H-atom transfer from **2**.^14^ In addition to these transformations, the reagent pair Et_3_SiH/KO^*t*^Bu has found wider applications in silylation of alcohols^10^ and amines,^11^ as well as the silylation of terminal alkynes.^15^ The broad range of possible pathways featuring different reactive intermediates is what makes this reagent-pair so fascinating (Scheme 1).^12^

**Scheme 2:**
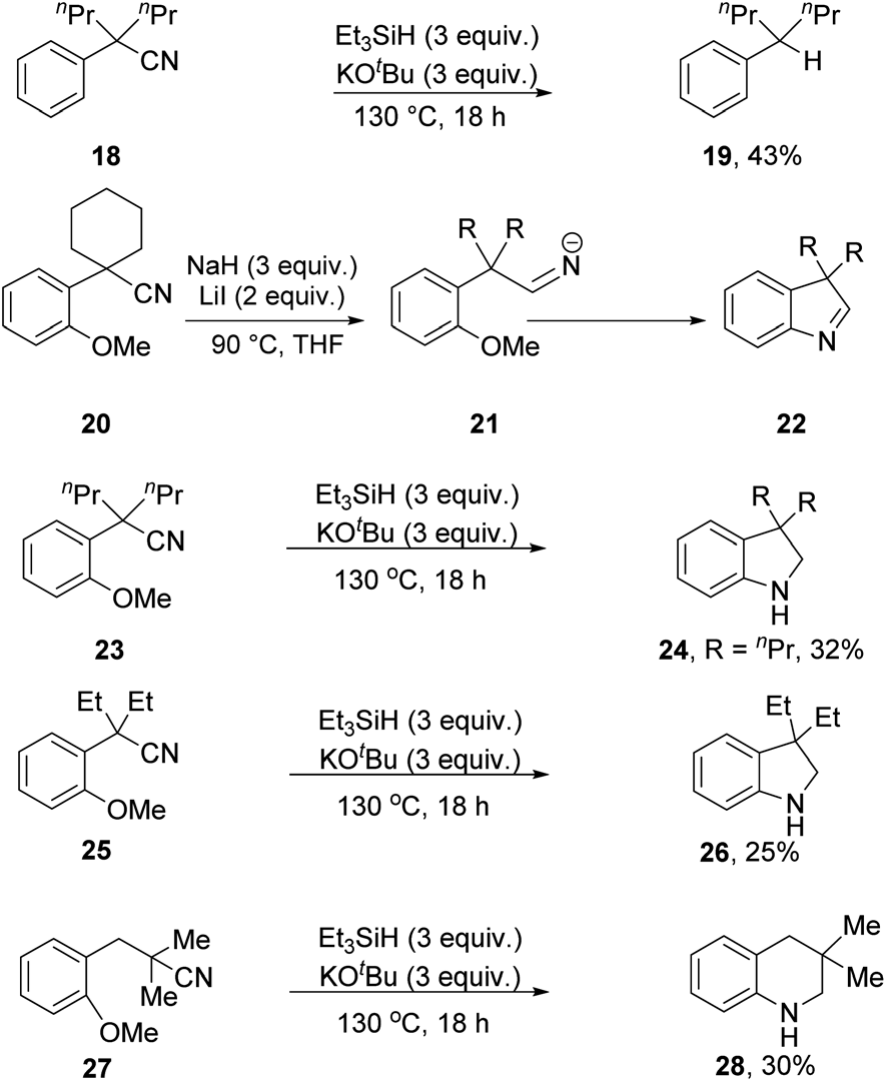


During a recent study, we showed that Et_3_SiH/KO^*t*^Bu carries out reductive decyanation of benzylic nitriles (*e.g.***18** → **19**, Scheme 2)^7,17^ and our starting point for this current study was to find out more about the reactivity of the substrates and intermediates.

In 2017, Chiba *et al.* uncovered^18^ a probe for hydride-based reduction of nitriles, where substrates *e.g.***20** reacted with a composite of NaH and LiI to form an iminyl anion **21** that displaced the *o*-MeO group in a concerted cS_N_Ar reaction^19^ to form indolenine **22** [R = (CH_2_)_4_]. In his elegant paper, aminyl anions also underwent efficient cyclisation. We wondered whether nitriles that are subjected to the Et_3_SiH + KO^*t*^Bu reagent would behave similarly, giving evidence for formation of iminyl anion intermediates through hydride ion delivery from **2**.^20^

## Results and discussion

**Scheme 3:**
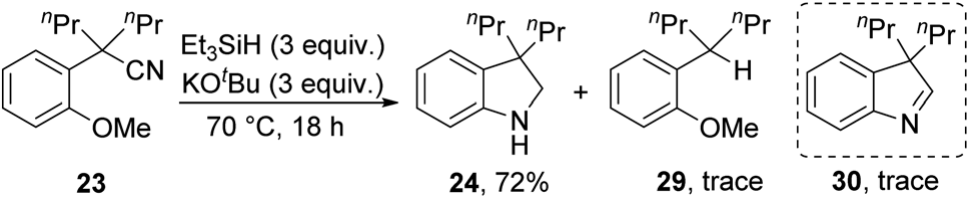


Substrates **23**, **25** and **27** (Scheme 2) were prepared (see ESI†) and reacted with the Et_3_SiH/KO^*t*^Bu mixture. In each case, cyclisation with displacement of the methoxy group was observed. Our initial conversion of **23** → **24** occurred in 32% yield (Scheme 2), but upon optimisation, the yield of **24** was increased to 72% by lowering the temperature to 70 °C (Scheme 3 and ESI†). The detection of imine **30** during the optimisation studies suggests that an iminyl anion **21** (R = ^*n*^Pr) was a key intermediate in the reactions. These reactions are therefore proposed to occur by hydride ion delivery to the nitrile by intermediate **2**. The mechanism of conversion of imines *e.g.***30** to amines (in this case, **24**) comes up for discussion later in this paper.

The effect of the identity of the base and silane present was then investigated. On changing the counter-ion on butoxide from potassium to sodium or lithium, no reaction was observed (see ESI†). Other potassium bases such as KHMDS, KOH, and KOEt were also unsuccessful. KH was somewhat successful with **24** being isolated in 12% yield, whereas NaH gave no reaction. These results underline the special reactivity of potassium *tert*-butoxide in this reagent system, which cannot be replicated by sodium *tert*-butoxide or lithium *tert*-butoxide. The effect of solvent on the reaction was also investigated, and solvent-free conditions were found to be optimal for the cyclisation (see ESI†).

The optimised conditions were then used to further study the scope of the reaction with substrates related to **23**. Firstly, the ethoxy derivative **31** was also successful, with cyclised product **24** (R = Et) isolated in 65% yield. Halide leaving groups were then tested (Table 1, entries 3–6). Interestingly, methoxide out-performed halide leaving groups for the formation of **24**, with the halides following the general trend of S_N_Ar reactivity (F > Cl > Br = I). Bromo- and iodo-substituted substrates **34** and **35** did not afford any cyclised products and instead, dehalogenated compound **37** was isolated, suggesting that dehalogenation of iodides and bromides was more rapid than activation of the nitrile. Although many mechanisms can be considered, dehalogenation is a hallmark of reactions of silyl radicals **1** or can result from electron transfer chemistry of **3**.

**Table 1:** Testing different leaving groups

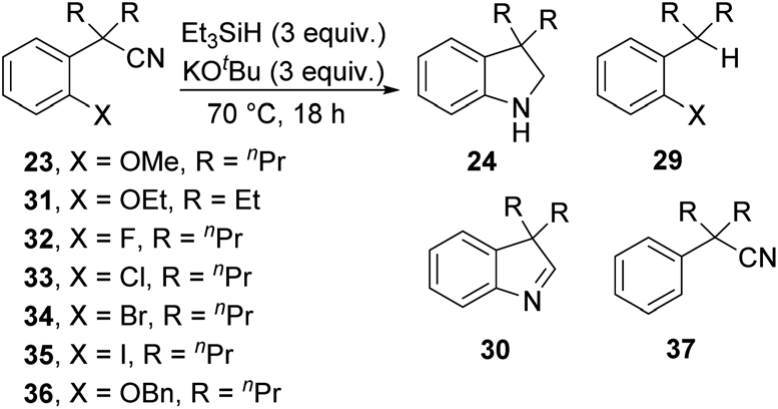					
Entry	Substrate	**24**/%	**29**/%	**30**/%	**37**/%
1	**23**	72	11	—	—
2	**31**	65	8	—	—
3	**32**	44	—	Trace	—
4	**33**	19	—	—	—
5	**34**	—	—	—	57
6	**35**	—	—	—	78

**Scheme 4:**
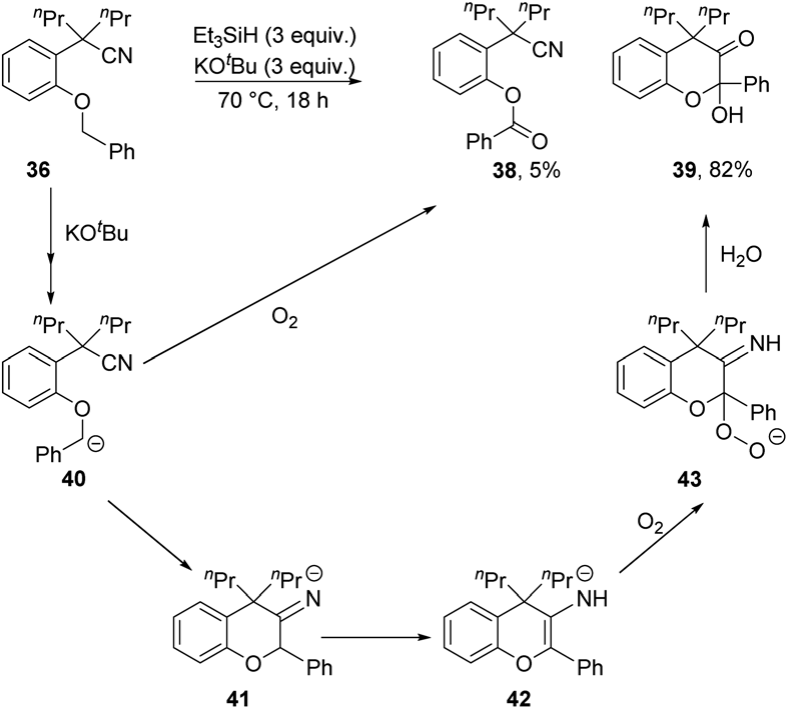


Changing the methoxy group to benzyloxy in **36** brought about different chemistry. No displacement of the benzyloxy group was detected and, instead, compounds **38** (5%) and **39** (82%) were isolated (Scheme 4). Both products suggested an initial activation at the benzyl position, most likely *via* anion **40**. Cyclisation onto the nitrile would afford **41** which would be converted to **42** through a proton shift. The electron-rich alkene in **42** will readily undergo electron transfer and coupling to molecular oxygen to afford **43**.^21–23^ If this can convert into a hydroperoxide, then reductive cleavage of the O–O bond can occur during the reaction. Otherwise, **43** could protonate on workup to a hydroperoxyketal, which can lose hydroperoxide anion in a hydrolysis that then leads to **39**. Alternatively, any residual anion **40** would also react with air on work up, ultimately leading to ester **38**.

Our next steps were to establish the necessary components for the cyclisation of **23** to **24**. Control reactions were now performed (Table 2). The parent reaction is shown as entry 1.

**Table 2:** Cyclisation reaction – mechanistic studies

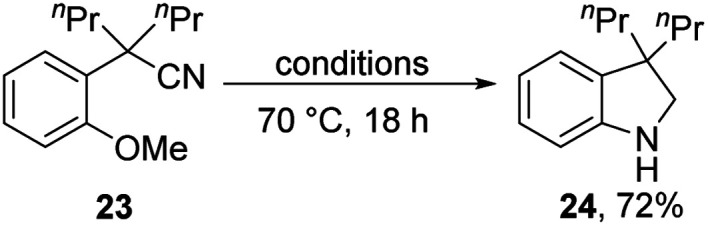		
Entry	Conditions^a^	**24**/%
1	Et_3_SiH + KO^*t*^Bu	72
2	**47** + Me_3_SiSiMe_3_ + KO^*t*^Bu	19
3	**47** + KO^*t*^Bu	Trace
4	Me_3_SiSiMe_3_ + KO^*t*^Bu	—
5	KO^*t*^Bu	—

a 3 equiv. of all reagents were used.

**Scheme 5:**
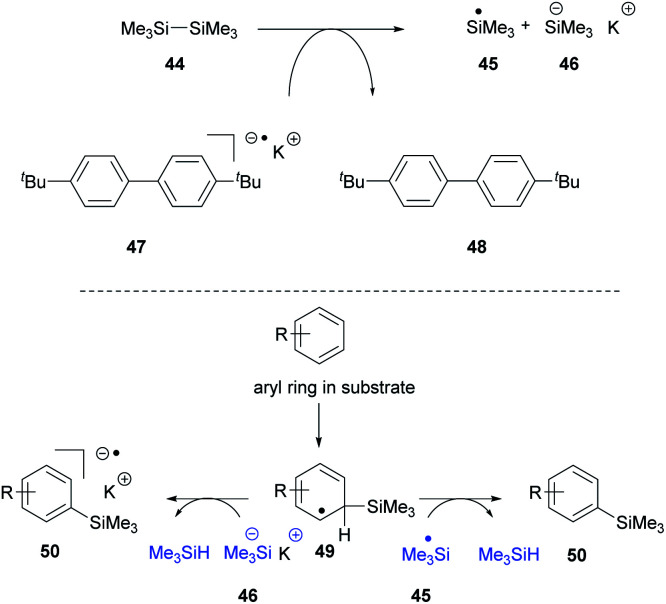


Recently, we proposed^14^ that the reactivity of the Et_3_SiH/KO^*t*^Bu couple could be reproduced in the absence of the silane, provided that an alternative source of silyl radicals was present. To this end, entry 2 shows that when the silane was replaced by the disilane **44** (Scheme 5) in the presence of the electron donor **47**,^24^ the radical anion of di-*p-tert*-butylbiphenyl, the cyclisation reaction was still observed, affording **24** (19%).

Entries 3 and 4 show that in the absence of a silyl source or an electron donor source, the reaction is not observed, while entry 5 shows that KO^*t*^Bu alone cannot bring about the reaction.

Our explanation for entry 2 is that the electron donor **47** can cleave the disilane **44** to a silyl radical **45** and a silyl anion **46** as shown in Scheme 5.^25^ The silyl radical can react with many species in solution. Notably, it can add to arene rings in the substrate to generate intermediates **49** that feature a labile H atom.^2–6,13^ This can react with a silyl radical **45** to form trimethylsilane or with silyl anion **46** to form trimethylsilane as shown in Scheme 5. This would mean that the missing trialkylsilane reagent (Me_3_SiH in this experiment) would be generated *in situ*, starting from the disilane.

**Scheme 6:**
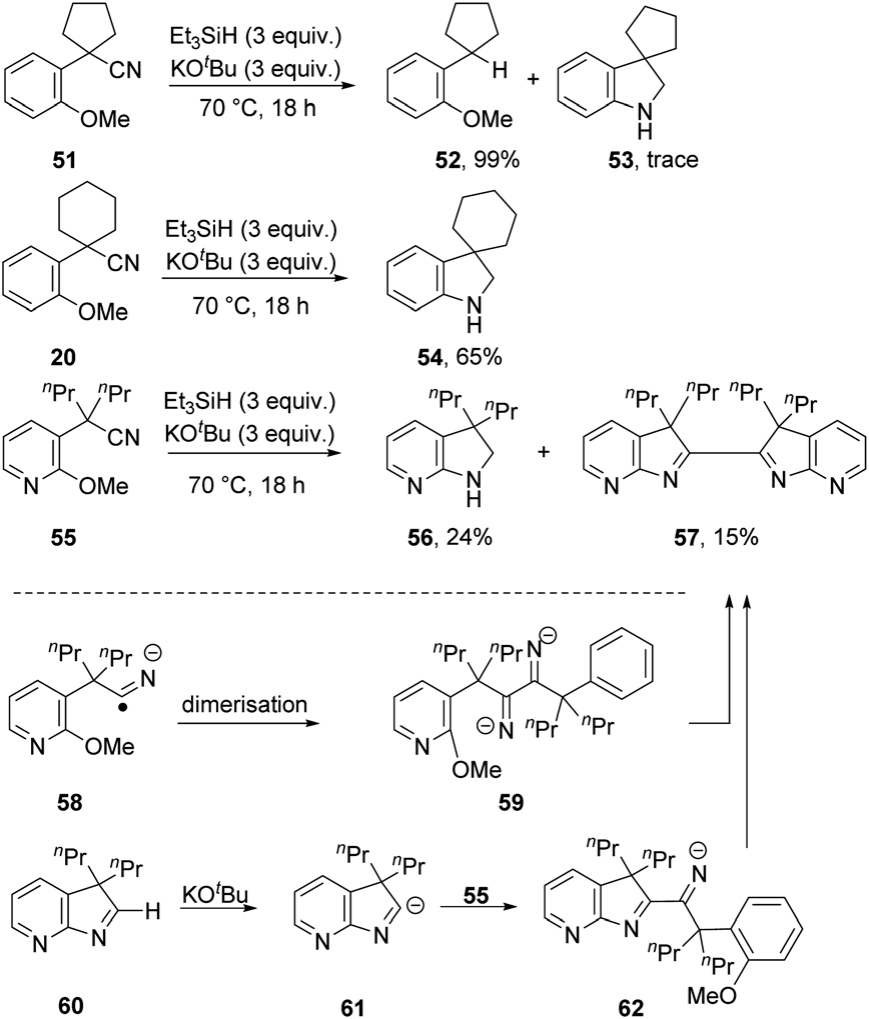


A series of substrates, **51**, **20** and **55** (Scheme 6), was now prepared and tested under the optimised conditions, with surprising results. From substrate **51**, reductive decyanation to **52** was observed in 99% yield, with only a trace amount of cyclised product **53** detected. However, from the analogous substrate **20**, cyclisation to **54** was observed in 65% yield. Pyridine-containing substrate **55** afforded product **56** (24%), along with dimer **57** (15%). This compound **57** might arise by dimerisation of radical anion **58**, *e.g.* if electron transfer occurred from radical anion **3**, followed by double cS_N_Ar cyclisation. Alternatively, and more probably, cyclic imine **60** could be deprotonated under the basic conditions to anion **61**,^26^ which could then attack another molecule of **55** to give anion **62**, which affords bis-indolenine **57** by cS_N_Ar cyclisation. Observation of this dimerisation solely for this substrate could then be attributed to enhancement of the acidity of the iminyl proton in **60** by the pyridine ring.

The results to date are consistent with bicyclic imines such as **60** as key intermediates in the formation of the final indolines, and so we were curious to probe the behaviour of related imines in the presence of the reductive silane–butoxide reagent pair.

**Scheme 7:**
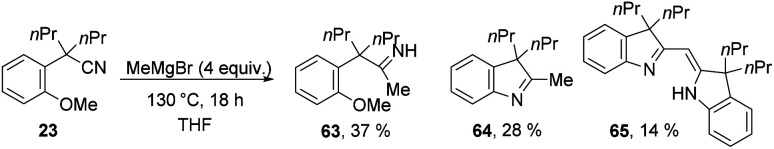


To access imines related to **30** (Scheme 3), we considered that an iminyl anion could form by addition of a Grignard reagent to a nitrile and then undergo cyclisation, in the manner of Gademann *et al.*^27^ To test this, substrate **23** was treated with MeMgBr at 70 °C, however no reaction occurred. Upon warming to 130 °C, however, products **63–65** were isolated with optimum yields arising from 4 equiv. of Grignard reagent (Scheme 7 and ESI†). Compounds **63** and **64** are indicative of the proposed mechanism for indolenine formation. Compound **65** could arise by deprotonation of the iminyl-CH_3_ group of **64** by MeMgBr, before attack onto the nitrile group of another molecule of **23**. The resulting imine anion can then undergo cS_N_Ar and tautomerism to yield **65**.

**Scheme 8:**
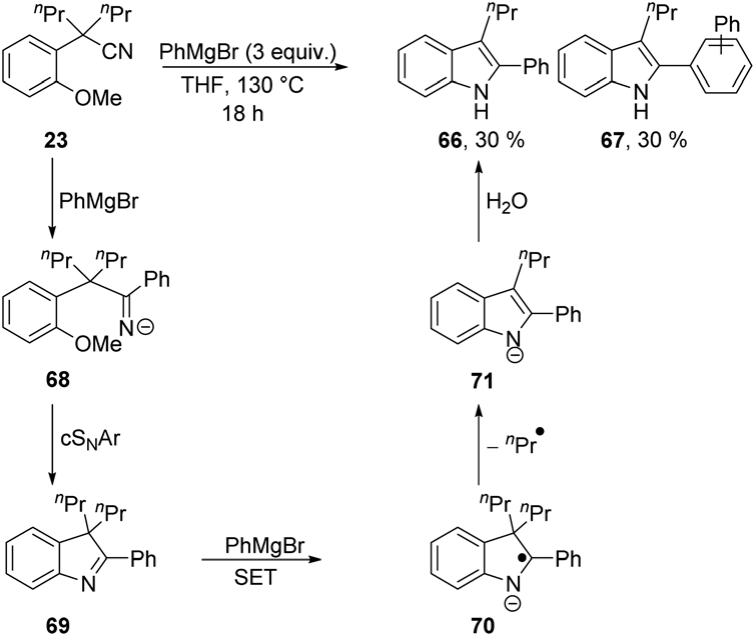


The complications in Scheme 7 leading to a low yield of **64** arose from the ease of deprotonation of the methyl group in **64**. To prevent such complications, a Grignard reagent was used that cannot be deprotonated in the α-position, *i.e.* PhMgBr. Interestingly, cyclisation to an inseparable mixture of indoles **66** and **67** (in 30% yield each, calculated by NMR internal standard) was observed (Scheme 8). This transformation shows loss of a propyl substituent, and aromatisation of the ring system to give indole products undoubtedly provides the driving force for this.

Grignard reagents have been previously reported in the literature to facilitate SET reactions to reducible substrates.^28^ Therefore, we propose that electron transfer from PhMgBr must occur to the conjugated indolenine **69**. First, PhMgBr adds to the nitrile of **23**, forming **68**, which can undergo cS_N_Ar to form **69**. Electron transfer to the conjugated indolenine **69** then occurs to form **70**, which can aromatise with loss of an alkyl group to form **71**, which ultimately protonates to **66** upon work-up. The formation of compound **67** is rationalised by the presence of phenyl radicals, generated upon single electron oxidation of PhMgBr, initiating a BHAS mechanism as previously reported in the literature.^29^

**Scheme 9:**
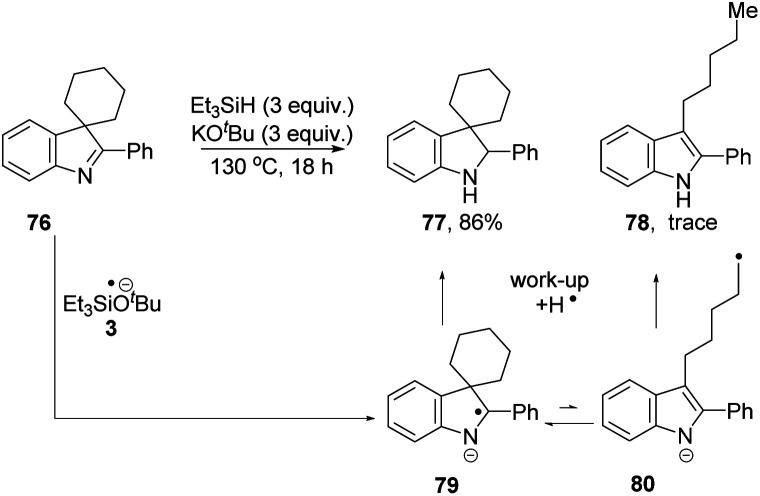


We then investigated if the reducing mixture resulting from the combination of Et_3_SiH and KO^*t*^Bu, could perform the same transformation of indolenine to indoline and thereby give evidence of electron transfer from intermediate **3**. Compound **72** was treated with Et_3_SiH and KO^*t*^Bu, and compounds **73** and **74** were isolated in 45% and 24% respectively (Table 3, entry 1). The elimination of a methyl group from **72** clearly mirrors the electron transfer reactions seen with PhMgBr. Moreover, the formation of silylated derivative **74** (an inseparable mixture of 2 regioisomers was isolated) results from the presence of triethylsilyl radicals, analogous to the phenyl radicals above. The reaction was repeated in the presence of TEMPO. The outcome was to improve the yield of indole **73** to 84% (entry 2). This outcome likely arises, at least in part, from trapping of triethylsilyl radicals by TEMPO, thereby inhibiting the formation of **74**. Two further pieces of evidence support the electron transfer proposal: (i) exposure of substrate **72** to potassium metal and KO^*t*^Bu also afforded **73** (36%), **72** (28%) and **75** (trace amounts) (entry 3); (ii) analogue **76** (Scheme 9) underwent reaction with Et_3_SiH + KO^*t*^Bu to afford principally indoline **77** (86%) together with indole **78** (trace amounts). Product **78** arises from an analogous cleavage in radical anion **79** to that seen for radical anion **70**. The pentyl side-chain of **78** shows the fate of the cleaved radical, which simply abstracts a hydrogen atom from silane or hydrogen atom donor **2**. The difference in outcome for substrates **72** and **76** relates to the fragmentation of their radical anions – when the radical anion of **72** fragments, a methyl group is lost and diffuses away from the substrate. In contrast, fragmentation of radical anion **79** sees the fragmented radical tethered to the indole structure in **80**. Radical re-addition to the indole anion reforms radical anion **79**, which then abstracts an H-atom (*e.g.* from triethylsilane or from species **2**) to give indoline **77**.

**Table 3:** Aromatisation reaction – mechanistic studies

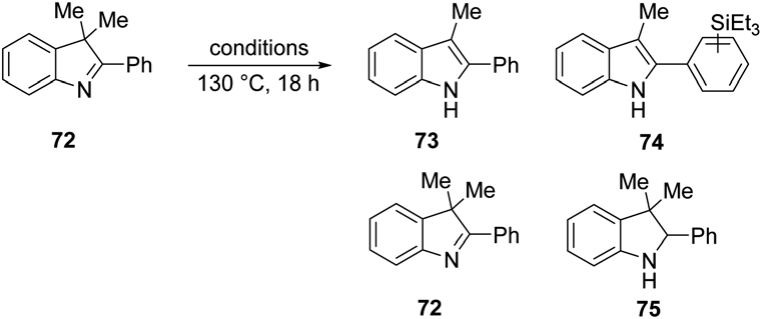					
Entry	Conditions^a^	**72**/%	**73**/%	**74**/%	**75**/%
1	Et_3_SiH + KO^*t*^Bu	—	45	24	—
2	Et_3_SiH + KO^*t*^Bu + TEMPO^b^	9	84	Trace	Trace
3	K + KO^*t*^Bu	28	36	—	Trace

a Entry 1 and 2: Et_3_SiH (3 equiv.), KO^*t*^Bu (3 equiv.), TEMPO (1 equiv.); entry 3: KO^*t*^Bu (1 equiv.), K (1.3 equiv.).

b TEMPO-SiEt_3_ was detected by GCMS (see ESI).

**Scheme 10:**
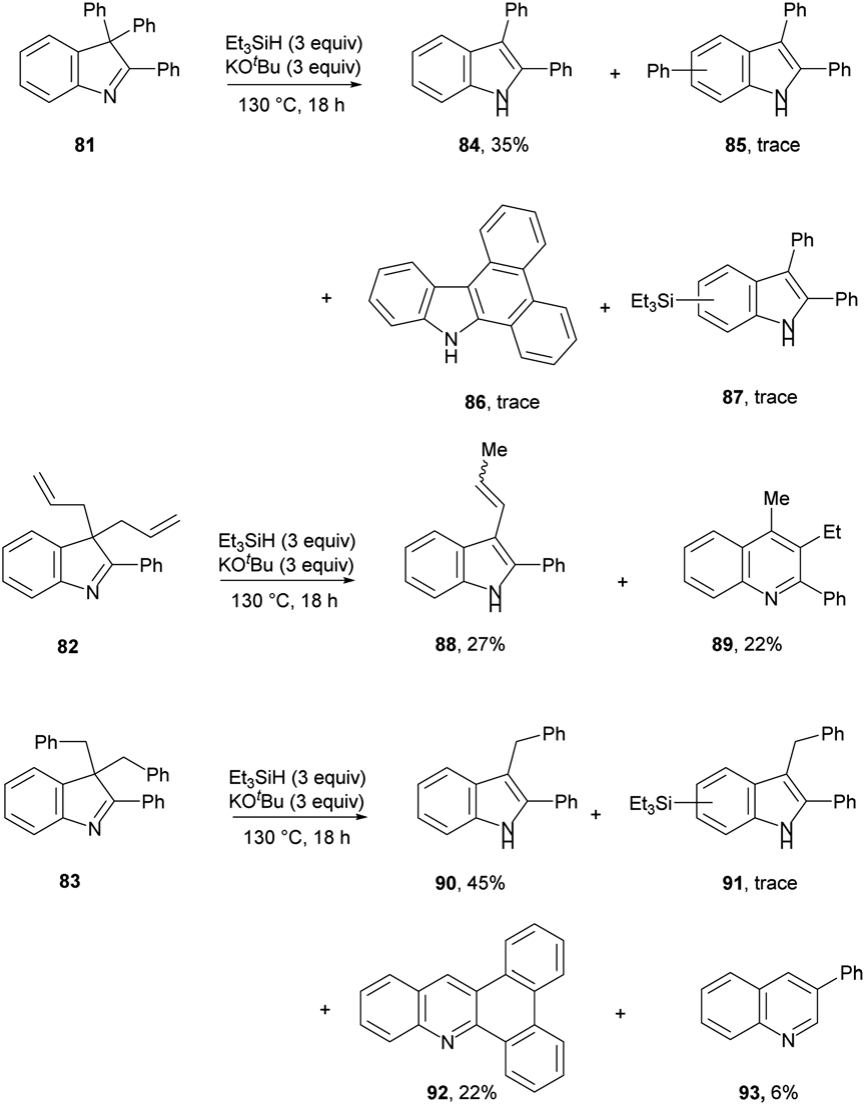


The scope of the groups that can be expelled upon aromatisation was also investigated (Scheme 10). These results show that phenyl, allyl and benzyl are feasible leaving groups.

Firstly taking substrate **81**, this mirrors the reactions of imines **69**, **72** and **76**. Loss of a phenyl radical is more difficult than loss of an alkyl radical, but indole **84** is still formed in 35% yield. Also detected were products **85** and **87**, resulting from attack on **84** by phenyl or triethylsilyl radicals and subsequent rearomatisation. Again, this mimics the addition of phenyl radicals and triethylsilyl radicals seen respectively in **67** and **74**. In addition, compound **86** was detected in an inseparable mixture with compound **85** with ^1^H NMR data and GC-MS data consistent with those previously reported in the literature. The mechanism envisaged for the formation of **86** is somewhat analogous to that for compound **92** (see below).

**Scheme 11:**
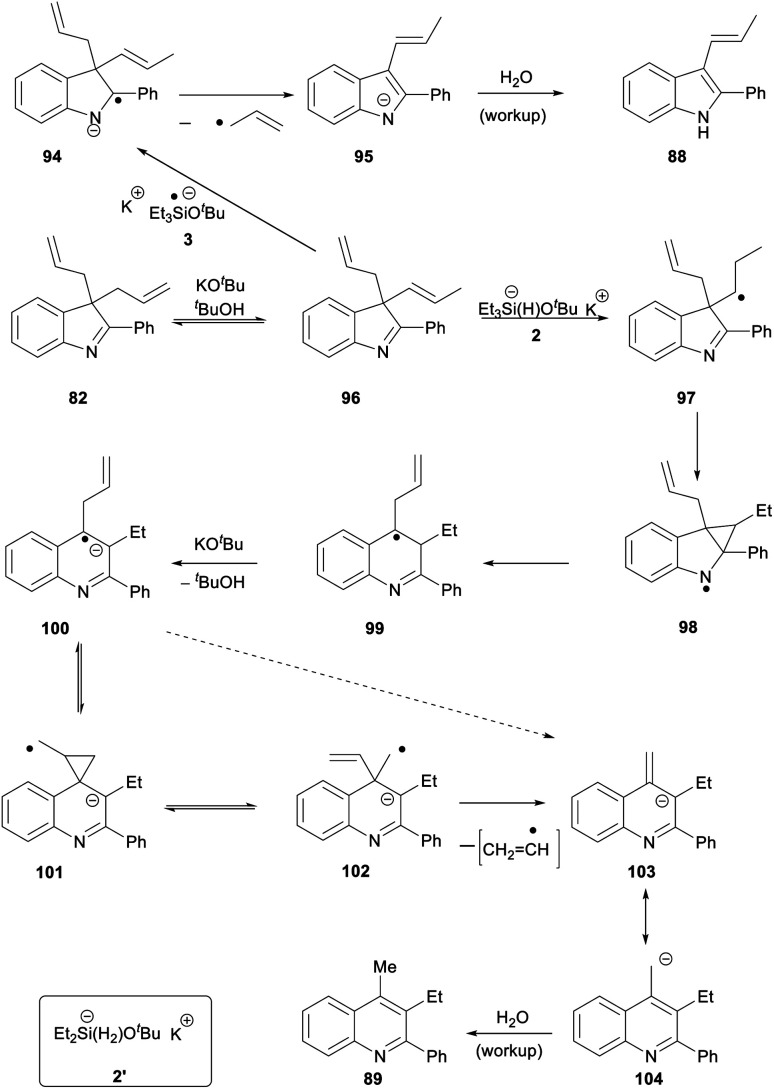


For substrate **82**, the products **88** (27%) and **89** (22%) can be explained by invoking KO^*t*^Bu-induced isomerisation of a terminal allyl group to internal alkene **96** (Scheme 11). We have recently reported that allyl groups undergo base-induced isomerisation under the Et_3_SiH/KO^*t*^Bu conditions.^14^ Subjecting this compound, **96,** to electron transfer from donor **3** gives radical anion **94**. Expulsion of an allyl radical accounts for the formation of indole **88**.

The second product formed from substrate **82** is the quinoline **89** (22%). This is a really interesting product. Focusing on the 6 carbons of the allyl substituents in **82**, it appears that one carbon has been incorporated into the ring system during a ring-expansion, two carbons have been lost during the rearrangement, and the remaining three carbons end up as the methyl and ethyl substituents on neighbouring ring carbons in **89** – a rearrangement of serious complexity. Our working hypothesis is that the product **89** arises also from intermediate diene **96**. Jeon recently demonstrated H-atom addition to styrenes by reactive intermediate **2′** (inset, Scheme 11) formed from diethylsilane. A K^+^ ion, complexed by the aromatic ring in the styrene, held the silanate anion in **2′** (and analogues) close to the aromatic ring, and this complexation was essential for H-atom addition. Here, the feasibility of H-atom addition from **2**, rather than **2′**, to a side-chain alkene should also increase when the alkene is nearer to the aromatic ring, thereby directing H-atom addition to **96** to form radical **97**. An aza-version of a cyclopropylcarbinyl rearrangement governs the ring-expansion to radical **99**. This radical has an adjacent H-atom that is easily acidic enough to be removed by KO^*t*^Bu, affording the quinoline radical anion **100**. This undergoes reversible cyclisation to cyclopropylcarbinyl radical **101**, which must very occasionally fragment to distal radical anion **102**; expulsion of a vinyl radical (or a vinyl anion) then affords benzylic anion **103** (or its benzylic radical counterpart) which affords **89** on workup.^30^

**Scheme 12:**
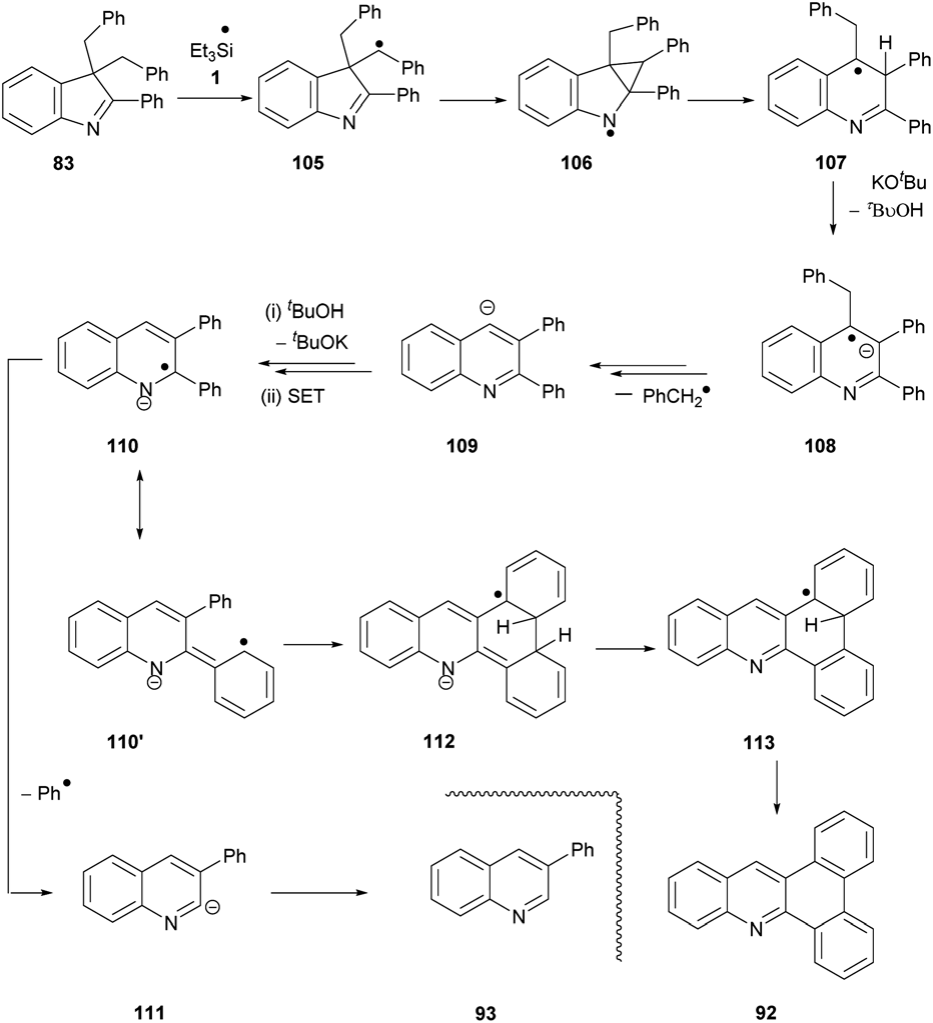


Having proposed a route to the quinoline **89** from substrate **82**, we note that two further quinolines, **92** and **93**, which arise from substrate **83**, require explanation. We have recently shown that benzylic C–H bonds can undergo abstraction of an H-atom under the conditions of these reactions, by triethylsilyl radicals **1**.^13^ In this case, this would lead to radical **105** (Scheme 12). Cyclopropylcarbinyl radical rearrangement would lead to ring-expansion to radical **107**, which, following deprotonation, would expel a benzyl radical to yield quinoline anion **109**. Protonation from ^*t*^BuOH, followed by electron transfer from **3** would give radical anion **110**. Expulsion of a phenyl radical affords anion **111** that abstracts a proton (from ^*t*^BuOH or on workup) to give **93**.

We again propose **110′** as the source of the other product, **92**. Cyclisation to the neighbouring phenyl ring gives radical anion **112**. The drive to aromaticity can then oversee the expulsion of an H atom (or a proton followed by an electron) and a hydride ion to give product **92**.

2-Phenyl-substituted indolenines **81–83** are likely to be more receptive towards electron transfer than analogues with H or alkyl groups substituted in the 2-position, but these substrates illustrate well here the array of reactive intermediates in the KO^*t*^Bu/Et_3_SiH reagent pair.

## Conclusions

The reagent pair KO^*t*^Bu + Et_3_SiH provides a unique interplay of reactive intermediates to react with substrates. This study of benzyl nitriles and indolenines features products arising from (i) hydride addition from silanate complex **2**, (ii) electron transfer from **3** (iii) hydrogen atom transfer from anion **2**, and (iv) hydrogen atom abstraction by silyl radicals **1**. The range of product types observed illustrates a unique diversity of outcomes.

## Conflicts of interest

There are no conflicts to declare.

## Supplementary Material

SC-011-D0SC04244G-s001

## References

[cit1] Fedorov A., Toutov A. A., Swisher N. A., Grubbs R. H. (2013). Chem. Sci..

[cit2] Toutov A. A., Liu W.-B., Betz K. N., Fedorov A., Stoltz B. M., Grubbs R. H. (2015). Nature.

[cit3] Toutov A. A., Liu W.-B., Betz K. N., Stoltz B. M., Grubbs R. H. (2016). Nat. Protoc..

[cit4] Liu W.-B., Schuman D. P., Yang Y.-F., Toutov A. A., Liang Y., Klare H. F. T., Nesnas N., Oestreich M., Blackmond D. G., Virgil S. C., Banerjee S., Zare R. N., Grubbs R. H., Houk K. N., Stoltz B. M. (2017). J. Am. Chem. Soc..

[cit5] Banerjee S., Yang Y.-F., Jenkins I. D., Liang Y., Toutov A. A., Liu W.-B., Schuman D. P., Grubbs R. H., Stoltz B. M., Krenske E. H., Houk K. N., Zare R. N. (2017). J. Am. Chem. Soc..

[cit6] Toutov A. A., Salata M., Fedorov A., Yang Y.-F., Liang Y., Cariou R., Betz K. N., Couzijn E. P. A., Shabaker J. W., Houk K. N., Grubbs R. H. (2017). Nat. Energy.

[cit7] Smith A. J., Young A., Rohrbach S., O'Connor E. F., Allison M., Wang H.-S., Poole D. L., Tuttle T., Murphy J. A. (2017). Angew. Chem., Int. Ed..

[cit8] (a) Toutov A. A., Betz K. N., Fedorov A., Stoltz B. M., Liu W.-B. and Grubbs R. H., US2019/0048030A1, 2019

[cit9] Asgari P., Hua Y., Thiamsiri C., Prasitwatcharakorn W., Karedath A., Chen X., Sardar S., Yum K., Leem G., Pierce B. S., Nam K., Gao J., Jeon J. (2019). Nat. Catal..

[cit10] Toutov A. A., Betz K. N., Romine A. M. and Grubbs R. H., US2019/0241587A1, 2019

[cit11] (a) Toutov A. A., Betz K. N., Romine A. M. and Grubbs R. H., US 2019/0218232A1, 2019

[cit12] Jenkins I. D., Krenske E. H. (2020). ACS Omega.

[cit13] Arokianathar J. N., Kolodziejczak K., Bugden F., Clark K., Tuttle T., Murphy J. A. (2020). Adv. Synth. Catal..

[cit14] Smith A. J., Dimitrova D., Arokianathar J. N., Kolodziejczak K., Young A., Alison M., Pole D. L., Leach S. G., Parkinson J. A., Tuttle T., Murphy J. A. (2020). Chem. Sci..

[cit15] Toutov A. A., Betz K. N., Schuman D. P., Liu W. B., Fedorov A., Stoltz B. M., Grubbs R. H. (2017). J. Am. Chem. Soc..

[cit16] Miller S. L. (1953). Science.

[cit17] Mattalia J. M. R. (2017). Beilstein J. Org. Chem..

[cit18] Kaga A., Hayashi H., Hakamata H., Oi M., Uchiyama M., Takita R., Chiba S. (2017). Angew. Chem., Int. Ed..

[cit19] Kwan E. E., Zeng Y., Besser H. A., Jacobsen E. N. (2018). Nat. Chem..

[cit20] We verified that such benzylic nitrile substrates can be cyclised also with LiAlH4 at 130 °C, although not at 70 °C, again indicative of cyclisation of an iminyl anion – See ESI†

[cit21] Murphy J. A. (2014). J. Org. Chem..

[cit22] Hanson S. S., Doni E., Traboulsee K. T., Coulthard G., Murphy J. A., Dyker C. A. (2015). Angew. Chem., Int. Ed..

[cit23] Aitken R. A., Harper A. D., Slawin A. M. Z. (2017). Synlett.

[cit24] Guijarro A., Yus M. (1993). Tetrahedron Lett..

[cit25] Däschlein C., Strohmann C. (2010). Dalton Trans..

[cit26] *N*-Methylindoles have also been proposed to be deprotonated in the 2-position by Et_3_SiH + KO^*t*^Bu^5^

[cit27] Huber F., Roesslein J., Gademann K. (2019). Org. Lett..

[cit28] Holm T., Baumann H., Lönn H., Lönngren J., Nyman H., Ottosson H. (1983). Acta Chem. Scand., Ser. B.

[cit29] Studer A., Curran D. P. (2011). Angew. Chem., Int. Ed..

[cit30] An alternative direct fragmentation from **100** → **103** is also considered. Vinyl radical fragmentations often have high energy transition states, but additional driving force is expected in converting **102** → **103** as the nitrogen-containing ring in **102** is not aromatic but becomes aromatic in **103**

