# Supplementary material for: Et3SiH + KOtBu provide multiple reactive intermediates that compete in the reactions and rearrangements of benzylnitriles and indolenines
Source: Chem Sci. 2020 Oct 21;11(45):12364–70. doi: 10.1039/d0sc04244g (PMC8162870; doi:10.1039/d0sc04244g)
Supplement: SC-011-D0SC04244G-s001 [file SC-011-D0SC04244G-s001.pdf]

Electronic Supplementary Material (ESI) for Chemical Science.
This journal is © The Royal Society of Chemistry 2020

Supplementary information for:

Et_3SiH + KO^{t}Bu Provide Multiple Reactive Intermediates that Compete in the Reactions and Rearrangements of Benzylnitriles and Indolenines

Andrew J. Smith, Daniela Dimitrova, Jude N. Arokianathar, Kenneth F. Clark, Darren L. Poole, Stuart G. Leach and John A. Murphy

General Information

Experimental Details

All solvents and reagents were used as received without any further purification. Anhydrous hexane, CH_2Cl_2, Et_2O, THF and toluene were obtained from Pure-Solv 400 solvent purification system (by Innovative Technology Inc., USA). DMF and DMSO were dried over 3 Å pre-activated molecular sieves. Molecular sieves were activated by three heating cycles in the microwave, followed by evacuation under vacuum. Powder NaH was obtained by washing a mixture of 60% NaH dispersed in mineral oil three times with anhydrous hexane under argon, followed by drying of the powder under high vacuum.

IR spectra were recorded on Shimadzu 1 IRAffinity-1 instrument.

NMR data were recorded on Bruker instruments operating at 400 MHz or 500 MHz for ^{1}H and 101 or 126 MHz for ^{13}C NMR experiments. All chemical shifts are recorded in parts per million (ppm) and coupling constants are measured in Hertz (Hz). Peak multiplicity is abbreviated as follows: s (singlet), d (doublet), t (triplet), q (quartet), sxt (sextet), hept (heptet), m (multiple) and br s (broad singlet). All spectra were referenced with respect to $CHCl_3$ peak at 7.26 ppm for ^{1}H and with respect to $CDCl_3$ peak at 77.16 ppm for ^{13}C.

High resolution mass spectrometry analysis was carried out at the National Mass Spectrometry Centre on LTQ Orbital instrument and at the University of Glasgow.

LC-MS data were recorded on Agilent Technologies 1200 series instrument utilising APCI coupled with ESI with UV detection at 254 nm. All sampled were prepared in MeOH or MeCN.

GC-MS data were recorded on Thermo Finnigan Polaris Q, mass range 50-650 Da. The column temperature was 320 °C, and the carrier gas was helium with a flow rate of 1 mL/min. The adsorbent was Crossbond® (0.25 µm) with column dimensions of 30 m x 0.25 mm. Results are reported as *m/z*. All samples were prepared in $CHCl_3$ and electron ionisation (EI) was used as the ionisation method.

General Procedures:

General Procedure A:

Substrate (1.0 eq., 0.5 mmol), Et_3SiH (3.0 eq., 1.5 mmol, 240 µL) and KO^{t}Bu (3.0 eq., 1.5 mmol, 168.32 mg) were sealed in a pressure tube in a nitrogen-filled glovebox. The contents of the pressure tube were stirred at 130 °C for 18 h before the pressure tube was cooled to room temperature, opened to air and diluted with water (50 mL). The organic products were extracted into Et_2O (3 x 50 mL). The combined organic phases were dried over Na_2SO_4, filtered and concentrated. Column chromatography was utilised as a purification method for the respective product(s) of the reaction.

General Procedure B:

Substrate (1.0 eq., 0.5 mmol) and KO^{t}Bu (3.0 eq., 1.5 mmol, 168.32 mg) were sealed in a pressure tube in a nitrogen-filled glovebox. The contents of the pressure tube were stirred at 130 °C for 18 h before the pressure tube was cooled to room temperature, opened to air and diluted with water (50 mL). The organic products were extracted into Et_2O (3 x 50 mL). The combined organic phases were dried over Na_2SO_4, filtered and concentrated. Column chromatography was utilised as a purification method for the respective product(s) of the reaction.

General Procedure C:

3,3-Dimethyl-2-phenyl-3*H*-indole (1.0 eq., 0.5 mmol, 110.65 mg) and RMgX (2.0 eq., 1 mmol, 1 mL or 500 µL) as a 1 M or 2 M solution in THF was added to a pressure tube in a nitrogen-filled glovebox. The total volume of solvent was made up to 1.5 mL of dry THF and the pressure tube was sealed and heated to 130 °C for 18 h. The reaction mixture was then cooled to room temperature, quenched with water (30 mL) at 0 °C and extracted with Et_2O (3 x 50 mL). The combined organic layers were dried over Na_2SO_4, filtered and concentrated.

General Procedure D:

A solution of an appropriate ketone (1.0 eq., 10 mmol or 7 mmol), phenylhydrazine (1.0 eq., 10 mmol or 7 mmol) and AcOH (10 mL) was refluxed under argon for 16 h. The reaction mixture was cooled to room temperature, poured onto sat. Na_2CO_3 (100 mL) and extracted with CH_2Cl_2 (4 x 40 mL). The combined organic layers were dried over $MgSO_4$, filtered and concentrated. Purification by column chromatography afforded the corresponding indolenine product.[1]

General Procedure E:

*i*PrMgCl (3.0 eq., 30 mmol, 15 mL) as a 2 M solution in THF was added dropwise to a solution of an appropriate benzonitrile (1.0 eq., 10 mmol) in dry THF (10 mL) at 0 °C. CuBr (1 mol%, 0.1 mmol, 14.35 mg) was added under argon and the reaction mixture was refluxed for 4 h before it was cooled to

0 °C and quenched by dropwise addition of water (11 mL). The reaction was then diluted with 1 M H_2SO_4 (36 mL) and the resulting yellow/green mixture was refluxed for 1 h before it was cooled to 0 °C and basified with 2 M NaOH until pH = 9 – 10. The reaction mixture was then extracted with EtOAc (3 x 100 mL) and the combined organic phases were dried over $MgSO_4$, filtered and concentrated. The respective ketone product was used without any further purification.[2]

Substrate synthesis:

Preparation of 2-phenyl-2-propylpentanenitrile (**18**)

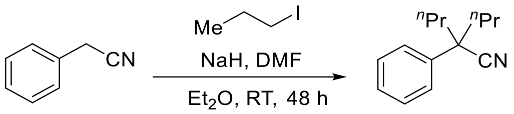

18, 79%

This substrate was prepared according to a modified literature procedure.[3] To a suspension of NaH (227 mg, 9.45 mmol, 3.15 equiv.) in dry DMF (11 mL) was added a solution of benzyl cyanide (0.35 mL, 3 mmol, 1 equiv.) and 1-iodopropane (1.32 mL, 13.5 mmol, 4.5 equiv.) in dry diethyl ether (5.5 mL) at room temperature. This mixture was stirred for 48 h at room temperature and was then quenched with methanol and concentrated under reduced pressure. The crude residue was redissolved in dry diethyl ether, and then washed with water, sodium bisulfite solution and sodium carbonate solution. The organic layer was then dried over Na_2SO_4, filtered and concentrated. Purification by column chromatography (hexane:diethyl ether, 49:1) afforded 2-phenyl-2-propylpentanenitrile **18** as a colourless oil (476 mg, 79%). **^{1}H NMR** (400 MHz, $CDCl_3$) 0.89 (t, *J* = 7.5 Hz, 6 H, 2 x CH_3), 1.05 - 1.24 (m, 2 H, 2 x CH), 1.40 - 1.58 (m, 2 H, 2 x CH), 1.81-1.89 (ddd, *J* = 13.6, 12.3, 4.8 Hz, 2 H, 2 x CH), 1.92-2.00 (ddd, *J* = 13.3, 12.0, 4.5 Hz, 2 H, 2 x CH), 7.25 - 7.34 (m, 1 H, ArH), 7.35 - 7.44 (m, 4 H, 4 x ArH). **^{13}C NMR** (101 MHz, $CDCl_3$) 13.9, 18.6, 43.2, 48.3, 122.7, 125.9, 127.5, 128.8, 138.8. **ATR-IR ν_{max}** (neat)/cm^{-1} 2958, 2872, 2233, 1600, 1492, 1463, 1448, 1379, 1201, 1112, 1083, 1029, 912, 763, 738, 698. ***m/z* (EI):** 201.2 (M^+, 25), 159.1 (77), 130.1 (78), 116.1 (100), 103.1 (48), 91.1 (14), 77.1 (16). **HRMS (CI):** calcd. for $C_{14}H_{20}N^+$ ($[M+H]^+$): 202.1596, found: 202.1594.

Preparation of 2-(2-methoxyphenyl)-2-propylpentanenitrile (**23**)

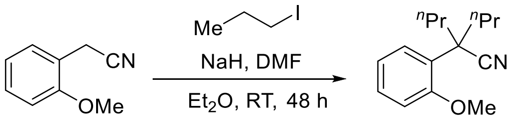

23, 81%

This substrate was prepared according to a modified literature procedure.[3] To a suspension of NaH (227 mg, 9.45 mmol, 3.15 equiv.) in dry DMF (11 mL) was added a solution 2-methoxyphenylacetonitrile (442 mg, 3 mmol, 1 equiv.) and 1-iodopropane (1.32 mL, 13.5 mmol, 4.5 equiv.) in dry diethyl ether (5.5 mL) at room temperature. This mixture was stirred for 48 h at room temperature and was then quenched with methanol and concentrated under reduced pressure. The crude residue was redissolved

in diethyl ether, and then washed with water, sodium bisulfite solution and sodium carbonate solution. The organic layer was then dried over Na_2SO_4, filtered and concentrated. Purification by column chromatography (hexane:diethyl ether, 49:1) afforded 2-(2-methoxyphenyl)-2-propylpentanenitrile **23** as a colourless oil (559 mg, 81%). **^{1}H NMR** (400 MHz, $CDCl_3$) 0.89 (t, *J* = 7.3 Hz, 6 H, 2 x CH_3), 1.02 - 1.22 (m, 2 H, 2 x CH), 1.38 - 1.51 (m, 2 H, 2 x CH), 1.91 (ddd, *J* = 13.5, 12.0, 4.9 Hz, 2 H, 2 x CH), 2.27 (ddd, *J* = 13.5, 12.2, 4.7 Hz, 2 H, 2 x CH), 3.85 (s, 3 H, OCH_3), 6.91 (dd, *J* = 8.3, 1.0 Hz, 1 H, ArH), 6.96 (td, *J* = 7.6, 1.5 Hz, 1 H, ArH), 7.25 - 7.33 (m, 1 H, ArH), 7.52 (dd, *J* = 7.8, 1.5 Hz, 1 H, ArH). **^{13}C NMR** (101 MHz, $CDCl_3$) 14.0, 19.0, 39.7, 48.4, 55.3, 111.8, 120.7, 123.7, 125.6, 129.0, 129.5, 157.0. **ATR-IR ν_{max}** (neat)/cm^{-1} 2958, 2931, 2872, 2231, 1583, 1492, 1463, 1435, 1242, 1097, 1024, 788. ***m/z* (EI):** 231.2 (M^+, 34), 188.1 (100), 161.1 (85), 146.1 (63), 131.1 (9), 116.1 (16), 105.1 (9), 91.1 (22), 77.1 (9), 65.1 (3), 51.0 (3). **HRMS (CI)** calcd. for $C_{15}H_{22}NO^+$ ($[M+H]^+$): 232.1696, found: 232.1696.

Preparation of 2-(2-fluorophenyl)-2-propylpentanenitrile (**32**)

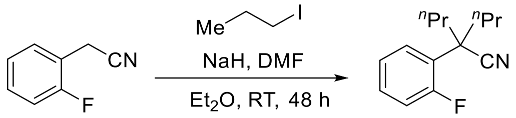

32, 79%

This substrate was prepared according to a modified literature procedure.[3] To a solution of sodium hydride (60% dispersion in mineral oil, 630 mg, 15.75 mmol, 3.15 equiv.) in dry DMF (15 mL), under nitrogen, was added a mixture of 2-(2-fluorophenyl)acetonitrile (676 mg, 5 mmol, 1 equiv.) and 1-iodopropane (2.19 mL, 22.5 mmol, 4.5 equiv.) in dry diethyl ether (7.5 mL) at room temperature. The resulting mixture was stirred for 48 h at room temperature and then quenched by slow addition of methanol. The resulting mixture was concentrated under reduced pressure, redissolved in diethyl ether and washed with water, sodium bisulfite solution and sodium carbonate solution. The organic layer was then dried over a hydrophobic frit and concentrated under reduced pressure. Purification by column chromatography (cyclohexane:ethyl acetate, 100:0 → 95:5) afforded 2-(2-fluorophenyl)-2-propyl pentanenitrile **32** as a colourless oil (866 mg, 79%). **^{1}H NMR** (400 MHz, $CDCl_3$) 0.91 (t, *J* = 7.1 Hz, 6 H, 2 x CH_3), 1.05 - 1.23 (m, 2 H, 2 x CH), 1.41 - 1.59 (m, 2 H, 2 x CH), 1.96 (td, *J* = 12.8, 4.9 Hz, 2 H, 2 x CH), 2.07 - 2.21 (m, 2 H, 2 x CH), 7.05 (ddd, *J* = 12.8, 8.4, 1.5 Hz, 1 H, ArH), 7.16 (td, *J* = 7.5, 1.5 Hz, 1 H, ArH), 7.27 - 7.35 (m, 1 H, ArH), 7.59 (td, *J* = 8.0, 1.7 Hz, 1 H, ArH). **^{13}C NMR** (101 MHz, $CDCl_3$) 13.8 (s), 19.0 (s), 40.7 (d, *J* = 3.9 Hz), 48.0 (d, *J* = 3.9 Hz), 116.6 (d, *J* = 23.1 Hz), 122.3 (s), 124.3 (d, *J* = 3.1 Hz), 125.1 (d, *J* = 10.8 Hz), 129.7 (d, *J* = 9.3 Hz), 130.0 (d, *J* = 4.6 Hz), 160.0 (d, *J* = 248.1 Hz). **ATR-IR ν_{max}** (neat)/cm^{-1} 2963, 2934, 2875, 2233, 1578, 1491, 1446, 1222, 1091, 805, 757, 545, 484. ***m/z* (EI):** 219.2 (M^+, 19), 190.1 (7), 177.1 (75), 148.1 (100), 134.1 (55), 121.1 (43), 110.0 (14), 101.1 (14), 75.1 (6). **HRMS (CI)** calcd. for $C_{14}H_{19}FN^+$ ($[M+H]^+$): 220.1502, found: 220.1507.

Preparation of 2-(2-chlorophenyl)-2-propylpentanenitrile (**33**)

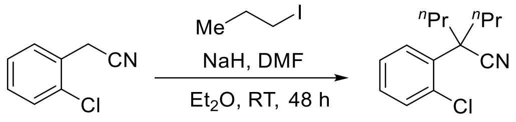

33, 100 %

This substrate was prepared according to a modified literature procedure.[3] To a solution of sodium hydride (60% dispersion in mineral oil, 630 mg, 15.75 mmol, 3.15 equiv.) in dry DMF (15 mL), under nitrogen, was added a mixture of 2-(2-chlorophenyl)acetonitrile (758 mg, 5 mmol, 1 equiv.) and 1-iodopropane (2.19 mL, 22.5 mmol, 4.5 equiv.) in dry diethyl ether (7.5 mL) at room temperature. The resulting mixture was stirred for 48 h at room temperature and then quenched by slow addition of methanol. The resulting mixture was concentrated under reduced pressure, redissolved in diethyl ether and washed with water, sodium bisulfite solution and sodium carbonate solution. The organic layer was then dried over a hydrophobic frit and concentrated under reduced pressure. Purification by column chromatography 247 (cyclohexane:ethyl acetate, 100:0 → 95:5) afforded 2-(2-chlorophenyl)-2-propylpentanenitrile **33** as a colourless oil (1.18 g, 100%). **^{1}H NMR** (400 MHz, $CDCl_3$) 0.92 (t, J = 7.5 Hz, 6 H, 2 x CH_3), 1.04 - 1.24 (m, 2 H, 2 x CH), 1.37 - 1.55 (m, 2 H, 2 x CH), 1.99 (ddd, J = 13.9, 12.1, 4.6 Hz, 2 H, 2 x CH), 2.51 (ddd, J = 13.9, 12.3, 4.6 Hz, 2 H, 2 x CH), 7.23 - 7.32 (m, 2 H, 2 x ArH), 7.35 - 7.41 (m, 1 H, ArH), 7.68 - 7.73 (m, 1 H, ArH). **^{13}C NMR** (101 MHz, $CDCl_3$) 13.9, 19.0, 39.4, 50.3, 123.0, 127.1, 129.1, 131.1, 131.8, 132.2, 134.1. **ATR-IR ν_{max}** (neat)/cm^{-1} 2961, 2933, 2874, 22334, 1569, 1467, 1430, 1120, 1039, 758, 466. ***m/z*** **(EI):** 237.1 (M^+, 7), 235.1 (M^+, 21), 208.1 (3), 206.1 (9), 195.1 (22), 193.1 (69), 166.0 (38), 164.1 (100), 152.0 (17), 150.0 (48), 139.0 (13), 137.0 (36), 128.1 (21), 115.1 (12), 101.1 (16), 89.1 (6), 77.1 (9), 63.1 (4), 51.1 (6). **HRMS (CI)** calcd. for $C_{14}H_{19}ClN^+$ ($[M+H]^+$): 236.1206 and 238.1180, found: 236.1211 and 238.1184.

Preparation of 2-(2-bromophenyl)-2-propylpentanenitrile (**34**)

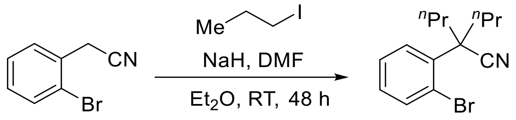

34, 100%

This substrate was prepared according to a modified literature procedure.[3] To a solution of sodium hydride (60% dispersion in mineral oil, 630 mg, 15.75 mmol, 3.15 equiv.) in dry DMF (15 mL), under nitrogen, was added a mixture of 2-(2-bromophenyl)acetonitrile (0.65 mL, 5 mmol, 1 equiv.) and 1-iodopropane (2.19 mL, 22.5 mmol, 4.5 equiv.) in dry diethyl ether (7.5 mL) at room temperature. The resulting mixture was stirred for 48 h at room temperature and then quenched by slow addition of methanol. The resulting mixture was concentrated under reduced pressure, redissolved in diethyl ether and washed with water, sodium bisulfite solution and sodium carbonate solution. The organic layer was then dried over a hydrophobic frit and concentrated under reduced pressure. Purification by column chromatography (cyclohexane:ethyl acetate, 100:0 → 95:5) afforded 2-(2-bromophenyl)-2-propyl

pentanenitrile **34** as a colourless oil (1.18 g, 100%). **^{1}H NMR** (400 MHz, $CDCl_3$) 0.92 (t, *J* = 7.3 Hz, 6 H, 2 x CH_3), 1.04 - 1.22 (m, 2 H, 2 x CH), 1.35 - 1.54 (m, 2 H, 2 x CH) 1.97 (ddd, *J* = 14.1, 12.2, 4.6 Hz, 2 H, 2 x CH), 2.61 (ddd, *J* = 13.9, 12.3, 4.6 Hz, 2 H, 2 x CH), 7.17 (m, 1 H, ArH), 7.33 (td, *J* = 7.4, 1.5 Hz, 1 H, ArH), 7.61 (dd, *J* = 7.9, 1.5 Hz, 1 H, ArH), 7.72 (dd, *J* = 8.1, 1.7 Hz, 1 H, ArH). **^{13}C NMR** (101 MHz, $CDCl_3$) 13.9, 19.0, 39.3, 50.9, 120.3, 123.0, 127.6, 129.3, 131.6, 135.4, 135.9. **ATR-IR ν_{max}** (neat)/cm^{-1} 2960, 2932, 2873, 2233, 1565, 1469, 1426, 1020, 757, 661, 532, 459. ***m/z* (EI):** 281.1 (M^+, 23), 279.1 (M^+, 23), 252.0 (9), 250.1 (10), 239.1 (70), 237.1 (74), 210.0 (98), 208.0 (100), 195.9 (45), 194.0 (47), 183.0 (30), 181.0 (31), 169.0 (8), 158.1 (12), 140.1 (9), 129.1 (33), 115.1 (37), 102.1 (32), 89.0 (13), 77.1 (20), 63.1 (9), 51.1 (15). **HRMS (CI)** calcd. for $C_{14}H_{19}BrN^+$ ($[M+H]^+$): 280.0701 and 282.0681, found: 280.0703 and 282.0684.

Preparation of 2-(2-iodophenyl)-2-propylpentanenitrile (**35**)

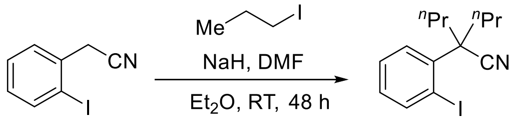

35, 84%

This substrate was prepared according to a modified literature procedure.[3] To a solution of sodium hydride (60% dispersion in mineral oil, 630 mg, 15.75 mmol, 3.15 equiv.) in dry DMF (15 mL), under nitrogen, was added a mixture of 2-(2-iodophenyl)acetonitrile (0.69 mL, 5 mmol, 1 equiv.) and 1-iodopropane (2.19 mL, 22.5 mmol, 4.5 equiv.) in dry diethyl ether (7.5 mL) at room temperature. The resulting mixture was stirred for 48 h at room temperature and then quenched by slow addition of methanol. The resulting mixture was concentrated under reduced pressure, redissolved in diethyl ether and washed with water, sodium bisulfite solution and sodium carbonate solution. The organic layer was then dried over a hydrophobic frit and concentrated under reduced pressure. Purification by column chromatography (cyclohexane:ethyl acetate, 100:0 → 95:5) afforded 2-(2-iodophenyl)-2-propyl pentanenitrile **35** as a white solid (1.38 g, 84%). **Mp** = 79-81 °C (lit. mp = 71-73 °C).[4] **^{1}H NMR** (400 MHz, $CDCl_3$) 0.93 (t, *J* = 7.4 Hz, 6 H, 2 x CH_3), 1.04 - 1.23 (m, 2 H, 2 x CH), 1.35 - 1.53 (m, 2 H, 2 x CH), 1.92 (ddd, *J* = 14.2, 12.2, 4.7 Hz, 2 H, 2 x CH), 2.70 (ddd, *J* = 14.0, 12.3, 4.7 Hz, 2 H, 2 x CH), 6.97 (td, *J* = 7.5, 1.7 Hz, 1 H, ArH), 7.36 (td, *J* = 7.6, 1.0 Hz, 1 H, ArH), 7.68 (dd, *J* = 8.1, 1.7 Hz, 1 H, ArH), 8.00 (dd, *J* = 7.9, 1.5 Hz, 1 H, ArH). **^{13}C NMR** (101 MHz, $CDCl_3$) 13.9, 18.9, 39.2, 51.3, 92.1, 123.1, 128.2, 129.3, 131.6, 137.7, 143.7. **ATR-IR ν_{max}** (neat)/cm^{-1} 2952, 2926, 2870, 2228, 1463, 1423, 1377, 1279, 1170, 1105, 1008, 764, 731, 719, 652, 528, 454. ***m/z* (EI):** 327.1 (M^+, 48), 298.1 (8), 285.1 (64), 256.0 (100), 241.9 (42), 228.9 (31), 214.9 (5), 200.2 (4), 157.1 (20), 142.1 (13), 129.1 (39), 115.1 (42), 102.1 (25), 77.0 (16), 63.1 (8), 51.0 (11). **HRMS (CI)** calcd. for $C_{14}H_{19}IN^+$ ($[M+H]^+$): 328.0562, found: 328.0559. The data for this compound are consistent with those previously reported in the literature.[4]

Preparation of 2-(2-(benzyloxy)phenyl)-2-propylpentanenitrile (**36**)

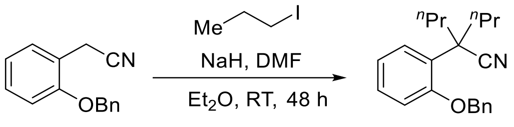

36, 64%

This substrate was prepared according to a modified literature procedure.[3] To a solution of sodium hydride (60% dispersion in mineral oil, 630 mg, 15.75 mmol, 3.15 equiv.) in dry DMF (15 mL), under nitrogen, was added a mixture of 2-(2-benzyloxy)phenylacetonitrile (1.12 g, 5 mmol, 1 equiv.) and 1-iodopropane (2.19 mL, 22.5 mmol, 4.5 equiv.) in dry diethyl ether (7.5 mL) at room temperature. The resulting mixture was stirred for 48 h at room temperature and then quenched by slow addition of methanol. The resulting mixture was concentrated under reduced pressure, redissolved in diethyl ether and washed with water, sodium bisulfite solution and sodium carbonate solution. The organic layer was then dried over a hydrophobic frit and concentrated under reduced pressure. Purification by column chromatography (cyclohexane:ethyl acetate, 100:0 → 9:1) afforded 2-(2-(benzyloxy)phenyl)-2-propyl pentanenitrile **36** as a white solid (979 mg, 64%). **Mp** = 94-97 °C. **^{1}H NMR** (400 MHz, $CDCl_3$) 0.90 (t, *J* = 7.4 Hz, 6 H, 2 x CH_3), 1.11 - 1.26 (m, 2 H, 2 x CH), 1.40 - 1.57 (m, 2 H, 2 x CH), 1.93 (ddd, *J* = 12.3, 4.4, 1.0 Hz, 2 H, 2 x CH), 2.36 (ddd, *J* = 12.3, 5.0, 1.0 Hz, 2 H, 2 x CH), 5.14 (s, 2 H, CH2), 6.96 - 7.06 (m, 2 H, 2 x ArH), 7.27 - 7.34 (m, 1 H, ArH), 7.37 - 7.43 (m, 1 H, ArH), 7.43 - 7.48 (m, 4 H, 4 x ArH), 7.61 (dd, *J* = 7.9, 1.5 Hz, 1 H, ArH). **^{13}C NMR** (101 MHz, $CDCl_3$) 13.9, 19.0, 39.7, 48.6, 70.4, 112.6, 120.8, 123.6, 125.5, 127.5, 128.1, 128.6, 128.9, 129.8, 136.5, 156.0. **ATR-IR ν_{max}** (neat)/cm^{-1} 2956, 2870, 2233, 1596, 1489, 1445, 1268, 1220, 1097, 1019, 787, 756, 737, 491. **HRMS (CI)** calcd. for $C_{21}H_{26}NO^+$ ($[M+H]^+$): 308.2014, found: 308.2014.

Preparation of 1-(2-methoxyphenyl)cyclopentane-1-carbonitrile (**51**)

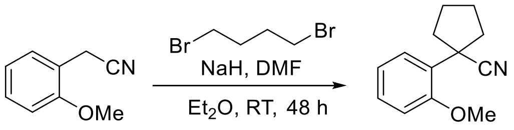

51, 85%

To a suspension of oil-free sodium hydride (378 mg, 15.75 mmol, 3.15 equiv.) in DMF (18 mL) under nitrogen was added a solution of 2-methoxyphenylacetonitrile (736 mg, 5 mmol, 1 equiv.) and 1,4-dibromobutane (2.69 mL, 22.5 mmol, 4.5 equiv.) in diethyl ether (9 mL) at room temperature. The resulting mixture was stirred at room temperature for 48 h, then quenched by addition of methanol. The resulting mixture was concentrated under reduced pressure and redissolved in diethyl ether. The solution was washed with water, sodium bisulfite and saturated sodium carbonate solution, then dried over Na_2SO_4, filtered and concentrated under reduced pressure. Purification by column chromatography (hexane:ethyl acetate, 100:0 → 95:5) afforded 1-(2-methoxyphenyl)cyclopentane-1-carbonitrile **51** as a white solid (854 mg, 85%). **Mp** = 71-73 °C (lit. mp = 67-68 °C).[5] **^{1}H NMR** (400 MHz, $CDCl_3$) 1.80 - 1.94 (m, 2 H, 2 x CH), 1.94 - 2.07 (m, 2 H, 2 x CH), 2.07 - 2.18 (m, 2 H, 2 x CH), 2.48 - 2.61 (m, 2

H, 2 x CH), 3.93 (s, 3 H, OCH_3), 6.90 - 7.00 (m, 2 H, 2 x ArH), 7.28 - 7.36 (m, 2 H, 2 x ArH). **^{13}C NMR** (101 MHz, $CDCl_3$) 23.8, 37.7, 44.7, 55.5, 111.7, 120.4, 124.5, 126.5, 127.5, 129.3, 157.6. **ATR-IR ν_{max}** (neat)/cm^{-1} 2962, 2916, 2223, 1597, 1490, 1462, 1435, 1294, 1253, 1126, 1056, 1024, 952, 943, 898, 786, 653. ***m/z* (EI):** 201.1 (M^+, 59), 186.1 (18), 172.1 (18), 159.1 (51), 144.1 (100), 131.1 (22), 116.1 (27), 103.1 (15), 89.0 (19), 77.0 (16), 63.0 (11), 51.0 (9). The data for this compound are consistent with those previously reported in the literature.[5]

Preparation of 1-(2-methoxyphenyl)cyclohexane-1-carbonitrile (**20**)

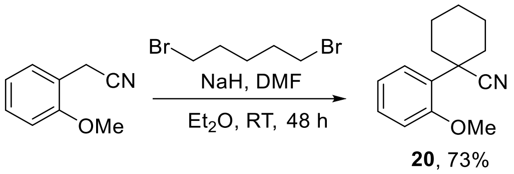

20, 73%

To a suspension of oil-free sodium hydride (378 mg, 15.75 mmol, 3.15 equiv.) in DMF (18 mL) under nitrogen was added a solution of 2-methoxyphenylacetonitrile (736 mg, 5 mmol, 1 equiv.) and 1,5-dibromopentane (3.06 mL, 22.5 mmol, 4.5 equiv.) in diethyl ether (9 mL) at room temperature. The resulting mixture was stirred at room temperature for 48 h, then quenched by addition of methanol. The resulting mixture was concentrated under reduced pressure and redissolved in diethyl ether. The solution was washed with water, sodium bisulfite and saturated sodium carbonate solution, then dried over Na_2SO_4, filtered and concentrated under reduced pressure. Purification by column chromatography (hexane:ethyl acetate, 100:0 → 9:1), followed by recrystallisation from hexane, afforded 1-(2-methoxyphenyl)cyclohexane-1-carbonitrile **20** as a white solid (796 mg, 73%). **Mp** = 101-103 °C (lit. mp = 102-103 °C).[5] **^{1}H NMR** (400 MHz, $CDCl_3$) 1.17 - 1.34 (m, 1 H, CH), 1.78 (td, J = 13.0, 3.5 Hz, 2 H, 2 x CH), 1.81 - 1.97 (m, 5 H, 5 x CH), 2.38 (d, J = 12.1 Hz, 2 H, 2 x CH), 3.93 (s, 3 H, OCH_3), 6.95 - 7.00 (m, 2 H, 2 x ArH), 7.29 - 7.36 (m, 2 H, 2 x ArH). **^{13}C NMR** (101 MHz, $CDCl_3$) 23.3, 25.3, 34.5, 40.8, 55.5, 112.2, 120.8, 122.5, 125.9, 129.1, 129.1, 157.6. **ATR-IR ν_{max}** (neat)/cm^{-1} 2931, 2860, 2229, 1597, 1581, 1489, 1456, 1436, 1292, 1249, 1122, 1012, 904, 785, 731. ***m/z* (EI):** 215.2 (M^+, 78), 200.1 (4), 186.1 (18), 172.1 (21), 159.1 (68), 144.1 (100), 131.1 (22), 116.1 (30), 103.1 (14), 89.0 (19), 77.0 (16), 63.0 (8), 51.0 (8). The data for this compound are consistent with those previously reported in the literature.[5]

Preparation of 2-(2-methoxypyridin-3-yl)-2-propylpentanenitrile (**55**)

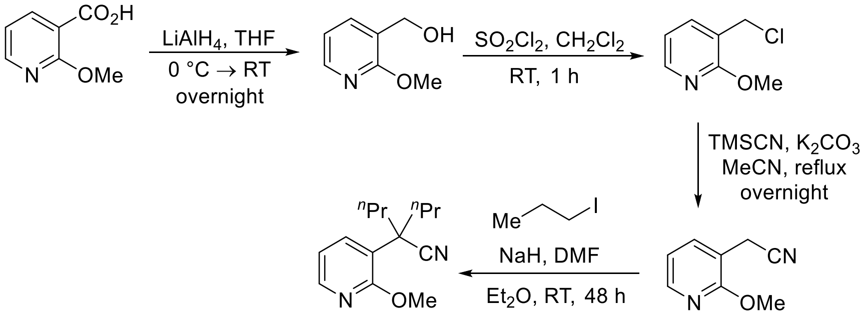

55

This substrate was prepared according to a modified literature procedure.[6] To a solution of $LiAlH_4$ (2 M in THF, 5.5 mL, 11 mmol, 1.1 equiv.) at 0 °C under nitrogen was added a solution of 2-methoxynicotinic acid (1.53 g, 10 mmol, 1 equiv.) in dry THF (21 mL). The resulting mixture was stirred overnight at room temperature and then cooled in an ice bath. The reaction was quenched by the dropwise addition of a THF:water (4:1) solution, until bubbling ceased. 2 M NaOH solution was then added and the reaction mixture was extracted into diethyl ether. The combined organic layers were dried over a hydrophobic frit and concentrated under reduced pressure to afford (2-methoxypyridin-3-yl)methanol as a yellow oil, which required no further purification (1.26 g, 90%). **^{1}H NMR** (400 MHz, $CDCl_3$) 4.00 (s, 3H, OCH_3), 4.66 (s, 2H, CH_2), 6.89 (dd, *J* = 5.1, 7.1 Hz, 1H, ArH), 7.65 - 7.54 (m, 1H, ArH), 8.10 (dd, *J* = 5.4, 2.0 Hz, 1H, ArH). **^{13}C NMR** (101 MHz, $CDCl_3$) 53.3, 61.0, 116.9, 123.3, 136.5, 145.8, 161.6. **ATR-IR ν_{max}** (neat)/cm^{-1} 3326, 2950, 1588, 1462, 1410, 1362, 1307, 1110, 1019, 782. ***m/z* (CI)** 140.1 ($[M+H]^+$). The data for this compound are consistent with those previously reported in the literature.[7]

To a solution of (2-methoxypyridin-3-yl)methanol (1.257 g, 9.03 mmol, 1 equiv.) in dry DCM (43 mL) was added $SOCl_2$ (1.65 mL, 22.58 mmol, 2.5 equiv.) dropwise. The mixture was stirred for 1 h at room temperature. The mixture was then quenched by the addition of a sodium acetate/ice solution and the mixture was stirred for 10 min. The resulting solution was neutralized with $NaHCO_3$ solution and the organic phase was separated and dried over a hydrophobic frit. Removal of the solvent under reduced pressure afforded 3-(chloromethyl)-2-methoxypyridine as a colourless oil (1.35 g, 95%), which was used crude without any further purification. **^{1}H NMR** (400 MHz, $CDCl_3$) 4.02 (s, 3H, OCH_3), 4.61 (s, 2H, CH_2), 6.91 (dd, *J* = 7.3, 5.4 Hz, 1H, ArH), 7.68 (dd, *J* = 7.1, 1.7 Hz, 1H, ArH), 8.10 (dd, *J* = 5.4, 2.0 Hz, 1H, ArH). **^{13}C NMR** (101 MHz, $CDCl_3$) 40.9, 53.6, 116.8, 120.2, 138.5, 147.0, 161.5. **ATR-IR ν_{max}** (neat)/cm^{-1} 2954, 2854, 1585, 1466, 1409, 1307, 1253, 1199, 1017, 856, 776, 582, 497. **HRMS (CI)** calcd. for $C_7H_9NOCl^+$ ($[M+H]^+$): 158.0373, found: 158.0371.

To a mixture of 3-(chloromethyl)-2-methoxypyridine (473 mg, 3 mmol, 1 equiv.) and potassium carbonate (498 mg, 3.6 mmol, 1.2 equiv.) in acetonitrile (30 ml) was added trimethylsilyl cyanide (0.40 ml, 3 mmol, 1 equiv.). The reaction mixture was refluxed overnight, then cooled to room temperature and diluted with 2 M NaOH solution to pH 14. The layers were separated, and the aqueous layer was extracted with DCM. The combined organic phases were washed with 2 M NaOH and brine, dried over a hydrophobic frit, and the solvent was removed under reduced pressure. Purification by column chromatography (cyclohexane:ethyl acetate, 100:0 → 4:1) afforded 2-(2-methoxypyridin-3-yl)aceto nitrile as a colourless oil (212 mg, 48%). **^{1}H NMR** (400 MHz, $CDCl_3$) 3.67 (s, 2 H, CH_2), 4.00 (s, 3 H, OCH_3), 6.93 (dd, *J* = 7.4, 4.9 Hz, 1 H, ArH), 7.68 (d, *J* = 7.2 Hz, 1 H, ArH), 8.15 (dd, *J* = 4.9, 2.0 Hz, 1 H, ArH). **^{13}C NMR** (101 MHz, $CDCl_3$) 18.5, 53.7, 113.3, 116.9, 117.0, 137.4, 146.7, 161.1. **ATR-IR**

ν_{max} (neat)/cm^{-1} 2987, 2953, 2856, 2252, 1589, 1465, 1411, 1313, 1257, 1161, 1105, 1016, 779, 754. **HRMS (CI)** calcd. for $C_8H_9N_2O^+$ ($[M+H]^+$): 149.0715, found: 149.0717.

To a suspension of sodium hydride (108 mg, 4.5 mmol, 3.15 equiv.) in DMF (4 mL) under nitrogen was added a solution of 2-(2-methoxypyridin-3-yl)acetonitrile (212 mg, 1.43 mmol, 1 equiv.) and 1-iodopropane (0.63 mL, 6.44 mmol, 4.5 equiv.) in diethyl ether (2 mL). The resulting mixture was stirred for 48 h at room temperature and then quenched by addition of methanol. The crude residue was redissolved in diethyl ether, washed with water, saturated sodium sulfite solution and saturated sodium carbonate solution, dried over a hydrophobic frit and concentrated under reduced pressure. Purification by column chromatography (hexane:ethyl acetate, 100:0 → 4:1) afforded 2-(2-methoxypyridin-3-yl)-2-propylpentanenitrile **55** as a colourless oil (202 mg, 61%). **^{1}H NMR** (400 MHz, $CDCl_3$) 0.89 (t, *J* = 7.3 Hz, 6 H, 2 x CH_3), 1.00 - 1.14 (m, 2 H, 2 x CH), 1.43 (tdd, *J* = 12.5, 7.4, 4.8 Hz, 2 H, 2 x CH), 1.90 (td, *J* = 12.9, 4.7 Hz, 2 H, 2 x CH), 2.27 (td, *J* = 12.9, 4.7 Hz, 2 H, 2 x CH), 3.98 (s, 3 H, OCH_3), 6.91 (dd, *J* = 7.4, 5.0 Hz, 1 H, ArH), 7.83 (dd, *J* = 7.4, 1.6 Hz, 1 H, ArH), 8.13 (dd, *J* = 5.0, 1.6 Hz, 1 H, ArH). **^{13}C NMR** (101 MHz, $CDCl_3$) 14.0, 19.0, 39.1, 47.9, 53.3, 116.8, 120.4, 122.8, 138.3, 146.0, 160.4. **ATR-IR ν_{max}** (neat)/cm^{-1} 2958, 2931, 2873, 2233, 1581, 1462, 1406, 1099, 1014, 800, 777, 711. ***m/z* (EI):** 232.2 (M^+, 16), 203.1 (6), 189.1 (100), 173.1 (3), 162.1 (30), 147.1 (27), 130.1 (7), 117.0 (6), 104.1 (5), 92.0 (4), 77.0 (4), 65.0 (2), 51.0 (2). **HRMS (CI)** calcd. for $C_{14}H_{21}N_2O^+$ ($[M+H]^+$): 233.1648, found: 233.1650.

Preparation of 2-ethyl-2-(2-methoxyphenyl)butanenitrile (25)

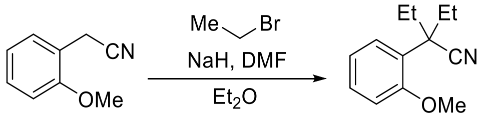

25, 58%

To a stirred solution of sodium hydride (227 mg, 9.45 mmol, 3.15 equiv.) in DMF (11 mL) was added 2-(2-methoxyphenyl)acetonitrile (442 mg, 3.00 mmol, 1.00 equiv.) and bromoethane (1 mL, 13.50 mmol, 4.50 equiv.) in Et_2O (5.5 mL) under argon. The resulting mixture was stirred at room temperature overnight. The reaction mixture was quenched with methanol and concentrated under reduced pressure. The resulting product was re-dissolved in Et_2O and was washed with water, sodium bisulfite and sodium carbonate and was dried by passing through a phase separator and concentrated under reduced pressure. Purification by column chromatography (0-5% Et_2O/hexane) afforded 2-ethyl-2-(2- methoxyphenyl) butanenitrile **25** as a brown oil (355 mg, 58%). **^{1}H NMR** (400 MHz, $CDCl_3$) δ 7.51 (dd, J = 7.7, 1.6 Hz, 1H, ArH), 7.33 – 7.28 (m, 1H, ArH), 6.97 (td, J = 7.6, 1.2 Hz, 1H, ArH), 6.91 (dd, J = 8.2, 1.0 Hz, 1H, ArH), 3.84 (s, 3H, OCH_3), 2.39 – 2.28 (m, 2H, CH_2), 2.06 – 1.95 (m, 2H, CH_2), 0.90 (t, J = 7.4 Hz, 6H, 2 x CH_3). **^{13}C NMR** (101 MHz, $CDCl_3$) δ 157.0, 129.9, 129.0, 124.8, 123.2, 120.6, 111.7, 55.2, 49.8,

30.2, 10.0. **ATR-IR ν_{max}** (neat)/cm^{-1} 2968, 2933, 2884, 2842, 2231, 1568, 1581, 1492. **HRMS (CI)** calcd for $C_{13}H_{18}NO^+$ ([M+H]$^+$) 204.1383, found 204.1383.

Preparation of 2-ethyl-2-(2-ethoxyphenyl)butanenitrile (**31**)

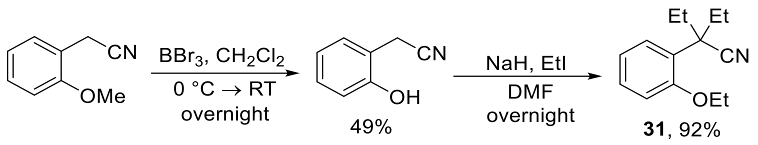

To a solution of 2-methoxyphenylacetonitrile (2.94 g, 20.0 mmol, 1 equiv.) in CH_2Cl_2 (88.0 mL), BBr_3 (5.78 mL, 60.0 mmol, 3 equiv.) was added slowly at 0 °C. The reaction mixture was then warmed to RT and stirred overnight. Upon completion, the reaction mixture was quenched by pouring in crushed ice. The aqueous layer was extracted with EtOAc (x 3) and the combined organic layers were dried over Na_2SO_4, filtered and reduced *in vacuo*. The residue was purified by column chromatography (100% hexane to 3:1 hexane:EtOAc) to afford 2-(2-hydroxyphenyl)acetonitrile (1.31 g, 49%) as a yellow solid. **Mp** = 110-111 °C (lit. mp = 113-114 °C).[8] **^{1}H NMR** (400 MHz, $CDCl_3$) δ 3.73 (s, 2H, CH_2), 4.99 (s, 1H, OH), 6.78 (d, *J* = 8.0 Hz, 1H, ArH), 6.97 (td, *J* = 7.5, 1.2 Hz, 1H, ArH), 7.21 (td, *J* = 7.8, 1.6 Hz, 1H, ArH), 7.31 - 7.40 (m, 1H, ArH). **^{13}C NMR** (101 MHz, $CDCl_3$) δ 18.6 (CH_2), 115.5 (CH), 117.1 (C), 118.1 (C), 121.5 (CH), 129.7 (CH), 129.8 (CH), 153.2 (C). **ATR-IR ν_{max}** (neat)/cm^{-1} 3325, 2259, 1595, 1506, 1460, 1410, 1358, 1273, 1233, 1171, 1099, 1040, 925, 912, 858, 845, 758, 725, 648. ***m/z* (EI)**: 133.1 (M$^+$, 51%), 104.3 (45), 78.1 (100), 64.0 (22), 55.1 (68).

An oven-dried 3-neck flask was taken into the glovebox, where NaH (649 mg, 27.0 mmol, 9 equiv.) was added. The flask was sealed under argon and taken outside the glovebox. DMF (12.0 mL) was then added followed by 2-(2-hydroxyphenyl)acetonitrile (400 mg, 3.00 mmol, 1 equiv.). EtI (2.17 mL, 27.0 mmol, 9 equiv.) was added slowly and the reaction mixture was stirred overnight at RT. The reaction was then quenched with water and the aqueous layer was extracted with EtOAc (x 3). The combined organic layers were dried over Na_2SO_4, filtered and reduced *in vacuo*. 2-(2-Ethoxyphenyl)-2-ethylbutanenitrile **31** (603 mg, 92%) was obtained as a yellow oil. **^{1}H NMR** (400 MHz, $CDCl_3$) δ 0.92 (t, *J* = 7.4 Hz, 6H, CH_3), 1.47 (t, *J* = 7.0 Hz, 3H, CH_3), 1.95 - 2.08 (m, 2H, CH_2), 2.33 - 2.46 (m, 2H, CH_2), 4.09 (q, *J* = 7.0 Hz, 2H, CH_2), 6.90 (dd, *J* = 8.2, 1.3 Hz, 1H, ArH), 6.96 (td, *J* = 7.6, 1.2 Hz, 1H, ArH), 7.24 - 7.33 (m, 1H, ArH), 7.52 (dd, *J* = 7.7, 1.6 Hz, 1H, ArH). **^{13}C NMR** (101 MHz, $CDCl_3$) δ 10.21 (CH_3), 14.89 (CH_3), 30.46 (CH_2), 50.06 (C), 63.85 (CH_2), 112.34 (CH), 120.57 (CH), 123.45 (C), 124.72 (C), 129.13 (CH), 130.13 (CH), 156.38 (C). **ATR-IR ν_{max}** (neat)/cm^{-1} 2232, 1599, 1584, 1493, 1445, 1393, 1294, 1244, 1090, 1040, 924, 889, 750. **HRMS (CI)** calculated for $C_{14}H_{20}NO$ ([M+H]$^+$): 218.1545, found: 218.1545.

Preparation of 3-(2-methoxyphenyl)-2,2-dimethylpropanenitrile (**27**)

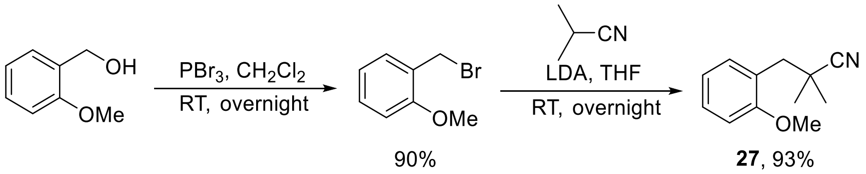

To a stirring solution of (2-methoxyphenyl)methanol (1 mL, 7.20 mmol, 1.00 equiv.) in DCM (14 mL) at 0 °C was added phosphorus tribromide (1 mL, 10.90 mmol, 1.50 equiv.) and the solution was allowed to warm to room temperature and stirred overnight. The reaction mixture was quenched with water, extracted into Et_2O and dried by passing through a phase separator. The solvent was removed under reduced pressure to afford 1-(bromomethyl)-2-methoxybenzene as a brown oil (1.30 g, 90%), without further purification required. **^{1}H NMR** (400 MHz, $CDCl_3$) δ 7.34 (dd, J = 7.5, 1.7 Hz, 1H, ArH), 7.31 (ddd, J = 8.1, 7.6, 1.7 Hz, 1H, ArH), 6.94 (td, J = 7.5, 1.0 Hz, 1H, ArH), 6.90 (d, J = 8.1 Hz, 1H, ArH), 4.59 (s, 2H, CH_2), 3.91 (s, 3H, OCH_3). **^{13}C NMR** (101 MHz, $CDCl_3$) δ 157.5, 130.9, 130.2, 126.1, 120.7, 111.0, 55.6, 29.0. **ATR-IR ν_{max}** (neat)/cm^{-1} 2993, 2935, 2831, 1599, 1490, 1460, 1288, 1026. ***m/z* (EI):** 200.0 (M^+, 75%), 121.2 (100), 106.1 (40), 91.2 (99).

To a stirring solution of isobutyronitrile 171 (0.11 mL, 1.25 mmol, 0.50 equiv.) in THF (3 mL) at −78 °C was added LDA in THF/heptane/ethylbenzene (0.34 M) (4.00 mL, 1.36 mmol, 0.55 equiv.) and the solution was stirred for 20 min. 1-(Bromomethyl)-2-methoxybenzene (500 mg, 2.50 mmol, 1.00 equiv.) was added to the mixture and the solution stirred for 30 min at −78 °C. The stirring solution was allowed to warm to room temperature and stirred overnight. The resulting solution was quenched with saturated NH4Cl and extracted into Et_2O, with the organic layers passed through a phase separator and solvent removed under reduced pressure. Purification by column chromatography (0-5% Et_2O/hexane), afforded 3-(2-methoxyphenyl)-2,2- dimethylpropanenitrile **27** as a colourless oil (220 mg, 93%). **^{1}H NMR** (400 MHz, $CDCl_3$) δ 7.30 – 7.25 (m, 2H, 2 x ArH), 6.95 (td, J = 7.5, 1.1 Hz, 1H, ArH), 6.90 (d, J = 8.6 Hz, 1H, ArH), 3.82 (s, 3H, OCH_3), 2.91 (s, 2H, CH_2), 1.36 (s, 6H, 2 x CH_3). **^{13}C NMR** (101 MHz, $CDCl_3$) δ 157.9, 131.9, 128.6, 125.2, 124.4, 120.4, 110.5, 55.1, 39.3, 33.8, 26.5. **ATR-IR ν_{max}** (neat)/cm^{-1} 2970, 2933, 2833, 2231, 1599, 1585, 1491, 1462. **HRMS (CI)** calcd for $C_{12}H_{19}N_2O^+$ ($[M+NH_4]^+$) 207.1492, found 207.1490.

Preparation of 3,3-dimethyl-2-phenyl-3*H*-indole (**72**)

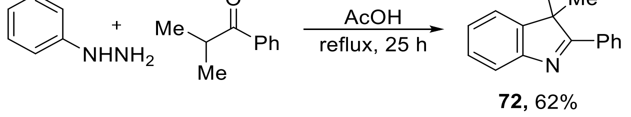

A solution of isobutyrylbenzene (1.0 eq., 20 mmol, 3.000 mL) and phenylhydrazine (1.0 eq., 20 mmol, 1.970 mL) in AcOH (30 mL) was stirred at reflux for 25 h under argon. Reaction was cooled to room temperature and concentrated under reduced pressure. The residue was redissolved in CH_2Cl_2 and

washed with sat. $NaHCO_3$. The aqueous layer was separated from the organic layer and was further washed with CH_2Cl_2. The combined organic phases were dried over Na_2SO_4, filtered and concentrated. Purification by column chromatography (hexane → 2% EtOAc/98% hexane) afforded 3,3-dimethyl-2-phenyl-3*H*-indole **72** (2.723 g, 62%) as a pale yellow solid. **Mp** = 37-39 °C (lit. mp = 39-41 °C).[9] **^{1}H NMR** (400 MHz, $CDCl_3$) δ 8.24 – 8.05 (m, 2H, 2 x ArH), 7.72 (d, *J* = 7.6 Hz, 1H, ArH), 7.53 – 7.45 (m, 3H, 3 x ArH), 7.42 – 7.33 (m, 2H, 2 x ArH), 7.33 – 7.22 (m, 1H, ArH), 1.61 (s, 6H, 2 x Me). **^{13}C NMR** (101 MHz, $CDCl_3$) δ 183.4, 153.1, 147.7, 133.4, 130.7, 128.7, 128.5, 127.9, 126.0, 121.1, 121.0, 53.7, 24.9. **ATR-IR ν_{max}** (neat)/cm^{-1} 3086, 3057, 3042, 3024, 2965, 2930, 2866, 1518, 1491, 1453, 1443, 1385, 1364, 1337, 1263, 1221, 1207, 1074, 1030, 1013, 1003, 988, 934, 922, 860, 746, 710, 694, 635. ***m/z* (EI):** 221.1 (M^+, 100%), 206.1 (53), 178.0 (6), 165.0 (11), 144.0 (16), 128.1 (10), 115.0 (40), 103.0 (74), 91.0 (28), 77.0 (88), 63.0 (18), 51.0 (41). The data are consistent with those previously reported in the literature[1]

Preparation of 2'-phenylspiro[cyclohexane-1,3'-indole] (**76**)

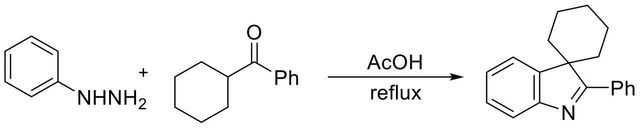

76, 57%

Prepared according to **General Procedure D** using cyclohexyl(phenyl)methanone (1.0 eq., 10 mmol, 1.883 g), phenylhydrazine (1.0 eq., 10 mmol, 985 µL) and AcOH (10 mL). Purification by column chromatography (hexane → 2% EtOAc/98% hexane) afforded 2'-phenylspiro[cyclohexane-1,3'-indole] **76** (1.496 g, 57%) as a viscous yellow oil. **^{1}H NMR** (400 MHz, $CDCl_3$) δ 8.06 (dd, *J* = 6.7, 2.9 Hz, 2H, 2 x ArH), 7.83 (d, *J* = 7.5 Hz, 1H, ArH), 7.74 (d, *J* = 7.7 Hz, 1H, ArH), 7.54 – 7.45 (m, 3H, 3 x ArH), 7.41 (td, *J* = 7.6, 1.1 Hz, 1H, ArH), 7.32 – 7.18 (m, 1H, ArH), 2.30 (td, *J* = 13.4, 4.5 Hz, 2H, Cy), 2.12 – 1.93 (m, 3H, Cy), 1.93 – 1.77 (m, 2H, Cy), 1.63 – 1.49 (m, 1H, Cy), 1.45 (d, *J* = 13.7 Hz, 2H, Cy). **^{13}C NMR** (101 MHz, $CDCl_3$) δ 184.1, 153.6, 146.2, 133.9, 130.2, 128.8, 128.5, 127.8, 125.1, 124.5, 121.4, 58.6, 31.3, 25.3, 21.8. **ATR-IR ν_{max}** 3057, 2924, 2862, 1520, 1491, 1466, 1452, 1439, 1348, 1335, 1310, 1263, 1221, 1180, 1155, 1115, 1074, 1061, 1030, 1020, 1001, 988, 924, 905, 885, 851, 773, 748, 692, 673, 635, 625, 613. ***m/z* (EI):** 261.1 (M^+, 100%), 246.1 (2), 232.1 (49), 217.1 (17), 204.1 (30), 184.1 (23), 170.1 (8), 156.1 (11), 143.1 (4), 131.1 (26), 115.1 (21), 103.0 (10), 91.0 (10), 77.0 (12), 63.0 (3), 51.0 (5). The data are consistent with those previously reported in the literature[1]

Preparation of 2-(4-methoxyphenyl)-3,3-dimethyl-3*H*-indole

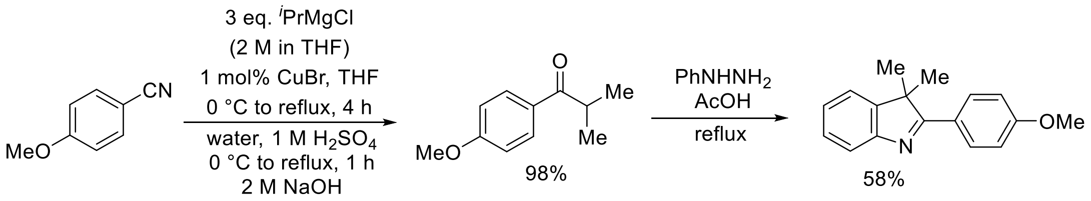

1-(4-Methoxyphenyl)-2-methylpropan-1-one was prepared according to **General Procedure E** using ^{i}PrMgCl (3.0 eq., 30 mmol, 15 mL) as a 2 M solution in THF, 4-methoxybenzonitrile (1.0 eq., 10 mmol, 1.322 g), dry THF (10 mL) and CuBr (1 mol%, 0.1 mmol, 14.35 mg). The 1-(4-methoxyphenyl)-2-methylpropan-1-one (1.752 g, 98%) product was isolated as a yellow liquid. **^{1}H NMR** (400 MHz, $CDCl_3$) δ 7.95 (d, *J* = 8.9 Hz, 2H, 2 x ArH), 6.94 (d, *J* = 8.9 Hz, 2H, 2 x ArH), 3.87 (s, 3H, MeO), 3.51 (hept, *J* = 6.8 Hz, 1H, CH), 1.20 (d, *J* = 6.8 Hz, 6H, 2 x Me). **^{13}C NMR** (101 MHz, $CDCl_3$) δ 203.2, 163.4, 130.7, 129.3, 113.9, 55.6, 35.1, 19.4. **ATR-IR ν_{max}** 3003, 2968, 2932, 2872, 2839, 1672, 1597, 1574, 1508, 1462, 1418, 1381, 1350, 1306, 1258, 1225, 1179, 1157, 1113, 1084, 1030, 1009, 978, 870, 841, 800, 760, 687, 633, 602. ***m/z* (EI):** 178.1 (M^+, 5%), 135.0 (100), 107.0 (8), 92.0 (20), 77.0 (22), 64.0 (11), 51.0 (2). The data are consistent with those previously reported in the literature[2]

2-(4-Methoxyphenyl)-3,3-dimethyl-3*H*-indole was prepared according to **General Procedure D** using 1-(4-methoxyphenyl)-2-methylpropan-1-one (1.0 eq., 7 mmol, 1.248 g), phenylhydrazine (1.0 eq., 7 mmol, 689.4 µL) and AcOH (10 mL). Purification by column chromatography (hexane → 7% EtOAc/93% hexane) afforded 2-(4-methoxyphenyl)-3,3-dimethyl-3*H*-indole (1.021 g, 58%) as a white solid. **Mp** = 97-100 °C (no mp reported in the lit.). **^{1}H NMR** (400 MHz, $CDCl_3$) δ 8.15 (d, *J* = 9.0 Hz, 2H, 2 x ArH), 7.67 (d, *J* = 7.6 Hz, 1H, ArH), 7.42 – 7.29 (m, 2H, 2 x ArH), 7.24 (td, *J* = 7.4, 1.2 Hz, 1H, ArH), 7.00 (d, *J* = 9.0 Hz, 2H, 2 x ArH), 3.88 (s, 3H, MeO), 1.59 (s, 6H, 2 x Me). **^{13}C NMR** (101 MHz, $CDCl_3$) δ 182.7, 161.6, 153.3, 147.6, 130.1, 127.7, 126.0, 125.4, 120.9, 120.5, 114.0, 55.4, 53.3, 25.0. **ATR-IR ν_{max}** 3042, 2999, 2970, 2957, 2928, 2833, 1603, 1578, 1506, 1468, 1454, 1420, 1385, 1366, 1337, 1314, 1269, 1246, 1213, 1184, 1167, 1109, 1088, 1030, 1013, 988, 972, 937, 866, 839, 818, 804, 772, 754, 694, 637. ***m/z* (EI):** 251.1 (M^+, 100%), 236.1 (44), 221.1 (4), 204.1 (5), 193.1 (8), 180.1 (3), 167.1 (5), 144.1 (10), 133.1 (7), 117.0 (28), 103.0 (32), 91.0 (20), 77.0 (31), 63.0 (10), 51.0 (9). The data are consistent with those previously reported in the literature[1]

Preparation of 2-(4-fluorophenyl)-3,3-dimethyl-3*H*-indole

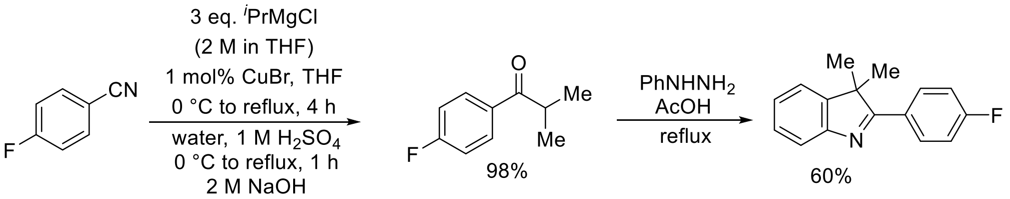

1-(4-Fluorophenyl)-2-methylpropan-1-one was prepared according to **General Procedure E** using ^{i}PrMgCl (3.0 eq., 30 mmol, 15 mL) as a 2 M solution in THF, 4-fluorobenzonitrile (1.0 eq., 10 mmol, 1.322 g), dry THF (10 mL) and CuBr (1 mol%, 0.1 mmol, 14.35 mg). The 1-(4-fluorophenyl)-2-methylpropan-1-one (1.630 g, 98%) product was isolated as an orange liquid. **^{1}H NMR** (400 MHz, $CDCl_3$) δ 8.05 – 7.92 (m, 2H, 2 x ArH), 7.20 – 7.07 (m, 2H, 2 x ArH), 3.51 (hept, *J* = 6.8 Hz, 1H, CH), 1.21 (d, *J* = 6.8 Hz, 6H, 2 x Me). **^{13}C NMR** (101 MHz, $CDCl_3$) δ 203.0, 165.7 (d, *J* = 254.3 Hz), 132.7 (d, *J* = 2.6 Hz), 131.1 (d, *J* = 9.2 Hz), 115.8 (d, *J* = 21.7 Hz), 35.5, 19.3. **ATR-IR ν_{max}** 3071, 2972,

2934, 2874, 1682, 1595, 1504, 1468, 1410, 1383, 1350, 1296, 1283, 1219, 1152, 1103, 1013, 980, 870, 845, 816, 756, 681, 631. ***m/z* (EI):** 166.1 (M^+, 6%), 123.0 (100), 95.0 (50), 75.0 (27), 69.0 (4), 51.0 (2). The data are consistent with those previously reported in the literature[2]

2-(4-Fluorophenyl)-3,3-dimethyl-3*H*-indole was prepared according to **General Procedure D** using 1-(4-fluorophenyl)-2-methylpropan-1-one (1.0 eq., 7 mmol, 1.163 g), phenylhydrazine (1.0 eq., 7 mmol, 689.4 µL) and AcOH (10 mL). Purification by column chromatography (hexane → 2% EtOAc/98% hexane) afforded 2-(4-fluorophenyl)-3,3-dimethyl-3*H*-indole (1.000 g, 60%) as a yellow solid. **Mp** = 61-64 °C (no mp reported in the lit.). **^{1}H NMR** (400 MHz, $CDCl_3$) δ 8.17 (dd, *J* = 8.6, 5.6 Hz, 2H, 2 x ArH), 7.69 (d, *J* = 7.6 Hz, 1H, ArH), 7.46 – 7.33 (m, 2H, 2 x ArH), 7.33 – 7.23 (m, 1H, ArH), 7.19 (t, *J* = 8.6 Hz, 2H, 2 x ArH), 1.59 (s, 6H, 2 x Me). **^{13}C NMR** (101 MHz, $CDCl_3$) δ 182.2, 164.3 (d, *J* = 252.2 Hz), 153.1, 147.6, 130.6 (d, *J* = 8.7 Hz), 129.71 (d, *J* = 2.3 Hz), 128.0, 126.0, 121.0 (d, *J* = 10.0 Hz), 116.0, 115.7, 53.6, 24.9. **ATR-IR ν_{max}** 3071, 2965, 2926, 2864, 1599, 1522, 1504, 1470, 1454, 1406, 1385, 1362, 1335, 1310, 1294, 1267, 1213, 1161, 1107, 1084, 1013, 989, 976, 935, 870, 841, 816, 770, 754, 735, 696, 637, 627. ***m/z* (EI):** 239.1 (M^+, 100%), 224.1 (57), 196.1 (4), 183.0 (12), 144.0 (16), 128.0 (11), 117.0 (43), 103.0 (34), 91.0 (25), 77.0 (49), 63.0 (13), 51.0 (20). The data are consistent with those previously reported in the literature[5]

Preparation of 3,3-dimethyl-2-(4-(trifluoromethyl)phenyl)-3*H*-indole

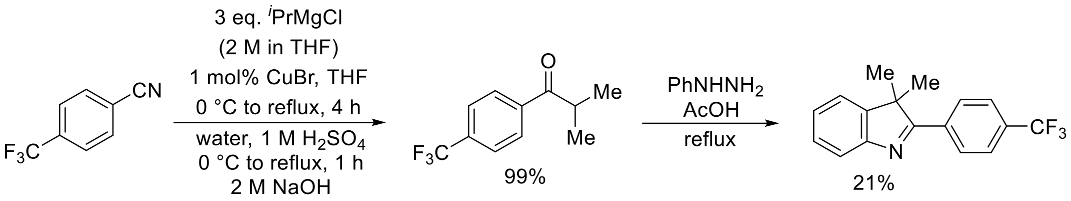

2-Methyl-1-(4-(trifluoromethyl)phenyl)propan-1-one was prepared according to **General Procedure E** using ^{i}PrMgCl (3.0 eq., 30 mmol, 15 mL) as a 2 M solution in THF, 4-(trifluoromethyl)benzonitrile (1.0 eq., 10 mmol, 1.712 g), dry THF (10 mL) and CuBr (1 mol%, 0.1 mmol, 14.35 mg). The 2-methyl-1-(4-(trifluoromethyl)phenyl)propan-1-one (2.131 g, 99%) product was isolated as a brown liquid. **^{1}H NMR** (400 MHz, $CDCl_3$) δ 8.05 (d, *J* = 8.1 Hz, 2H, 2 x ArH), 7.73 (d, *J* = 8.1 Hz, 2H, 2 x ArH), 3.54 (hept, *J* = 6.8 Hz, 1H, CH), 1.23 (d, *J* = 6.8 Hz, 6H, 2 x Me). **^{13}C NMR** (101 MHz, $CDCl_3$) δ 203.6, 139.1, 134.3 (q, *J* = 32.7 Hz), 128.8, 125.8 (q, *J* = 3.5 Hz), 123.8 (q, *J* = 272.9 Hz), 36.0, 19.0. **ATR-IR ν_{max}** 2974, 2936, 2876, 1690, 1582, 1510, 1468, 1408, 1385, 1321, 1219, 1163, 1126, 1113, 1067, 1016, 982, 853, 766, 723, 696. ***m/z* (EI):** 216.0 (M^+, 4%), 197.1 (3), 173.0 (100), 145.0 (46), 125.0 (7), 95.0 (7), 75.0 (5), 51.0 (1). The data are consistent with those previously reported in the literature[2]

3,3-Dimethyl-2-(4-(trifluoromethyl) phenyl)-3*H*-indole was prepared according to **General Procedure D** using 2-methyl-1-[4-(trifluoromethyl)phenyl]propan-1-one (1.0 eq., 7 mmol, 1.513 g), phenylhydrazine (1.0 eq., 7 mmol, 689.4 µL) and AcOH (10 mL). Purification by column

chromatography (hexane → 2% EtOAc/98% hexane) afforded 3,3-dimethyl-2-(4-(trifluoromethyl) phenyl)-3*H*-indole (424 mg, 21%) as a yellow solid. **Mp** = 96-98 °C (no mp reported in the lit.). **^{1}H NMR** (400 MHz, $CDCl_3$) δ 8.25 (d, *J* = 8.2 Hz, 2H, 2 x ArH), 7.80 – 7.67 (m, 3H, 3 x ArH), 7.44 – 7.28 (m, 3H, 3 x ArH), 1.60 (s, 6H, 2 x Me). **^{13}C NMR** (101 MHz, $CDCl_3$) δ 181.9, 153.0, 147.7, 136.7, 132.1 (q, *J* = 32.6 Hz), 128.7, 128.1, 126.7, 125.7 (q, 3.2 Hz), 124.1 (q, *J* = 271.1 Hz), 121.5, 121.2, 53.8, 24.6. **ATR-IR ν_{max}** 3073, 3026, 2976, 2967, 2930, 2868, 1618, 1518, 1472, 1454, 1408, 1321, 1308, 1165, 1125, 1111, 1090, 1067, 1013, 991, 934, 864, 845, 789, 772, 748, 698, 683, 600. ***m/z* (EI):** 289.1 (M^+, 100%), 274.1 (56), 254.1 (1), 233.1 (5), 218.1 (2), 204.1 (10), 179.1 (8), 144.1 (17), 130.5 (9), 117.1 (31), 103.1 (25), 91.0 (14), 77.0 (24), 63.0 (4). 51.0 (8). The data are consistent with those previously reported in the literature[1]

Preparation of 3,3-dimethyl-2-(*p*-tolyl)-3*H*-indole

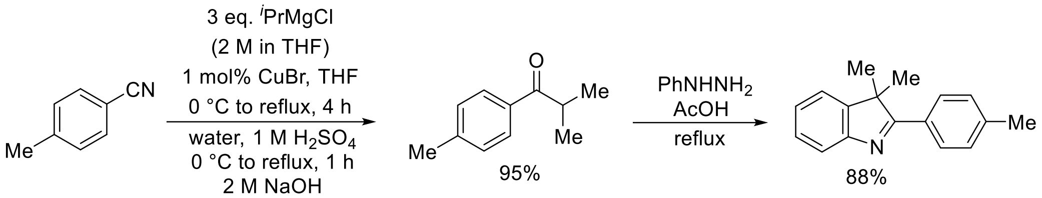

2-Methyl-1-(*p*-tolyl)propan-1-one was prepared according to **General Procedure E** using ^{i}PrMgCl (3.0 eq., 30 mmol, 15 mL) as a 2 M solution in THF, 4-methylbenzonitrile (1.0 eq., 10 mmol, 1.172 g), dry THF (10 mL) and CuBr (1 mol%, 0.1 mmol, 14.35 mg). The 2-methyl-1-(*p*-tolyl)propan-1-one (1.537 g, 95%) product was isolated as a yellow liquid. **^{1}H NMR** (400 MHz, $CDCl_3$) δ 7.86 (d, *J* = 8.0 Hz, 2H, 2 x ArH), 7.26 (d, *J* = 8.0 Hz, 2H, 2 x ArH), 3.53 (hept, *J* = 6.8 Hz, 1H, CH), 2.41 (s, 3H, Me), 1.21 (d, *J* = 6.8 Hz, 6H, 2 x Me). **^{13}C NMR** (101 MHz, $CDCl_3$) δ 204.3, 143.6, 133.8, 129.4, 128.6, 35.3, 21.7, 19.3. **ATR-IR ν_{max}** 3030, 2970, 2932, 2872, 1678, 1607, 1572, 1466, 1408, 1381, 1350, 1288, 1229, 1209, 1186, 1161, 1119, 1084, 1038, 976, 870, 827, 745, 685, 637. ***m/z* (EI):** 162.1 (M^+, 6%), 119.1 (100), 91.1 (38), 65.0 (16), 51.0 (2). The data are consistent with those previously reported in the literature[2]

3,3-Dimethyl-2-(*p*-tolyl)-3*H*-indole was prepared according to **General Procedure D** using 2-methyl-1-(*p*-tolyl)propan-1-one (1.0 eq., 7 mmol, 1.135 g), phenylhydrazine (1.0 eq., 7 mmol, 689.4 µL) and AcOH (10 mL). An extra portion of phenylhydrazine (1.0 eq., 7 mmol, 689.4 µL) was added after 16 h in order to push the reaction to completion and the reaction was further refluxed for 16 h. Purification by column chromatography (hexane → 2% EtOAc/98% hexane) afforded 3,3-dimethyl-2-(*p*-tolyl)-3*H*-indole (1.445 mg, 88%) as a yellow oil. **^{1}H NMR** (400 MHz, $CDCl_3$) δ 8.07 (d, *J* = 8.3 Hz, 2H, 2 x ArH), 7.69 (d, *J* = 7.6 Hz, 1H, ArH), 7.43 – 7.20 (m, 5H, 5 x ArH), 2.43 (s, 3H, Me), 1.60 (s, 6H, 2 x Me). **^{13}C NMR** (101 MHz, $CDCl_3$) δ 183.3, 153.3, 147.7, 141.0, 130.6, 129.5, 128.5, 127.8, 125.7, 121.0, 120.8, 53.5, 25.0, 21.6. **ATR-IR ν_{max}** 3061, 3024, 2963, 2926, 2864, 1607, 1508, 1468, 1454, 1408, 1385, 1364, 1337, 1314, 1265, 1211, 1184, 1169, 1117, 1107, 1088, 1036, 1013, 993, 935, 862,

822, 772, 750, 727, 694, 640, 629. ***m/z* (EI):** 235.2 (M^+, 100%), 220.1 (61), 204.1 (8), 191.1 (1), 179.1 (7), 165.1 (1), 153.1 (1), 144.1 (10), 128.1 (5), 117.1 (27), 103.2 (21), 91.1 (16), 77.1 (20), 65.1 (7), 51.1 (7). The data are consistent with those previously reported in the literature[10]

Preparation of 3,3-diallyl-2-phenyl-3*H*-indole (**82**)

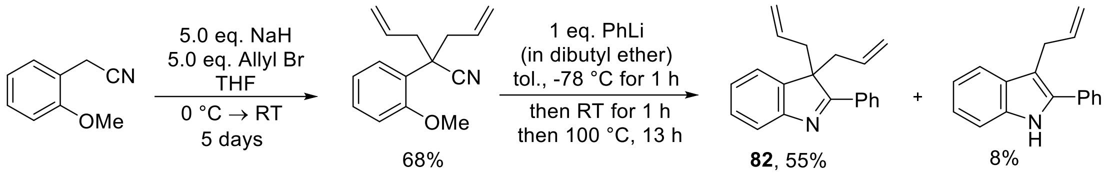

This substrate was prepared according to a modified literature procedure.[5] 2-Methoxyphenylacetonitrile (1.0 eq., 10.0 mmol, 1.472 g) was added portion wise to a slurry of NaH (5.0 eq., 50.0 mmol, 1.200 g) in dry THF (100 mL) at 0 °C under argon. Reaction mixture was warmed up to room temperature and was stirred for 1 h before allyl bromide (5.0 eq., 50.0 mmol, 4.327 mL) was added. Reaction was stirred a room temperature over ~ 5 days and the reaction progress was monitored by TLC. Once all 2-methoxyphenylacetonitrile was consumed, the reaction mixture was quenched with NH_4Cl (100 mL) at 0 °C. The reaction mixture was then extracted with EtOAc (3 x 50 mL). The combined organic phases were dried over $MgSO_4$, filtered and concentrated. Purification by column chromatography (hexane → 10% EtOAc/90% hexane) afforded 2-allyl-2-(2-methoxyphenyl)pent-4-enenitrile (1.544 g, 68%) as a yellow oil. **^{1}H NMR** (400 MHz, $CDCl_3$) δ 7.44 (dd, *J* = 7.7, 1.6 Hz, 1H, ArH), 7.30 (ddd, *J* = 8.2, 7.5, 1.6 Hz, 1H, ArH), 6.99 – 6.89 (m, 2H, 2 x ArH), 6.00 – 5.39 (m, 2H, 2 x alkene CH), 5.17 – 5.03 (m, 4H, 2 x alkene CH_2), 3.88 (s, 3H, Me), 3.03 (ddt, *J* = 13.9, 6.9, 1.2 Hz, 2H, 2 x CH), 2.75 (ddt, *J* = 13.9, 7.6, 1.0 Hz, 2H, 2 x CH). **^{13}C NMR** (101 MHz, $CDCl_3$) δ 157.0 132.8, 129.5, 129.5, 124.7, 122.4, 120.9, 119.4, 111.9, 55.4, 47.4, 41.0. **ATR-IR ν_{max}** (neat)/cm^{-1} 3078, 2980, 2918, 2837, 2234, 1641, 1584, 1493, 1464, 1437, 1288, 1246, 1105, 1024, 991, 920, 787, 752, 606. ***m/z* (EI):** 227.1 (M^+, 17%), 186.1 (100), 171.1 (25), 155.1 (20), 144.1 (40), 127.1 (21), 115.1 (42), 102.1 (8), 89.0 (18), 77.1 (16), 63.0 (11), 51.0 (10). The data are consistent with those previously reported in the literature[5]

2-Allyl-2-(2-methoxyphenyl)pent-4-enenitrile (1.0 eq., 1.5 mmol, 341 mg) and dry toluene (15 mL) were sealed in a microwave vial in a nitrogen-filled glovebox. PhLi as a 1.56 M solution in dibutyl ether (1.05 eq., 1.56 mmol, 1.00 mL) was added dropwise at – 78 °C and the reaction mixture was stirred at – 78 °C for 1 h before it was warmed up to room temperature and stirred for an additional 1 h. The reaction mixture was then heated to 100 °C and stirred at that temperature for 13 h. The contents of the microwave vial were cooled to room temperature and were quenched with sat. NH_4Cl (50 mL). The organic products were extracted into Et_2O (3 x 50 mL). The combined organic layers were dried over Na_2SO_4, filtered and concentrated. Purification by column chromatography (hexane → 2% EtOAc/98% hexane) afforded 3,3-diallyl-2-phenyl-3*H*-indole **82** (228 mg, 55%) as a yellow solid and 3-allyl-2-phenyl-1*H*-indole (27 mg, 8%) as a yellow solid.

3,3-Diallyl-2-phenyl-3*H*-indole: **Mp** = 51-52 °C (no lit. mp). **^{1}H NMR** (400 MHz, $CDCl_3$) δ 8.12 (m, 2H, 2 x ArH), 7.70 – 7.65 (d, *J* = 7.7 Hz, 1H, ArH), 7.50 – 7.46 (m, 3H, 3 x ArH), 7.38 – 7.32 (m, 2H, 2 x ArH), 7.30 – 7.25 (m, 1H, ArH), 5.12 (ddt, *J* = 17.2, 10.1, 7.2 Hz, 2H, 2 x alkene CH), 4.82 – 4.69 (m, 4H, 2 x alkene CH_2), 2.94 – 2.87 (m, 4H). **^{13}C NMR** (101 MHz, $CDCl_3$) δ 180.4, 154.7, 143.1, 134.2, 132.0, 130.7, 128.7, 128.2, 128.2, 125.8, 121.9, 120.9, 118.3, 62.5, 41.9. **ATR-IR ν_{max}** (neat)/cm^{-1} 3071, 3003, 2951, 2887, 1638, 1522, 1493, 1454, 1441, 1416, 1344, 1261, 1217, 1113, 1072, 995, 961, 918, 868, 785, 756, 723, 692, 675, 635, 619, 610. ***m/z* (EI)**: 271.1 (M^+, 100%), 246.1 (29), 232.1 (78), 217.0 (41), 204.1 (25), 154.0 (14), 128.1 (51), 115.0 (14), 103.1 (22), 77.0 (27), 51.0 (14). The data for this compound were consistent with those previously reported in the literature.[5]

3-Allyl-2-phenyl-1*H*-indole: **Mp =** 94-96 °C (lit. mp = 98-99 °C).[11] **^{1}H NMR** (400 MHz, $CDCl_3$) δ 8.06 (br s, 1H, NH), 7.65 – 7.61 (m, 1H, ArH), 7.60 – 7.56 (m, 2H, 2 x ArH), 7.52 – 7.45 (m, 3H, 3 x ArH), 7.41 – 7.36 (m, 2H, 2 x ArH), 7.23 (ddd, *J* = 8.3, 7.2, 1.2 Hz, 1H, ArH), 7.15 (ddd, *J* = 8.0, 7.1, 1.0 Hz, 1H, ArH), 6.15 (ddt, *J* = 16.1, 10.4, 5.6 Hz, 1H, alkene CH), 5.16 – 5.08 (m, 2H, alkene CH_2), 3.65 (2 x t, *J* = 1.8 Hz, 2H, 2 x CH). **^{13}C NMR** (101 MHz, $CDCl_3$) δ 137.5, 136.1, 135.0, 133.1, 129.5, 129.0, 128.0, 127.8, 122.5, 119.8, 119.6, 115.3, 110.9, 110.7, 29.1. **ATR-IR ν_{max}** (neat)/cm^{-1} 3395, 3075, 3003, 2922, 2893, 1636, 1603, 1485, 1450, 1423, 1341, 1304, 1240, 1204, 1153, 1074, 1028, 997, 920, 764, 748, 694, 669, 629. ***m/z* (EI)**: 233.1 (M^+, 99%), 217.1 (29), 206.1 (100), 190.0 (4), 178.1 (16), 165.0 (5), 154.1 (13), 138.9 (4), 128.0 (25), 115.1 (8), 108.7 (26), 102.1 (18), 89.0 (8), 77.0 (32), 63.0 (12), 51.0 (21). The data for this compound were consistent with those previously reported in the literature.[11]

Preparation of 3,3-dibenzyl-2-phenyl-3*H*-indole (**83**)

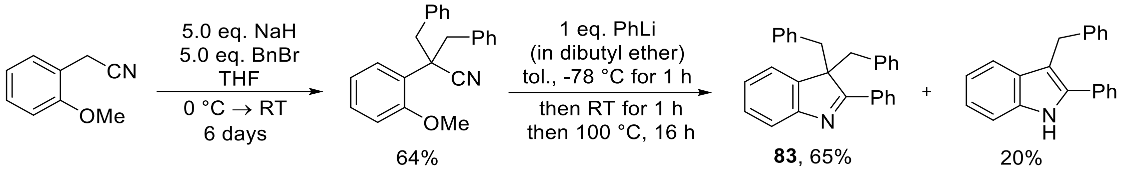

This substrate was prepared according to a modified literature procedure.[5] 2-Methoxyphenylacetonitrile (1.0 eq., 10.0 mmol, 1.472 g) was added portion wise to a slurry of NaH (5.0 eq., 50.0 mmol, 1.200 g) in dry THF (100 mL) at 0 °C under argon. Reaction mixture was warmed up to room temperature and was stirred for 1 h before benzyl bromide (5.0 eq., 50.0 mmol, 5.947 mL) was added. Reaction was stirred a room temperature over ~ 6 days and the reaction progress was monitored by TLC. Once all 2-methoxyphenylacetonitrile was consumed, the reaction mixture was quenched with NH_4Cl (100 mL) at 0 °C. The reaction mixture was then extracted with EtOAc (3 x 50 mL). The combined organic phases were dried over $MgSO_4$, filtered and concentrated. Purification by recrystallisation from hexane/EtOAc afforded 2-benzyl-2-(2-methoxyphenyl)-3-phenylpropanenitrile (2.089 g, 64%) as a white solid. **Mp** = 133-134 °C (lit. mp = 139 °C).[12] **^{1}H NMR** (400 MHz, $CDCl_3$) δ 7.24 (ddd, *J* = 8.2, 7.5, 1.7 Hz, 1H, ArH), 7.19 – 7.14 (m, 6H, 6 x ArH), 7.11 – 7.02 (m, 5H, 5 x ArH), 6.96 (dd, *J* = 8.2, 0.9 Hz, 1H, ArH),

6.69 (td, J = 7.6, 1.2 Hz, 1H, ArH), 4.06 (s, 3H, Me), 3.83 (d, J = 13.4 Hz, 2H, 2 x CH), 3.31 (d, J = 13.5 Hz, 2H, 2 x CH). **^{13}C NMR** (101 MHz, $CDCl_3$) δ 156.8, 136.3, 130.8, 130.3, 129.5, 128.1, 127.1, 123.8, 122.2, 121.0, 111.4, 55.3, 52.5, 42.9. **ATR-IR ν_{max}** (neat)/cm^{-1} 3065, 3024, 3001, 2926, 2841, 2232, 1601, 1582, 1493, 1470, 1452, 1439, 1406, 1339, 1294, 1269, 1246, 1186, 1167, 1153, 1121, 1080, 1065, 1043, 1022, 997, 970, 934, 916, 905, 851, 841, 799, 785, 770, 750, 739, 698, 679, 658, 608. ***m/z* (EI):** 327.2 (M^+, 47%), 236.1 (86), 220.1 (11), 209.1 (8), 194.1 (32), 178.1 (6), 165.1 (20), 152.1 (4), 140.1 (1), 131.1 (3), 115.1 (4), 103.1 (3), 91.1 (100), 77.0 (5), 65.1 (18), 51.0 (3). **HRMS (ESI)** calcd. for $C_{23}H_{22}NO^+$ ($[M+H]^+$): 328.1696, found 328.1694.

2-Benzyl-2-(2-methoxyphenyl)-3-phenylpropanenitrile (1.0 eq., 1.5 mmol, 491 mg) and dry toluene (15 mL) were sealed in a microwave vial in a nitrogen-filled glovebox. PhLi as a 1.56 M solution in dibutyl ether (1.05 eq., 1.56 mmol, 1.00 mL) was added dropwise at – 78 °C and the reaction mixture was stirred at – 78 °C for 1 h before it was warmed up to room temperature and stirred for an additional 1 h. The reaction mixture was then heated to 100 °C and stirred at that temperature for 16 h. The contents of the microwave vial were cooled to room temperature and were quenched with sat. NH_4Cl (50 mL). The organic products were extracted into Et_2O (3 x 50 mL). The combined organic layers were dried over Na_2SO_4, filtered and concentrated. Purification by column chromatography (hexane → 3% EtOAc/98% hexane) afforded 3,3-dibenzyl-2-phenyl-3*H*-indole **83** (336 mg, 65%) as a white-off solid and 3-benzyl-2-phenyl-1*H*-indole (84 mg, 20%) as a yellow oil.

3,3-Dibenzyl-2-phenyl-3*H*-indole: **Mp** = 122-123 °C (lit. mp =123-125 °C).[13] **^{1}H NMR** (400 MHz, $CDCl_3$) δ 8.44 – 8.17 (m, 2H, 2 x ArH), 7.62 – 7.51 (m, 3H, 3 x ArH), 7.38 – 7.29 (m, 2H, 2 x ArH), 7.30 – 7.21 (m, 2H, 2 x ArH), 6.96 (tt, J = 7.4, 1.5 Hz, 2H, 2 x ArH), 6.88 (t, J = 7.4 Hz, 4H, 4 x ArH), 6.64 – 6.51 (m, 4H, 4 x ArH), 3.67 (d, J = 13.6 Hz, 2H, 2 x CH), 3.57 (d, J = 13.6 Hz, 2H, 2 x CH). **^{13}C NMR** (101 MHz, $CDCl_3$) δ 179.0, 154.6, 142.4, 135.6, 134.6, 130.8, 129.4, 128.9, 128.7, 128.2, 127.6, 126.6, 125.2, 123.0, 120.8, 64.6, 44.1. **ATR-IR ν_{max}** (neat)/cm^{-1} 3084, 3061, 3028, 2943, 2926, 2859, 1599, 1584, 1520, 1493, 1468, 1454, 1441, 1354, 1344, 1263, 1248, 1213, 1186, 1179, 1155, 1111, 1082, 1022, 1001, 957, 937, 914, 860, 845, 783, 758, 696, 671, 650. ***m/z* (EI)**: 373.1 (M^+, 87%), 296.1 (30), 282.1 (45), 252.1 (4), 218.1 (2), 204.1 (39), 178.0 (26), 152.0 (9), 91.0 (100), 65.0 (15), 51.0 (3). **HRMS (ESI)** calcd. for $C_{28}H_{24}N^+$ ($[M+H]^+$): 374.1903, found 374.1916.

3-Benzyl-2-phenyl-1*H*-indole: **^{1}H NMR** (400 MHz, $CDCl_3$) δ 8.13 (br s, 1H, NH), 7.60 – 7.49 (m, 2H, 2 x ArH), 7.48 – 7.39 (m, 4H, 4 x ArH), 7.36 (t, J = 7.3 Hz, 1H, ArH), 7.31 – 7.15 (m, 6H, 6 x ArH), 7.08 (t, J = 7.5 Hz, 1H, ArH), 4.29 (s, 2H, CH_2). **^{13}C NMR** (101 MHz, $CDCl_3$) δ 141.6, 136.2, 135.6, 133.1, 129.7, 129.0, 128.5, 128.4, 128.0, 127.9, 125.9, 122.5, 119.9, 119.8, 111.3, 110.9, 30.6. **ATR-IR ν_{max}** (neat)/cm^{-1} 3410, 3057, 3024, 2955, 2928, 2870, 1601, 1518, 1491, 1452, 1425, 1341, 1304, 1244, 1177, 1153, 1144, 1113, 1096, 1074, 1043, 1007, 991, 955, 914, 847, 808, 764, 741, 725, 696, 669, 627, 608. ***m/z* (EI)**: 283.0 (M^+, 15%), 206.0 (62), 190.9 (3), 178.0 (21), 165.0 (7), 151.9 (7), 139.9

(4), 128.0 (11), 115.0 (3), 103.0 (10), 91.0 (14), 77.0 (100), 65.0 (10), 51.0 (38). The data for this compound were consistent with those previously reported in the literature.[14]

Preparation of 2,3,3-triphenyl-3*H*-indole (**81**)

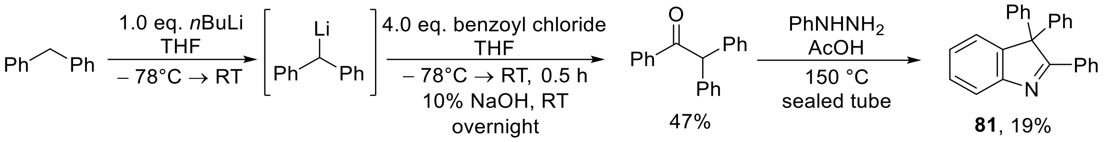

1,2,2-Triphenylethan-1-one was prepared according to a modified literature procedure.[15] ^{n}BuLi as a 2.2 M solution in hexanes (1.0 eq., 17.8 mmol, 8.100 mL) was added dropwise to a solution of diphenylmethane (1.0 eq., 17.8 mmol, 2.982 mL) in dry THF (20 mL) at – 78 °C under argon, resulting in a deep red solution. The red benzhydryllithium solution was added dropwise to a solution of benzoyl chloride (4.0 eq., 71.2 mmol, 8.258 mL) in dry THF (25 mL) at – 78 °C under argon, resulting in immediate quenching of the red colour. Reaction mixture was warmed to room temperature and stirred at room temperature for 30 min before it was carefully poured onto ice-cold 10% NaOH solution (100 mL). The resulting biphasic mixture was vigorously stirred at room temperature overnight. The mixture was then extracted with Et_2O/EtOAc mixture three times and the combined organic phases were dried over $MgSO_4$, filtered and concentrated. Recrystallisation from CH_2Cl_2/hexane afforded 1,2,2-triphenylethan-1-one (2.254 g, 47%) as a white powder. **Mp =** 129-131 °C (lit. mp = 135-136 °C).[15] **^{1}H NMR** (400 MHz, $CDCl_3$) δ 8.00 (d, *J* = 7.1 Hz, 2H, 2 x ArH), 7.51 (t, *J* = 7.4 Hz, 1H, ArH), 7.41 (t, *J* = 7.6 Hz, 2H, 2 x ArH), 7.36 – 7.22 (m, 10H, 10 x ArH), 6.04 (s, 1H, CH). **^{13}C NMR** (101 MHz, $CDCl_3$) δ 198.3, 139.2, 137.0, 133.2, 129.3, 129.1, 128.9, 128.8, 127.3, 59.6. **ATR-IR ν_{max}** (neat)/cm^{-1} 3084, 3059, 3026, 2916, 1678, 1593, 1578, 1493, 1447, 1356, 1304, 1273, 1233, 1206, 1180, 1169. 1076, 1032, 1013, 997, 858, 827, 764, 739, 692, 681, 669, 611. ***m/z* (EI)**: 272.0 (M^+, 1%), 165.1 (48), 152.1 (14), 139.1 (3), 128.0 (2), 115.0 (4), 105.1 (100), 89.0 (3), 77.0 (53), 63.0 (1), 51.0 (17). The data for this compound were consistent with those previously reported in the literature.[16]

A solution of 1,2,2-triphenylethanone (1.0 eq., 7.0 mmol, 1.906 g) and phenylhydrazine (1.0 eq., 7.0 mmol, 690 µL) in AcOH (10 mL) was refluxed overnight under argon. A further portion of phenylhydrazine (1.0 eq., 7.0 mmol, 690 µL) was added and the reaction mixture was refluxed overnight. An additional portion of phenylhydrazine (1.0 eq., 7.0 mmol, 690 µL) was added and the reaction mixture was further refluxed overnight. The reaction mixture was then cooled to RT and transferred into a microwave vial. A further portion of phenylhydrazine (0.73 eq., 5.0 mmol, 500 µL) was added and the reaction mixture was sealed in a nitrogen-filled glovebox. The reaction mixture was stirred at 150 °C overnight outside the glovebox. Two further charges of phenylhydrazine (0.73 eq., 5.0 mmol, 2 x 500 µL) were added portion wise over a few hours and the reaction mixture was stirred at 150 °C overnight. The reaction mixture was cooled to room temperature, poured onto sat. Na_2CO_3 (100 mL) and extracted into CH_2Cl_2 (4 x 40 mL). The combined organic phases were dried over $MgSO_4$,

filtered and concentrated. Two purifications by column chromatography (hexane → 10% EtOAc/90% hexane, followed by hexane → toluene) afforded 2,3,3-triphenyl-3*H*-indole **81** (457 mg, 19%) as yellow needles. **Mp** = 138-141 °C (lit. mp = 153 °C).[17] **^{1}H NMR** (400 MHz, $CDCl_3$) δ 7.97 – 7.91 (m, 2H, 2 x ArH), 7.78 (d, *J* = 7.7 Hz, 1H, ArH), 7.39 – 7.18 (m, 15H, 15 x ArH), 7.15 (td, *J* = 7.4, 1.0 Hz, 1H, ArH). **^{13}C NMR** (101 MHz, $CDCl_3$) δ 181.5, 153.9, 148.5, 140.0, 133.2, 130.6, 129.6, 128.7, 128.3, 128.1, 127.4, 126.7, 123.8, 121.4, 71.9 (one signal missing due to peak overlap). **ATR-IR ν_{max}** (neat)/cm^{-1} 3057, 3024, 2980, 1595, 1524, 1489, 1454, 1443, 1341, 1315, 1290, 1261, 1240, 1223, 1179, 1155, 1144, 1101, 1078, 1022, 1003, 976, 968, 930, 910, 885, 868, 831, 783, 768, 746, 692, 673, 654, 631. ***m/z* (EI)**: 345.2 (M^+, 100%), 267.1 (5), 241.1 (40), 226.1 (7), 215.1 (5), 165.1 (22), 133.6 (3), 119.6 (3), 103.1 (4), 77.1 (4), 51.1 (3). **HRMS (ESI)** calcd. for $C_{26}H_{20}N^+$ ($[M+H]^+$): 346.1590, found 346.1597.

Optimisation of the reaction conditions

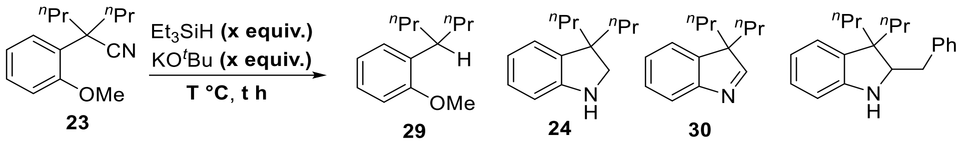

Entry	*T/°C*	*t/h*	*Silane*	*Base*	*x*	*Solvent*	**24**/%	**29**/%	**30**/%	**23**/%
1	130	18	Et_3SiH	KO^{t}Bu	3	None	32	-	-	-
2	90	18	Et_3SiH	KO^{t}Bu	3	None	59	20	-	-
3	rt	18	Et_3SiH	KO^{t}Bu	3	None	5	-	2	[a]
4	60	18	Et_3SiH	KO^{t}Bu	3	None	60	1	-	Trace
5	40	18	Et_3SiH	KO^{t}Bu	3	None	36	15	Trace	18
6	70	18	Et_3SiH	KO^{t}Bu	3	None	72	11	-	Trace
7	80	18	Et_3SiH	KO^{t}Bu	3	None	66	12	-	Trace
8	70	6	Et_3SiH	KO^{t}Bu	3	None	38	8	-	10
9	70	18	Et_3SiH	LiO^{t}Bu	3	None	-	-	-	95
10	70	18	Et_3SiH	NaO^{t}Bu	3	None	-	-	-	93
11	70	18	Et_3SiH	KHMDS	3	None	-	-	-	81
12	70	18	Et_3SiH	KOH	3	None	-	-	-	85
13	70	18	Et_3SiH	KOEt	3	None	-	-	-	68
14	70	18	Et_3SiH	Et_3N	3	None	-	-	-	66
15	70	18	Et_3SiH	NaH	3	None	-	-	-	97
16	70	18	Et_3SiH	KH	3	None	12	-	-	63
17	70	18	Et_3SiH	KO^{t}Bu	3	THF	42	34	1	-
18	70	18	Et_3SiH	KO^{t}Bu	3	dioxane	63	6	6	Trace
19	70	18	Et_3SiH	KO^{t}Bu	3	Toluene	53	25	-	Trace
20	70	18	Et_3SiH	KO^{t}Bu	3	Hexane	44	Trace	-	13
21	70	18	Et_3SiH	KO^{t}Bu	4	None	69	16	-	Trace
22	70	18	Et_3SiH	KO^{t}Bu	2	None	56	20	-	[b]
23	70	18	Me_2PhSiH	KO^{t}Bu	3	None	55	-	-	-
24	70	18	$MePh_2SiH$	KO^{t}Bu	3	None	57	-	-	-
25	70	18	Ph_3SiH	KO^{t}Bu	3	None	55	-	-	-
26	70	18	^{i}Pr$_3$SiH	KO^{t}Bu	3	None	-	-	-	98 [c]

[a] contains an inseparable mixture of **23** and an aldehyde by-product (total mass = 66 mg, 0.16:1 aldehyde:**23**). The mass of **23** at the start of the reaction was 116 mg.

[b] contains an inseparable mixture of **23** and an aldehyde by-product (total mass = 24 mg, 0.12:1 aldehyde:**23**). The mass of **23** at the start of the reaction was 116 mg.
[c] Yield determined by ^{1}H NMR using 1,3,5-trimethoxybenzene as an internal standard

To a pressure tube in the glovebox, under nitrogen, was added 2-(2-methoxyphenyl)-2-propylpentanenitrile (116 mg, 0.5 mmol, 1 equiv.), triethylsilane [(0.16 mL, 1 mmol, 2 equiv.) or (0.24 mL, 1.5 mmol, 3 equiv.) or (0.32 mL, 2 mmol, 4 equiv.)] or Me_2PhSiH (0.23 mL, 1.5 mmol, 3 equiv.) or $MePh_2SiH$ (0.30 mL, 1.5 mmol, 3 equiv.) or Ph_3SiH (0.39 mL, 1.5 mmol, 3 equiv.) or $^{i}Pr_3SiH$ (0.31 mL, 1.5 mmol, 3 equiv.), and the appropriate base {[potassium *tert*-butoxide (112 mg, 1 mmol, 2 equiv.) or (168 mg, 1.5 mmol, 3 equiv.) or (224 mg, 2 mmol, 4 equiv.)], or lithium *tert*-butoxide (120 mg, 1.5 mmol, 3 equiv.), or sodium *tert*-butoxide (144 mg, 1.5 mmol, 3 equiv.), or KHMDS (299 mg, 1.5 mmol, 3 equiv.), or potassium hydroxide (84 mg, 1.5 mmol, 3 equiv.), or potassium ethoxide (126 mg, 1.5 mmol, 3 equiv.), or triethylamine (0.21 mL, 1.5 mmol, 3 equiv.) or sodium hydride (36 mg, 1.5 mmol, 3 equiv.) or potassium hydride (60 mg, 1.5 mmol, 3 equiv.)]} with the appropriate solvent [no solvent, or THF (1 mL), 1,4-dioxane (1 mL), toluene (1 mL) or hexane (1 mL)]. The tube was sealed, removed from the glovebox and stirred at the appropriate temperature [room temperature, 40 °C, 60 °C, 70 °C, 80 °C, 90 °C or 130 °C) for the appropriate time [6 h or 18 h]. After cooling to room temperature, the reaction mixture was quenched by addition of water (50 mL). The products were extracted into diethyl ether (3 x 50 mL) and the combined organic layers were dried over a hydrophobic frit and concentrated under reduced pressure. Column chromatography (cyclohexane:ethyl acetate, 100:0 → 4:1) afforded 1-(heptan-4-yl)-2-methoxybenzene **29** as a colourless oil in the yields shown above. **^{1}H NMR** (400 MHz, $CDCl_3$) 0.85 (t, *J* = 7.3 Hz, 6 H, 2 x CH_3), 1.08 - 1.30 (m, 4 H, 2 x CH_2), 1.50 - 1.63 (m, 4 H, 2 x CH_2), 3.07 - 3.20 (m, 1 H, CH), 3.81 (s, 3 H, OCH_3), 6.86 (d, *J* = 8.3 Hz, 1 H, ArH), 6.92 (td, *J* = 7.3, 1.0 Hz, 1 H, ArH), 7.11 - 7.18 (m, 2 H, 2 x ArH). **^{13}C NMR** (101 MHz, $CDCl_3$) 14.2, 20.6, 36.8, 38.2, 55.5, 110.6, 120.5, 126.3, 127.5, 134.7, 157.7. **ATR-IR ν_{max}** (neat)/cm^{-1} 2953, 2854, 1598, 1500, 1462, 1375, 1236, 1049, 881, 738. ***m/z* (EI):** 206.2 (M^+, 59), 163.2 (60), 147.2 (6), 134.1 (10), 121.2 (100), 103.1 (13), 91.1 (93), 77.1 (26), 65.1 (24), 51.1 (7). **HRMS (CI)** calcd. for $C_{14}H_{23}O^+$ ([M+H]$^+$): 207.1749, found: 207.1745. Also isolated was recovered starting material **23**, with data consistent with those reported above. Also isolated was 3,3-dipropylindoline **24** as a colourless oil, with data consistent with those reported below. Also isolated was 3,3-dipropyl-3*H*-indole **30** as a colourless oil. **^{1}H NMR** (400 MHz, $CDCl_3$) 0.78 (t, *J* = 6.9 Hz, 6 H, 2 x CH_3), 0.83 - 0.92 (m, 2 H, 2 x CH), 0.98 - 1.12 (m, 2 H, 2 x CH), 1.70 - 1.92 (m, 4 H, 2 x CH_2), 7.22 - 7.27 (m, 2 H, 2 x ArH), 7.30 - 7.37 (m, 1 H, ArH), 7.62 (d, *J* = 7.3 Hz, 1 H, ArH), 8.03 (s, 1 H, CHN). **^{13}C NMR** (101 MHz, $CDCl_3$) 14.5, 17.7, 37.6, 62.4, 121.0, 121.8, 125.9, 127.5, 142.5, 155.8, 179.3. **ATR-IR ν_{max}** (neat)/cm^{-1} 2957, 2931, 2871, 1556, 1455, 1378, 1295, 1201, 1014, 937, 773, 753. **HRMS (CI)** calcd. for $C_{14}H_{20}N^+$ ([M+H]$^+$): 202.1596, found: 202.1601.

When toluene was used as the solvent, 2-benzyl-3,3-dipropylindoline was also isolated as a white solid (17 mg, 12%). **Mp** = 85-86 °C. **^{1}H NMR** (400 MHz, $CDCl_3$) 0.90 (t, *J* = 6.9 Hz, 3 H, CH_3), 0.94 (t, *J* = 7.1 Hz, 3 H, CH_3), 1.22 - 1.49 (m, 5 H, 2 x CH_2 + CH), 1.65 - 1.84 (m, 3 H, CH + CH_2), 2.76 (dd, *J* = 13.2, 11.3 Hz, 1 H, CH), 2.92 (dd, *J* = 13.2, 3.0 Hz, 1 H, CH), 3.78 (dd, *J* = 11.3, 2.5 Hz, 1 H, CH), 6.54 (d, *J* = 7.8 Hz, 1 H, ArH), 6.71 (td, *J* = 7.3, 1.0 Hz, 1 H, ArH), 6.91 - 7.08 (m, 2 H, 2 x ArH), 7.22 - 7.30 (m, 3 H, 3 x ArH), 7.30 - 7.38 (m, 2 H, 2 x ArH). **^{13}C NMR** (101 MHz, $CDCl_3$) 14.8, 15.0, 17.5, 17.9, 35.8, 36.5, 38.7, 49.9, 69.2, 109.3, 118.1, 124.0, 126.4, 127.1, 1287, 129.1, 135.6, 140.2, 149.6. **ATR-IR ν_{max}** (neat)/cm^{-1} 3370, 3026, 2958, 2927, 1604, 1462, 1452, 1356, 1237, 1073, 847, 764, 740, 694, 591. **HRMS (CI)** calcd. for $C_{21}H_{28}N^+$ ($[M+H]^+$): 294.2222, found: 294.2230.

Treatment of substrates

Treatment of 2-phenyl-2-propylpentanenitrile (**18**) with Et_3SiH/KO^tBu

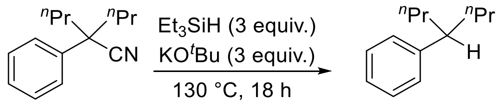

This reaction was carried out according to **General Procedure A** from **18** (101 mg, 0.5 mmol, 1 equiv.). Purification by column chromatography (hexane) afforded 4-phenylheptane **19** which was inseparable from some silyl-derived by-products. This crude mixture was then dissolved in diethyl ether (3 mL) and conc. HCl (3 mL) added. This mixture was stirred at room temperature for 48 h under air before diluting with water and extracting into hexane. The combined organic layers were washed with $NaHCO_3$, dried over Na_2SO_4, filtered and concentrated to afford 4-phenylheptane **19** as a colourless oil (38 mg, 43%) **^{1}H NMR** (400 MHz, $CDCl_3$) 0.86 (t, *J* = 7.3 Hz, 6 H, 2 x CH_3), 1.11 - 1.25 (m, 4 H, 2 x CH_2), 1.48 - 1.70 (m, 4 H, 2 x CH_2), 2.47 - 2.59 (m, 1 H, CH), 7.11 - 7.22 (m, 3 H, 3 x ArH), 7.26 - 7.33 (m, 2 H, 2 x ArH). **^{13}C NMR** (101 MHz, $CDCl_3$) 14.1, 20.7, 39.2, 45.5, 125.7, 127.7, 128.1, 146.3. **ATR-IR ν_{max}** (neat)/cm^{-1} 2953, 2924, 2870, 1610, 1492, 1452, 1377, 1099, 1066, 1029, 1008, 759, 731, 665. ***m/z* (EI):** 176.2 (M+, 13), 133.1 (25), 91.1 (100). **HRMS (CI)** calcd. for $C_{13}H_{20}^+$ (M^+): 176.1560, found: 176.1562.

Treatment of 2-phenyl-2-propylpentanenitrile (**18**) with KO^{t}Bu

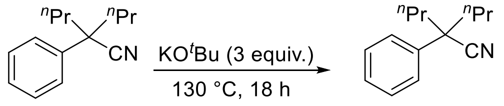

This reaction was carried out according to **General Procedure B** from **18** (101 mg, 0.5 mmol, 1 equiv.). No reaction was found to occur, and **18** was recovered with data consistent with those reported above (101 mg, 100%).

Treatment of 2-(2-methoxyphenyl)-2-propylpentanenitrile (**23**) with Et_3SiH/KO^{t}Bu

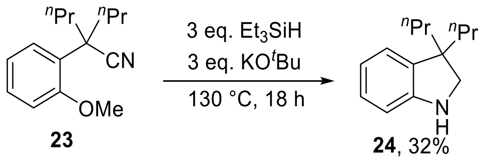

This reaction was carried out according to **General Procedure A** from **23** (116 mg, 0.5 mmol, 1 equiv.). Purification by column chromatography (hexane:diethyl ether, 100:0 → 9:1) afforded 3,3-dipropylindoline **24** as a colourless oil (33 mg, 32%). **^{1}H NMR** (400 MHz, $CDCl_3$) 0.89 (t, J = 7.3 Hz, 6 H, 2 x CH_3), 1.11 - 1.25 (m, 2 H, 2 x CH), 1.27 - 1.47 (m, 2 H, 2 x CH), 1.55 (ddd, J = 13.7, 12.2, 4.4 Hz, 2 H, 2 x CH), 1.68 (ddd, J = 13.7, 12.2, 4.4 Hz, 2 H, 2 x CH), 3.37 (s, 2 H, CH_2), 6.62 (d, J = 7.8 Hz, 1 H, ArH), 6.72 (td, J = 7.3, 1.0 Hz, 1 H, ArH), 6.99 (dd, J = 7.3, 1.0 Hz, 1 H, ArH), 7.03 (td, J = 7.3, 1.5 Hz, 1 H, ArH). **^{13}C NMR** (101 MHz, $CDCl_3$) 14.8, 17.6, 41.3, 48.8, 57.6, 109.4, 118.2, 123.3, 127.2, 136.0, 151.1. **ATR-IR ν_{max}** (neat)/cm^{-1} 3383, 2953, 2927, 2868, 1604, 1487, 1462, 1377, 1311, 1232, 1180, 1151, 1116, 1028, 896, 707. ***m/z* (EI):** 203.2 (M^+, 13) 160.2 (100), 144.1 (3), 130.1 (27), 118.1 (55), 103.1 (4), 91.1 (9), 77.1 (5), 65.1 (2), 51.1 (2). **HRMS (CI)** calcd. for $C_{14}H_{22}N^+$ ($[M+H]^+$): 204.1747, found: 204.1745.

Treatment of 2-(2-methoxyphenyl)-2-propylpentanenitrile (**23**) with KO^{t}Bu

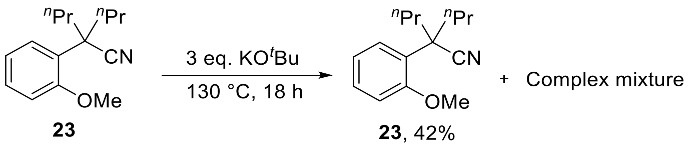

This reaction was carried out according to **General Procedure B** from **23** (116 mg, 0.5 mmol, 1 equiv.). Purification by column chromatography (hexane:diethyl ether, 100:0 → 9:1) afforded a complex mixture of products from which nothing could be identified (19 mg), and starting material **23**, with data consistent with those reported above (49 mg, 42%).

Treatment of 2-(2-fluorophenyl)-2-propylpentanenitrile (**32**) with Et_3SiH/KO^{t}Bu

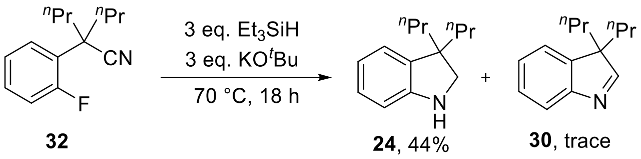

To a pressure tube in the glovebox, under nitrogen, was added 2-(2-fluorophenyl)-2-propylpentanenitrile **32** (110 mg, 0.5 mmol, 1 equiv.), triethylsilane (0.24 mL, 1.5 mmol, 3 equiv.), and potassium *tert*-butoxide (168 mg, 1.5 mmol, 3 equiv.). The tube was sealed, removed from the glovebox and stirred at 70 °C for 18 h. After cooling to room temperature, the reaction mixture was quenched by addition of water (50 mL). The products were extracted into diethyl ether (3 x 50 mL) and the combined

organic layers were dried over a hydrophobic frit and concentrated under reduced pressure. Purification by column chromatography (cyclohexane:ethyl acetate, 100:0 → 4:1) afforded, in order of elution, 3,3-dipropylindoline **24** as a colourless oil, with data consistent with those reported above (45 mg, 44%), and 3,3-dipropyl-3*H*-indole **30** as a colourless oil, with data consistent with those reported above (<1 mg).

Treatment of 2-(2-chlorophenyl)-2-propylpentanenitrile (**33**) with Et_3SiH/KO^tBu

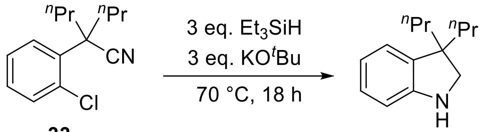

To a pressure tube in the glovebox, under nitrogen, was added 2-(2-chlorophenyl)-2-propylpentanenitrile **33** (118 mg, 0.5 mmol, 1 equiv.), triethylsilane (0.24 mL, 1.5 mmol, 3 equiv.), and potassium *tert*-butoxide (168 mg, 1.5 mmol, 3 equiv.). The tube was sealed, removed from the glovebox and stirred at 70 °C for 18 h. After cooling to room temperature, the reaction mixture was quenched by addition of water (50 mL). The products were extracted into diethyl ether (3 x 50 mL) and the combined organic layers were dried over a hydrophobic frit and concentrated under reduced pressure. Purification by column chromatography (cyclohexane:ethyl acetate, 100:0 → 4:1) afforded 3,3-dipropylindoline **24** as a colourless oil, with data consistent with those reported above (19 mg, 19%).

Treatment of 2-(2-bromophenyl)-2-propylpentanenitrile (**34**) with Et_3SiH/KO^tBu

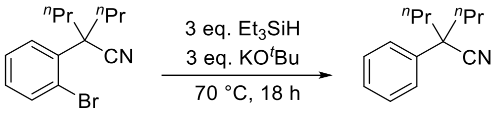

To a pressure tube in the glovebox, under nitrogen, was added 2-(2-bromophenyl)-2-propylpentanenitrile **34** (140 mg, 0.5 mmol, 1 equiv.), triethylsilane (0.24 mL, 1.5 mmol, 3 equiv.), and potassium *tert*-butoxide (168 mg, 1.5 mmol, 3 equiv.). The tube was sealed, removed from the glovebox and stirred at 70 °C for 18 h. After cooling to room temperature, the reaction mixture was quenched by addition of water (50 mL). The products were extracted into diethyl ether (3 x 50 mL) and the combined organic layers were dried over a hydrophobic frit and concentrated under reduced pressure. Purification by column chromatography (hexane) afforded an inseparable mixture of compounds from which **37** could be identified as the major component, with data consistent with those reported above. The addition of 1,3,5-trimethoxybenzene (8.4 mg, 0.05 mmol, 0.1 equiv.) allowed for calculation of the yield (57%) by comparison of the integration of the methoxy groups of the internal standard (9 integral units) to the signal at 1.92 – 2.00 ppm, corresponding to 2 x CH (11.3 integral units).

Treatment of 2-(2-iodophenyl)-2-propylpentanenitrile (**35**) with Et_3SiH/KO^tBu

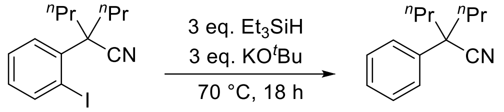

35 **37**, 78%

To a pressure tube in the glovebox, under nitrogen, was added 2-(2-iodophenyl)-2- propylpentanenitrile **35** (164 mg, 0.5 mmol, 1 equiv.), triethylsilane (0.24 mL, 1.5 mmol, 3 equiv.), and potassium *tert*-butoxide (168 mg, 1.5 mmol, 3 equiv.). The tube was sealed, removed from the glovebox and stirred at 70 °C for 18 h. After cooling to room temperature, the reaction mixture was quenched by addition of water (50 mL). The products were extracted into diethyl ether (3 x 50 mL) and the combined organic layers were dried over a hydrophobic frit and concentrated under reduced pressure. Purification by column chromatography (cyclohexane:ethyl acetate, 100:0 → 95:5) afforded 2-phenyl-2-propyl pentanenitrile **37** (79 mg, 78%) as a colourless oil, with data consistent with those reported above.

Treatment of 2-(2-(benzyloxy)phenyl)-2-propylpentanenitrile (**36**) with Et_3SiH/KO^tBu

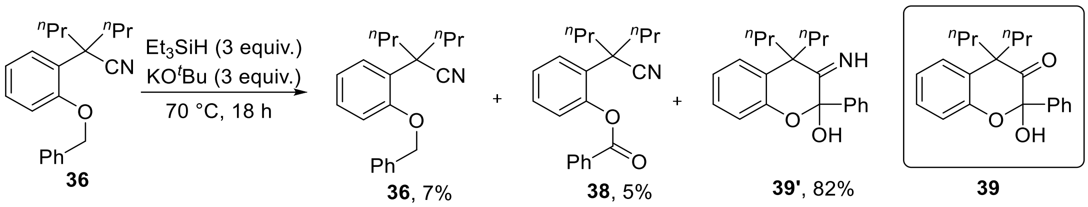

36 **36**, 7% **38**, 5% **39'**, 82% **39**

To a pressure tube in the glovebox, under nitrogen, was added 2-(2-(benzyloxy)phenyl)-2-propylpentanenitrile **36** (154 mg, 0.5 mmol, 1 equiv.), triethylsilane (0.24 mL, 1.5 mmol, 3 equiv.), and potassium *tert*-butoxide (168 mg, 1.5 mmol, 3 equiv.). The tube was sealed, removed from the glovebox and stirred at 70 °C for 18 h. After cooling to room temperature, the reaction mixture was quenched by addition of water (50 mL). The products were extracted into diethyl ether (3 x 50 mL) and the combined organic layers were dried over a hydrophobic frit and concentrated under reduced pressure. Purification by column chromatography (cyclohexane:ethyl acetate, 100:0 → 9:1) afforded recovered starting material **36** with data consistent with those reported above (10 mg, 7%), and 2-(4-cyanoheptan-4-yl)phenyl benzoate **38** as a colourless oil (8 mg, 5%). **^{1}H NMR** (400 MHz, $CDCl_3$) 0.88 (t, J = 7.3 Hz, 6 H, 2 x CH_3), 1.17 - 1.31 (m, 2 H, CH_2), 1.43 - 1.52 (m, 2 H, CH_2), 1.93 (ddd, J = 13.7, 12.2, 4.4 Hz, 2 H, CH_2), 2.08 (ddd, J = 13.7, 12.7, 4.9 Hz, 2 H, CH_2), 7.18 (dd, J = 7.8, 1.5 Hz, 1 H, ArH), 7.30 (td, J = 7.2, 1.5 Hz, 1 H, ArH), 7.37 - 7.44 (m, 1 H, ArH), 7.57 (t, J = 7.5 Hz, 2 H, 2 x ArH), 7.64 (dd, J = 7.8, 1.5 Hz, 1 H, ArH), 7.67 - 7.73 (m, 1 H, ArH), 8.19 - 8.23 (m, 2 H, 2 x ArH). **^{13}C NMR** (101 MHz, $CDCl_3$) 13.9, 18.8, 41.1, 47.7, 122.8, 124.7, 126.2, 128.9, 129.0, 129.2, 129.4, 129.7, 130.2, 134.0, 148.2, 164.8. **ATR-IR ν_{max}** (neat)/cm^{-1} 2961, 2933, 2873, 2233, 1740, 1606, 1489, 1447, 1257, 1206, 1089, 1057, 1023, 756, 706, 494. **HRMS (CI)** calcd. for $C_{21}H_{24}NO_2^+$ ([M+H]$^+$): 322.1807, found: 322.1806.

Also isolated was 3-imino-2-phenylchroman-2-ol **39'** (133 mg, 82%) as a white solid. **Mp** = 83-85 °C. **^{1}H NMR** (400 MHz, $CDCl_3$) 0.68 (t, *J* = 7.1 Hz, 3 H, CH_3), 0.99 (t, *J* = 7.3 Hz, 3 H, CH_3), 1.05 - 1.20 (m, 2 H, CH_2), 1.49 - 1.70 (m, 4 H, 2 x CH_2), 1.79 - 1.98 (m, 2 H, CH_2), 2.43 (br s, 2 H, NH + OH), 6.87 (d, *J* = 7.8 Hz, 1 H, ArH), 6.92 (td, *J* = 7.3, 1.0 Hz, 1 H, ArH), 7.09 (dd, *J* = 7.3, 1.0 Hz, 1 H, ArH), 7.17 (td, *J* = 7.8, 1.5 Hz, 1 H, ArH), 7.39 - 7.46 (m, 2 H, 2 x ArH), 7.53 (tt, *J* = 7.3, 1.5 Hz, 1 H, ArH), 8.11 - 8.17 (m, 2 H, 2 x ArH). **^{13}C NMR** (101 MHz, $CDCl_3$) 14.4, 15.0, 17.1, 17.7, 35.2, 37.5, 55.2, 105.8, 110.4, 120.9, 124.5, 128.1, 128.2, 130.0, 132.4, 133.7, 137.4, 156.0, 199.5. **ATR-IR ν_{max}** (neat)/cm^{-1} 3070, 3028, 2958, 2931, 2871, 1695, 1648, 1595, 1477, 1456, 1225, 1102, 1010, 906, 729, 699, 463. **HRMS (CI)** calcd. for $C_{21}H_{26}NO_2^+$ ([M+H]$^+$): 324.1958, found: 324.1966.

Treatment of 1-(2-methoxyphenyl)cyclopentane-1-carbonitrile (**51**) with Et_3SiH/KO^{t}Bu

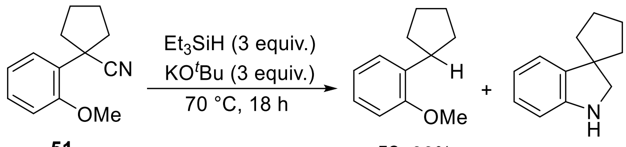

To a pressure tube in the glovebox, under nitrogen, was added 1-(2-methoxyphenyl)cyclopentane-1-carbonitrile **51** (101 mg, 0.5 mmol, 1 equiv.), triethylsilane (0.24 mL, 1.5 mmol, 3 equiv.), and potassium *tert*-butoxide (168 mg, 1.5 mmol, 3 equiv.). The tube was sealed, removed from the glovebox and stirred at 70 °C for 18 h. After cooling to room temperature, the reaction mixture was quenched by addition of water (50 mL). The products were extracted into diethyl ether (3 x 50 mL) and the combined organic layers were dried over a hydrophobic frit and concentrated under reduced pressure. Purification by column chromatography (hexane:ethyl acetate, 100:0 → 9:1) afforded 1-cyclopentyl-2-methoxy benzene **52** as a colourless oil (89 mg, 99%). **^{1}H NMR** (400 MHz, $CDCl_3$) 1.51 - 1.64 (m, 2 H, 2 x CH), 1.64 - 1.75 (m, 2 H, 2 x CH), 1.76 - 1.86 (m, 2 H, 2 x CH), 1.95 - 2.10 (m, 2 H, 2 x CH), 3.26 - 3.45 (m, 1 H, CH), 3.84 (s, 3 H, OCH_3), 6.86 (dd, *J* = 8.0, 1.0 Hz, 1 H, ArH), 6.92 (td, *J* = 7.5, 1.1 Hz, 1 H, ArH), 7.17 (td, *J* = 7.7, 1.6 Hz, 1 H, ArH), 7.23 (dd, *J* = 7.5, 1.8 Hz, 1 H, ArH). **^{13}C NMR** (101 MHz, $CDCl_3$) 25.4, 33.0, 39.0, 55.4, 110.4, 120.4, 126.5, 126.7, 134.6, 157.4. **ATR-IR ν_{max}** (neat)/cm^{-1} 2949, 2866, 1598, 1583, 1490, 1462, 1436, 1359, 1313, 1288, 1238, 1174, 1111, 1053, 1029, 923, 748. ***m/z* (EI):** 176.2 (M$^+$, 84), 161.1 (17), 147.1 (100), 134.1 (27), 121.1 (35), 105.1 (18), 91.1 (87), 77.1 (23), 65.1 (19), 51.1 (12). The data for this compound are consistent with those previously reported in the literature.[18] Also tentatively identified was a trace amount of impure spiro[cyclopentane-1,3'-indoline] **53** (<1%). **^{1}H NMR** (400 MHz, $CDCl_3$) 1.66 - 1.78 (m, 2 H, 2 x CH), 1.78 - 1.93 (m, 6 H, 6 x CH), 3.39 (s, 2 H, NCH_2), 6.68 (d, *J* = 7.8 Hz, 1 H, ArH), 6.77 (td, *J* = 7.5, 1.3 Hz, 1 H, ArH), 7.03 (dd, *J* = 7.8, 1.5 Hz, 1 H, ArH), 7.06 - 7.12 (m, 1 H, ArH). The ^{1}H NMR spectrum for this compound is consistent with that reported in the literature.[19]

Treatment of 1-(2-methoxyphenyl)cyclohexane-1-carbonitrile (20) with Et_3SiH/KO^{t}Bu

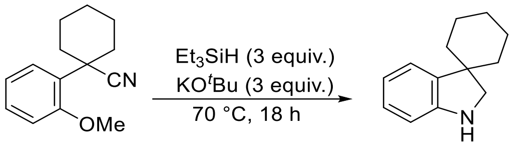

To a pressure tube in a glovebox under nitrogen was added 1-(2-methoxyphenyl)cyclohexane-1-carbonitrile **20** (108 mg, 0.5 mmol, 1 equiv.), triethylsilane (0.24 mL, 1.5 mmol, 3 equiv.) and potassium *tert*-butoxide (168 mg, 1.5 mmol, 3 equiv.). The tube was sealed, removed from the glovebox and heated at 70 °C for 18 h behind a shield. After cooling to room temperature, the mixture was quenched with water (50 mL) and extracted into diethyl ether (3 x 50 mL). The combined organic layers were dried over Na_2SO_4, filtered and concentrated under reduced pressure. Purification by column chromatography (hexane:ethyl acetate, 100:0 → 9:1) spiro[cyclohexane-1,3'-indoline] **54** as an orange solid (60 mg, 64%). **Mp** = 69-70 °C (lit. mp = 76-77 °C).[20] **^{1}H NMR** (400 MHz, $CDCl_3$) 1.23 - 1.47 (m, 3 H, 3 x CH), 1.54 - 1.67 (m, 2 H, 2 x CH), 1.67 - 1.82 (m, 5 H, 5 x CH), 3.44 (s, 2 H, NCH_2), 6.65 (d, J = 7.6 Hz, 1 H, ArH), 6.75 (t, J = 7.3 Hz, 1 H, ArH), 7.04 (td, J = 7.6, 1.1 Hz, 1 H, ArH), 7.07 (d, J = 7.5 Hz, 1 H, ArH). **^{13}C NMR** (101 MHz, $CDCl_3$) 23.2, 25.8, 36.4, 46.1, 56.7, 109.6, 118.6, 122.5, 127.4, 138.4, 150.4. **ATR-IR ν_{max}** (neat)/cm^{-1} 3201, 2920, 2850, 1602, 1485, 1448, 1325, 1247, 1190, 1022, 933, 964, 871, 839, 825, 740. ***m/z* (EI):** 187.1 (M^+, 22), 144.1 (19), 130.1 (100), 117.1 (14), 103.1 (4), 90.0 (4), 77.0 (5), 63.0 (1), 56.1 (1), 51.0 (1). The data for this compound are consistent with those previously reported in the literature.[19]

Treatment of 2-(2-methoxypyridin-3-yl)-2-propylpentanenitrile (55) with Et_3SiH/KO^{t}Bu

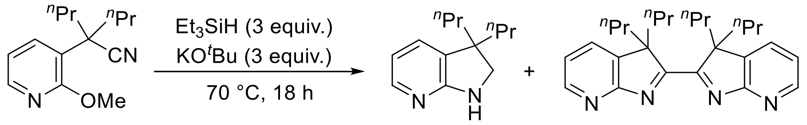

To a pressure tube in a glovebox under nitrogen was added 2-(2-methoxypyridin-3-yl)-2-propylpentanenitrile **55** (116 mg, 0.5 mmol, 1 equiv.), triethylsilane (0.24 mL, 1.5 mmol, 3 equiv.) and potassium *tert*-butoxide (168 mg, 1.5 mmol, 3 equiv.). The tube was sealed, removed from the glovebox and heated at 70 °C for 18 h behind a shield. After cooling to room temperature, the mixture was quenched with water (50 mL) and extracted into diethyl ether (3 x 50 mL). The combined organic layers were dried over Na_2SO_4, filtered and concentrated under reduced pressure. Purification by column chromatography (hexane:ethyl acetate, 100:0 → 7:3) afforded starting material **55** as a colourless oil, with data consistent with those reported above (23 mg, 20%) and 3,3,3',3'-tetrapropyl-3*H*,3'*H*-2,2'-bipyrrolo[2,3-b]pyridine **57** as a yellow solid (31 mg, 15%). **Mp** = 192 °C (decomposition). **^{1}H NMR** (400 MHz, $CDCl_3$) 0.58 - 0.80 (m, 20 H, 4 x CH_3 + 4 x CH_2), 2.04 - 2.18 (m, 4 H, 4 x CH), 2.59 - 2.75 (m, 4 H, 4 x CH), 7.25 - 7.30 (m, 2 H, 2 x ArH), 7.71 (dd, J = 7.4, 1.6 Hz, 2 H, 2 x ArH), 8.59 (dd, J =

4.9, 1.6 Hz, 2 H, 2 x ArH). **^{13}C NMR** (101 MHz, $CDCl_3$) 14.1, 17.6, 39.5, 64.2, 121.9, 130.5, 138.2, 148.7, 168.0, 181.0. **ATR-IR ν_{max}** (neat)/cm^{-1} 2958, 2927, 2870, 2285, 1581, 1462, 1392, 1226, 1134, 1054, 1029, 1014, 902, 800, 729, 623. ***m/z* (EI):** 402.4 (M^+, 2), 373.4 (100), 360.3 (65), 331.3 (75), 317.3 (98), 301.2 (12), 287.2 (23), 271.2 (30), 259.2 (44), 201.2 (52), 173.1 (35), 159.1 (13), 144.1 (10), 130.1 (14), 117.1 (19), 104.1 (4), 92.1 (4), 77.1 (5), 65.1 (2), 51.1 (1). **HRMS (CI)** calcd. for $C_{26}H_{35}N_4^+$ ($[M+H]^+$): 403.2856, found: 403.2854.

Also isolated was 3,3-dipropyl-2,3-dihydro-1*H*-pyrrolo[2,3-b]pyridine **56** as a yellow semi-solid (24 mg, 24%). **^{1}H NMR** (400 MHz, $CDCl_3$) 0.90 (t, *J* = 7.5 Hz, 6 H, 2 x CH_3), 1.12 - 1.24 (m, 2 H, 2 x CH), 1.30 - 1.40 (m, 2 H, 2 x CH), 1.51 - 1.61 (m, 2 H, 2 x CH), 1.61 - 1.71 (m, 2 H, 2 x CH), 3.40 (s, 2 H, CH_2N), 4.50 (br s., 1 H, NH), 6.53 (dd, *J* = 7.0, 5.3 Hz, 1 H, ArH), 7.14 (dd, *J* = 7.0, 1.5 Hz, 1 H, ArH), 7.79 - 7.91 (m, 1 H, ArH). **^{13}C NMR** (101 MHz, $CDCl_3$) 14.6, 17.4, 41.4, 47.4, 54.8, 113.0, 128.7, 130.4, 145.7, 163.7. **ATR-IR ν_{max}** (neat)/cm^{-1} 3224, 2956, 2927, 2870, 1654, 1608, 1463, 1421, 1249, 767, 734. ***m/z* (EI):** 204.2 (M^+, 12), 173.1 (7), 161.1 (100), 145.1 (5), 131.1 (31), 119.1 (26), 104.1 (5), 92.0 (4), 77.0 (4), 65.0 (2), 51.0 (2). **HRMS (CI)** calcd. for $C_{13}H_{21}N_2^+$ ($[M+H]^+$): 205.1705, found: 205.1701.

Treatment of 2-ethyl-2-(2-methoxyphenyl)butanenitrile (**25**) with Et_3SiH/KO^{t}Bu

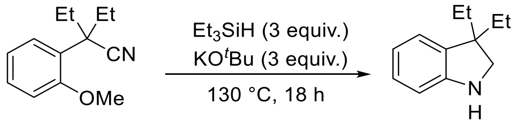

25 **26**, 25%

Preparation *via* **General Procedure A** from 2-ethyl-2-(2-methoxyphenyl)butanenitrile **25** (101 mg, 0.50 mmol, 1.00 eq.), Et_3SiH (240 µL, 1.5 mmol, 3.0 eq.) and KO^{t}Bu (168.32 mg, 1.5 mmol, 3.0 eq.). Purification by column chromatography (0-5% Et_2O/hexane), followed by concentration under reduced pressure afforded 3,3-diethylindoline **26** as an orange/brown oil (22 mg, 25%). **^{1}H NMR** (400 MHz, $CDCl_3$) δ 7.04 (td, J = 7.6, 1.3 Hz, 1H, ArH), 6.98 (dd, J = 7.4, 0.8 Hz, 1H, ArH), 6.73 (td, J = 7.4, 1.0 Hz, 1H, ArH), 6.63 (d, J = 7.8 Hz, 1H, ArH), 3.36 (s, 2H, CH_2), 1.78 – 1.68 (m, 2H, CH_2), 1.68 – 1.58 (m, 2H, CH_2), 0.84 (t, J = 7.5 Hz, 6H, 2 x CH_3). **^{13}C NMR** (101 MHz, $CDCl_3$) δ 151.4, 135.3, 127.2, 123.5, 118.2, 109.4, 56.8, 49.3, 30.6, 8.7. **ATR-IR ν_{max}** (neat)/cm^{-1} 3373, 2962, 2914, 2875, 2856, 1606, 1545, 1454. ***m/z* (EI):** 175.2 (M^+, 28), 146.2 (100), 130.1 (58), 118.1 (74). The data for this compound are consistent with those previously reported in the literature.[21]

Treatment of 2-ethyl-2-(2-ethoxyphenyl)butanenitrile (**31**) with Et_3SiH/KO^{t}Bu

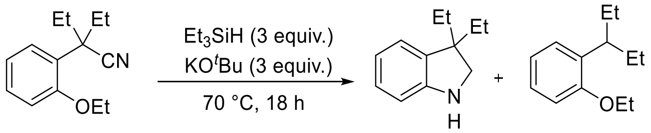

31 **24**, 65% **29**, 8%

To an oven-dried pressure tube, equipped with a stirrer bar, 2-ethyl-2-(2-ethoxyphenyl) butanenitrile **31** (109 mg, 0.5 mmol, 1 equiv.) was added. The tube was taken into the glovebox, where KO^{t}Bu (168 mg, 1.5 mmol, 3 equiv.) followed by Et_3SiH (229 μL, 1.5 mmol, 3 equiv.) was added and then sealed. The pressure tube was removed from the glovebox and heated at 70 °C for 18 h. Upon completion, the reaction was cooled to RT and quenched with water. The aqueous layer was extracted with Et_2O (x 3) and the combined organic layers were dried over Na_2SO_4, filtered and reduced *in vacuo*. The residue was purified by column chromatography (100% hexane to 1:2 hexane:Et_2O) affording 3,3-diethylindoline **24** as a yellow oil (66 mg, 65%) and 1-ethoxy-2-(pentan-3-yl)benzene **29** as a colourless oil (8 mg, 8%).

3,3-Diethylindoline: **^{1}H NMR** (400 MHz, $CDCl_3$) δ 0.83 (t, *J* = 7.5 Hz, 6H, CH_3), 1.52 - 1.78 (m, 4H, CH_2), 3.35 (s, 2H, CH_2), 6.62 (d, *J* = 7.7, 1H, ArH), 6.67 - 6.76 (m, 1H, ArH), 6.93 - 7.01 (m, 1H, ArH), 6.98 - 7.07 (td, 1H, ArH). **^{13}C NMR** (101 MHz, $CDCl_3$) δ 8.9 (CH_3), 30.7 (CH_2), 49.4 (C), 56.9 (CH_2), 109.5 (CH), 118.3 (CH), 123.6 (CH), 127.4 (CH), 135.4 (C), 151.6 (C). **ATR-IR ν_{max}** (neat)/cm^{-1} 3385, 1601, 1530, 1479, 1454, 1410, 1377, 1285, 1244, 1182, 1022, 739. ***m/z* (EI):** 175.3 (M^+, 17), 146.2 (100), 130.1 (54), 118.2 (61), 103.0 (11), 90.1 (17), 77.1 (16), 63.2 (11), 51.1 (7).

1-Ethoxy-2-(pentan-3-yl)benzene: **^{1}H NMR** (400 MHz, $CDCl_3$) δ 0.78 (t, *J* = 7.4 Hz, 6H, CH_3), 1.37 - 1.43 (m, 3H, CH_3), 1.49 - 1.74 (m, 4H, CH_2), 2.86 - 3.00 (m, 1H, CH), 4.01 (q, *J* = 7.0 Hz, 2H, CH_2), 6.84 (d, *J* = 8.1 Hz, 1H, ArH), 6.90 (t, *J* = 7.4 Hz, 1H, ArH), 7.07 - 7.18 (m, 2H, ArH). **^{13}C NMR** (101 MHz, $CDCl_3$) δ 12.2 (CH_3), 15.1 (CH_3), 28.1 (CH_2), 41.3 (CH), 63.8 (CH_2), 111.7 (CH), 120.5 (CH), 126.4 (CH), 127.7 (CH), 134.4 (C), 157.4 (C). **ATR-IR ν_{max}** (neat)/cm^{-1} 1597, 1584, 1491, 1450, 1391, 1287, 1234, 1150, 1117, 1047, 924, 743. ***m/z* (EI):** 192.2 (M^+, 27), 163.2 (88), 135.1 (72), 115.1 (20), 107.1 (100), 91.1 (55), 77.1 (27), 51.1 (7).

Treatment of 2-ethyl-2-(2-ethoxyphenyl)butanenitrile (**31**) with KO^{t}Bu

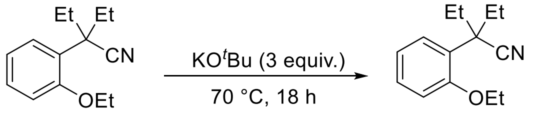

31 **31**, 100%

To an oven-dried pressure tube, equipped with a stirrer bar, 2-ethyl-2-(2-ethoxyphenyl) butanenitrile (109 mg, 0.5 mmol, 1 equiv.) was added. The tube was taken into the glovebox, where KO^{t}Bu (168 mg, 1.5 mmol, 3 equiv.) was added and then sealed. The pressure tube was removed from the glovebox and heated at 70 °C for 18 h. Upon completion, the reaction was cooled to RT and quenched with water. The aqueous layer was extracted with Et_2O (x 3) and the combined organic layers were dried over Na_2SO_4, filtered and reduced *in vacuo*. Starting material **31** was obtained as a yellow oil (109 mg, 100%). Recovered 2-(2-ethoxyphenyl)-2-ethylbutanenitrile **31** gave spectroscopic data that were consistent with those reported above.

Treatment of 3-(2-methoxyphenyl)-2,2-dimethylpropanenitrile (27) with Et_3SiH/KO^{t}Bu

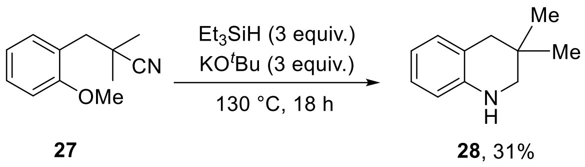

Preparation *via* **General Procedure A** from 3-(2- methoxyphenyl)-2,2-dimethylpropanenitrile **27** (95 mg, 0.50 mmol, 1.00 eq.), Et_3SiH (240 µL, 1.5 mmol, 3.0 eq.) and KO^{t}Bu (168.32 mg, 1.5 mmol, 3.0 eq.). Purification by column chromatography (0-5% Et_2O/hexane), followed by concentration under reduced pressure afforded 3,3-dimethyl-1,2,3,4-tetrahydroquinoline **28** as a colourless oil (25 mg, 31%). **^{1}H NMR** (400 MHz, $CDCl_3$) δ 7.03 – 6.97 (m, 1H, ArH), 6.95 (dd, *J* = 7.4, 0.9 Hz, 1H, ArH), 6.63 (td, *J* = 7.4, 1.1 Hz, 1H, ArH), 6.51 (dd, *J* = 8.0, 0.9 Hz, 1H, ArH), 3.87 (br s, 1H, NH), 2.94 (t, *J* = 0.8 Hz, 2H, CH_2), 2.52 (s, 2H, CH_2), 1.04 (s, 6H, 2 x CH_3). **^{13}C NMR** (101 MHz, $CDCl_3$) δ 143.5, 130.1, 126.8, 120.7, 117.0, 113.8, 53.4, 41.3, 28.2, 26.6. **ATR-IR ν_{max}** (neat)/cm^{-1} 3358, 2954, 2901, 2862, 2853, 1606, 1527, 1455. ***m/z* (EI):** 161.2 (54), 146.8 (11), 137.0 (5), 121.0 (100), 114.8 (5), 108.0 (10), 103.7 (6), 98.8 (36), 91.2 (58), 82.5 (30), 77.0 (58), 70.5 (16), 65.3 (32), 56.9 (81), 50.7 (18). The data for this compound are consistent with those previously reported in the literature.[22]

Treatment of 2-(2-methoxyphenyl)-2-propylpentanenitrile (23) with MeMgBr

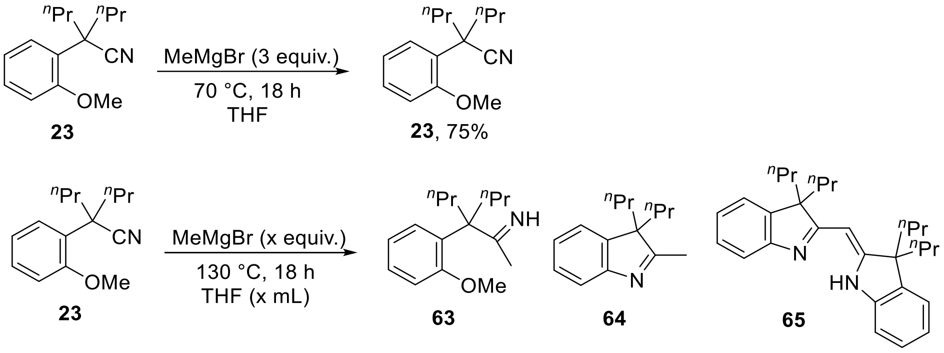

To a pressure tube in the glovebox, under nitrogen, was added 2-(2-methoxyphenyl)-2-propylpentanenitrile **23** (116 mg, 0.5 mmol, 1 equiv.) and methylmagnesium bromide (3.6 M in 2-methylTHF, 0.44 mL, 1.5 mmol, 3 equiv.) and THF (0.56 mL). The tube was sealed, removed from the glovebox and stirred at 70 °C for 18 h. After cooling to room temperature, the reaction mixture was quenched by addition of water (50 mL). The products were extracted into diethyl ether (3 x 50 mL) and the combined organic layers were dried over a hydrophobic frit and concentrated under reduced pressure. Purification by column chromatography (cyclohexane:ethyl acetate, 100:0 → 9:1) afforded starting material **23** only (87 mg, 75%).

Entry	x	**63** / %	**64** / %	**65** / %
1	3	40	-	9
2	2	24	-	4

3	4	37	28	14
4	10	15	40	Trace

To a pressure tube in the glovebox, under nitrogen, was added 2-(2-methoxyphenyl)-2-propylpentanenitrile **23** (116 mg, 0.5 mmol, 1 equiv.) and methylmagnesium bromide [3.6 M in 2-methylTHF, [(0.28 mL, 1 mmol, 2 equiv.), or (0.42 mL, 1.5 mmol, 3 equiv.), or (0.56 mL, 2 mmol, 4 equiv.) or (1.39 mL, 10 mmol, 10 equiv.)] and THF (0.72 mL or 0.58 mL or 0.44 mL or 0 mL respectively). The tube was sealed, removed from the glovebox and stirred at 130 °C for 18 h. After cooling to room temperature, the reaction mixture was quenched by addition of water (50 mL). The products were extracted into diethyl ether (3 x 50 mL) and the combined organic layers were dried over a hydrophobic frit and concentrated under reduced pressure. Purification by column chromatography (cyclohexane:ethyl acetate, 100:0 → 0:0100) afforded 2-((3,3-dipropylindolin-2-ylidene)methyl)-3,3-dipropyl-3*H*-indole **65** as a yellow solid. **Mp** = 155-158 °C. **^{1}H NMR** (400 MHz, $CDCl_3$) 0.65 - 0.80 (m, 16 H, 4 x CH_3 + 2 x CH_2), 0.89 - 1.02 (m, 4 H, 2 x CH_2), 1.65 - 1.76 (m, 4 H, 2 x CH_2), 1.85 - 1.96 (m, 4 H, 2 x CH_2), 5.08 (s, 1 H, CH), 7.01 (td, *J* = 7.1, 1.5 Hz, 2 H, 2 x ArH), 7.16 (d, *J* = 6.9 Hz, 2 H, 2 x ArH), 7.18 - 7.21 (m, 2 H, 2 x ArH), 7.21 - 7.26 (m, 2 H, 2 x ArH). **^{13}C NMR** (101 MHz, $CDCl_3$) 14.3, 17.4, 42.5, 58.2, 82.6, 113.4, 121.7, 121.9, 127.5, 137.8, 150.9, 174.3. **ATR-IR ν_{max}** (neat)/cm^{-1} 3063, 2952, 2927, 2903, 2871, 2844, 1599, 1492, 1448, 1426, 1332, 1227, 1180, 1097, 1013, 772, 745, 677. ***m/z* (EI):** 414.4 (M^+, 85), 385.3 (81), 371.3 (100), 342.3 (8), 329.2 (15), 313.2 (8), 299.2 (26), 285.1 (36), 269.1 (71), 256.1 (62), 241.1 (1), 228.1 (1), 214.2 (48), 200.2 (36), 185.2 (5), 170.1 (55), 158.1 (14), 144.1 (11), 130.1 (12), 115.5 (4), 103.1 (1), 91.1 (1), 77.1 (1), 65.1 (1), 51.1 (1). **HRMS (CI)** calcd. for $C_{29}H_{39}N_2^+$ ($[M+H]^+$): 415.3113, found: 415.3112.

Also isolated was 2-methyl-3,3-dipropyl-3*H*-indole **64** as a colourless oil. **^{1}H NMR** (400 MHz, $CDCl_3$) 0.57 - 0.67 (m, 2 H) 0.70 - 0.83 (m, 8 H) 1.64 - 1.78 (m, 2 H) 1.81 - 1.95 (m, 2 H) 2.26 (s, 3 H) 7.18 - 7.24 (m, 2 H) 7.29 - 7.35 (m, 1 H) 7.54 (d, *J* = 7.5 Hz, 1 H). **^{13}C NMR** (101 MHz, $CDCl_3$) 14.2, 16.0, 17.0, 39.2, 62.8, 119.5, 121.6, 124.8, 127.4, 142.3, 155.0, 186.5. **ATR-IR ν_{max}** (neat)/cm^{-1} 2956, 2872, 2846, 1575, 1456, 1377, 756. ***m/z* (EI):** 215.2 (M^+, 39), 200.5 (5), 186.2 (68), 172.2 (38), 157.1 (28), 144.1 (100), 128.1 (12), 115.1 (20), 102.1 (9), 91.1 (9), 77.1 (9), 63.1 (3), 51.1 (3). **HRMS (CI)** calcd. for $C_{15}H_{22}N^+$ ($[M+H]^+$): 216.1752, found: 216.1755.

Also isolated was 3-(2-methoxyphenyl)-3-propylhexan-2-imine **63** as a yellow oil. **^{1}H NMR** (400 MHz, $CDCl_3$) 0.82 - 0.98 (m, 8 H, 2 x CH_3 + 2 x CH), 1.04 - 1.19 (m, 2 H, 2 x CH), 1.63 - 1.79 (m, 2 H, 2 x CH), 1.82 (s, 3 H, CH_3), 1.94 - 2.09 (m, 2 H, 2 x CH), 3.72 (s, 3 H, OCH_3), 6.84 (dd, *J* = 8.2, 1.1 Hz, 1 H, ArH), 6.95 (td, *J* = 7.6, 1.1 Hz, 1 H, ArH), 7.21 - 7.27 (m, 1 H, ArH), 7.29 (dd, *J* = 7.8, 1.5 Hz, 1 H, ArH). **^{13}C NMR** (101 MHz, $CDCl_3$) 14.8, 17.4, 23.8, 34.7, 53.0, 55.0, 111.3, 120.3, 127.8, 128.0, 133.0, 157.4, 185.6. **ATR-IR ν_{max}** (neat)/cm^{-1} 2956, 2870, 1633, 1489, 1435, 1373, 1321, 1290, 1238, 1153,

1099, 1028, 902, 877, 846, 746. ***m/z* (EI):** 247.2 (M^+, 1), 216.2 (81), 206.1 (8), 174.1 (15), 163.1 (25), 144.1 (11), 131.1 (14), 121.1 (100), 115.1 (14), 105.1 (14), 91.1 (45), 77.0 (11), 65.0 (5), 55.0 (4). **HRMS (CI)** calcd. for $C_{16}H_{26}NO^+$ ($[M+H]^+$): 248.2014, found: 248.2017.

Treatment of 2-(2-methoxyphenyl)-2-propylpentanenitrile (**23**) with PhMgBr

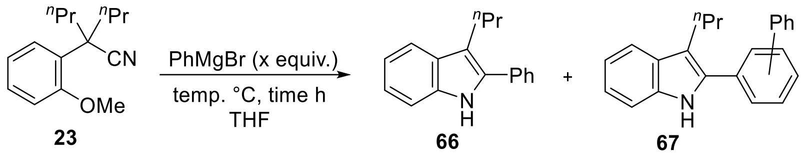

Entry	x	Temp. / °C	Time / h	**66** / %	**67** / %	**23** / %
1	3	130	18	30	30	2
2	2	130	18	28	22	5
3	1	130	18	7	5	52
4	2	130	6	6	3	78
5	2	100	18	3	3	87

To a pressure tube in the glovebox under nitrogen was added 2-(2-methoxyphenyl)-2-propylpentanenitrile **23** (116 mg, 0.5 mmol, 1 equiv.) and phenylmagnesium bromide [1 M in THF, (1.5 mL, 1.5 mmol, 3 equiv.) or (1 mL, 1 mmol, 2 equiv.) or (0.5 mL, 0.5 mmol, 1 equiv.)] and THF (0 mL, 0.5 mL or 1 mL respectively). The tube was removed and stirred at the appropriate temperature (100 °C or 130 °C) for the appropriate time (6 h or 18 h) behind a shield. After cooling to room temperature, the mixture was quenched with water (50 mL) and extracted into diethyl ether (3 x 50 mL). The combined organic layers were dried over a hydrophobic frit and concentrated under reduced pressure. 1,3,5-Trimethoxybenzene (8.4 mg, 0.05 mmol, 0.1 equiv.) was added as an internal standard to determine the yield of both compounds **66** and **67** by comparison of the integration of the methoxy group of the internal standard (3.80 ppm, 9 integral units) to the CH_2 of **66** and **67** [2.91 ppm (5.99 integral units) and 2.62 ppm (5.98 integral units) respectively]. From one experiment (3 equiv. of PhMgBr, 130 °C, 18 h), compounds **66** and **67** were purified by preparative HPLC to afford 2-phenyl-3-propyl-1*H*-indole **66** as a white solid (33 mg, 28%). **Mp** = 77-79 °C (lit. mp = 78-79 °C).[11] **^{1}H NMR** (400 MHz, $CDCl_3$) 1.01 (t, J = 7.4 Hz, 3 H, CH_3), 1.78 (sxt, J = 7.8 Hz, 2 H, CH_2), 2.82 - 2.94 (m, 2 H, CH_2), 7.10 - 7.18 (m, 1 H, ArH), 7.22 (td, J = 7.5, 1.3 Hz, 1 H, ArH), 7.34 - 7.42 (m, 2 H, ArH), 7.45 - 7.52 (m, 2 H, 2 x ArH), 7.54 - 7.61 (m, 2 H, 2 x ArH), 7.66 (dd, J = 7.8, 0.8 Hz, 1 H, ArH), 7.99 (br s., 1 H, NH). **^{13}C NMR** (101 MHz, $CDCl_3$) 14.4, 24.2, 26.7, 110.7, 114.0, 119.4, 119.4, 122.2, 127.5, 127.9, 128.8, 129.4, 133.5, 134.1, 135.9. **ATR-IR ν_{max}** (neat)/cm^{-1} 3400, 3055, 2956, 2868, 1604, 1537, 1487, 1446, 1340, 1305, 1072, 906, 738, 696. ***m/z* (EI):** 235.1 (M^+, 28), 206.1 (100), 178.1 (15), 165.0 (3), 152.1 (3), 128.0 (4), 115.0 (3), 102.1 (7), 89.0 (3), 77.0 (8), 63.0 (3), 51.0 (4) and 2-(biphenyl)-3-propyl-1*H*-indole **67** as a white solid (41 mg, 26%). **Mp** = 90-92 °C. **^{1}H NMR** (400 MHz, $CDCl_3$) 0.90

(t, J = 7.3 Hz, 3 H, CH_3), 1.54 (sxt, J = 7.5 Hz, 2 H, CH_2), 2.56 - 2.65 (m, 2 H, CH_2), 7.03 - 7.18 (m, 3 H, 3 x ArH), 7.19 - 7.25 (m, 5 H, 5 x ArH), 7.41 - 7.56 (m, 5 H, 4 x ArH + NH), 7.58 (d, J = 7.3 Hz, 1 H, ArH). **^{13}C NMR** (101 MHz, $CDCl_3$) 14.5, 23.5, 26.8, 110.5, 114.5, 119.0, 119.3, 121.6, 127.0, 127.3, 128.3, 128.4, 128.5, 128.8, 130.6, 131.4, 131.6, 133.6, 135.7, 140.9, 141.3. **ATR-IR ν_{max}** (neat)/cm^{-1} 3402, 2954, 2927, 1479, 1456, 1425, 1305, 1008, 738, 700. ***m/z* (EI):** 311.2 (M_+, 42), 282.1 (100), 267.1 (19), 254.1 (5), 239.1 (3), 204.1 (7), 165.1 (7), 152.1 (3), 139.2 (3), 77.1 (4), 63.0 (1), 51.1 (1). **HRMS (CI)** calcd. for $C_{23}H_{21}N^+$ ($[M+H]^+$): 312.1747, found: 312.1748.

Treatment of 3,3-dimethyl-2-phenyl-3*H*-indole (72) with Et_3SiH/KO^{t}Bu

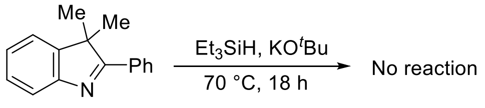

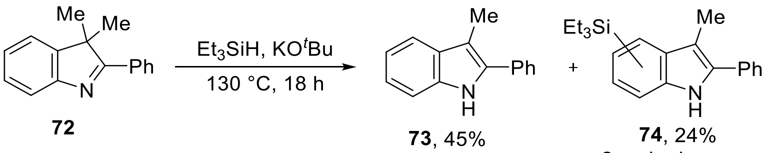

Carried out according to **General Procedure A** using 3,3-dimethyl-2-phenyl-3*H*-indole **72** (1.0 eq., 0.5 mmol, 110.65 mg), Et_3SiH (3.0 eq., 1.5 mmol, 240 µL) and KO^{t}Bu (3.0 eq., 1.5 mmol, 168.32 mg). Purification by column chromatography (petroleum ether → 1% EtOAc/99% petroleum ether) followed by another purification by column chromatography (petroleum ether → 2% Et_2O/98% petroleum ether) afforded an isomeric mixture of silylated 3-methyl-2-phenyl-1*H*-indole **74** (2 major isomers) (38 mg, 24%) as a colourless oil and 3-methyl-2-phenyl-1*H*-indole **73**(47 mg, 45%) as a white solid.

Isomeric mixture of silylated 3-methyl-2-phenyl-1*H*-indole: **^{1}H NMR** (400 MHz, $CDCl_3$) δ 8.03 (br s, 1H, NH), 7.73 (s, 1H, ArH), 7.64 – 7.44 (m, 3H, 3 x ArH), 7.53 – 7.44 (m, 1H, ArH), 7.39 (t, J = 7.4 Hz, 1H, ArH), 7.29 – 7.12 (m, 2H, 2 x ArH), 2.50 (2 x s, 3H, Me), 1.04 (t, J = 7.8 Hz, 9H, 3 x $RSiCH_2\mathit{CH_3}$), 0.87 (2 x q, J = 7.6, 6H, 3 x $RSi\mathit{CH_2}CH_3$). **^{13}C NMR** (101 MHz, $CDCl_3$) δ 138.4, 136.8, 136.0, 136.0, 134.8, 134.2, 133.7, 133.7, 133.3, 132.7, 130.3, 130.2, 129.0, 128.2, 126.9, 122.5, 122.4, 119.7, 119.6, 119.1, 119.1, 110.8, 109.0, 108.7, 9.9, 9.8, 7.6, 3.5. **ATR-IR ν_{max}** (neat)/cm^{-1} 3291, 3051, 2951, 2934, 2909, 2872, 1584, 1522, 1450, 1414, 1381, 1364, 1314, 1261, 1236, 1196, 1107, 1092, 1055, 1009, 943, 864, 833, 800, 729, 718, 698. ***m/z* (EI) isomer 1:** 321.1 (M^+, 100%), 292.0 (2), 264.1 (48), 236.0 (52), 204.1 (36), 178.1 (6), 146.0 (4), 118.0 (44), 77.0 (9), 51.0 (1). ***m/z* (EI) isomer 2:** 321.1 (M^+, 94%), 292.1 (12), 264.1 (29), 236.0 (100), 204.0 (17), 178.0 (6), 146.0 (2), 117.9 (47), 77.0 (8), 51.0 (1).

3-Methyl-2-phenyl-1*H*-indole: **Mp =** 111-114 °C (lit. mp 93-95 °C).[23] **^{1}H NMR** (400 MHz, $CDCl_3$) δ 8.01 (br s, 1H, NH), 7.70 – 7.57 (m, J = 13.6, 7.8 Hz, 3H, 3 x ArH), 7.54 – 7.44 (m, 2H, 2 x ArH), 7.38 – 7.35 (m, 2H, 2 x ArH), 7.29 – 7.13 (m, 2H, 2 x ArH), 2.48 (s, 3H, Me). **^{13}C NMR** (101 MHz, $CDCl_3$)

δ 136.0, 134.2, 133.5, 130.2, 129.0, 127.9, 127.5, 122.5, 119.7, 119.1, 110.8, 108.9, 9.8. **ATR-IR ν_{max}** (neat)/cm^{-1} 3311, 3055, 3030, 2976, 2924, 2860, 1533, 1489, 1445, 1368, 1335, 1314, 1304, 1263, 1246, 1234, 1196, 1094, 1003, 941, 862, 768, 741, 696, 623, 613. ***m/z* (EI):** 207.0 (M$^+$, 100%), 177.9 (7), 151.8 (2), 140.9 (2), 129.9 (33), 114.8 (2), 102.1 (17), 89.0 (8), 77.0 (80), 63.0 (27), 51.0 (43). The data are consistent with those previously reported in the literature[24]

Treatment of 3,3-dimethyl-2-phenyl-3*H*-indole (**72**) with KO*t*Bu

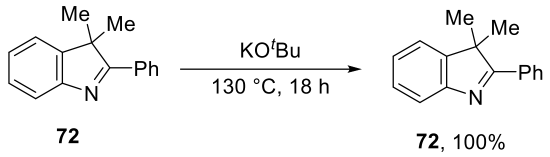

To a pressure tube in the glovebox under nitrogen was added 3,3-dimethyl-2-phenyl-3*H*-indole **72** (111 mg, 0.5 mmol, 1 equiv.) and potassium *tert*-butoxide (168 mg, 1.5 mmol, 3 equiv.). The tube was sealed and removed from the glovebox, and then heated at 130 °C for 18 h behind a shield. After cooling to room temperature, the mixture was diluted with water (50 mL) and extracted into diethyl ether (3 x 50 mL). The organic layers were dried over a hydrophobic frit and concentrated under reduced pressure. No reaction was found to occur, and starting material **72** was recovered, with data consistent with those reported above (111 mg, 100%).

Treatment of 3,3-dimethyl-2-phenyl-3*H*-indole (**72**) with PhMgBr

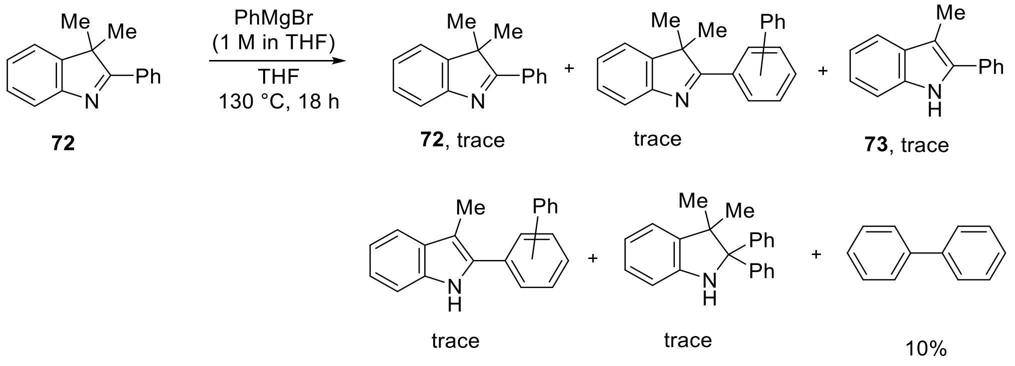

Carried out according to **General Procedure C** using 3,3-dimethyl-2-phenyl-3*H*-indole **72** (1.0 eq., 0.5 mmol, 110.65 mg) and PhMgBr (2.0 eq., 1 mmol, 1 mL) as a 1 M solution in THF and dry THF (500 µL). Purification by column chromatography (hexane → 5% EtOAc/95% hexane) afforded biphenyl (8 mg, 10%) as a white solid (8 mg, 10%). **Mp** = 65-67 °C (lit. mp = 65-67 °C).[25] **^{1}H NMR** (400 MHz, $CDCl_3$) δ 7.65 – 7.57 (m, 2H, 2 x ArH), 7.53 – 7.41 (m, 2H, 2 x ArH), 7.42 – 7.32 (m, 1H, ArH). **^{13}C NMR** (101 MHz, $CDCl_3$) δ 141.4, 128.9, 127.4, 127.3. **ATR-IR ν_{max}** (neat)/cm^{-1} 3061, 3032, 1597, 1570, 1479, 1429, 1344, 1265, 1182, 1169, 1074, 1007, 781, 727, 696, 610. ***m/z* (EI):** 154.0 (M$^+$, 100%), 139.0 (2), 128.0 (6), 115.0 (7), 102.0 (7), 87.0 (4), 76.0 (18), 63.0 (13), 51.0 (21). The data are consistent with those previously reported in the literature[25]

All other fractions contained mixtures of products that were tentatively identified by GC-MS. Trace amounts of the 3,3-dimethyl-2-phenyl-3*H*-indole starting material **72** (RT 13.91 min, *m/z* 221.0), arylated 3,3-dimethyl-2-phenyl-3*H*-indole (RT 16.12 min, *m/z* 297.1), 3-methyl-2-phenyl-1*H*-indole **73** (RT 14.93 min, *m/z* 207.0), arylated 3-methyl-2-phenyl-1*H*-indole (RT 16.69 min, *m/z* 283.0) and 3,3-dimethyl-2,2-diphenylindoline (RT 16.27, *m/z* 299.1) were detected in the crude reaction mixture by GC-MS.

A small sample of PhMgBr (1 M in THF) was quenched with water at 0 °C and a sample of the organic layer was analysed by GC-MS. A small amount of biphenyl was present in the Grignard reagent by GC-MS.

Treatment of 3,3-dimethyl-2-phenyl-3*H*-indole (72) with ^{n}BuMgBr

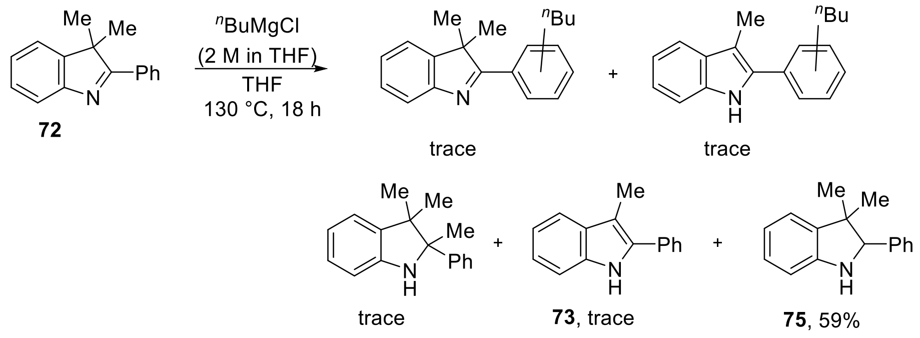

Carried out according to **General Procedure C** using 3,3-dimethyl-2-phenyl-3*H*-indole **72**(1.0 eq., 0.5 mmol, 110.65 mg) and ^{n}BuMgCl (2.0 eq., 1 mmol, 500 µL) as a 2 M solution in THF and dry THF (1 mL). Purification by column chromatography (3% CH_2Cl_2/97% hexane → CH_2Cl_2) afforded 3,3-dimethyl-2-phenylindoline **75** (65 mg, 59%) as a yellow oil. **^{1}H NMR** (400 MHz, $CDCl_3$) δ 7.49 – 7.42 (m, 2H, 2 x ArH), 7.41 – 7.27 (m, 3H, 3 x ArH), 7.13 – 7.03 (m, 2H, 2 x ArH), 6.79 (td, *J* = 7.4, 1.0 Hz, 1H, ArH), 6.72 (d, *J* = 7.7 Hz, 1H, ArH), 4.61 (s, 1H, CH), 4.09 (br s, 1H, NH), 1.44 (s, 3H, Me), 0.74 (s, 3H, Me). **^{13}C NMR** (101 MHz, $CDCl_3$) δ 149.4, 140.1, 138.2, 128.2, 127.6, 127.6, 127.5, 122.6, 119.1, 109.3, 74.7, 45.5, 26.7, 24.6. **ATR-IR ν_{max}** (neat)/cm^{-1} 3364, 3053, 3028, 2961, 2926, 2864, 1607, 1520, 1483, 1452, 1385, 1358, 1315, 1292, 1258, 1240, 1138, 1088, 1030, 1003, 812, 772, 741, 694. ***m/z* (EI):** 223.2 (M$^+$, 53%), 208.2 (100), 193.1 (40), 180.1 (3), 165.1 (5), 146.1 (9), 130.1 (16), 115.1 (7), 103.1 (6), 91.1 (13), 77.1 (15), 65.1 (4), 51.1 (7). The data are consistent with those previously reported in the literature.[1]

Trace amounts of butylated 3,3-dimethyl-2-phenyl-3*H*-indole (RT 14.98 min, *m/z* 277.1), butylated 3-methyl-2-phenyl-1*H*-indole (RT 15.46 min, *m/z* 263.1), 2,3,3-trimethyl-2-phenylindoline (RT 14.22 min, *m/z* 237.2) and 3-methyl-2-phenyl-1*H*-indole **73** (RT 15.02 min, *m/z* 207.0) were detected by GC-MS.

Treatment of 3,3-dimethyl-2-phenyl-3*H*-indole (**72**) with ^{t}BuMgBr

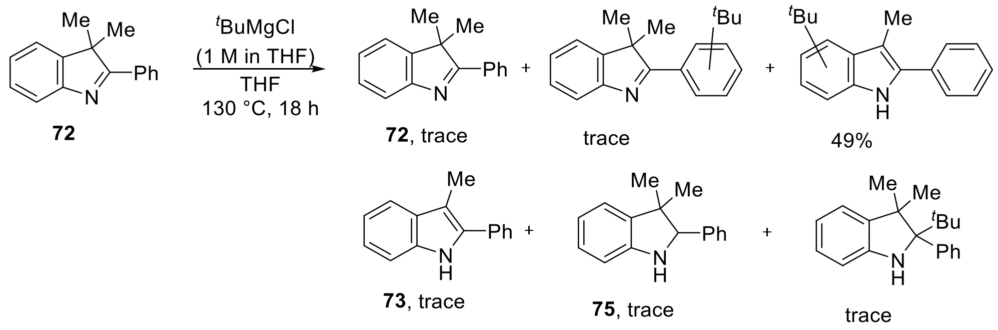

Carried out according to **General Procedure C** using 3,3-dimethyl-2-phenyl-3*H*-indole **72** (1.0 eq., 0.5 mmol, 110.65 mg) and ^{t}BuMgCl (2.0 eq., 1 mmol, 1 mL) as a 1 M solution in THF and dry THF (500 µL). Purification by column chromatography afforded *t*-butylated 3-methyl-2-phenyl-1*H*-indole (65 mg, 49%) as a yellow oil. **^{1}H NMR** (400 MHz, $CDCl_3$) δ 7.99 (br s, 1H, NH), 7.65 (d, *J* = 7.7 Hz, 1H, ArH), 7.64 – 7.49 (m, 4H, 4 x ArH), 7.44 – 7.35 (m, 2H, 2 x ArH), 7.30 – 7.16 (m, 2H, 2 x ArH), 2.51 (s, 3H, Me), 1.43 (s, 9H, ^{t}Bu). **^{13}C NMR** (101 MHz, $CDCl_3$) δ 150.5, 135.9, 134.2, 130.6, 130.2, 127.5, 125.9, 122.2, 119.6, 119.0, 110.7, 108.5, 34.8, 31.5, 9.8. **ATR-IR ν_{max}** (neat)/cm^{-1} 3406, 3055, 3030, 2959, 2903, 2864, 1605, 1510, 1483, 1362, 1335, 1304, 1267, 1242, 1109, 1020, 908, 837, 739, 700. ***m/z* (EI):** 263.1 (M$^+$, 98%), 248.1 (100), 233.1 (22), 217.1 (11), 204.1 (22), 191.1 (4), 178.1 (5), 152.1 (2), 130.1 (19), 109.8 (11), 90.0 (5), 77.0 (12), 57.0 (5).

Trace amounts of the 3,3-dimethyl-2-phenyl-3*H*-indole starting material (RT 13.91 min, *m/z* 221.1), *t*-butylated 3,3-dimethyl-2-phenyl-3*H*-indole starting material (RT 15.59 min, *m/z* 276.1), 3-methyl-2-phenyl-1*H*-indole **73** (RT 15.02 min, *m/z* 207.0), 3,3-dimethyl-2-phenylindoline **75** (RT 13.79 min, *m/z* 223.1) and 2-(*tert*-butyl)-3,3-dimethyl-2-phenylindoline (RT 16.71 min, *m/z* 279.1) were detected in the crude reaction mixture by GC-MS.

Treatment of 3,3-dimethyl-2-phenyl-3*H*-indole (**72**) with BnMgCl

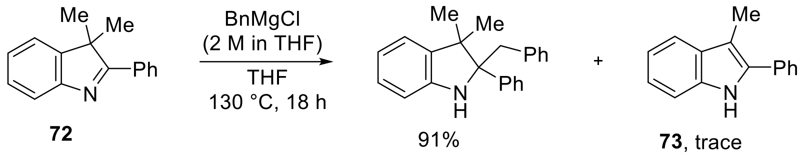

Carried out according to **General Procedure C** using 3,3-dimethyl-2-phenyl-3*H*-indole **72** (1.0 eq., 0.5 mmol, 110.65 mg) and BnMgCl (2.0 eq., 1 mmol, 500 µL) as a 2 M solution in THF and dry THF (1 mL). Purification by column chromatography afforded 2-benzyl-3,3-dimethyl-2-phenylindoline (143 mg, 91%) as a yellow oil. **^{1}H NMR** (400 MHz, $CDCl_3$) δ 7.61 (d, *J* = 6.8 Hz, 2H, 2 x ArH), 7.45 – 7.30 (m, 3H, 3 x ArH), 7.21 – 7.10 (m, 5H, 5 x ArH), 6.88 (td, *J* = 7.4, 0.7 Hz, 1H, ArH), 6.77 (d, *J* = 7.6 Hz, 1H, ArH), 6.68 (dd, *J* = 7.7, 1.5 Hz, 2H, 2 x ArH), 4.03 (br s, 1H, NH), 3.37 (d, *J* = 12.9 Hz, 1H, CH_2), 3.07 (d, *J* = 13.0 Hz, 1H, CH_2), 1.68 (s, 3H, Me), 0.75 (s, 3H, Me). **^{13}C NMR** (101 MHz, $CDCl_3$)

δ 147.7, 141.9, 138.2, 137.5, 130.3, 128.1, 128.0, 127.7, 127.1, 126.9, 126.5, 122.6, 118.9, 109.6, 75.7, 48.8, 40.9, 27.9, 20.1. **ATR-IR ν$_{max}$** (neat)/cm^{-1} 3362, 3082, 3055, 3026, 2961, 2924, 2905, 2860, 1607, 1493, 1483, 1458, 1445, 1387, 1362, 1333, 1319, 1260, 1242, 1209, 1180, 1153, 1111, 1092, 1076, 1053, 1032, 1018, 988, 922, 912, 887, 853, 810, 777, 741, 702, 685, 635, 610. ***m/z* (EI):** 313.2 (M^+, 1%), 270.1 (1), 222.2 (100), 207.1 (100), 191.1 (3), 178.1 (130), 165.1 (5), 153.0 (5), 144.1 (6), 130.0 (18), 115.1 (8), 103.0 (5), 91.1 (55), 77.1 (10), 65.1 (18), 51.1 (4). **HRMS (ESI)** calcd. for $C_{23}H_{24}N^+$ ($[M+H]^+$): 314.1903, found 314.1906.

Trace amounts of 3-methyl-2-phenyl-1*H*-indole **73** (RT 15.02 min, *m/z* 207.0) were also detected in the crude reaction mixture by GC-MS.

Treatment of 2-(4-methoxyphenyl)-3,3-dimethyl-3*H*-indole with Et_3SiH/KO^{t}Bu

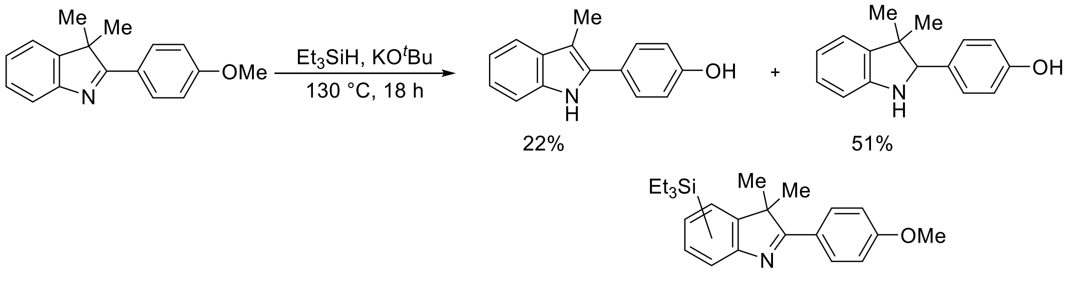

22% 51%

trace

Carried out according to **General Procedure A** using 2-(4-methoxyphenyl)-3,3-dimethyl-3*H*-indole (1.0 eq., 0.5 mmol, 125.67 mg), Et_3SiH (3.0 eq., 1.5 mmol, 240 µL) and KO^{t}Bu (3.0 eq., 1.5 mmol, 168.32 mg). The aqueous layer after the Et_2O (3 x 50 mL) washes was further acidified with 2 M HCl and was further extracted with Et_2O (3 x 50 mL). Purification by column chromatography (100% hexane → 8% EtOAc/92% hexane) afforded 4-(3,3-dimethylindolin-2-yl)phenol (61 mg, 51%) as an yellow oil and a compound that was tentatively identified as 4-(3-methyl-1*H*-indol-2-yl)phenol (25 mg, 22%) as a very unstable fluorescent yellow oil.

4-(3,3-dimethylindolin-2-yl)phenol: **^{1}H NMR** (400 MHz, $CDCl_3$) δ 7.31 (d, *J* = 8.5 Hz, 2H, 2 x ArH), 7.13 – 7.01 (m, 2H, 2 x ArH), 6.86 – 6.75 (m, 2H, 2 x ArH), 6.71 (d, *J* = 7.7 Hz, 1H, ArH), 4.68 (br s, 1H, NH), 4.53 (s, 1H, CH), 1.40 (s, 3H, Me), 0.74 (s, 3H, Me). **^{13}C NMR** (101 MHz, $CDCl_3$) δ 155.2, 149.5, 138.3, 132.2, 128.8, 127.5, 122.7, 119.1, 115.1, 109.3, 74.2, 45.4, 26.6, 24.5. **ATR-IR ν$_{max}$** (neat)/cm^{-1} 3370, 3049, 3028, 2957, 2922, 2864, 1609, 1512, 1483, 1458, 1387, 1360, 1317, 1237, 1167, 1138, 1111, 1086, 1045, 1016, 908, 841, 820 789, 746, 733, 689, 638, 613. ***m/z* (EI):** 237.1 ($[M-2]^+$, 100%), 221.8 (52), 191.0 (6), 180.9 (9), 167.0 (4), 144.1 (35), 129.8 (9), 118.9 (93), 103.0 (35), 91.0 (84), 77.0 (53), 64.1 (43), 50.9 (62). ***m/z* (EI):** 239.1 (M^+, 16%), 223.8 (100), 175.7 (3), 158.3 (4), 129.7 (19), 117.0 (40), 106.8 (26), 90.9 (40), 76.8 (48), 65.0 (15), 50.9 (14). **HRMS (ESI)** calcd. for $C_{16}H_{16}NO^-$ ($[M-H]^-$): 238.1237, found 238.1230.

4-(3-methyl-1*H*-indol-2-yl)phenol: **^{1}H NMR** (400 MHz, $CDCl_3$) δ 8.03 (br s, 1H, NH), 7.63 – 7.56 (m, 2H, 2 x ArH), 7.54 – 7.42 (m, 2H, 2 x ArH), 7.41 – 7.31 (m, 2H, 2 x ArH), 7.24 – 7.09 (m, 2H, 2 x ArH), 2.47 (s, 3H, Me). ***m/z* (EI):** 223 (M^+, 33%), 207.8 (3), 194.0 (100), 180.1 (7), 165.0 (3), 151.9 (19), 127.1 (4), 117.0 (22), 104.0 (47), 92.0 (36), 77.0 (100), 65.0 (32), 51.0 (86). No further data was obtained due to the quick decomposition of the compound.

Trace amounts of the silylated 2-(4-methoxyphenyl)-3,3-dimethyl-3*H*-indole (RT 17.29 min, *m/z* 365.3) were detected in the crude reaction mixture by GC-MS.

Treatment of 2-(4-fluorophenyl)-3,3-dimethyl-3*H*-indole with Et_3SiH/KO^{t}Bu

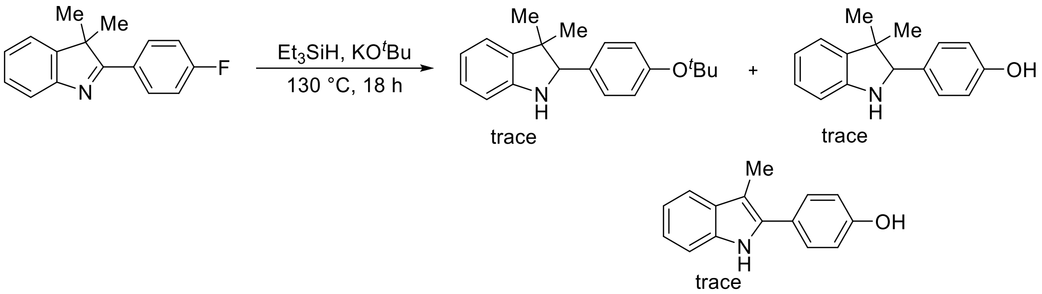

Carried out according to **General Procedure A** using 2-(4-fluorophenyl)-3,3-dimethyl-3*H*-indole (1.0 eq., 0.5 mmol, 119.65 mg), Et_3SiH (3.0 eq., 1.5 mmol, 240 µL) and KO^{t}Bu (3.0 eq., 1.5 mmol, 168.32 mg). The crude mixture was purified by column chromatography (100% hexane → 8% EtOAc/92% hexane) but no clean products were isolated. Trace amounts of 2-(4-(*tert*-butoxy)phenyl)-3,3-dimethylindoline (RT 16.26 min, *m/z* 295.2), 4-(3,3-dimethylindolin-2-yl)phenol (RT 15.93 min, *m/z* 239.1), 4-(3-methyl-1*H*-indol-2-yl)phenol (RT 16.99 min, m/z 223.0) were detected in the crude reaction mixture by GC-MS.

Treatment of 3,3-dimethyl-2-(4-(trifluoromethyl)phenyl)-3*H*-indole with Et_3SiH/KO^{t}Bu

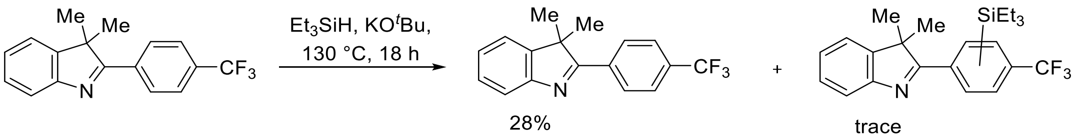

Carried out according to **General Procedure A** using 3,3-dimethyl-2-(4-(trifluoromethyl)phenyl)-3*H*-indole (1.0 eq., 0.5 mmol, 144.65 mg), Et_3SiH (3.0 eq., 1.5 mmol, 240 µL) and KO^{t}Bu (3.0 eq., 1.5 mmol, 168.32 mg). Purification by column chromatography (100% hexane → 1% EtOAc/99% hexane) afforded the 3,3-dimethyl-2-(4-(trifluoromethyl)phenyl)-3*H*-indole starting material (40 mg, 28%) as a yellow solid. **Mp** = 97-100 °C (no mp reported in the lit.). **^{1}H NMR** (400 MHz, $CDCl_3$) δ 8.26 (d, *J* = 8.2 Hz, 2H, 2 x ArH), 7.74 (t, *J* = 7.9 Hz, 3H, 3 x ArH), 7.44 – 7.28 (m, 3H, 3 x ArH), 1.60 (s, 6H, 2 x Me). **^{13}C NMR** (101 MHz, $CDCl_3$) δ 181.9, 152.9, 147.7, 136.7, 132.1 (q, *J* = 32.8 Hz), 128.7, 128.1, 126.7, 125.7 (q, *J* = 3.3 Hz), 124.1 (q, *J* = 271.9 Hz), 121.5, 121.2, 53.8, 24.6. **ATR-IR ν_{max}** (neat)/cm^{-1} 3073, 2969, 2930, 2870, 1618, 1518, 1472, 1454, 1408, 1321, 1308, 1165, 1125, 1111, 1090, 1067,

1013, 993, 934, 845, 826, 789, 772, 746, 698, 683. ***m/z* (EI):** 289.1 (M^+, 100%), 274.1 (56), 254.1 (1), 233.1 (5), 218.1 (2), 204.1 (10), 179.1 (8), 144.1 (19), 130.6 (13), 117.1 (37), 103.1 (29), 91.0 (18), 77.1 (31), 63.1 (6). 51.1 (11). The data are consistent with those previously reported in the literature.[1]

Trace amounts of silylated 3,3-dimethyl-2-(4-(trifluoromethyl)phenyl)-3*H*-indole starting material (RT 16.11 min, *m/z* 403.1) were detected by GC-MS.

Treatment of 3,3-dimethyl-2-(*p*-tolyl)-3*H*-indole with Et_3SiH/KO^{t}Bu

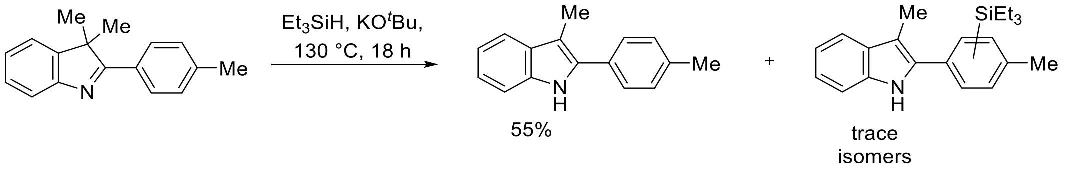

Carried out according to **General Procedure A** using 3,3-dimethyl-2-(*p*-tolyl)-3*H*-indole (1.0 eq., 0.5 mmol, 110.65 mg), Et_3SiH (3.0 eq., 1.5 mmol, 240 µL) and KO^{t}Bu (3.0 eq., 1.5 mmol, 168.32 mg). Purification by column chromatography (hexane → 0.5% EtOAc/99.5% hexane) followed by another purification by column chromatography (hexane → 1% Et_2O/99% hexane) afforded 3-methyl-2-(*p*-tolyl)-1*H*-indole (61 mg, 55%) as a white solid. **Mp =** 108-111 °C (lit. mp. 110-111 °C).[26] **^{1}H NMR** (400 MHz, $CDCl_3$) δ 7.97 (br s, 1H, NH), 7.61 (d, *J* = 7.8 Hz, 1H, ArH), 7.49 (d, *J* = 8.1 Hz, 2H, 2 x ArH), 7.37 (d, *J* = 7.9 Hz, 1H, ArH), 7.30 (d, *J* = 7.9 Hz, 2H, 2 x ArH), 7.25 – 7.11 (m, 2H, 2 x ArH), 2.47 (s, 3H, Me), 2.43 (s, 3H, Me). **^{13}C NMR** (101 MHz, $CDCl_3$) δ 137.3, 135.9, 134.3, 130.6, 130.2, 129.7, 127.8, 122.3, 119.6, 119.0, 110.7, 108.4, 21.4, 9.8. **ATR-IR ν_{max}** (neat)/cm^{-1} 3377, 3053, 3024, 2913, 2859, 1508, 1485, 1458, 1437, 1369, 1331, 1314, 1302, 1244, 1237, 1186, 1157, 1113, 1032, 1018, 997, 845, 824, 812, 756, 741, 727, 681, 627. ***m/z* (EI):** 221.1 (M^+, 100%), 204.1 (27), 191.1 (2), 178.1 (4), 167.1 (1), 152.1 (2), 139.0 (1), 130.1 (19), 117.1 (2), 109.6 (6), 102.1 (9), 89.1 (3), 77.1 (6), 63.0 (2), 51.1 (2). The data are consistent with those previously reported in the literature.[26]

Trace amounts of isomers of silylated 3-methyl-2-(*p*-tolyl)-1*H*-indole (RT 18.53 min, 18.65 min, 18.87 min (major isomer), *m/z* 335.2) were present in the crude reaction mixture by GC-MS.

Treatment of 2,3,3-triphenyl-3*H*-indole (**81**) with Et_3SiH/KO^{t}Bu

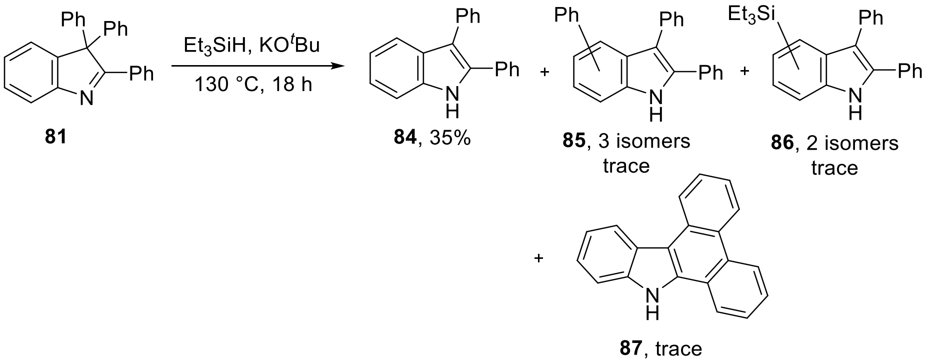

Carried out according to **General Procedure A** using 2,3,3-triphenyl-3*H*-indole **81** (1.0 eq., 0.5 mmol, 172.73 mg), Et_3SiH (3.0 eq., 1.5 mmol, 240 µL) and KO^tBu (3.0 eq., 1.5 mmol, 168.32 mg). Two purifications by column chromatography (hexane → 10% Et_2O/90% hexane, hexane → 1.5% EtOAc/98.5% hexane) afforded 2,3-diphenyl-1*H*-indole **84** (47 mg, 35%) as a yellow oil. **^{1}H NMR** (400 MHz, $CDCl_3$) δ 8.13 (br s, 1H, NH), 7.69 (d, *J* = 7.8 Hz, 1H, ArH), 7.45 – 7.44 (m, 2H, 2 x ArH), 7.42 – 7.34 (m, 5H, 5 x ArH), 7.32 – 7.20 (m, 5H, 5 x ArH), 7.19 – 7.11 (m, 1H, ArH). **^{13}C NMR** (101 MHz, $CDCl_3$) δ 136.0, 135.2, 134.2, 132.8, 130.3, 128.9, 128.8, 128.7, 128.3, 127.8, 126.4, 122.8, 120.6, 119.8, 115.2, 111.0. **ATR-IR ν_{max}** (neat)/cm^{-1} 3406, 3055, 3026, 2951, 2924, 2855, 1601, 1553, 1504, 1481, 1454, 1439, 1423, 1329, 1304, 1250, 1152, 1115, 1070, 1028, 1011, 984, 964, 908, 764, 743, 696, 608. ***m/z* (EI)**: 269.1 (M^+, 100%), 254.0 (7), 239.0 (8), 213.0 (3), 190.0 (5), 165.0 (26), 133.5 (17), 120.5 (7), 113.0 (5), 103.0 (3), 89.0 (4), 77.0 (10), 63.0 (9), 51.0 (14). The data for this compound are consistent with those previously reported in the literature.[27]

Trace amounts of three isomers of arylated 2,3-diphenyl-1*H*-indole **85** (RT 18.81, 19.721, 21.16 min, *m/z* 345.3) and two isomers of silylated 2,3-diphenyl-1*H*-indole **86** (RT 19.08 and 20.04 min, *m/z* 383.3 and 383.4) were detected by GC-MS. Trace amounts of 9*H*-dibenzo[a,c]carbazole **87** (RT 20.12 min, *m/z* 267.2) was detected by GC-MS and ^{1}H NMR with ^{1}H NMR data consistent with those previously reported in the literature.[28]

Treatment of 3,3-diallyl-2-phenyl-3*H*-indole (**82**) with Et_3SiH/KO^tBu

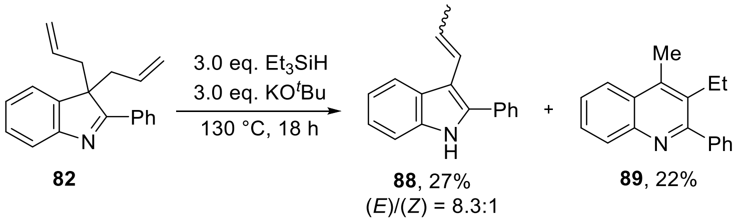

Carried out according to **General Procedure A** using 3,3-diallyl-2-phenyl-3*H*-indole (1.0 eq., 0.5 mmol, 137 mg), Et_3SiH (3.0 eq., 1.5 mmol, 240 µL) and KO^tBu (3.0 eq., 1.5 mmol, 168 mg). Purification by column chromatography (hexane → 3% EtOAc/97% hexane) afforded 2-a mixture of phenyl-3-(prop-1-en-1-yl)-1*H*-indole (*E*)/(*Z*)=8.3:1 (31 mg, 27%) as a yellow oil and 3-ethyl-4-methyl-2-phenylquinoline (27 mg, 22%) as a yellow oil.

(*E*)/(*Z*)-Phenyl-3-(prop-1-en-1-yl)-1*H*-indole: **^{1}H NMR** (400 MHz, $CDCl_3$) δ 8.23 (br s, 1H, (*Z*)-NH), 8.07 (br s, 1H, (*E*)-NH), 7.93 (d, *J* = 7.8 Hz, 1H, (*E*)-ArH), 7.68 (dd, *J* = 8.3, 1.1 Hz, 2H, 2 x (*Z*)-ArH), 7.60 (dd, *J* = 8.2, 1.1 Hz, 2H, 2 x (*E*)-ArH), 7.53 – 7.46 (m, 3H, (*Z*)-ArH, 2 x (*E*)-ArH), 7.45 – 7.42 (m, 2H, 2 x (*Z*)-ArH), 7.42 – 7.35 (m, 2H, 2 x (*E*)-ArH), 7.36 – 7.30 (m, 2H, 2 x (*Z*)-ArH), 7.21 (tdd, *J* = 14.8, 7.3, 1.2 Hz, 4H, 2 x (*Z*)-ArH, 2 x (*E*)-ArH), 6.65 (dq, *J* = 15.9, 1.6 Hz, 1H, (*E*)-alkene *CH*Ar), 6.60 – 6.52 (m, 1H, (*Z*)-alkene *CH*Ar), 6.35 (dq, *J* = 16.0, 6.5 Hz, 1H, (*E*)-alkene *CH*CH_3), 5.90 (dq, *J* = 11.1, 6.9 Hz, 1H, (*Z*)-alkene *CH*CH_3), 1.95 (dd, *J* = 6.5, 1.6 Hz, 3H, (*E*)-CH_3), 1.62 (dd, *J* = 6.9, 1.7

Hz, 3H, (*Z*)-CH_3). Due to the instability of the compounds in this mixture (their tendency to polymerise), it was not possible to obtain further characterisation data.

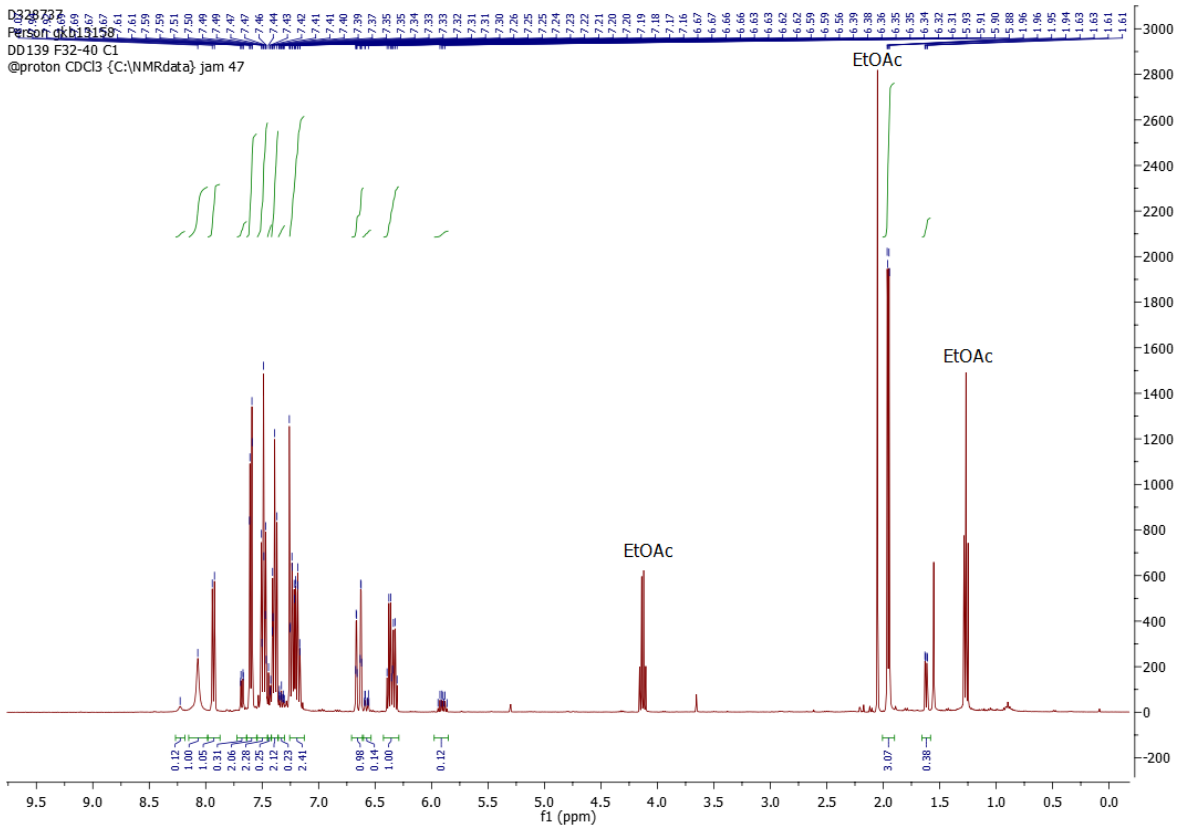

Figure S1. ^{1}H NMR data evidance for the formation of **88**

3-Ethyl-4-methyl-2-phenylquinoline: **^{1}H NMR** (400 MHz, $CDCl_3$) δ 8.12 (dd, *J* = 8.3, 0.5 Hz, 1H, ArH), 8.04 (dd, *J* = 8.4, 0.7 Hz, 1H, ArH), 7.66 (td, *J* = 8.3, 1.3 Hz, 1H, ArH), 7.55 (td, *J* = 8.2, 1.2 Hz, 1H, ArH), 7.53 – 7.38 (m, 5H, 5 x ArH), 2.81 (q, *J* = 7.5 Hz, 2H, CH_2), 2.74 (s, 3H, Ar*CH_3*), 1.08 (t, *J* = 7.5 Hz, 3H, CH_2*CH_3*). **^{13}C NMR** (101 MHz, $CDCl_3$) δ 161.0, 145.9, 142.2, 141.7, 133.5, 130.2, 128.6, 128.5, 128.3, 127.9, 127.8, 126.3, 123.6, 23.3, 14.9, 14.4. **ATR-IR ν_{max}** (neat)/cm^{-1} 3057, 3026, 2967, 2928, 2870, 1574, 1558, 1493, 1452, 1441, 1400, 1373, 1354, 1346, 1321, 1271, 1258, 1233, 1184, 1146, 1136, 1098, 1072, 1057, 1043, 1024, 1001, 964, 908, 862, 799, 754, 727, 700, 677, 665. ***m/z* (EI)**: 247.2 (M^+, 39%), 246.2 (100), 231.1 (24), 217.1 (10), 204.1 (3), 189.1 (1), 167.1 (2), 140.1 (1), 128.1 (5), 115.2 (8), 108.6 (4), 103.1 (4), 89.0 (3), 77.1 (7), 63.1 (3), 51.1 (5). **HRMS (ESI)** calcd. for $C_{18}H_{18}N^+$ ($[M+H]^+$): 248.1434, found 248.1434.

Treatment of 3,3-dibenzyl-2-phenyl-3*H*-indole (**83**) with Et_3SiH/KO^{t}Bu

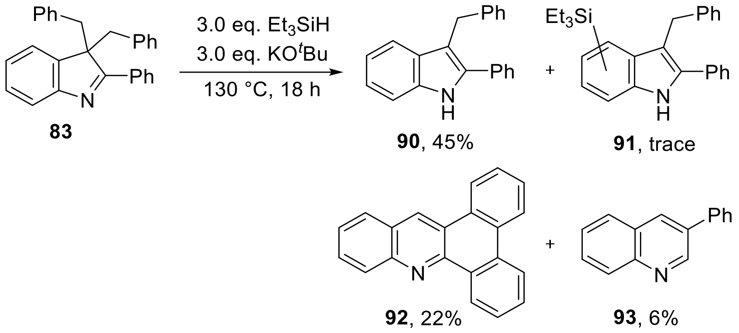

Carried out according to **General Procedure A** using 3,3-dibenzyl-2-phenyl-3*H*-indole **83** (1.0 eq., 0.5 mmol, 186.75 mg), Et_3SiH (3.0 eq., 1.5 mmol, 240 µL) and KO^{t}Bu (3.0 eq., 1.5 mmol, 168.32 mg). Purifications by column chromatography (hexane → 5% EtOAc/95% hexane) afforded 3-benzyl-2-phenyl-1*H*-indole **90** (64 mg, 45%) as a bright yellow oil. The fractions containing the slightly impure dibenzo[a,c]acridine **92** were purified by recrystallisation from hexane/EtOAc affording pure dibenzo[a,c]acridine **92** as a bright yellow solid (31 mg, 22%). The fractions containing the impure 3-phenylquinoline **93** were purified by preparative TLC (35% EtOAc/65% hexane) affording pure 3-phenylquinoline **93** as a pale brown oil (6 mg, 6%).

3-Benzyl-2-phenyl-1*H*-indole: **^{1}H NMR** (400 MHz, $CDCl_3$) δ 8.13 (br s, 1H, NH), 7.57 – 7.50 (m, 2H, 2 x ArH), 7.48 – 7.39 (m, 4H, 4 x ArH), 7.39 – 7.32 (m, 1H, ArH), 7.30 – 7.17 (m, 6H, 6 x ArH), 7.08 (t, J = 8.0 Hz, 1H, ArH), 4.29 (s, 2H, CH_2). **^{13}C NMR** (101 MHz, $CDCl_3$) δ 141.6, 136.2, 135.6, 133.1, 129.7, 129.0, 128.5, 128.4, 128.0, 127.9, 125.9, 122.5, 119.9, 119.8, 111.3, 110.9, 30.6. **ATR-IR ν_{max}** (neat)/cm^{-1} 3410, 3057, 3024, 2955, 2928, 2870, 1601, 1518, 1491, 1452, 1425, 1341, 1304, 1244, 1177, 1153, 1144, 1113, 1096, 1074, 1043, 1007, 991, 955, 914, 847, 808, 764, 741, 725, 696, 669, 627, 608. ***m/z* (EI)**: 283.3 (M$^+$, 83%), 206.2 (100), 191.1 (3), 178.2 (20), 165.2 (4), 152.1 (4), 141.7 (4), 128.1 (8), 102.4 (5), 91.2 (3), 77.2 (23), 65.1 (3), 51.0 (10). The data for this compound are consistent with those previously reported in the literature.[29]

Dibenzo[a,c]acridine: **Mp** = 203-205 °C (decomp.) (lit. mp = 204-205 °C).[30] **^{1}H NMR** (400 MHz, $CDCl_3$) δ 9.57 – 9.45 (m, 1H, ArH), 9.20 (s, 1H, ArH), 8.58 – 8.56 (m, 1H, ArH), 8.54 – 8.44 (m, 2H, 2 x ArH), 8.34 (d, J = 8.6 Hz, 1H, ArH), 8.00 (d, J = 8.1 Hz, 1H, ArH), 7.80 (ddd, J = 8.4, 6.8, 1.3 Hz, 1H, ArH), 7.75 – 7.70 (m, 2H, 2 x ArH), 7.66 – 7.54 (m, 3H, 3 x ArH). **^{13}C NMR** (101 MHz, $CDCl_3$) δ 147.0, 146.7, 132.2, 130.9, 130.3, 130.0, 129.7, 128.7, 128.3, 128.1, 127.9, 127.7, 127.0, 126.4, 126.4, 123.6, 123.5, 123.4, 122.8 (two overlapping signals). **ATR-IR ν_{max}** (neat)/cm^{-1} 3067, 3049, 1597, 1543, 1504, 1491, 1472, 1458, 1445, 1410, 1381, 1342, 1323, 1306, 1261, 1236, 1229, 1202, 1171, 1146, 1129, 1051, 1036, 1007, 995, 949, 905, 876, 854, 800, 758, 718, 700, 683, 668, 654, 633, 608. ***m/z* (EI)**: 279.3 (M$^+$, 100%), 251.3 (3), 224.2 (2), 139.3 (15), 125.2 (3), 112.2. (2), 74.2 (1), 63.1 (2), 51.2 (2). The data for this compound are consistent with those previously reported in the literature.[30]

3-Phenylquinoline: **^{1}H NMR** (400 MHz, $CDCl_3$) δ 9.19 (d, *J* = 2.2 Hz, 1H, ArH), 8.31 (d, *J* = 2.0 Hz, 1H, ArH), 8.15 (d, *J* = 8.4 Hz, 1H, ArH), 7.89 (dd, *J* = 8.2, 1.2 Hz, 1H, ArH), 7.76 – 7.70 (m, 3H, 3 x ArH), 7.62 – 7.51 (m, 3H, 3 x ArH), 7.45 (ddd, *J* = 7.4, 3.9, 1.2 Hz, 1H, ArH). **^{13}C NMR** (101 MHz, $CDCl_3$) δ 150.1, 147.5, 138.1, 134.0, 133.4, 129.5, 129.4, 129.3, 128.3, 128.2, 128.2, 127.6, 127.2. **ATR-IR ν_{max}** (neat)/cm^{-1} 3057, 3030, 2922, 2853, 1597, 1491, 1449, 1412, 1364, 1341, 1290, 1261, 1227, 1182, 1159, 1142, 1125, 1076, 1043, 1026, 953, 920, 903, 862, 787, 762, 750, 696, 669, 610. ***m/z* (EI)**: 205.3 (M^+, 100%), 176.2 (20), 151.2 (9), 139.2 (3), 126.3 (3), 113.2 (1), 102.3 (10), 88.2 (6), 76.2 (14), 63.1 (7), 51.1 (9). The data for this compound are consistent with those previously reported in the literature.[30]

Trace amounts of silylated 3-benzyl-2-phenyl-1*H*-indole **91** were detected by GC-MS (RT 19.83 min, *m/z* 397.5).

Mechanistic studies

Preparation of potassium 4,4'-di-*tert*-butyl-1,1'-biphenyl

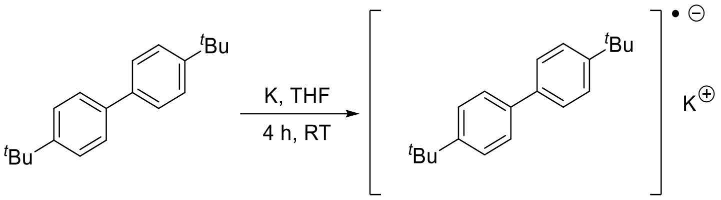

KDTBB

This reaction was carried out according to a literature procedure.[31] Potassium metal was washed with hexane under argon atmosphere to remove mineral oil. The hexane was removed by needle and syringe and quenched with isopropanol, and the potassium metal was then transferred to the glovebox where the residual hexane was removed under vacuum. The oxide layer was removed from potassium in the glovebox by scraping with a knife, exposing a fresh metallic surface. Potassium metal (177 mg, 4.5 mmol, 1 equiv.) was then added to a solution of 4,4'-di-*tert*- butylbiphenyl (1.2 g, 4.5 mmol, 1 equiv.) in THF (180 mL). The resulting solution was stirred under nitrogen until an intense green colour formed (~4 h). THF was then evaporated under vacuum in the glovebox, affording a green/white solid (KDTBB) which was used immediately without further purification.

Treatment of 2-(2-methoxyphenyl)-2-propylpentanenitrile (**23**) with potassium 4,4'-di-*tert*-butyl-1,1'-biphenyl (KDTBB), $Me_3SiSiMe_3$ and KO^{t}Bu

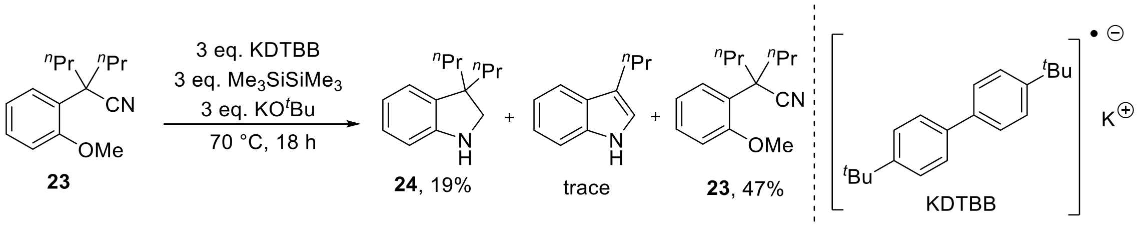

KDTBB was prepared as described above. To a pressure tube in the glovebox under nitrogen was added 2-(2-methoxyphenyl)-2-propylpentanenitrile **23** (116 mg, 0.5 mmol, 1 equiv.), potassium di-*tert*-butylbiphenylide (KDTBB) (458 mg, 1.5 mmol, 3 equiv.), hexamethyldisilane (0.31 mL, 1.5 mmol, 3 equiv.) and potassium *tert*-butoxide (168 mg. 1.5 mmol, 3 equiv.). The tube was sealed, removed from the glovebox and heated at 70 °C for 18 h behind a shield. After cooling to room temperature, the mixture was diluted with water (50 mL) and extracted into diethyl ether (3 x 50 mL). The combined organic layers were dried over Na_2SO_4, filtered and concentrated under reduced pressure. The crude mixture was purified by column chromatography eluting with hexane:ethyl acetate (100:0 → 9:1) to afford starting material **23** as a colourless oil (55 mg, 47%), with data consistent with those reported above, 3,3-dipropylindoline **24** as a colourless oil (19 mg, 19%), with data consistent with those reported above, and 3-propyl-1*H*-indole (<1 mg), with data consistent with those reported above.

Treatment of 2-(2-methoxyphenyl)-2-propylpentanenitrile (23) with potassium 4,4'-di-*tert*-butyl-1,1'-biphenyl (KDTBB) and KO^{t}Bu

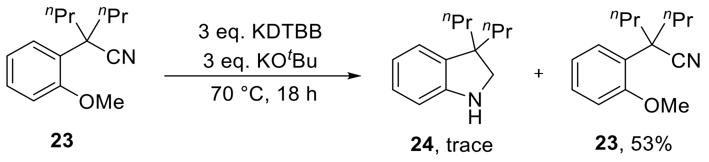

To a pressure tube in the glovebox under nitrogen was added 2-(2-methoxyphenyl)-2-propylpentanenitrile **23** (116 mg, 0.5 mmol, 1 equiv.), KDTBB (458 mg, 1.5 mmol, 3 equiv.), and potassium *tert*-butoxide (168 mg. 1.5 mmol, 3 equiv.). The tube was sealed, removed from the glovebox and heated at 70 °C for 18 h behind a shield. After cooling to room temperature, the mixture was diluted with water (50 mL) and extracted into diethyl ether (3 x 50 mL). The combined organic layers were dried over Na_2SO_4, filtered and concentrated under reduced pressure. The crude mixture was purified by column chromatography eluting with hexane:ethyl acetate (100:0 → 9:1) to afford starting material **23** as a colourless oil (61 mg, 53%), with data consistent with those reported above, and 3,3-dipropylindoline **24** as a colourless oil (<1 mg), with data consistent with those reported above.

Treatment of 2-(2-methoxyphenyl)-2-propylpentanenitrile (23) with $Me_3SiSiMe_3$ and KO^{t}Bu

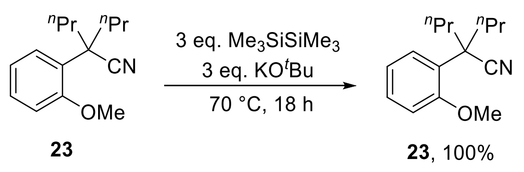

To a pressure tube in the glovebox under nitrogen was added 2-(2-methoxyphenyl)-2-propylpentanenitrile **23** (116 mg, 0.5 mmol, 1 equiv.), hexamethyldisilane (0.31 mL, 1.5 mmol, 3 equiv.), and potassium *tert*-butoxide (168 mg. 1.5 mmol, 3 equiv.). The tube was sealed, removed from the glovebox and heated at 70 °C for 18 h behind a shield. After cooling to room temperature, the mixture was diluted with water (50 mL) and extracted into diethyl ether (3 x 50 mL). The combined

organic layers were dried over Na_2SO_4, filtered and concentrated under reduced pressure. No reaction was found to have taken place, and 2-(2-methoxyphenyl)-2-propylpentanenitrile **23** was recovered with data consistent with those reported above (116 mg, 100%).

Treatment of 2-(2-methoxyphenyl)-2-propylpentanenitrile (**23**) with KO*t*Bu

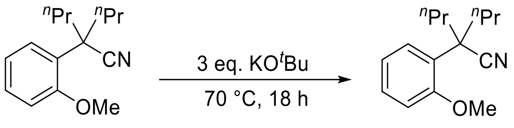

23   **23**, 90%

To a pressure tube in the glovebox under nitrogen was added 2-(2-methoxyphenyl)-2-propylpentanenitrile **23** (116 mg, 0.5 mmol, 1 equiv.), and potassium *tert*-butoxide (168 mg. 1.5 mmol, 3 equiv.). The tube was sealed, removed from the glovebox and heated at 70 °C for 18 h behind a shield. After cooling to room temperature, the mixture was diluted with water (50 mL) and extracted into diethyl ether (3 x 50 mL). The combined organic layers were dried over Na_2SO_4, filtered and concentrated under reduced pressure. No reaction was found to have taken place, and 2-(2-methoxyphenyl)-2-propylpentanenitrile **23** was recovered with data consistent with those reported above (104 mg, 90%).

Treatment of 2-(2-methoxyphenyl)-2-propylpentanenitrile (**23**) with $LiAlH_4$ at 70 °C

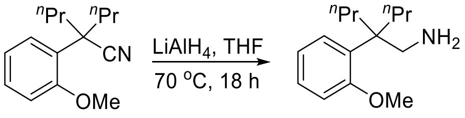

23   74%

To a pressure tube in the glovebox, under nitrogen, was added 2-(2-methoxyphenyl)-2-propylpentanenitrile **23** (116 mg, 0.50 mmol, 1.0 equiv.) and lithium aluminium hydride (2 M in THF, 0.75 mL, 1.50 mmol, 3.0 equiv.) and THF (0.25 mL). The tube was sealed, removed from the glovebox and stirred at 70 °C for 18 h. After cooling to room temperature, the reaction mixture was quenched by addition of water:THF (1:4, 50 mL) then 2 M NaOH (50 mL). The products were extracted into diethyl ether (3 x 50 mL) and the combined organic layers were dried over a hydrophobic frit and concentrated under reduced pressure. Purification by column chromatography (cyclohexane:ethyl acetate, 100:0 → 9:1) afforded 2-(2-methoxyphenyl)-2-propylpentan-1-amine as a colourless oil (87 mg, 74%). **^{1}H NMR** (400 MHz, $CDCl_3$) 0.88 (t, J = 6.9 Hz, 6 H, 2 x CH_3), 1.01 - 1.20 (br m, 6 H, 2 x CH_2 + NH_2), 1.69 (ddd, J = 13.8, 11.8, 5.4 Hz, 2 H, CH_2), 1.82 (ddd, J = 13.8, 11.8, 4.9 Hz, 2 H, CH_2), 3.05 (s, 2 H, CH_2N), 3.82 (s, 3 H, OCH_3), 6.88 (dd, J = 8.0, 1.0 Hz, 1 H, ArH), 6.92 (app. td, J = 7.4, 1.0 Hz, 1 H, ArH), 7.15 - 7.24 (m, 2 H, 2 x ArH). **^{13}C NMR** (101 MHz, $CDCl_3$) 15.0, 17.3, 36.1, 46.5, 46.6, 55.1, 111.6, 120.3, 127.2, 129.4, 133.0, 158.5. **ATR-IR ν_{max}** (neat)/cm^{-1} 2954, 2869, 1578, 1488, 1455, 1289, 1236, 1180, 1095, 1027, 746, 470. **HRMS (CI)** calcd. for $C_{15}H_{26}NO^+$ ($[M+H]^+$): 236.2014, found: 236.2016.

Treatment of 2-(2-methoxyphenyl)-2-propylpentanenitrile (**23**) with $LiAlH_4$ at 130 °C

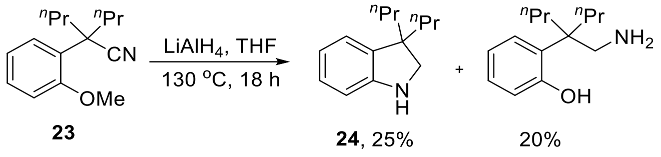

To a pressure tube in the glovebox, under nitrogen, was added 2-(2-methoxyphenyl)-2-propylpentanenitrile **23** (116 mg, 0.50 mmol, 1.0 equiv.) and lithium aluminium hydride (28.0 mg, 1.50 mmol, 3.0 equiv.) and THF (1 mL). The tube was sealed, removed from the glovebox and stirred at 130 °C for 18 h. After cooling to room temperature, the reaction mixture was quenched by addition of water (50 mL) then 2 M NaOH (50 mL). The products were extracted into diethyl ether (3 x 50 mL) and the combined organic layers were dried over a hydrophobic frit and concentrated under reduced pressure. Purification by column chromatography (hexane:ethyl acetate, 100:0 → 1:9) afforded 3,3-dipropylindoline **23** as a yellow oil (25 mg, 25%), with data consistent with those reported above, and 2-(4-(aminomethyl)heptan-4-yl)phenol as a white solid (22 mg, 20%). **Mp** = 90-91 °C. **^{1}H NMR** (400 MHz, $CDCl_3$) 0.92 (t, *J* = 7.4 Hz, 6 H, 2 x CH_3), 1.06 - 1.21 (m, 2 H, 2 x CH), 1.24 - 1.38 (m, 2 H, 2 x CH), 1.62 - 1.73 (m, 2 H, 2 x CH), 1.81 (ddd, *J* = 13.6, 12.4, 4.8 Hz, 2 H, 2 x CH), 3.05 (s, 2 H, NCH_2), 6.72 - 6.80 (m, 1 H, ArH), 6.87 (dd, *J* = 7.9, 1.3 Hz, 1 H, ArH), 7.04 - 7.13 (m, 2 H, 2 x ArH). **^{13}C NMR** (101 MHz, $CDCl_3$) 14.8, 17.1, 38.5, 45.3, 49.1, 118.2, 119.1, 127.6, 128.5, 130.6, 157.5. **ATR-IR ν_{max}** (neat)/cm^{-1} 3371, 2954, 2868, 1620, 1581, 1463, 1440, 1425, 1280, 1224, 904, 839, 748. **HRMS (CI)** calcd. for $C_{14}H_{24}NO^+$ ($[M+H]^+$): 222.1852, found: 222.1853.

Treatment of 2'-phenylspiro[cyclohexane-1,3'-indole] (**76**) with Et_3SiH/KO^{t}Bu

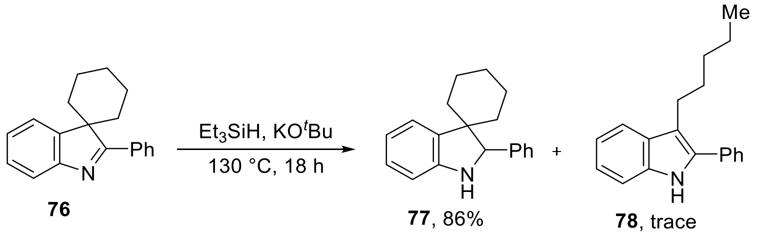

Carried out according to **General Procedure A** using 2'-phenylspiro[cyclohexane-1,3'-indole] **76** (1.0 eq., 0.5 mmol, 130.685 mg), Et_3SiH (3.0 eq., 1.5 mmol, 240 µL) and KO^{t}Bu (3.0 eq., 1.5 mmol, 168.32 mg). Purification by column chromatography (100% hexane → 2% Et_2O/98% hexane) afforded 2'-phenylspiro [cyclohexane-1,3'-indoline] **77** (113 mg, 86%) as a viscous yellow oil. **^{1}H NMR** (400 MHz, $CDCl_3$) δ 7.42 – 7.19 (m, 6H, 6 x ArH), 7.10 (t, *J* = 7.6 Hz, 1H, ArH), 6.79 (t, *J* = 7.4 Hz, 1H, ArH), 6.70 (d, *J* = 7.7 Hz, 1H, ArH), 4.59 (s, 1H, CH), 4.13 (br s, 1H, NH), 1.91 – 1.81 (m, 2H, Cy), 1.77 – 1.67 (m, 2H, Cy), 1.65 – 1.54 (m, 1H, Cy), 1.53 – 1.36 (m, 3H, Cy), 1.29 – 1.08 (m, 2H, Cy). **^{13}C NMR** (101 MHz, $CDCl_3$) δ 150.2, 141.4, 137.2, 128.2, 128.0, 127.7, 127.6, 124.4, 118.6, 108.8, 73.1, 49.3, 37.4, 31.9, 25.9, 23.1, 22.3. **ATR-IR** ν_{max} 3368, 3053, 3026, 2926, 2849, 1605, 1481, 1462, 1452, 1395, 1364, 1350, 1317, 1300, 1252, 1240, 1169, 1028, 957, 914, 881, 847, 802, 768, 739, 700, 685, 660,

625, 615. ***m/z* (EI):** 261.1 ([M-2]$^+$, 100%), 246.1 (2), 232.1 (50), 218.1 (21), 204.1 (49), 184.1 (26), 170.1 (9), 156.1 (16), 131.0 (33), 115.0 (43), 91.0 (21), 77.0 (34), 56.0 (19), 51.0 (16). ***m/z* (ESI+APCI)** 264 ([M+H]$^+$). The data are consistent with those previously reported in the literature[1]

At RT 15.69 min, *m/z* (EI): 263.0 was detected in the GC-MS of the crude reaction mixture, which can be equally attributed to the presence of any of the two products as they have the same *m/z* of 263.2. Trace amounts of 3-pentyl-2-phenyl-1*H*-indole **78** were detected in some column fractions by ^{1}H NMR (Broad NH peak at ~ 7.99 ppm integrating to 1H unit and deshielded CH_2 triplet at ~ 2.88 ppm integrating to 2H units.

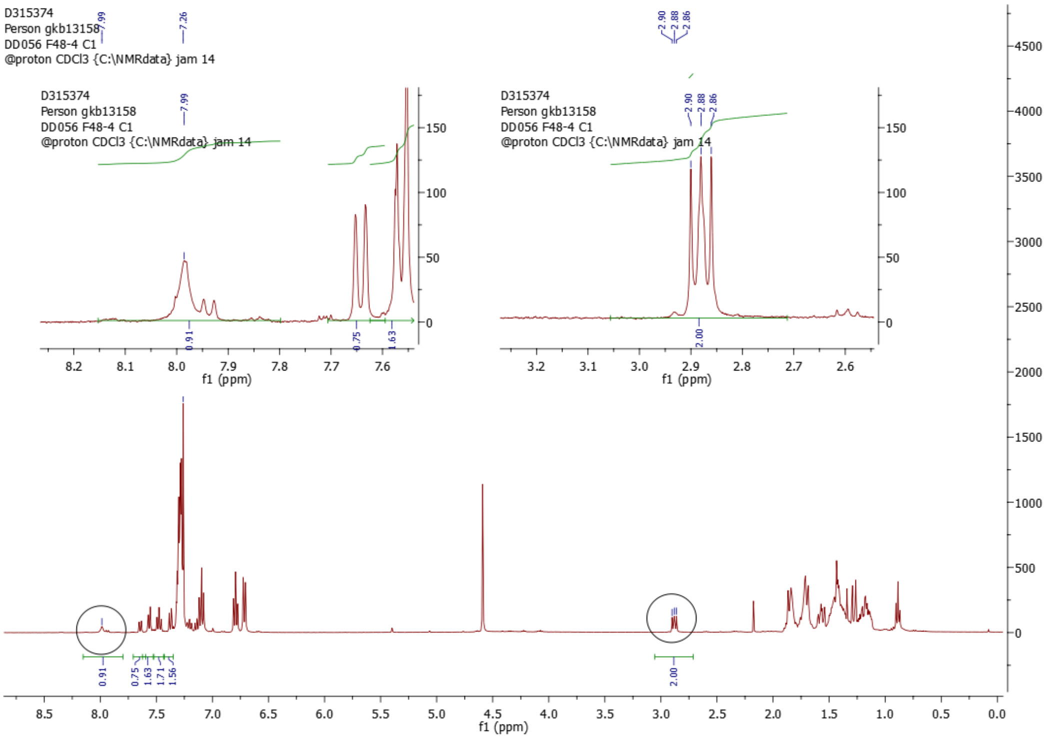

Figure S2. ^{1}H NMR data evidance for the formation of **78**

Treatment of 3,3-dimethyl-2-phenyl-3*H*-indole (72) with K/KO^{t}Bu

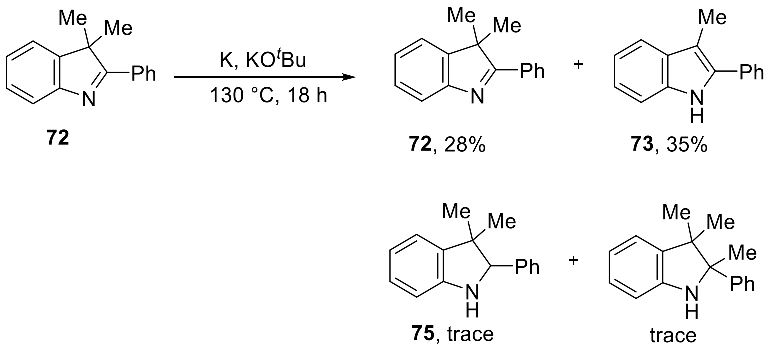

3,3-Dimethyl-2-phenyl-3*H*-indole **72** (1.0 eq., 0.5 mmol, 110.65 mg), potassium metal (1.3 eq., 0.66 mmol, 26 mg) and KO^{t}Bu (1.0 eq., 0.5 mmol, 56.11 mg) were sealed in a pressure tube in a nitrogen-

filled glovebox. The contents of the pressure tube were stirred at 130 °C for 18 h before the pressure tube was cooled to room temperature, opened to air, quenched with ^{i}PrOH at 0 °C and diluted with water (50 mL). The organic products were extracted into Et_2O (3 x 50 mL). The combined organic phases were dried over Na_2SO_4, filtered and concentrated. Purification by column chromatography (petroleum ether → 2% EtOAc/98% petroleum ether) afforded the 3,3-dimethyl-2-phenyl-3*H*-indole starting material **72** (31 mg, 28%) as a yellow oil and 3-methyl-2-phenyl-1*H*-indole **73** (36 mg, 35%) as a white solid.

3-methyl-2-phenyl-1*H*-indole: **^{1}H NMR** (400 MHz, $CDCl_3$) δ 8.01 (br s, 1H, NH), 7.70 – 7.55 (m, 3H, 3 x ArH), 7.52 – 7.48 (m, 2H, 2 x Ar), 7.40 – 7.37 (m, 2H, 2 x ArH), 7.26 – 7.16 (m, 2H, 2 x ArH), 2.49 (s, 3H, Me). ***m/z* (EI):** 207.1 (M^+, 100), 190.9 (2), 178.1 (14), 152.1 (4), 139.1 (2), 130.0 (39), 115.0 (3), 102.1 (21), 89.0 (7), 77.0 (45), 63.0 (12), 51.0 (24). The data are consistent with the data reported earlier in this report.

3,3-dimethyl-2-phenyl-3*H*-indole: **^{1}H NMR** (400 MHz, $CDCl_3$) δ 8.19 – 8.14 (m, 2H, 2 x ArH), 7.72 (d, *J* = 7.6 Hz, 1H, ArH), 7.58 – 7.45 (m, 3H, 3 x ArH), 7.42 – 7.32 (m, 2H, 2 x ArH), 7.33 – 7.23 (m, 1H, ArH), 1.61 (s, 6H, 2 x Me). **^{13}C NMR** (101 MHz, $CDCl_3$) δ 183.4, 153.0, 147.7, 133.3, 130.8, 128.8, 128.5, 127.9, 126.1, 121.1, 121.0, 53.7, 24.9. **ATR-IR** ν_{max} 3057, 2965, 2926, 2864, 1520, 1491, 1470, 1454, 1441 ,1385, 1364, 1337, 1315, 1265, 1209, 1169, 1156, 1109, 1088, 1074, 1032, 1003, 988, 935, 862, 824, 772, 750, 731. ***m/z* (EI):** 221.1 (M^+, 100%), 206.1 (54), 178.0 (5), 144.0 (15), 128.1 (9), 115.0 (33), 103.0 (56), 91.0 (24), 77.0 (62), 63.0 (12), 51.0 (27). The data are consistent with those previously reported in the literature[1]

Trace amounts of 3,3-dimethyl-2-phenylindoline **75** (RT 13.75 min, *m/z* 223.1) and 2,3,3-trimethyl-2-phenylindoline (RT 13.97 min, *m/z* 237.1) were detected in the crude reaction mixture by GC-MS and ^{1}H NMR.

Treatment of 3,3-dimethyl-2-phenyl-3*H*-indole (72) with Et_3SiH/KO^{t}Bu/TEMPO

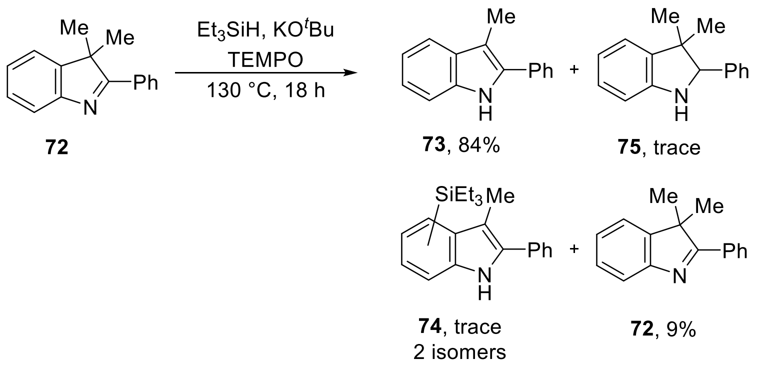

3,3-Dimethyl-2-phenyl-3*H*-indole **72** (1.0 eq., 0.5 mmol, 110.65 mg), Et_3SiH (3.0 eq., 1.5 mmol, 240 µL), KO^{t}Bu (3.0 eq., 1.5 mmol, 168.32 mg) and TEMPO (1.0 eq., 0.5 mmol, 78.125 mg) were sealed in a pressure tube in a nitrogen-filled glovebox. The contents of the pressure tube were stirred at 130

°C for 18 h before the pressure tube was cooled to room temperature, opened to air and diluted with water (50 mL). The organic products were extracted into Et_2O (3 x 50 mL). The combined organic phases were dried over Na_2SO_4, filtered and concentrated. Purification by column chromatography afforded 3-methyl-2-phenyl-1*H*-indole **73** (87 mg, 84%) as a white solid and the 3,3-dimethyl-2-phenyl-3*H*-indole starting material **72** (10 mg, 9%) as a brown oil.

3-methyl-2-phenyl-1*H*-indole: **^{1}H NMR** (400 MHz, $CDCl_3$) δ 8.05 (br s, 1H, NH), 7.72 – 7.56 (m, 2H, 2 x ArH), 7.54 – 7.46 (m, 2H, 2 x ArH), 7.46 – 7.29 (m, 2H, 2 x ArH), 7.30 – 7.16 (m, 2H, 2 x ArH), 2.50 (s, 3H, Me). ***m/z* (EI):** 207.1 (M^+, 100%), 190.9 (2), 178.0 (15), 167.0 (2), 151.9 (4), 139.1 (2), 130.0 (40), 115.0 (3), 102.1 (22), 89.0 (8), 77.0 (48), 63.0 (14), 51.0 (26). The data are consistent with the data reported for this compound earlier in this report.

3,3-dimethyl-2-phenyl-3*H*-indole: **^{1}H NMR** (400 MHz, $CDCl_3$) δ 8.19 – 8.16 (m, 2H, 2 x ArH), 7.73 (d, *J* = 7.5 Hz, 1H, ArH), 7.60 – 7.45 (m, 3H, 3 x ArH), 7.42 – 7.32 (m, 2H, 2 x ArH), 7.32 – 7.26 (m, 1H, ArH), 1.61 (s, 6H, 2 x Me). **^{13}C NMR** (101 MHz, $CDCl_3$) δ 183.5, 155.1, 147.5, 133.0, 131.0, 128.9, 128.7, 128.0, 126.2, 121.1, 120.9, 53.7, 25.0. **ATR-IR** ν_{max} 3059, 2963, 2926, 2857, 1520, 1491, 1468, 1454, 1443, 1387, 1364, 1337, 1315, 1263, 1248, 1221, 1209, 1169, 1155, 1132, 1109, 1003, 935, 862, 773, 752, 696. ***m/z* (EI):** 220.9 (M^+, 48%), 205.9 (32), 180.0 (2), 164.8 (8), 144.0 (13), 127.9 (5), 115.0 (31), 103.0 (82), 90.9 (29), 77.0 (100), 63.0 (18), 51.0 (51). The data are consistent with those previously reported in the literature[1]

Trace amounts of 3,3-dimethyl-2-phenylindoline **75** (RT 13.72 min, *m/z* 223.1) and 2 major isomers of silylated 3-methyl-2-phenyl-1*H*-indole **74** (RT 17.41 and 17.81 min, *m/z* 321.1 and *m/z* 320.9) were detected in the crude reaction mixture by GC-MS and ^{1}H NMR.

Et_3Si-TEMPO adduct (predicted *m/z* 271.2) was detected by GC-MS when the reaction mixture was quenched carefully with chloroform at 0 °C and no aqueous work-up was performed prior to analysis. This was consistent with the detection of the Et_3Si-TEMPO adduct that was previosuly reported in the literature.[32,33] It is important to note that diminished amounts of 3-methyl-2-phenyl-1*H*-indole were observed by crude GC-MS, possibly due to 3-methyl-2-phenyl-1*H*-indole reacting with dichlorocarbene, resulting from the reaction between chloroform and KO^{t}Bu. The reaction between indoles and dichlorocarbenes is known in the literature as the Ciamician-Dennstedt rearrangement.

```
File        :D:\Data\July20\DD178_Crude.D
Operator    :
Acquired    : 21 Aug 2020   12:02      using AcqMethod EI320.M
Instrument :    GCMS_AG
Sample Name: DD178_Crude
Misc Info   :
Vial Number: 3
```

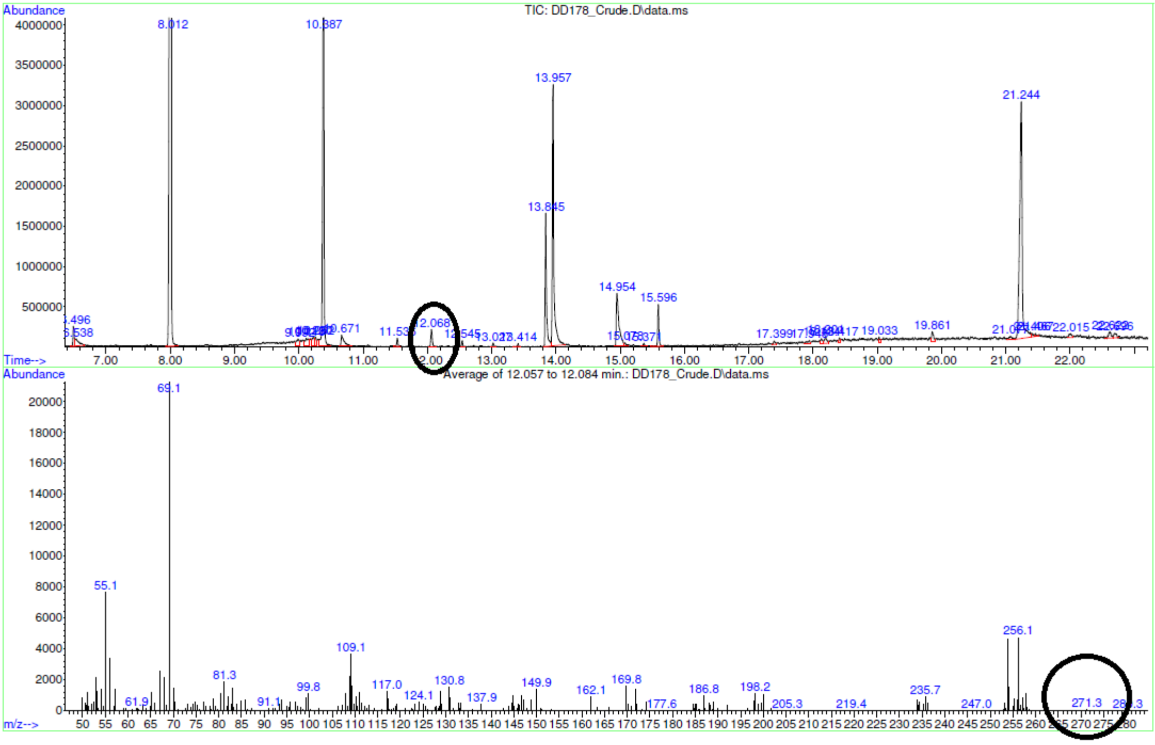

Figure S3. GC-MS evidance for the formation of Et_3Si-TEMPO

NMR Spectra

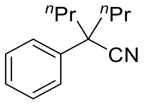

nPr
nPr
CN

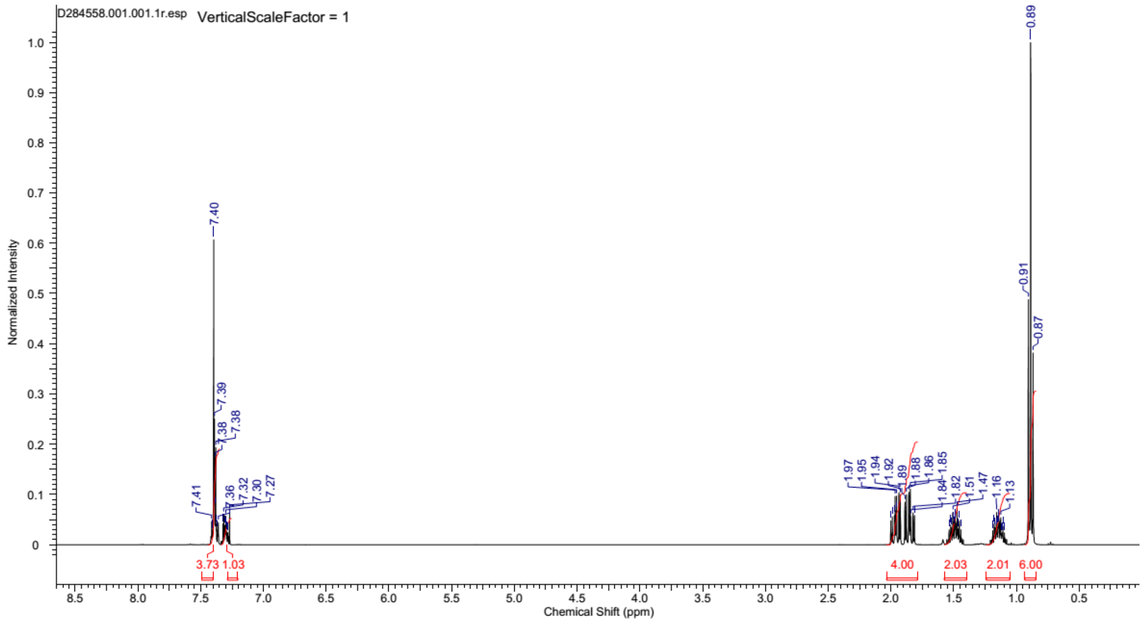

D284558.001.001.1r.esp
VerticalScaleFactor = 1
Normalized Intensity
Chemical Shift (ppm)
7.40
0.89
0.91
0.87
3.73
1.03
4.00
2.03
2.01
6.00

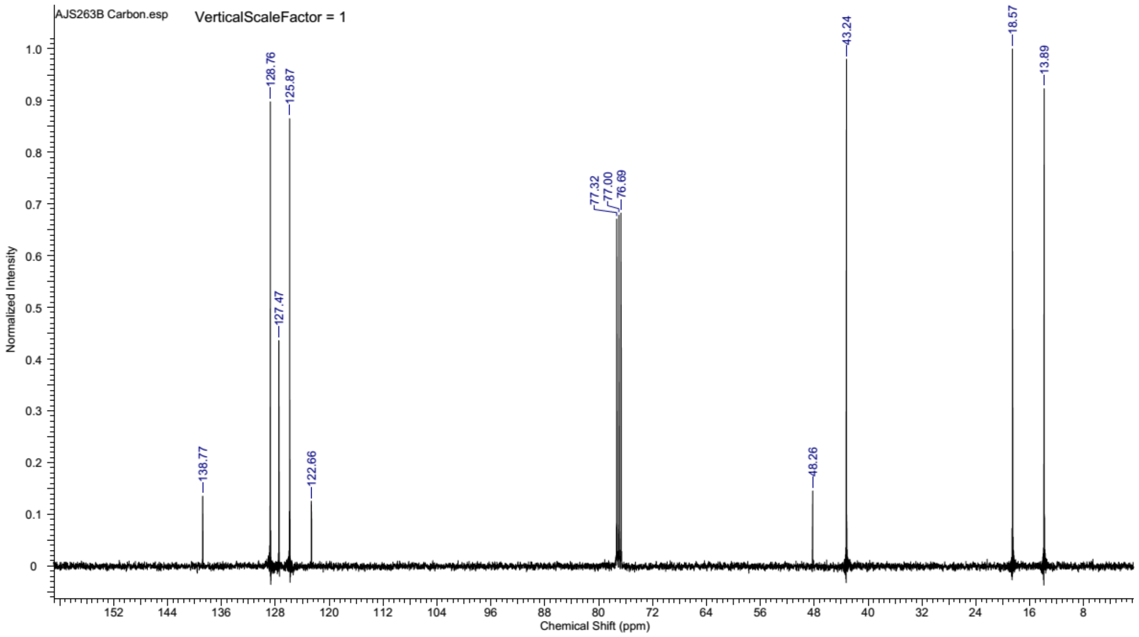

AJS263B Carbon.esp
VerticalScaleFactor = 1
Normalized Intensity
Chemical Shift (ppm)
128.76
125.87
127.47
138.77
122.66
77.32
77.00
76.69
48.26
43.24
18.57
13.89

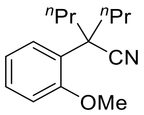

nPr
nPr
CN
OMe

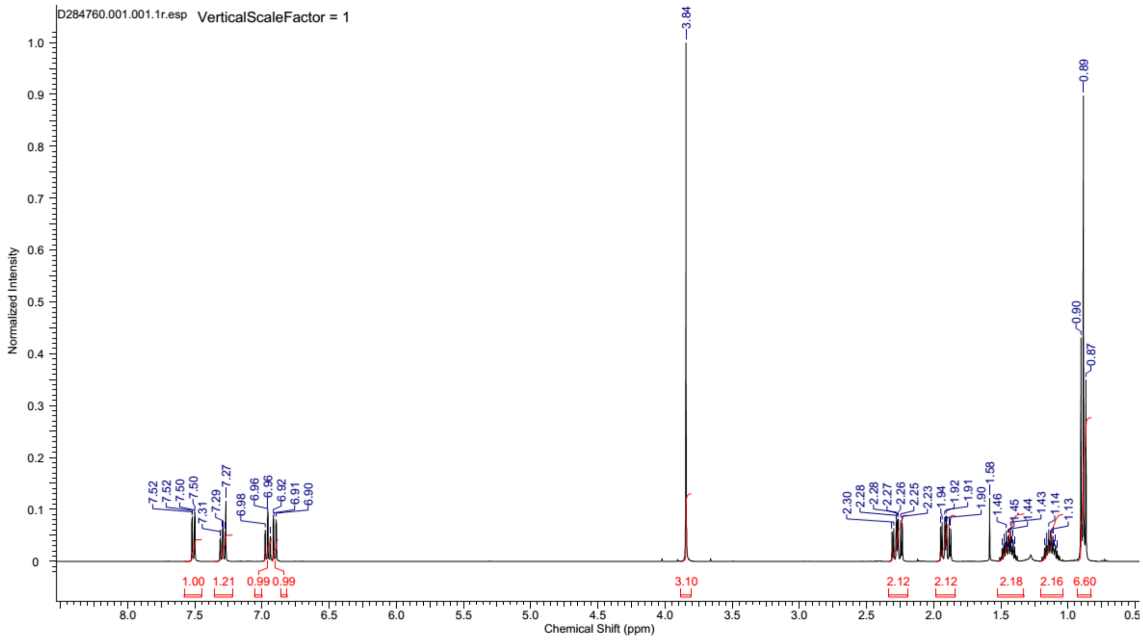

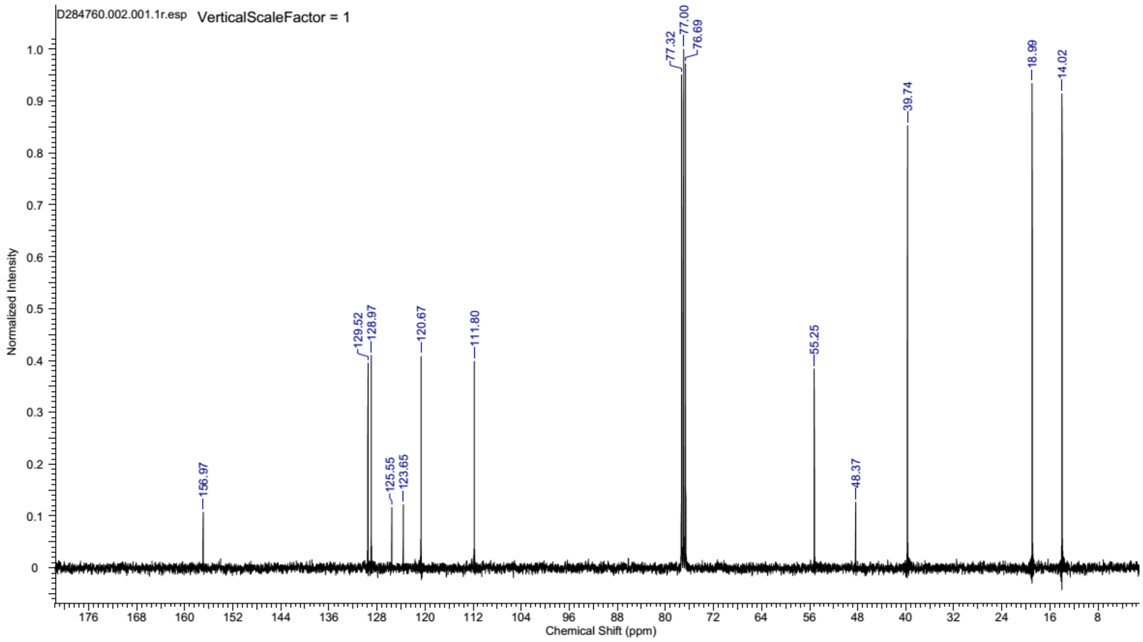

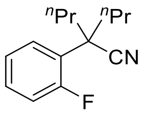

^{n}Pr
^{n}Pr
CN
F

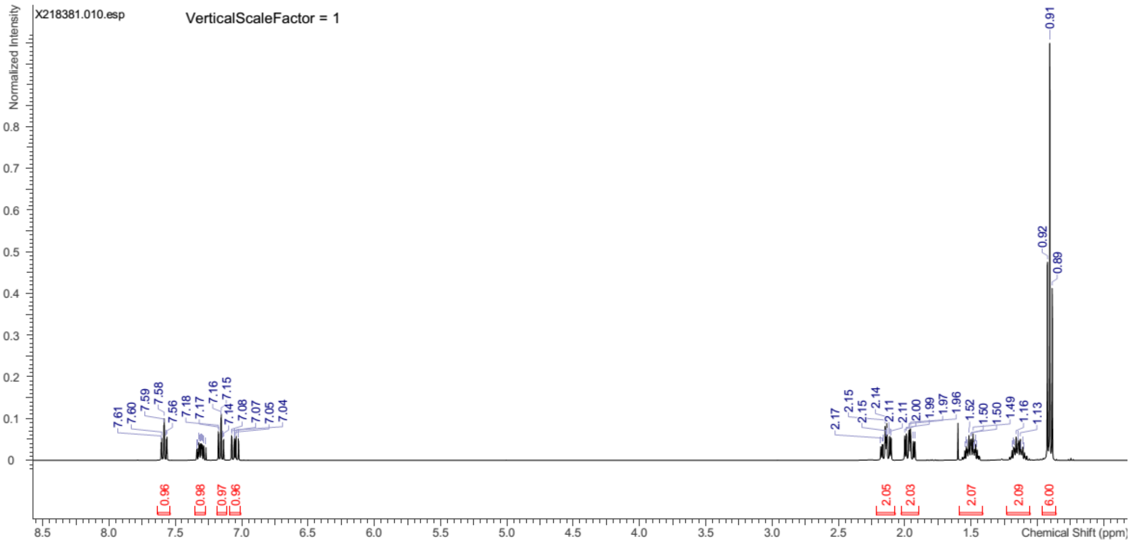

X218381.010.esp
VerticalScaleFactor = 1
Normalized Intensity
0.91
0.92
0.89
7.61
7.60
7.59
7.58
7.56
7.18
7.17
7.16
7.15
7.14
7.08
7.07
7.05
7.04
2.17
2.15
2.15
2.14
2.11
2.11
2.00
1.99
1.97
1.96
1.52
1.50
1.50
1.49
1.16
1.13
0.96
0.98
0.97
0.96
2.05
2.03
2.07
2.09
6.00
Chemical Shift (ppm)

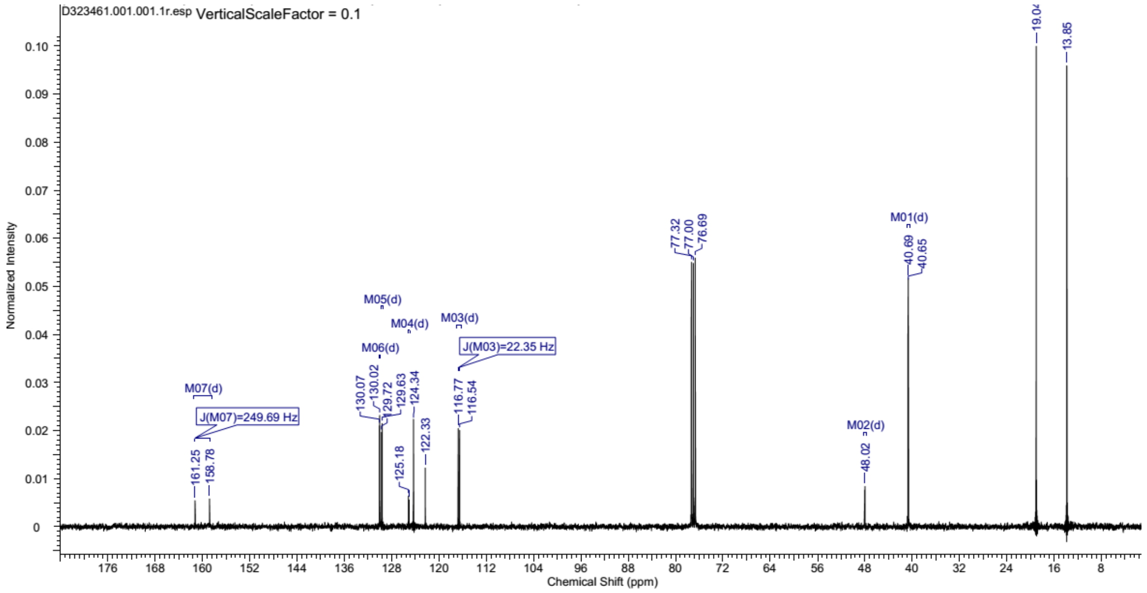

D323461.001.001.1r.esp
VerticalScaleFactor = 0.1
Normalized Intensity
19.04
13.85
77.32
77.00
76.69
M01(d)
40.69
40.65
M02(d)
48.02
M05(d)
M04(d)
M03(d)
M06(d)
J(M03)=22.35 Hz
130.07
130.02
129.72
129.63
124.34
125.18
122.33
116.77
116.54
M07(d)
J(M07)=249.69 Hz
161.25
158.78
Chemical Shift (ppm)

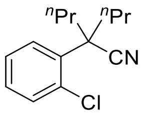

nPr
nPr
CN
Cl

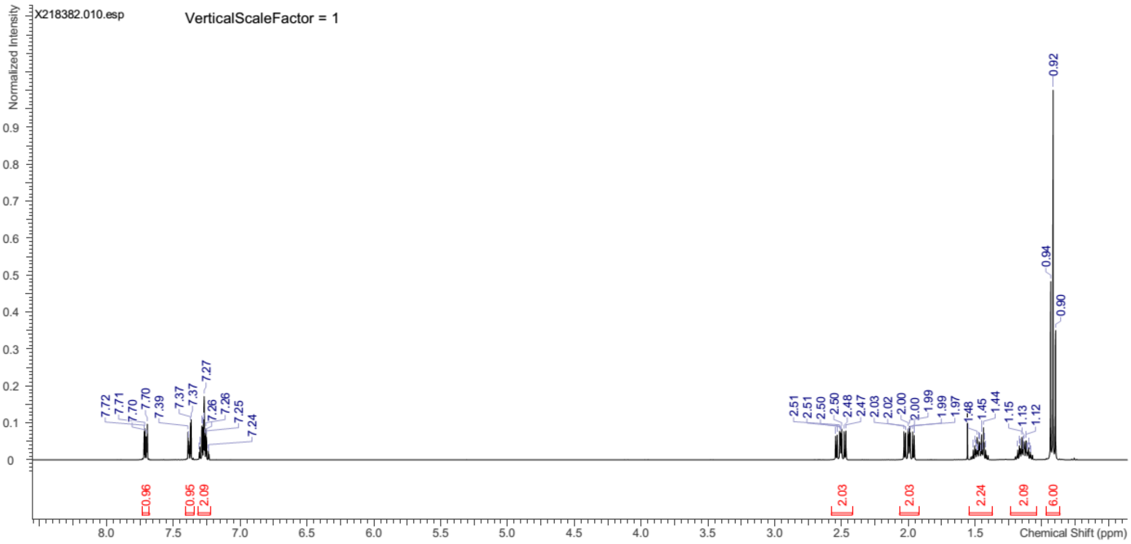

X218382.010.esp
VerticalScaleFactor = 1
Normalized Intensity
7.72
7.71
7.70
7.70
7.39
7.37
7.37
7.27
7.26
7.26
7.25
7.24
2.51
2.51
2.50
2.50
2.48
2.47
2.03
2.02
2.00
2.00
1.99
1.99
1.97
1.48
1.45
1.44
1.15
1.13
1.12
0.94
0.92
0.90
0.96
0.95
2.09
2.03
2.03
2.24
2.09
6.00
Chemical Shift (ppm)

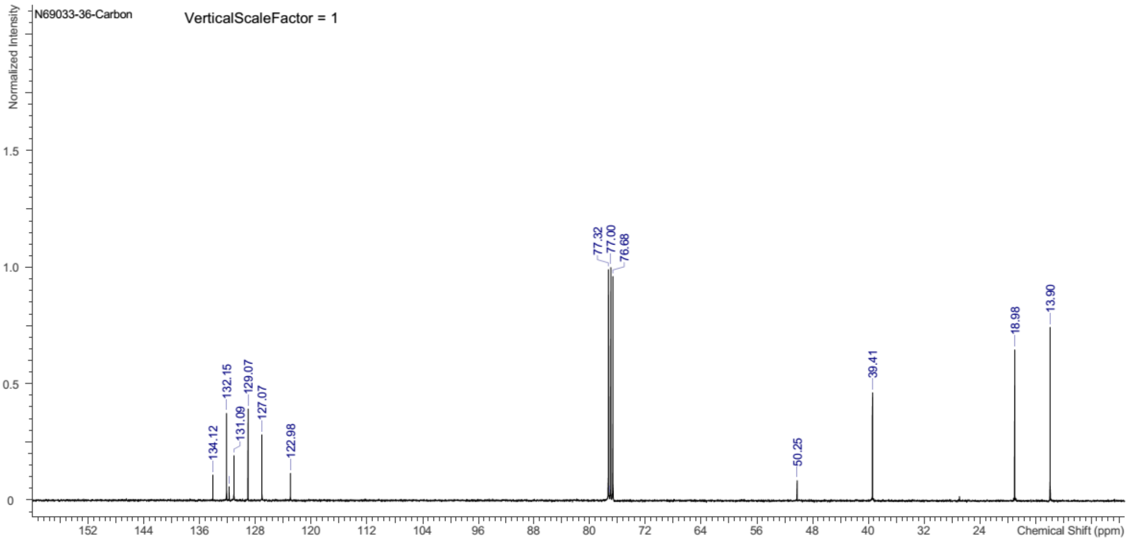

N69033-36-Carbon
VerticalScaleFactor = 1
Normalized Intensity
134.12
132.15
131.09
129.07
127.07
122.98
77.32
77.00
76.68
50.25
39.41
18.98
13.90
Chemical Shift (ppm)

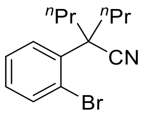

^{n}Pr
^{n}Pr
CN
Br

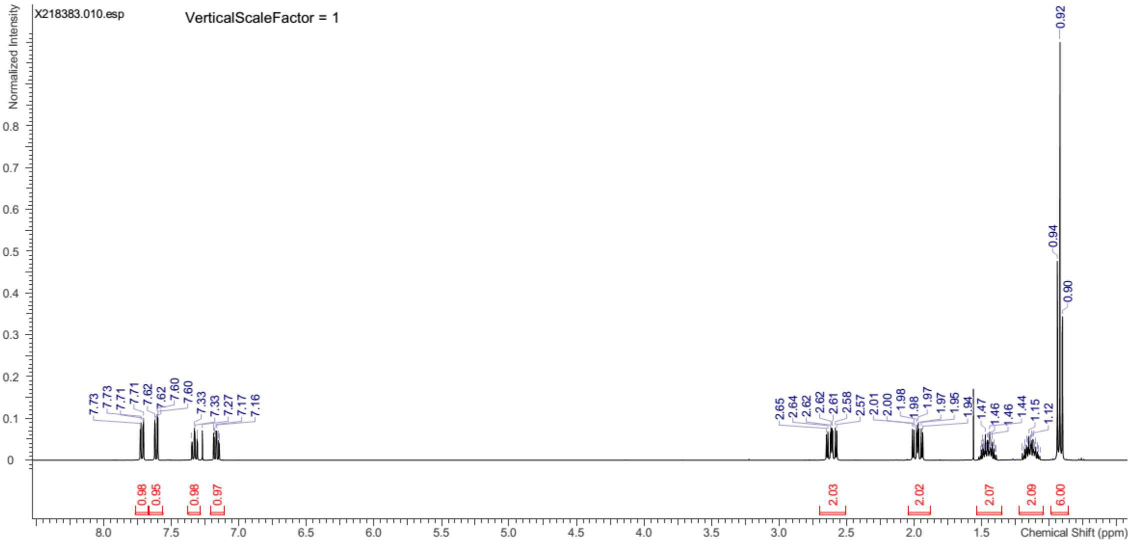

X218383.010.esp
VerticalScaleFactor = 1
Normalized Intensity
7.73 7.73 7.71 7.71 7.62 7.62 7.60 7.60 7.33 7.33 7.27 7.17 7.16
2.65 2.64 2.62 2.62 2.61 2.58 2.57 2.01 2.00 1.98 1.98 1.97 1.97 1.95 1.94
1.47 1.46 1.46 1.44 1.15 1.12
0.94 0.92 0.90
0.98 0.95 0.98 0.97 2.03 2.02 2.07 2.09 6.00
Chemical Shift (ppm)

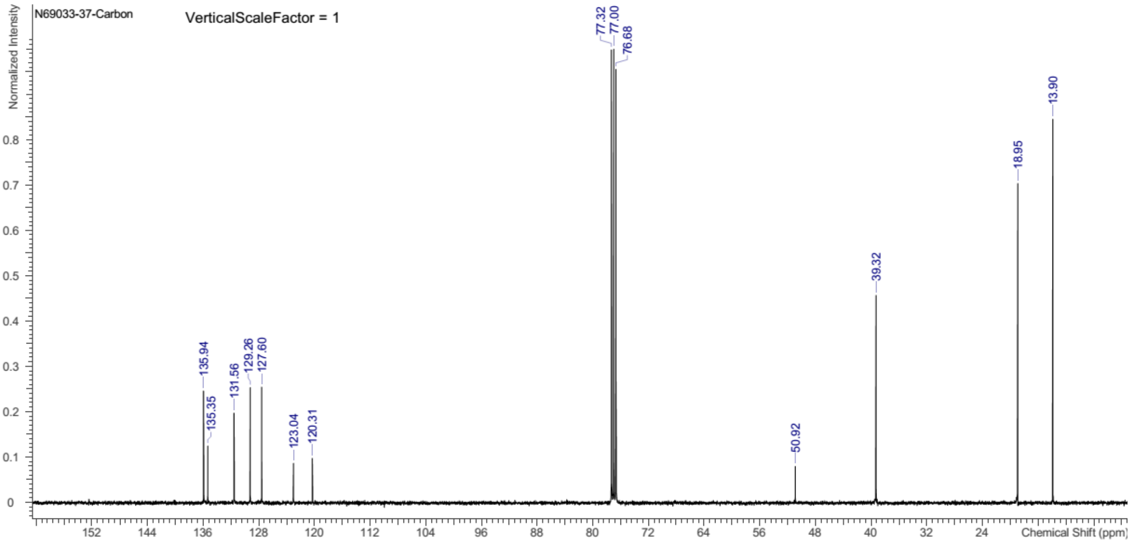

N69033-37-Carbon
VerticalScaleFactor = 1
Normalized Intensity
135.94 135.35 131.56 129.26 127.60 123.04 120.31
77.32 77.00 76.68
50.92
39.32
18.95
13.90
Chemical Shift (ppm)

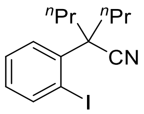

^{n}Pr
^{n}Pr
CN
I

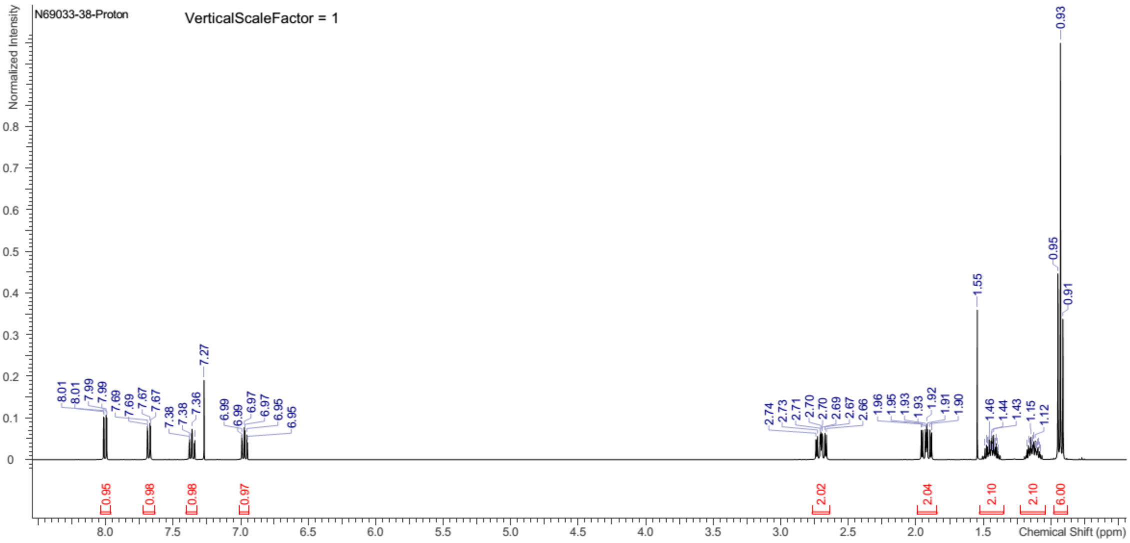

N69033-38-Proton
VerticalScaleFactor = 1
Normalized Intensity
8.01 8.01 7.99 7.99 7.69 7.69 7.67 7.67 7.38 7.38 7.36 7.27 6.99 6.99 6.97 6.97 6.95 6.95
2.74 2.73 2.71 2.70 2.70 2.69 2.67 2.66 1.96 1.95 1.93 1.93 1.92 1.91 1.90 1.55 1.46 1.44 1.43 1.15 1.12 0.95 0.93 0.91
0.95 0.98 0.98 0.97 2.02 2.04 2.10 2.10 6.00
Chemical Shift (ppm)

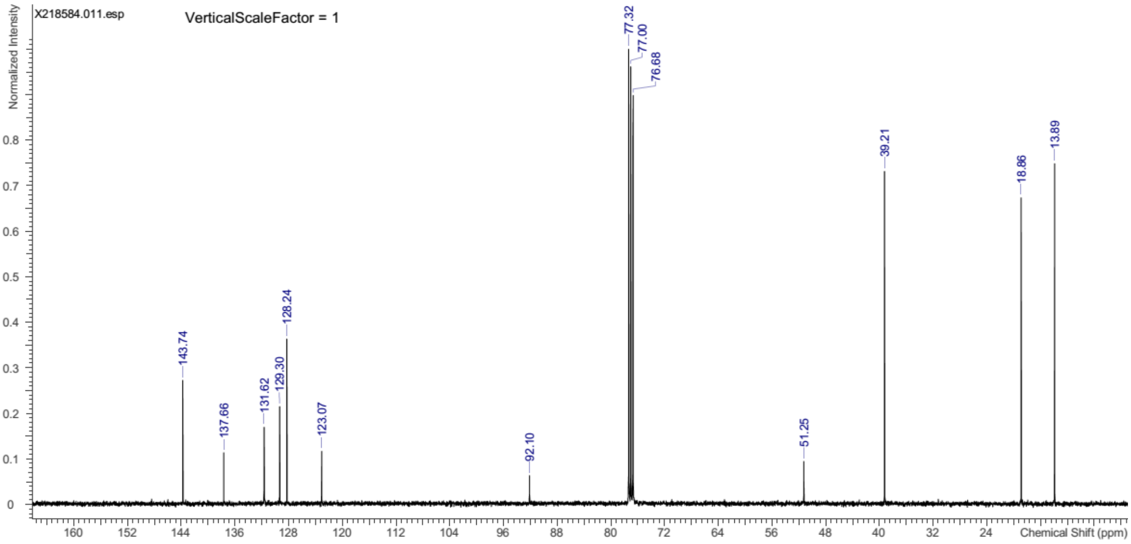

X218584.011.esp
VerticalScaleFactor = 1
Normalized Intensity
143.74 137.66 131.62 129.30 128.24 123.07 92.10 77.32 77.00 76.68 51.25 39.21 18.86 13.89
Chemical Shift (ppm)

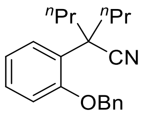

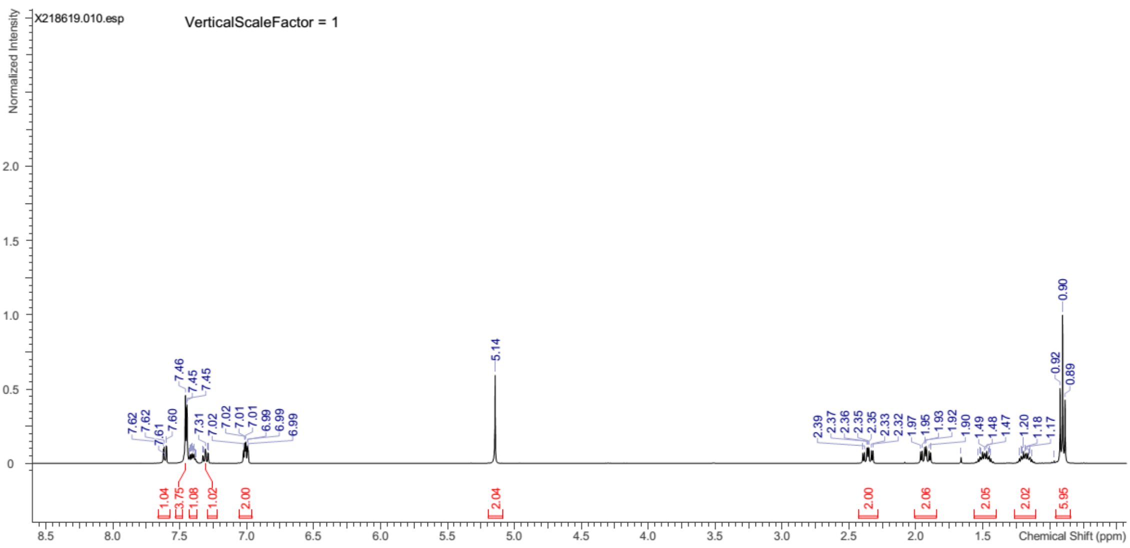
X218619.010.esp
VerticalScaleFactor = 1
Normalized Intensity
Chemical Shift (ppm)

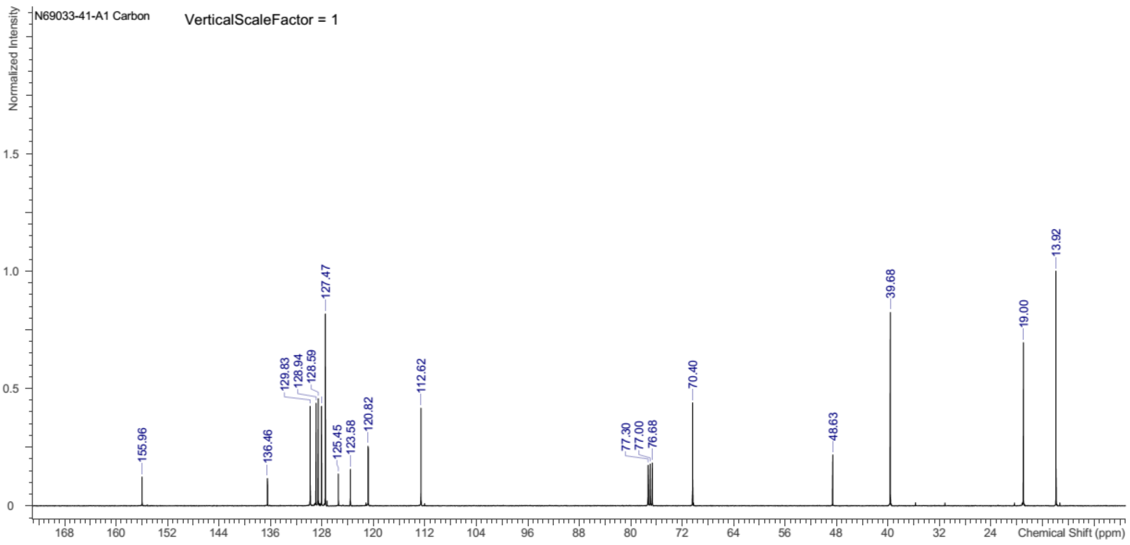
N69033-41-A1 Carbon
VerticalScaleFactor = 1
Normalized Intensity
Chemical Shift (ppm)

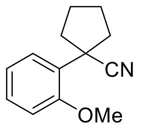
CN
OMe

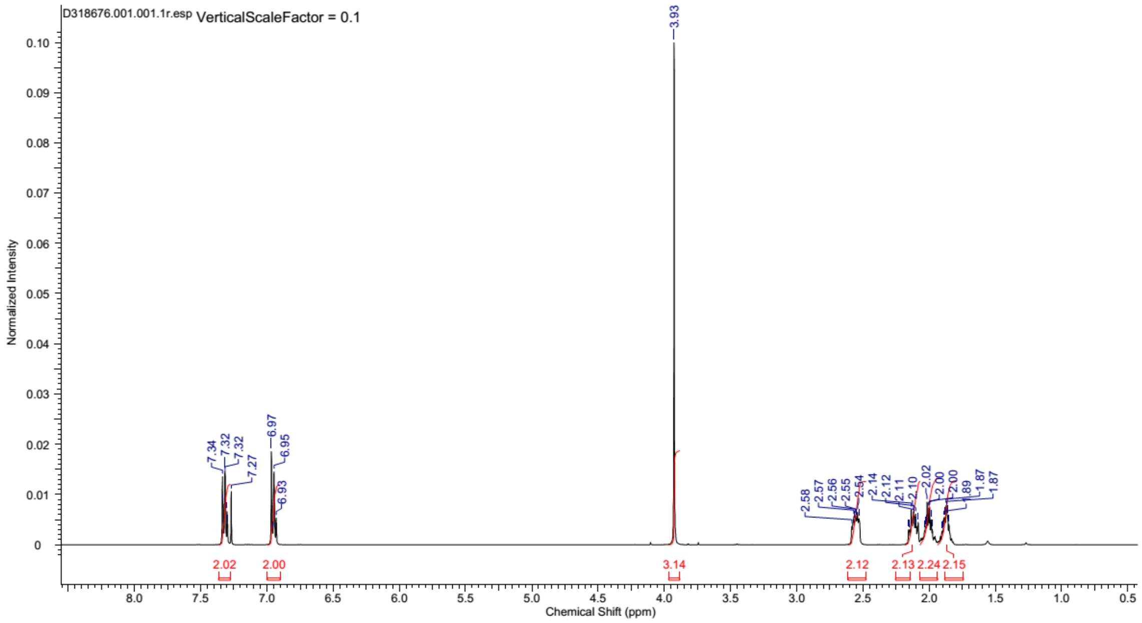
D318676.001.001.1r.esp VerticalScaleFactor = 0.1
Normalized Intensity
Chemical Shift (ppm)
3.93
7.34
7.32
7.32
7.27
6.97
6.95
6.93
2.58
2.57
2.56
2.55
2.54
2.14
2.12
2.11
2.10
2.02
2.00
2.00
1.89
1.87
1.87
2.02
2.00
3.14
2.12
2.13
2.24
2.15

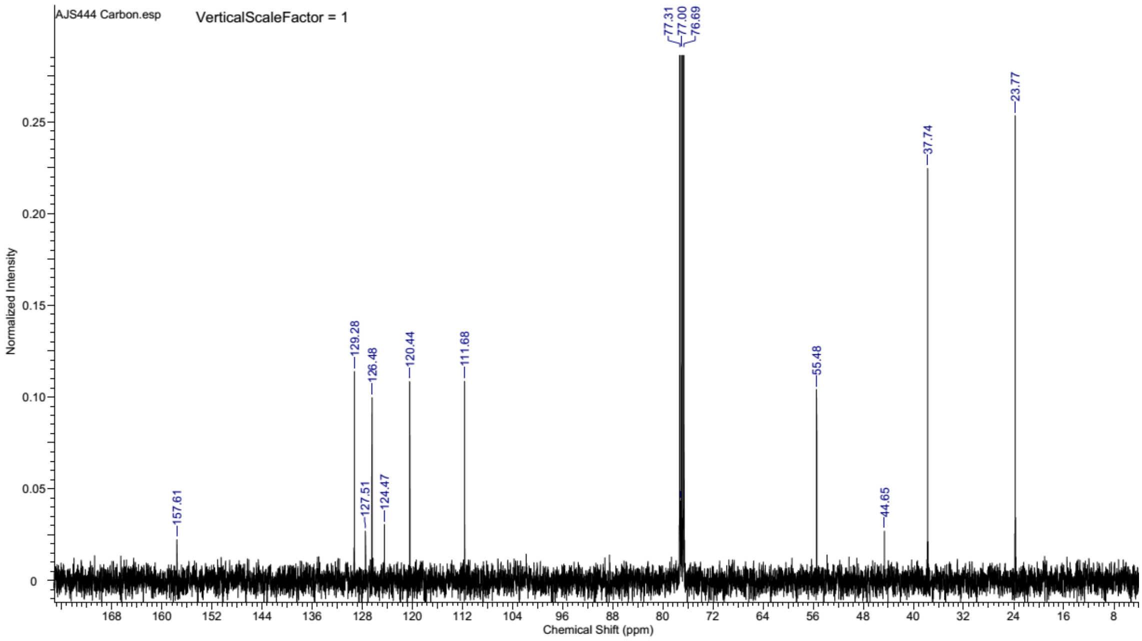
AJS444 Carbon.esp
VerticalScaleFactor = 1
Normalized Intensity
Chemical Shift (ppm)
77.31
77.00
76.69
23.77
37.74
129.28
128.48
120.44
111.68
55.48
157.61
127.51
124.47
44.65

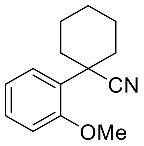
CN
OMe

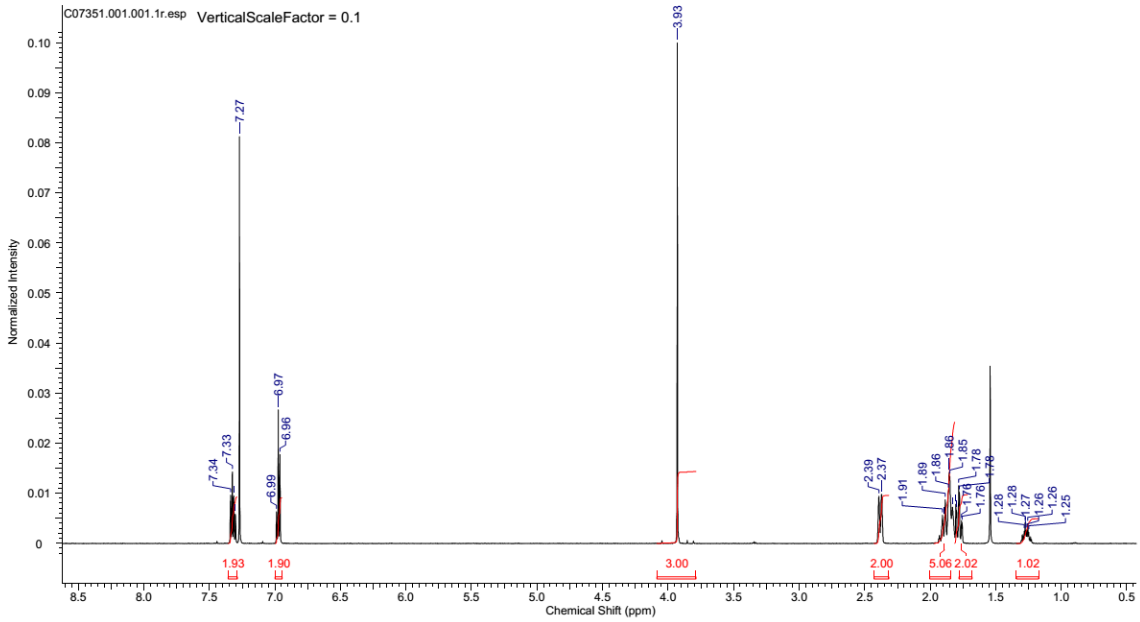
C07351.001.001.1r.esp VerticalScaleFactor = 0.1
Normalized Intensity
Chemical Shift (ppm)
7.34
7.33
7.27
6.99
6.97
6.96
3.93
2.39
2.37
1.91
1.89
1.86
1.86
1.85
1.78
1.78
1.78
1.76
1.28
1.28
1.27
1.26
1.25
1.93
1.90
3.00
2.00
5.06
2.02
1.02

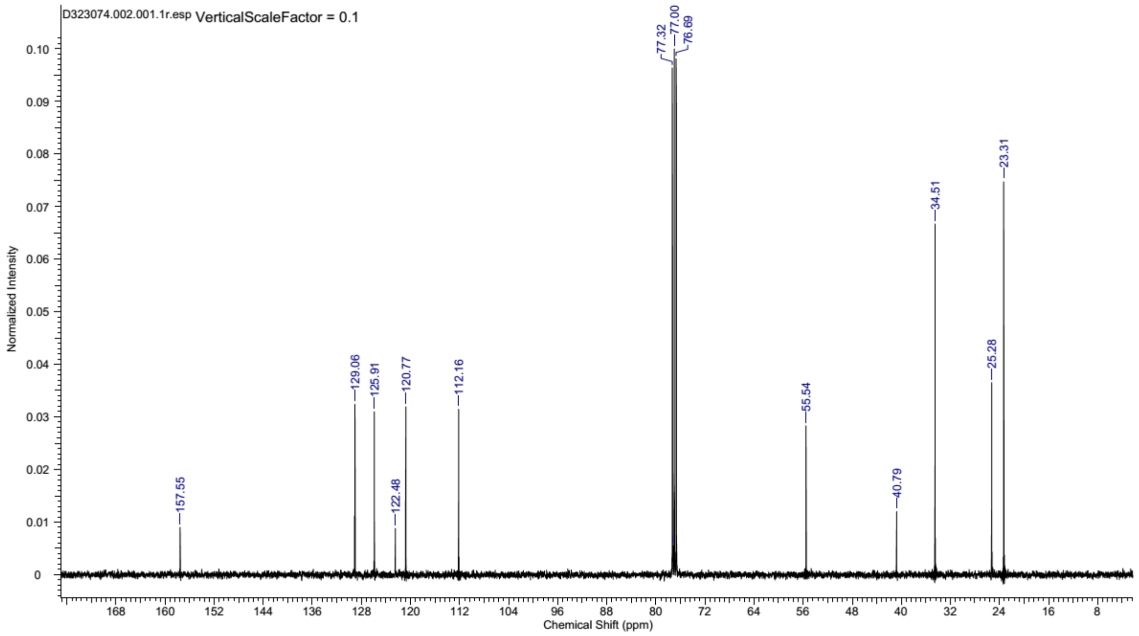
D323074.002.001.1r.esp VerticalScaleFactor = 0.1
Normalized Intensity
Chemical Shift (ppm)
157.55
129.06
125.91
122.48
120.77
112.16
77.32
77.00
76.69
55.54
40.79
34.51
25.28
23.31

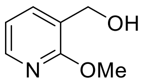
OH
N
OMe

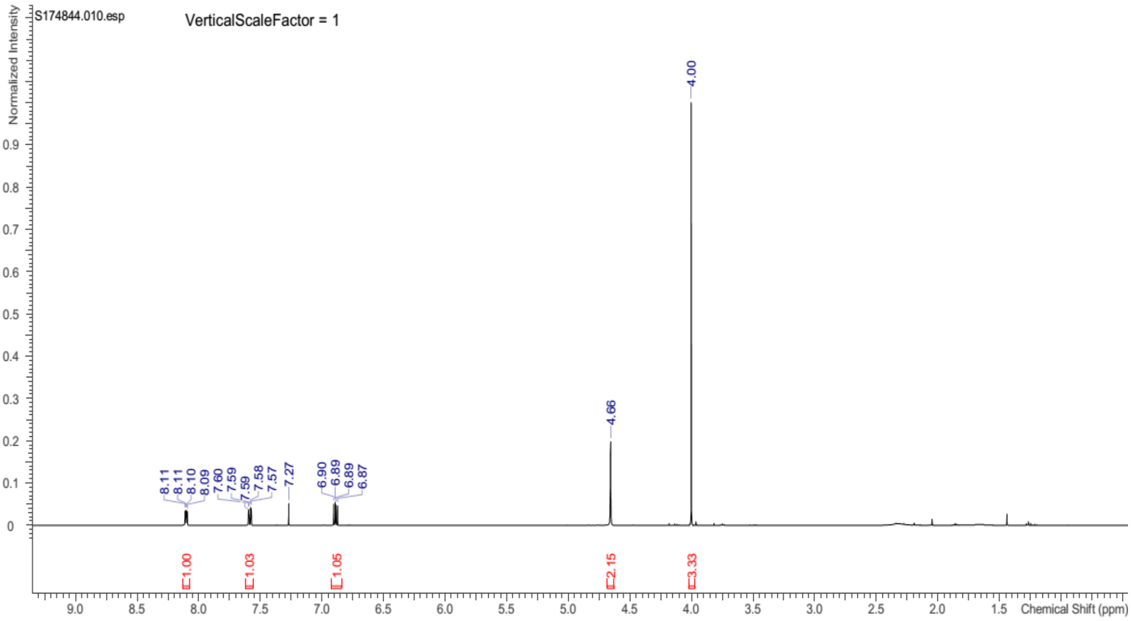
S174844.010.esp
VerticalScaleFactor = 1
Normalized Intensity
8.11
8.11
8.10
8.09
7.60
7.59
7.59
7.58
7.57
7.27
6.90
6.89
6.89
6.87
4.66
4.00
1.00
1.03
1.05
2.15
3.33
Chemical Shift (ppm)

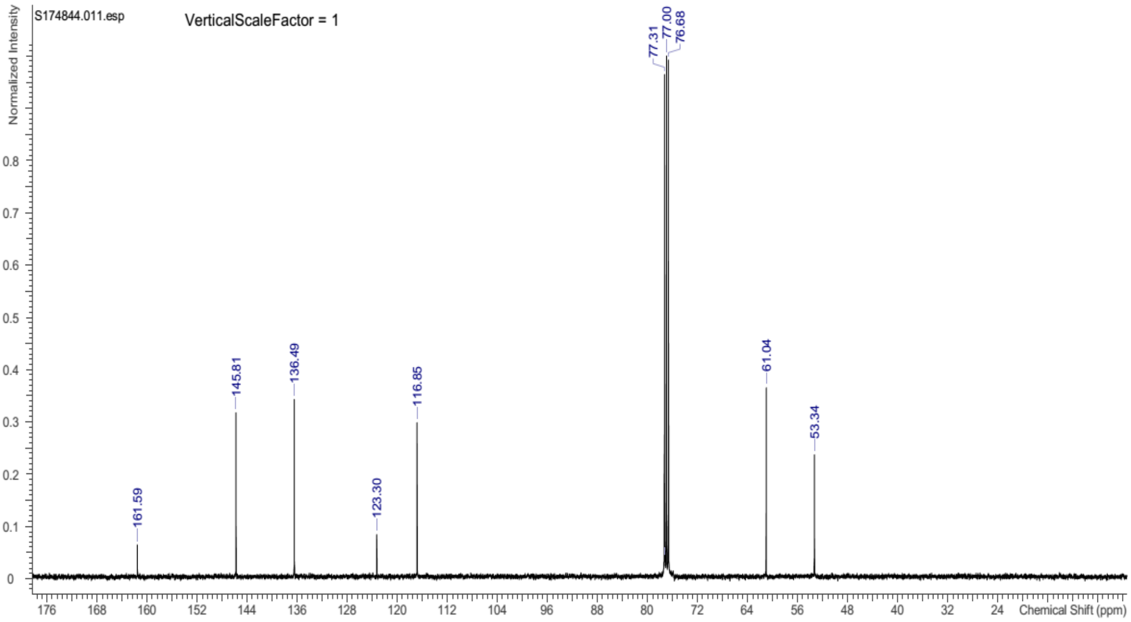
S174844.011.esp
VerticalScaleFactor = 1
Normalized Intensity
161.59
145.81
136.49
123.30
116.85
77.31
77.00
76.68
61.04
53.34
Chemical Shift (ppm)

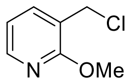

Cl
N
OMe

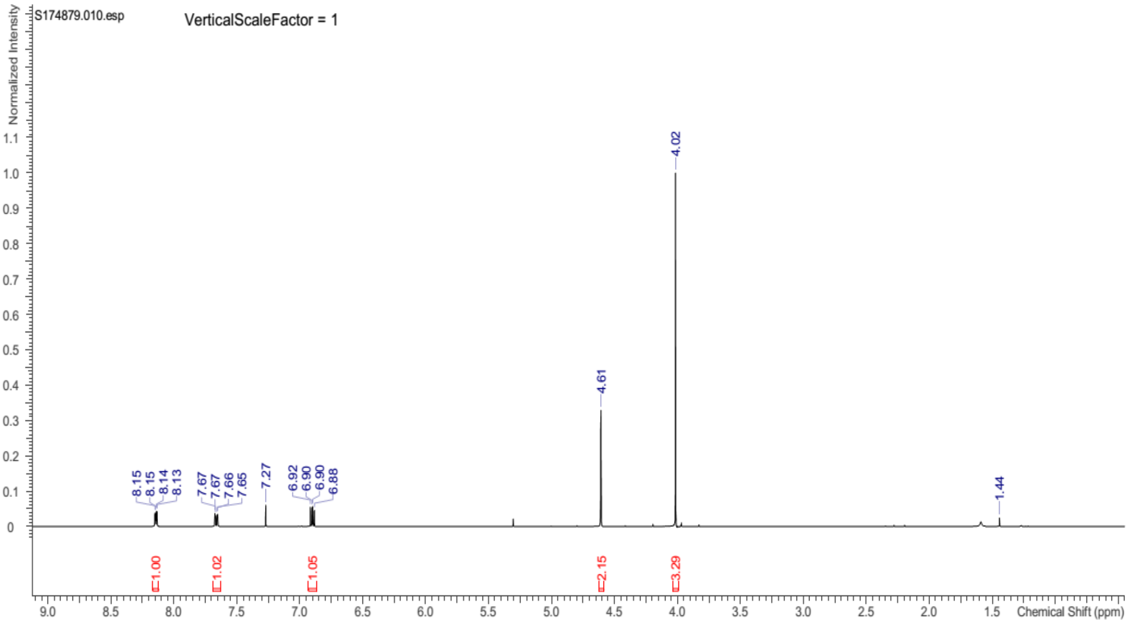

S174879.010.esp
VerticalScaleFactor = 1
Normalized Intensity
8.15
8.15
8.14
8.13
7.67
7.67
7.66
7.65
7.27
6.92
6.90
6.90
6.88
4.61
4.02
1.44
1.00
1.02
1.05
2.15
3.29
Chemical Shift (ppm)

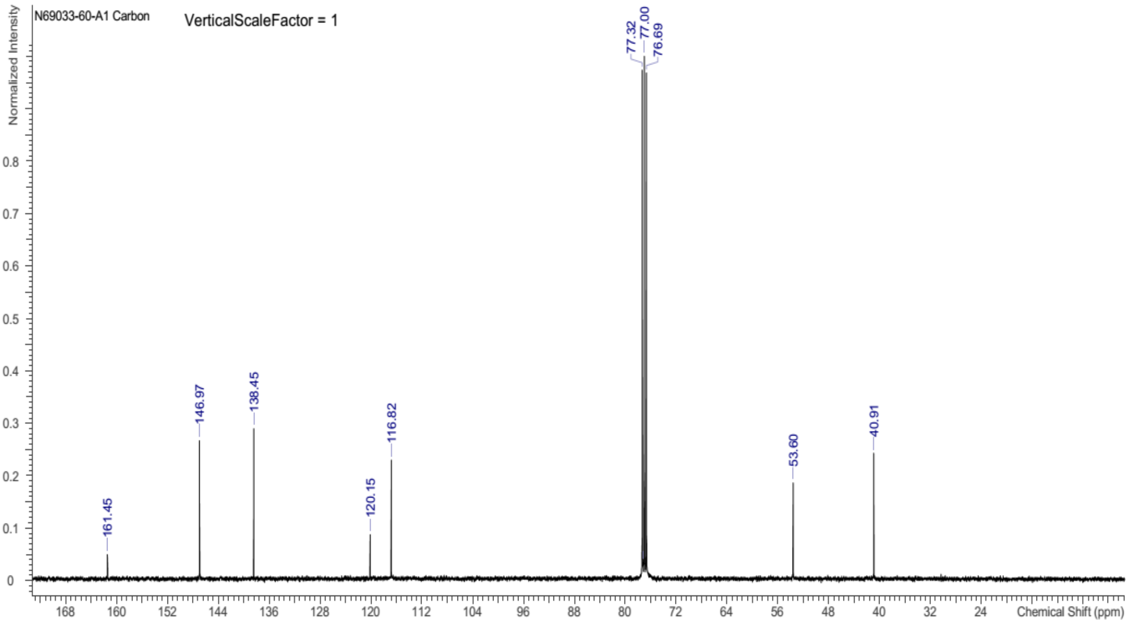

N69033-60-A1 Carbon
VerticalScaleFactor = 1
Normalized Intensity
161.45
146.97
138.45
120.15
116.82
77.32
77.00
76.69
53.60
40.91
Chemical Shift (ppm)

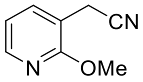
CN
N
OMe

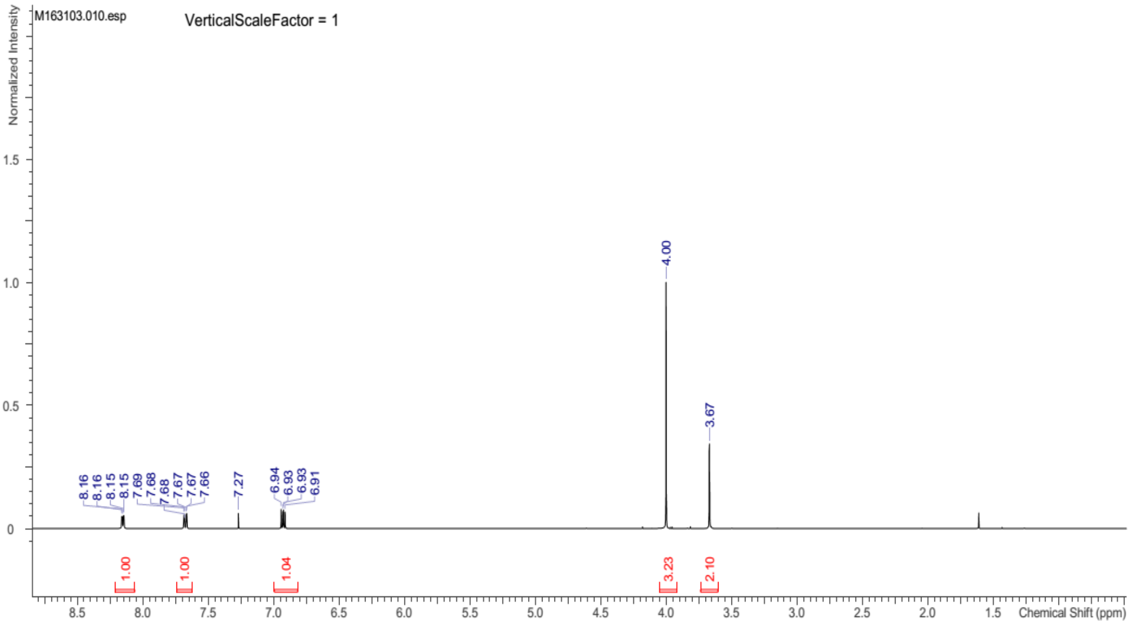
M163103.010.esp
VerticalScaleFactor = 1
Normalized Intensity
8.16
8.16
8.15
8.15
7.69
7.68
7.68
7.67
7.67
7.66
7.27
6.94
6.93
6.93
6.91
4.00
3.67
1.00
1.00
1.04
3.23
2.10
Chemical Shift (ppm)

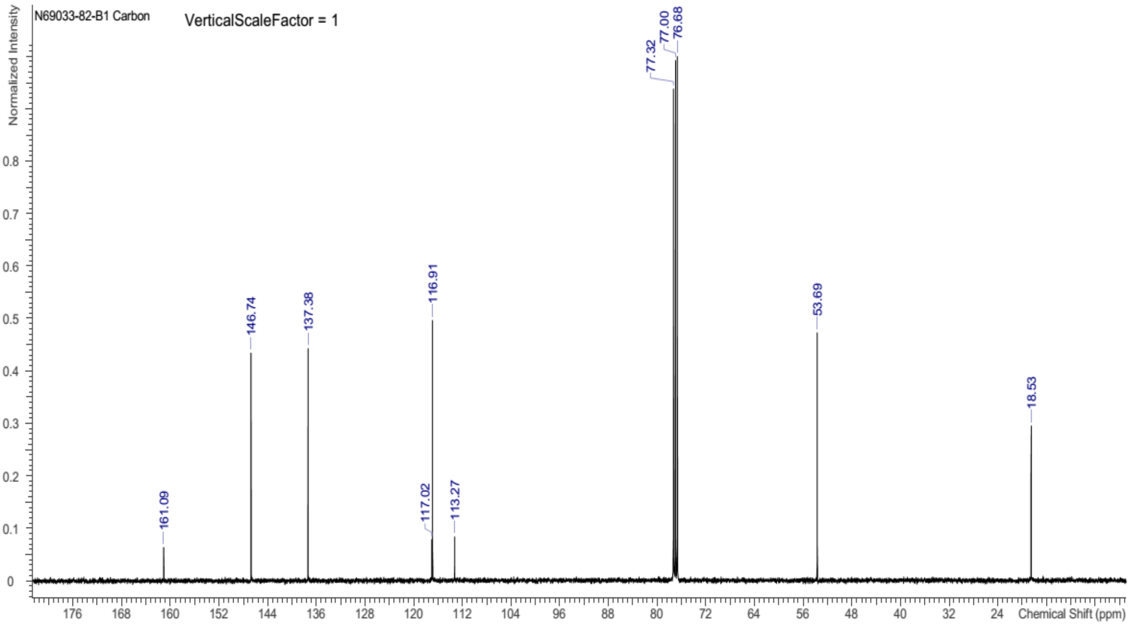
N69033-82-B1 Carbon
VerticalScaleFactor = 1
Normalized Intensity
161.09
146.74
137.38
117.02
116.91
113.27
77.32
77.00
76.68
53.69
18.53
Chemical Shift (ppm)

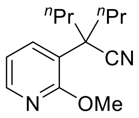

nPr
nPr
CN
N
OMe

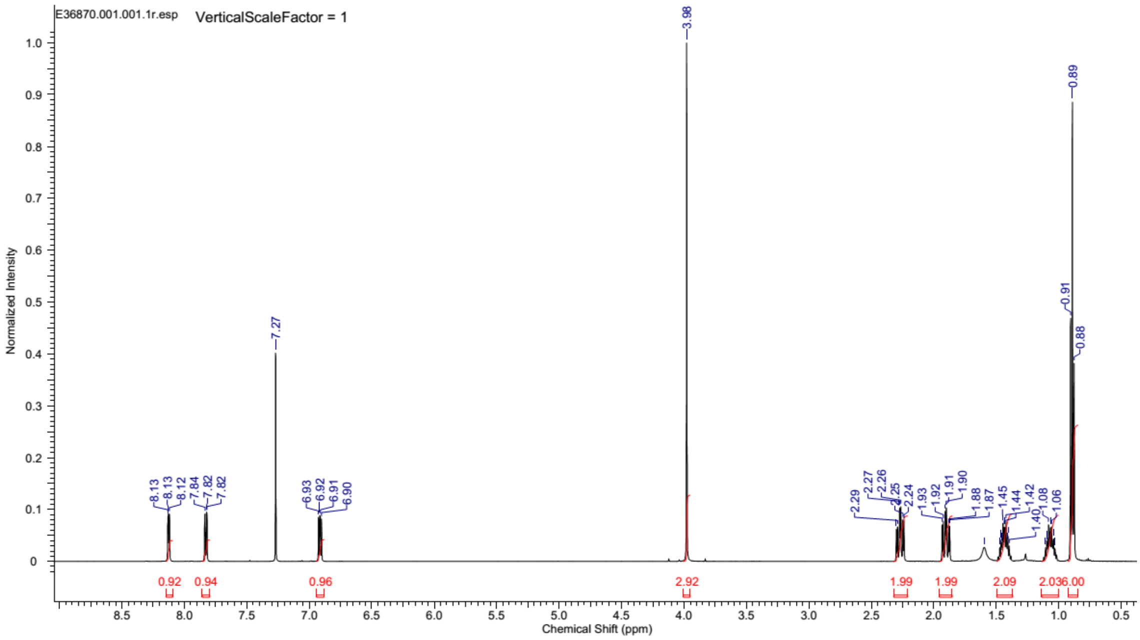

E36870.001.001.1r.esp
VerticalScaleFactor = 1
Normalized Intensity
Chemical Shift (ppm)
8.13 8.13 8.12 7.84 7.82 7.82
7.27
6.93 6.92 6.91 6.90
3.98
2.29 2.27 2.26 2.25 2.24 1.93 1.92 1.91 1.90 1.88 1.87 1.45 1.44 1.42 1.40 1.08 1.06
0.91 0.89 0.88
0.92 0.94 0.96 2.92 1.99 1.99 2.09 2.03 6.00

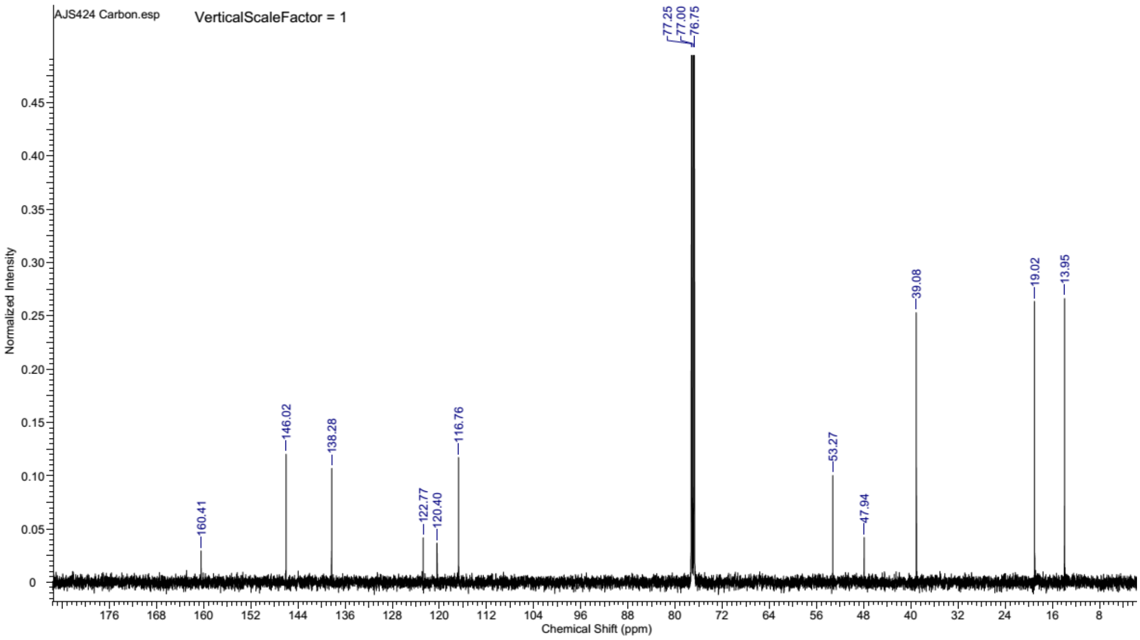

AJS424 Carbon.esp
VerticalScaleFactor = 1
Normalized Intensity
Chemical Shift (ppm)
160.41
146.02
138.28
122.77 120.40
116.76
77.25 77.00 76.75
53.27
47.94
39.08
19.02
13.95

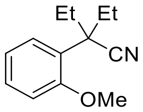
Et
Et
CN
OMe

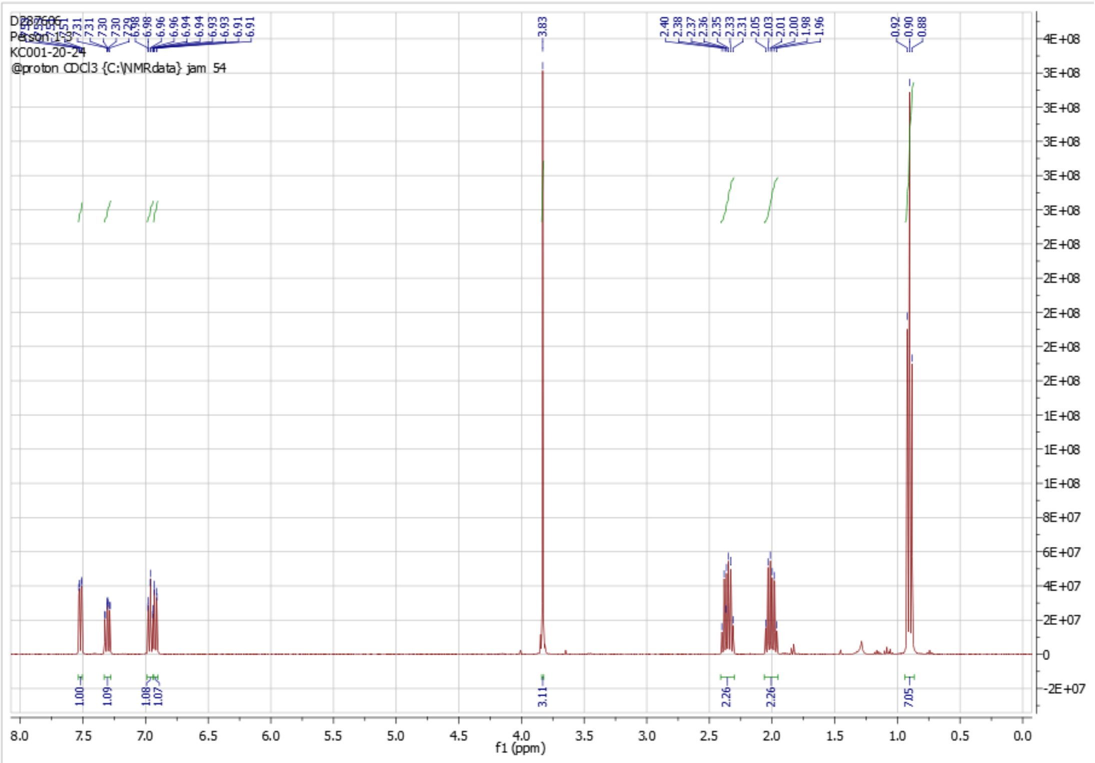
KC001-20-24
@proton CDCl3 {C:\NMRdata} jam 54
3.83
2.40 2.38 2.37 2.36 2.35 2.33 2.31 2.05 2.03 2.01 2.00 1.98 1.96
0.92 0.90 0.88
1.00
1.09
1.08
1.07
3.11
2.26
2.26
7.05
f1 (ppm)

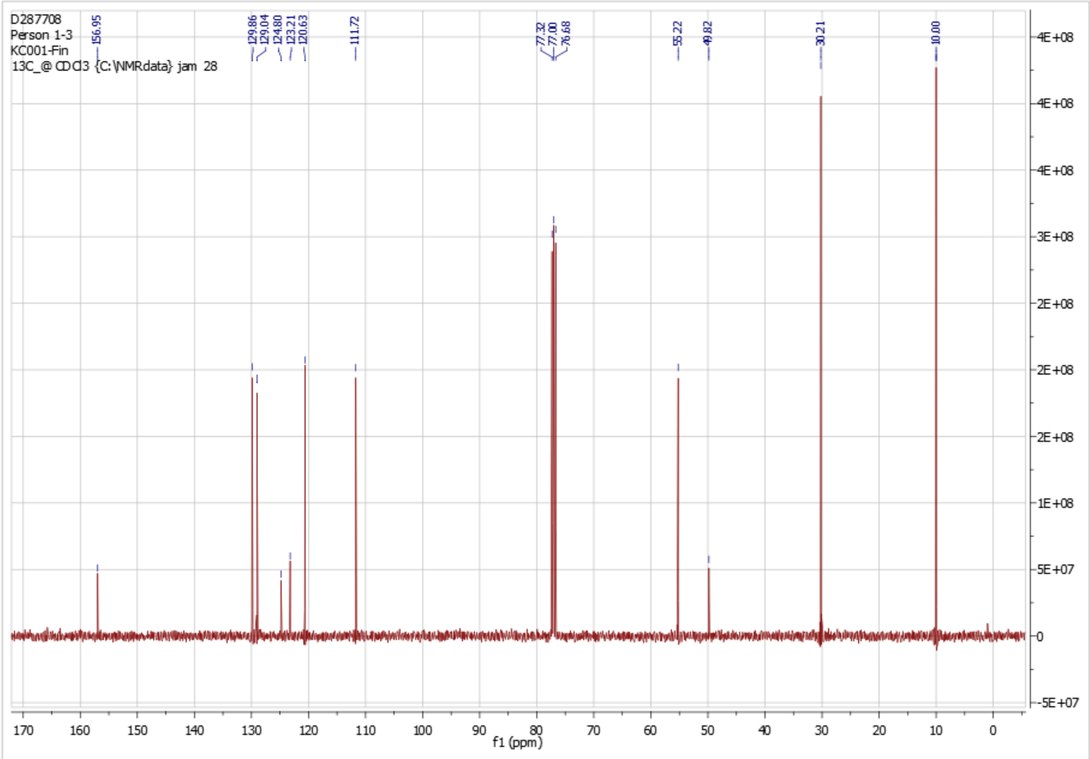
D287708
Person 1-3
KC001-Fin
13C_@ CDCl3 {C:\NMRdata} jam 28
156.95
129.86 129.04 124.80 123.21 120.63
111.72
77.32 77.00 76.68
55.22
49.82
30.21
10.00
f1 (ppm)

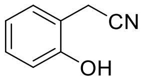
CN
OH

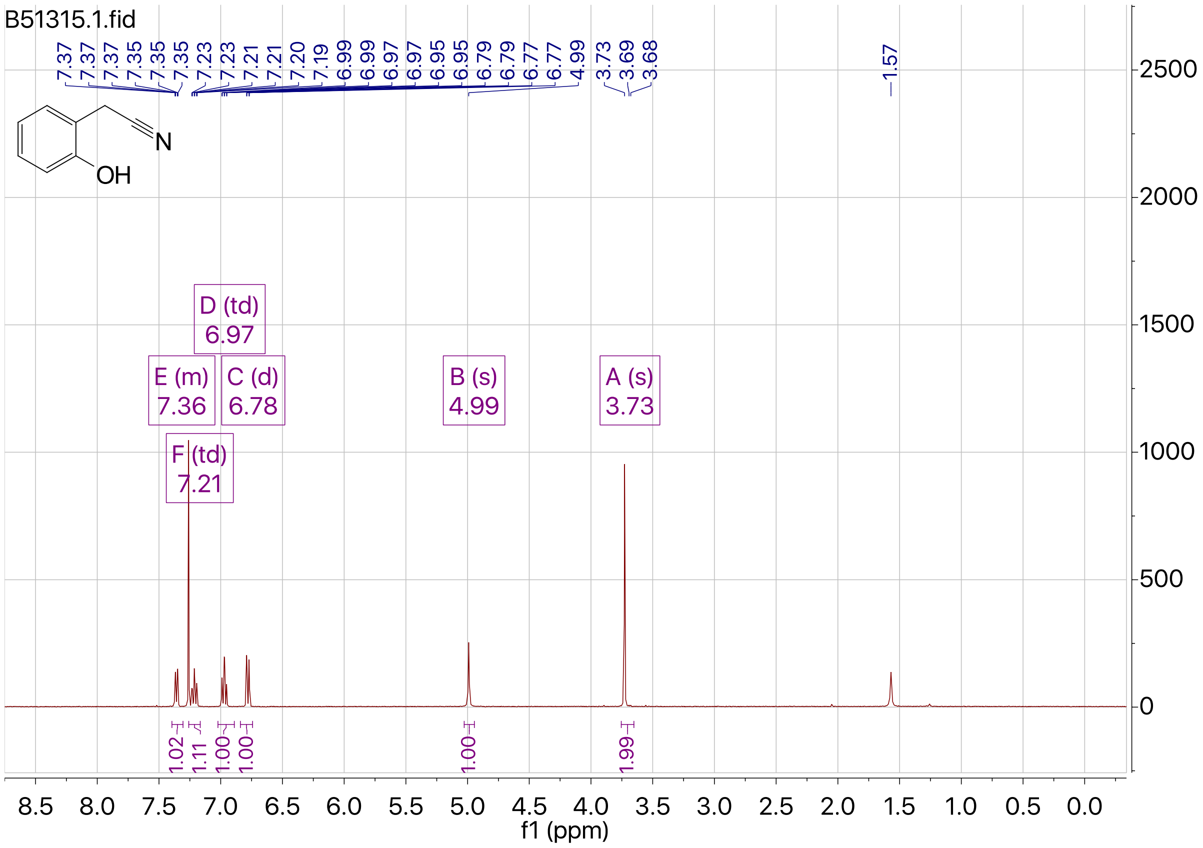
B51315.1.fid
7.37
7.37
7.37
7.35
7.35
7.35
7.23
7.23
7.21
7.21
7.20
7.19
6.99
6.99
6.97
6.97
6.95
6.95
6.79
6.79
6.77
6.77
4.99
3.73
3.69
3.68
1.57
N
OH
D (td)
6.97
E (m)
7.36
C (d)
6.78
B (s)
4.99
A (s)
3.73
F (td)
7.21
1.02
1.11
1.00
1.00
1.00
1.99
2500
2000
1500
1000
500
0
8.5 8.0 7.5 7.0 6.5 6.0 5.5 5.0 4.5 4.0 3.5 3.0 2.5 2.0 1.5 1.0 0.5 0.0
f1 (ppm)

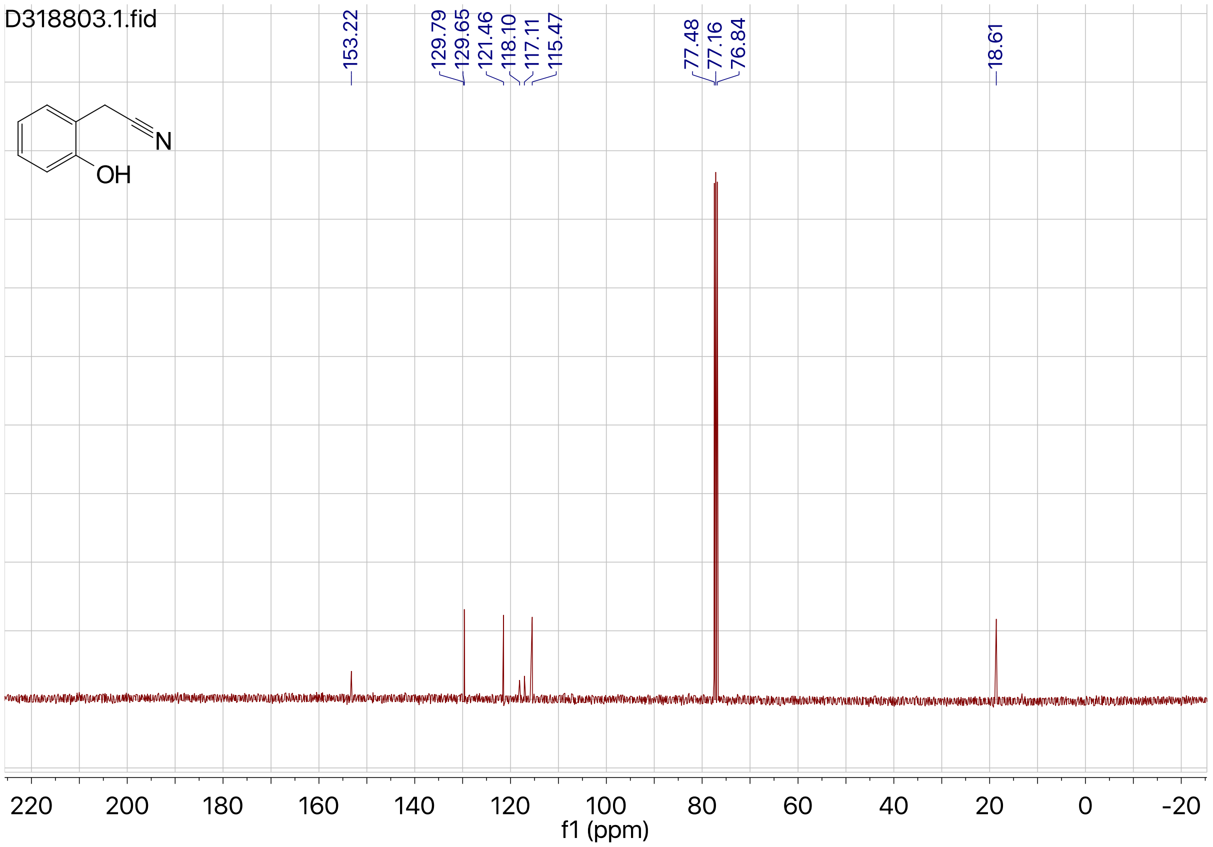
D318803.1.fid
153.22
129.79
129.65
121.46
118.10
117.11
115.47
77.48
77.16
76.84
18.61
N
OH
220 200 180 160 140 120 100 80 60 40 20 0 -20
f1 (ppm)

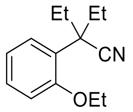

Et
Et
CN
OEt

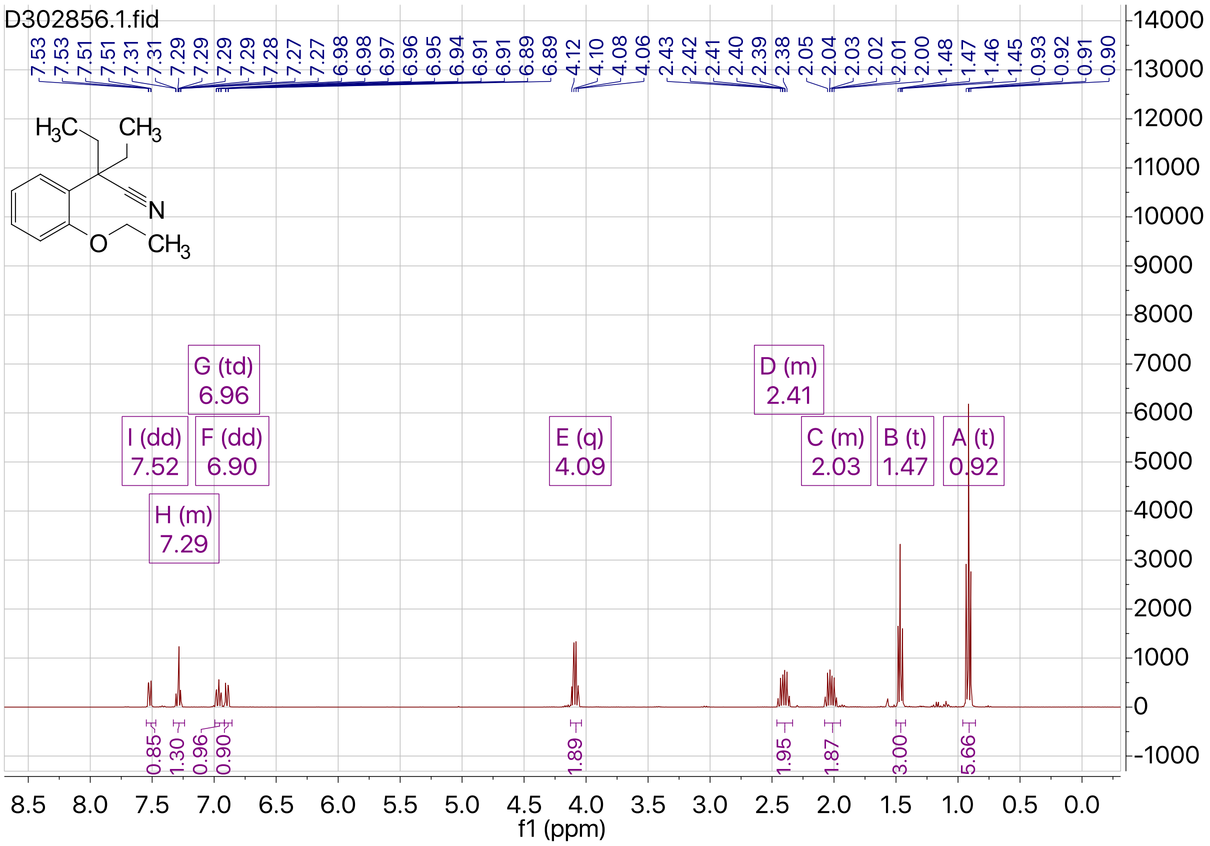

D302856.1.fid
7.53 7.53 7.51 7.51 7.31 7.31 7.29 7.29 7.29 7.29 7.28 7.27 7.27 6.98 6.98 6.97 6.96 6.95 6.94 6.91 6.91 6.89 4.12 4.10 4.08 4.06 2.43 2.42 2.41 2.40 2.39 2.38 2.05 2.04 2.03 2.02 2.01 2.00 1.48 1.47 1.46 1.45 0.93 0.92 0.91 0.90
G (td) 6.96
D (m) 2.41
I (dd) 7.52
F (dd) 6.90
E (q) 4.09
C (m) 2.03
B (t) 1.47
A (t) 0.92
H (m) 7.29
0.85 1.30 0.96 0.90 1.89 1.95 1.87 3.00 5.66
f1 (ppm)

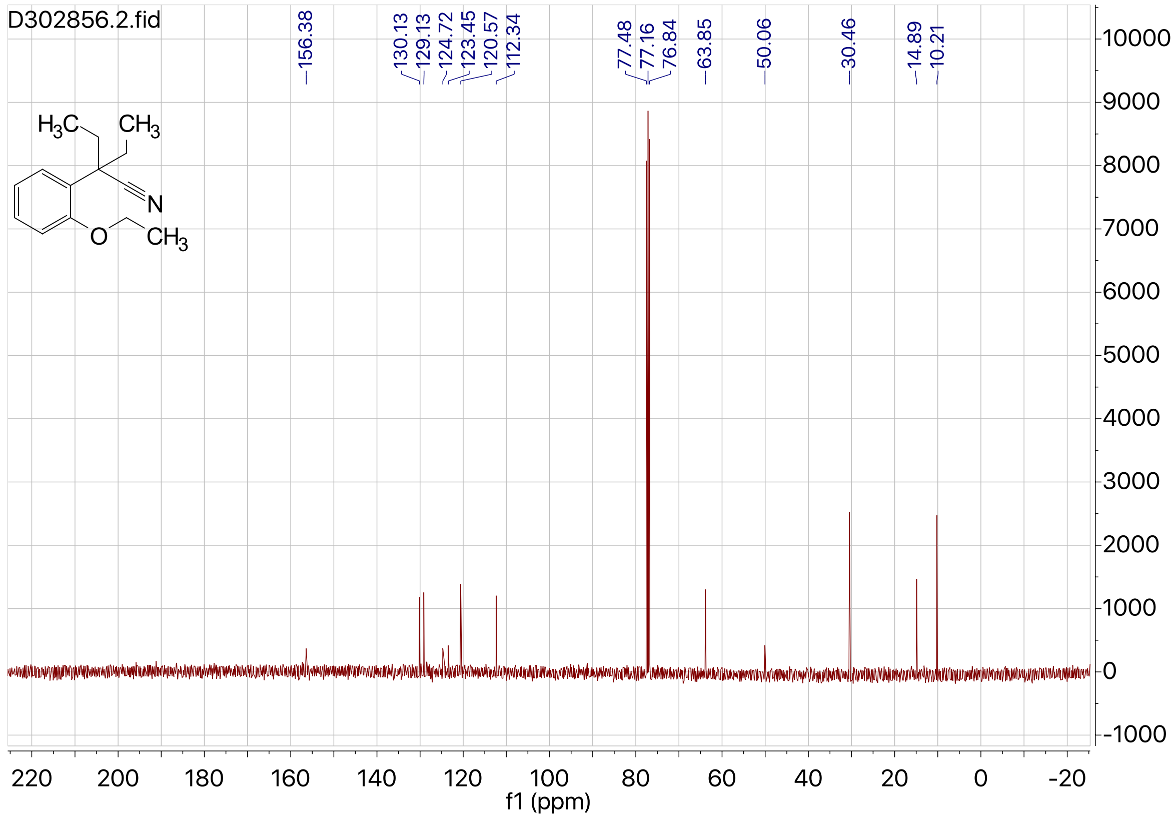

D302856.2.fid
156.38
130.13 129.13 124.72 123.45 120.57 112.34
77.48 77.16 76.84
63.85
50.06
30.46
14.89 10.21
f1 (ppm)

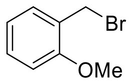

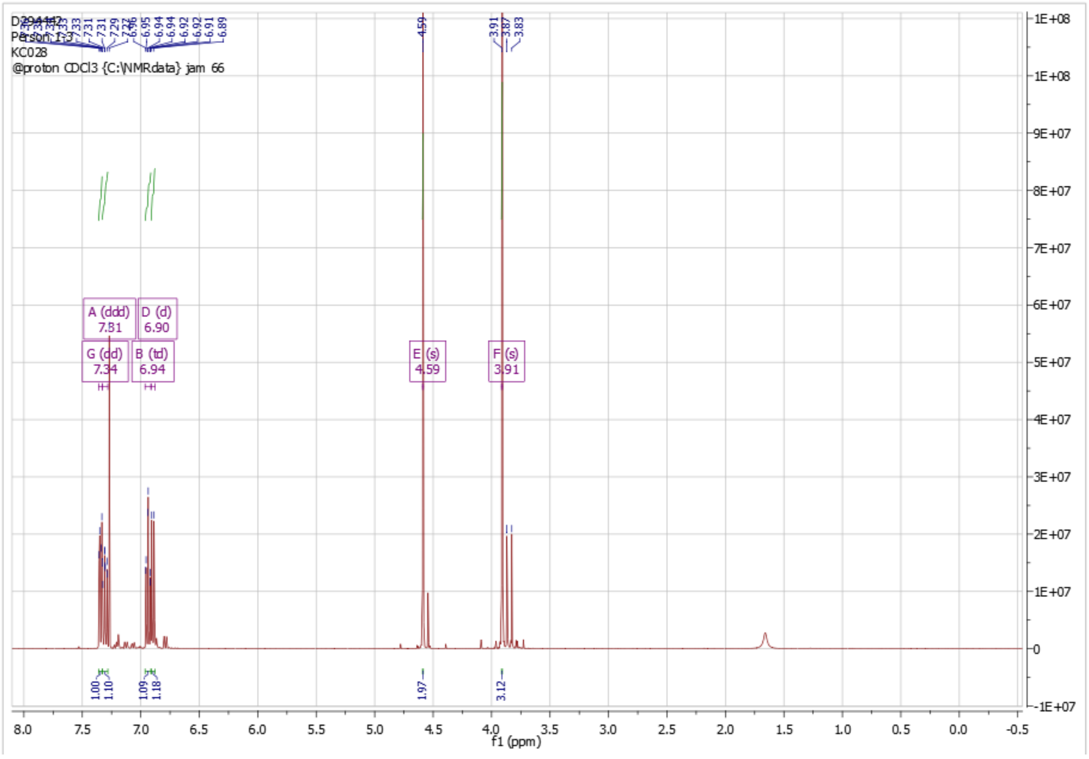
D294442
Person 1-3
KC028
@proton CDCl3 {C:\NMRdata} jam 66
A (ddd) 7.31
D (d) 6.90
G (dd) 7.34
B (td) 6.94
E (s) 4.59
F (s) 3.91
f1 (ppm)

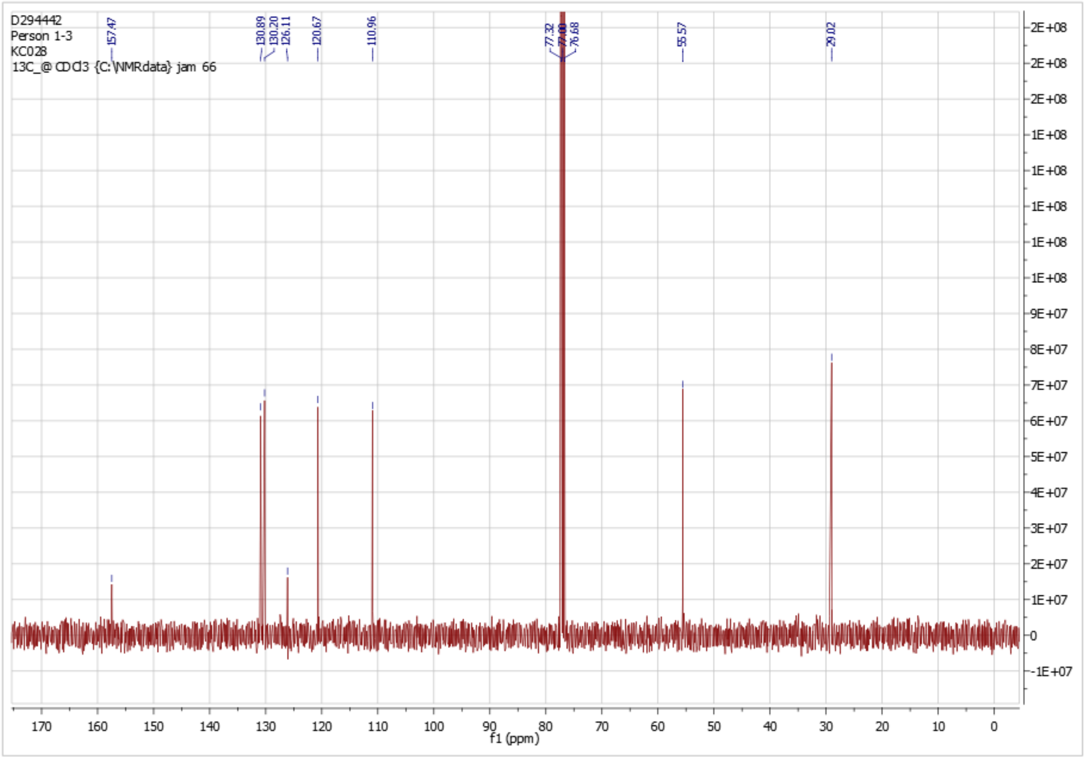
D294442
Person 1-3
KC028
13C_@ CDCl3 {C:\NMRdata} jam 66
157.47
130.89
130.20
126.11
120.67
110.96
77.32
76.68
55.57
29.02
f1 (ppm)

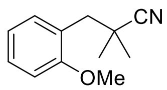
CN
OMe

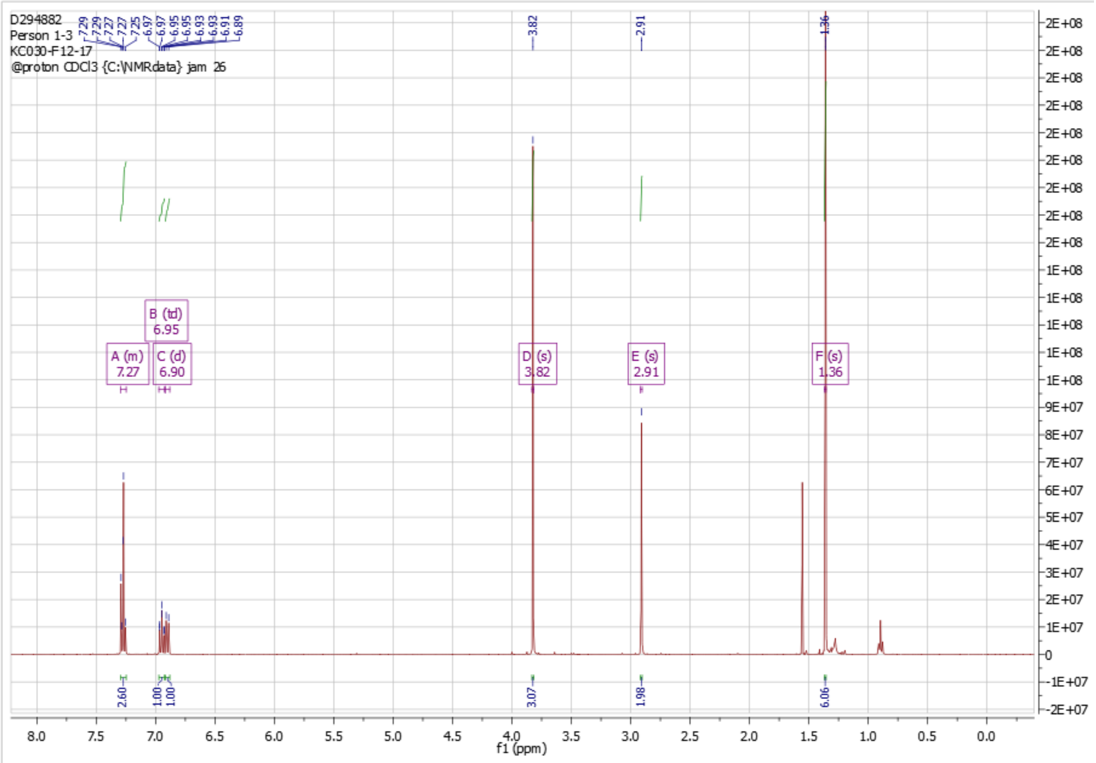
D294882
Person 1-3
KC030-F12-17
@proton CDCl3 {C:\NMRdata} jam 26
7.29 7.29 7.27 7.27 7.25 6.97 6.97 6.95 6.95 6.93 6.93 6.91 6.89
3.82
2.91
1.36
B (td) 6.95
A (m) 7.27
C (d) 6.90
D (s) 3.82
E (s) 2.91
F (s) 1.36
2.60 1.00 1.00 3.07 1.98 6.06
f1 (ppm)

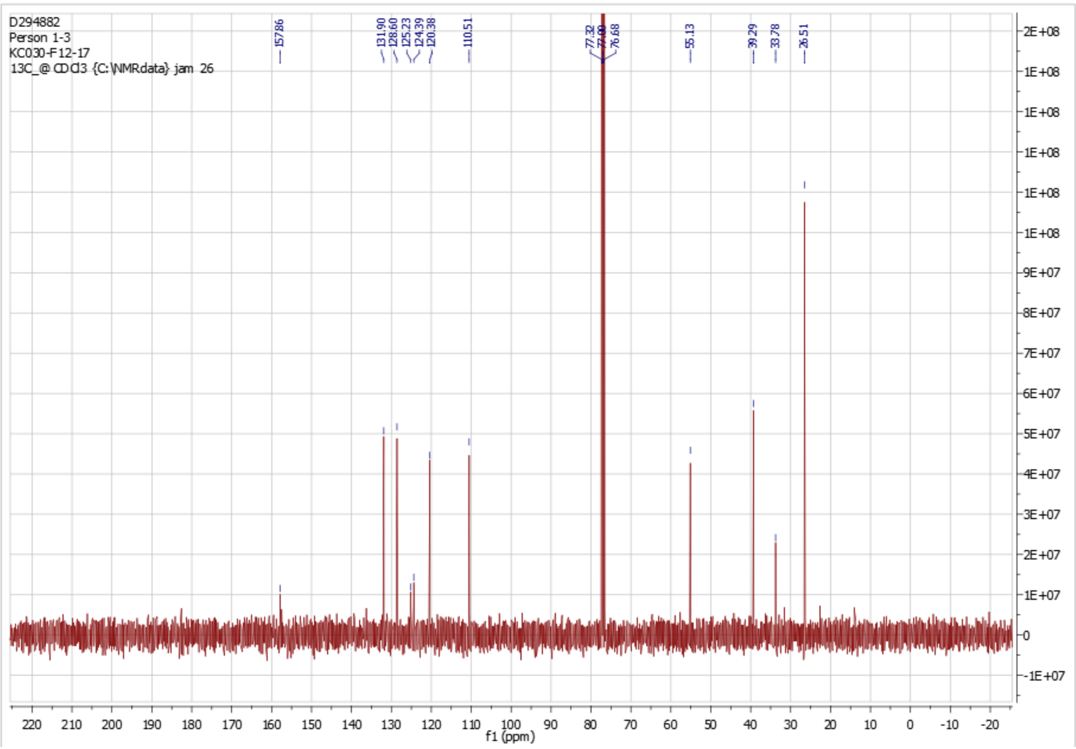
D294882
Person 1-3
KC030-F12-17
13C_@ CDCl3 {C:\NMRdata} jam 26
157.86
131.90 128.60 125.23 124.39 120.38
110.51
77.32 77.00 76.68
55.13
39.29 33.78
26.51
f1 (ppm)

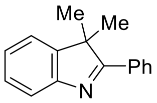

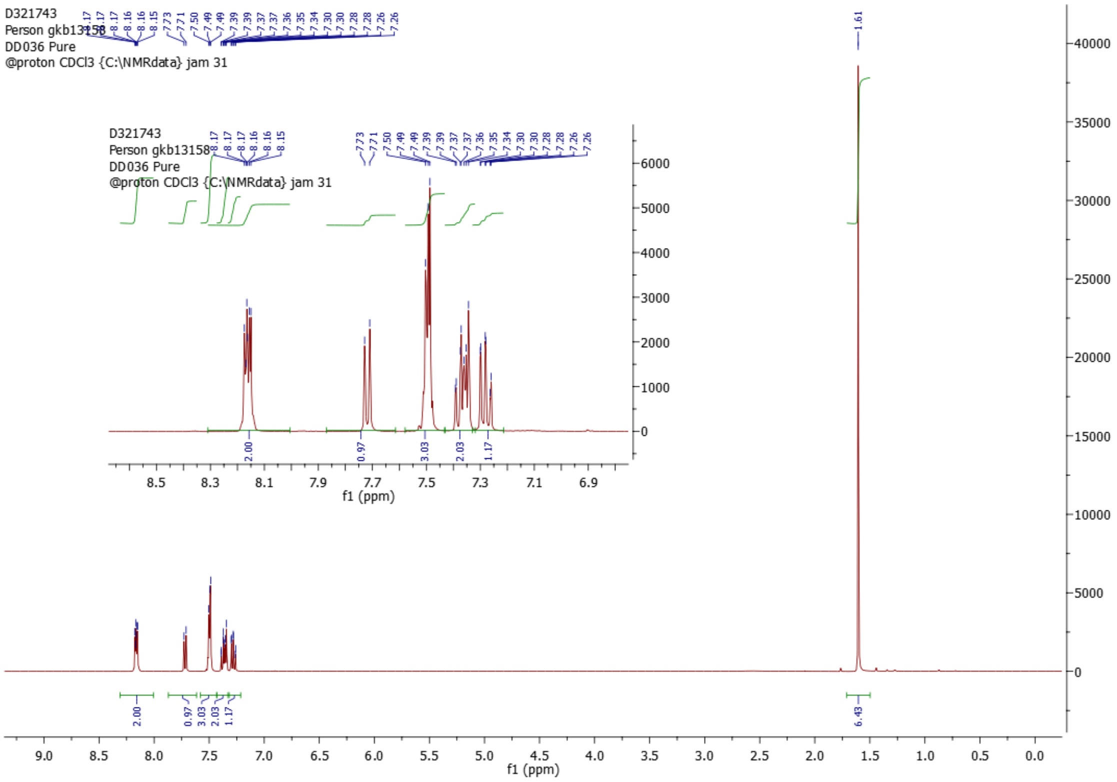

D321743
Person gkb13158
DD036 Pure
@proton CDCl3 {C:\NMRdata} jam 31
f1 (ppm)

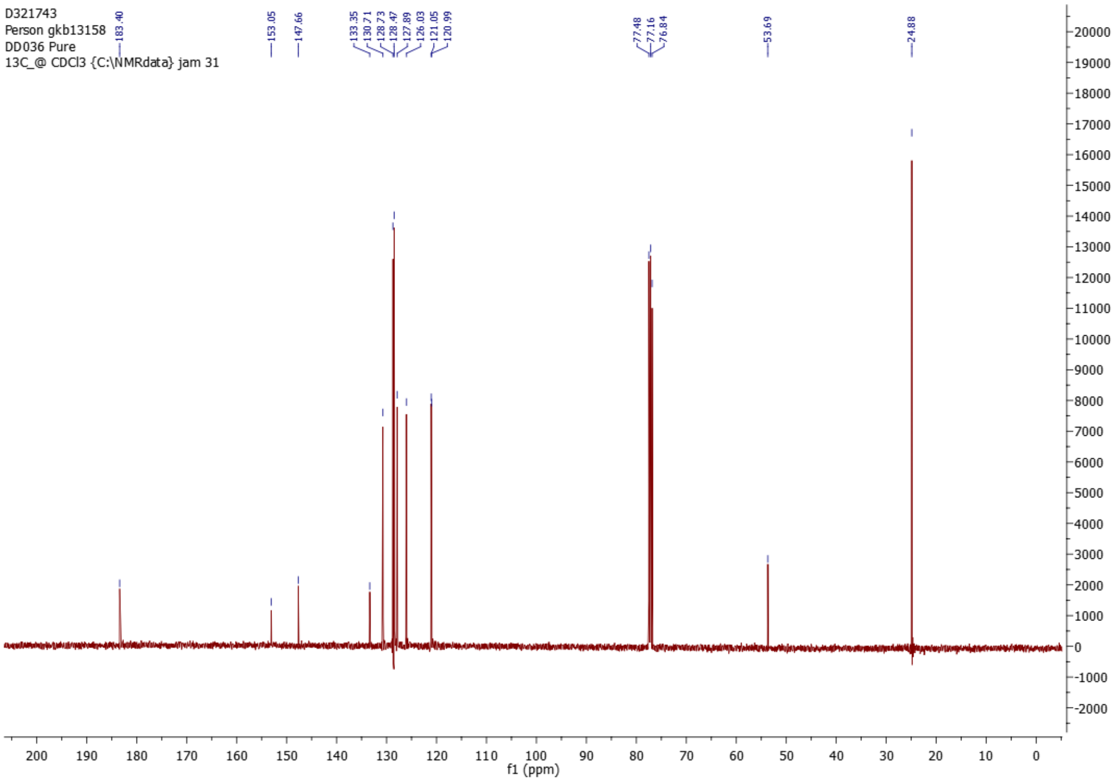

D321743
Person gkb13158
DD036 Pure
13C_@ CDCl3 {C:\NMRdata} jam 31
f1 (ppm)

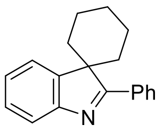
Ph
N

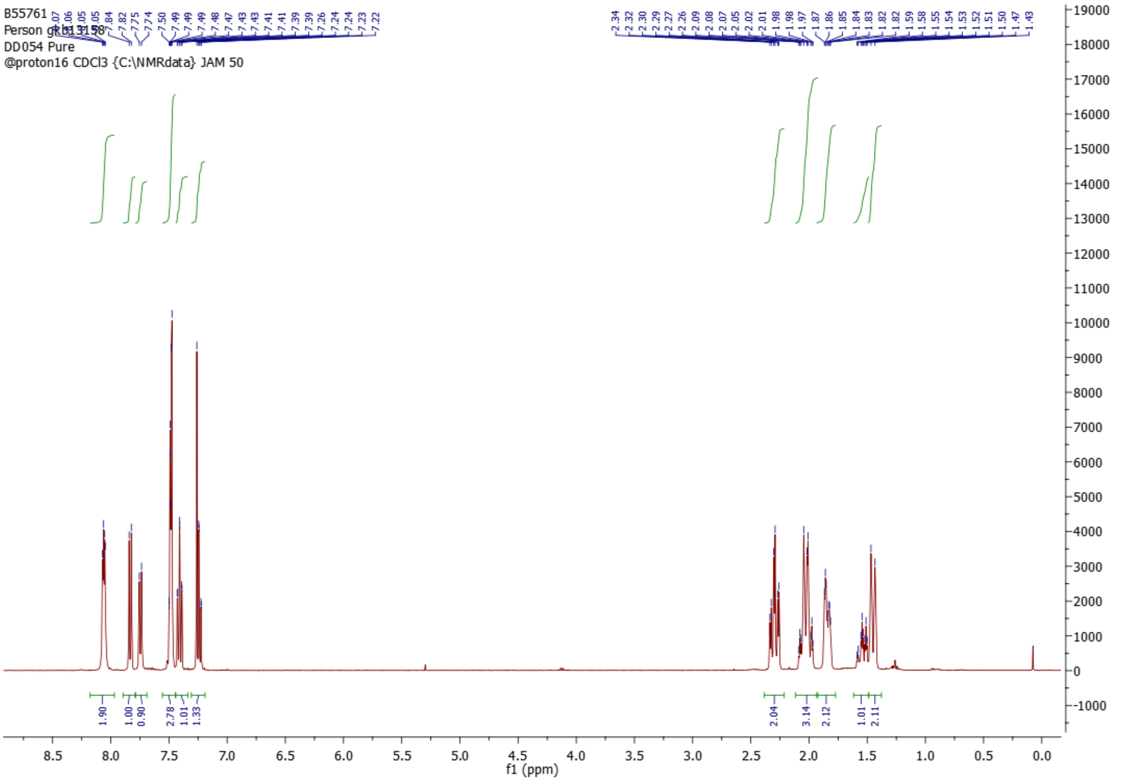
B55761
Person gkb13158
DD054 Pure
@proton16 CDCl3 {C:\NMRdata} JAM 50
8.07 8.06 8.05 8.05 7.84 7.82 7.75 7.74 7.50 7.49 7.49 7.49 7.48 7.47 7.43 7.43 7.41 7.41 7.39 7.26 7.24 7.24 7.23 7.22
2.34 2.32 2.30 2.29 2.27 2.26 2.09 2.08 2.07 2.05 2.02 2.01 1.98 1.98 1.97 1.87 1.86 1.85 1.84 1.83 1.82 1.82 1.59 1.58 1.55 1.54 1.53 1.52 1.51 1.50 1.47 1.43
1.90 1.00 0.90 2.78 1.01 1.33 2.04 3.14 2.12 1.01 2.11
f1 (ppm)

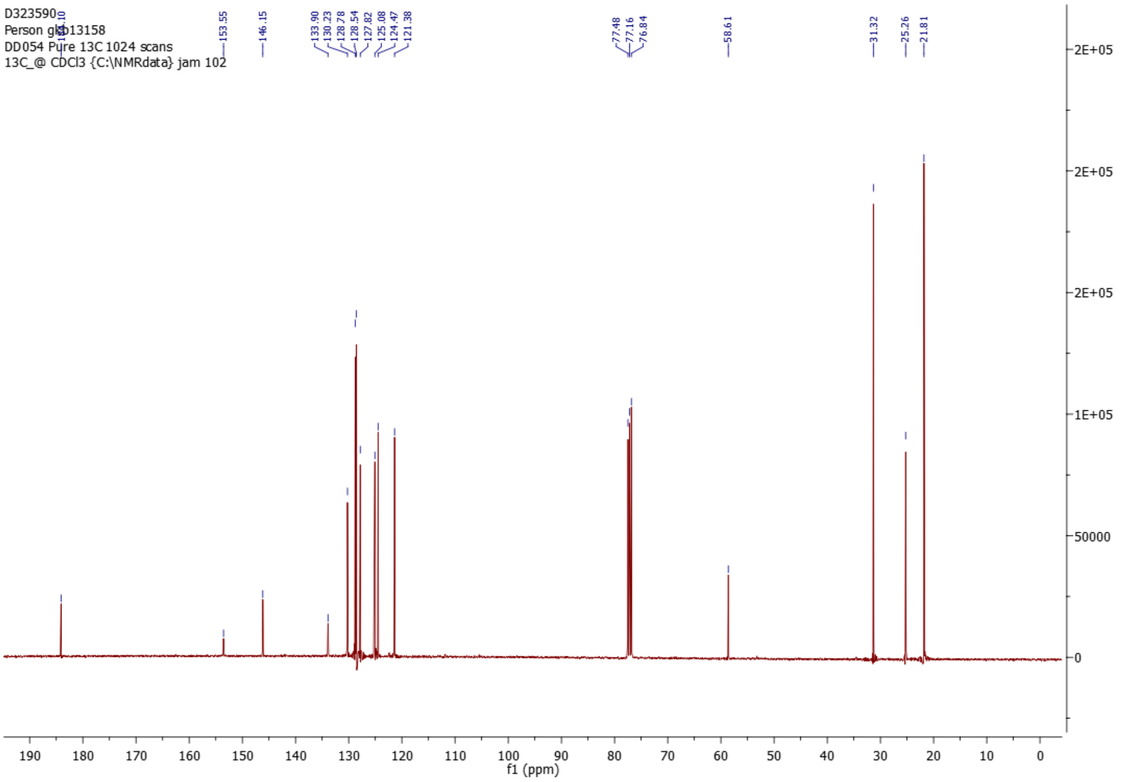
D323590
Person gkb13158
DD054 Pure 13C 1024 scans
13C_@ CDCl3 {C:\NMRdata} jam 102
184.10 153.55 146.15 133.90 130.23 128.78 128.54 127.82 125.08 124.47 121.38 77.48 77.16 76.84 58.61 31.32 25.26 21.81
f1 (ppm)

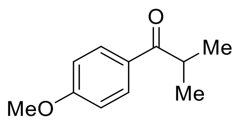
O
Me
Me
MeO

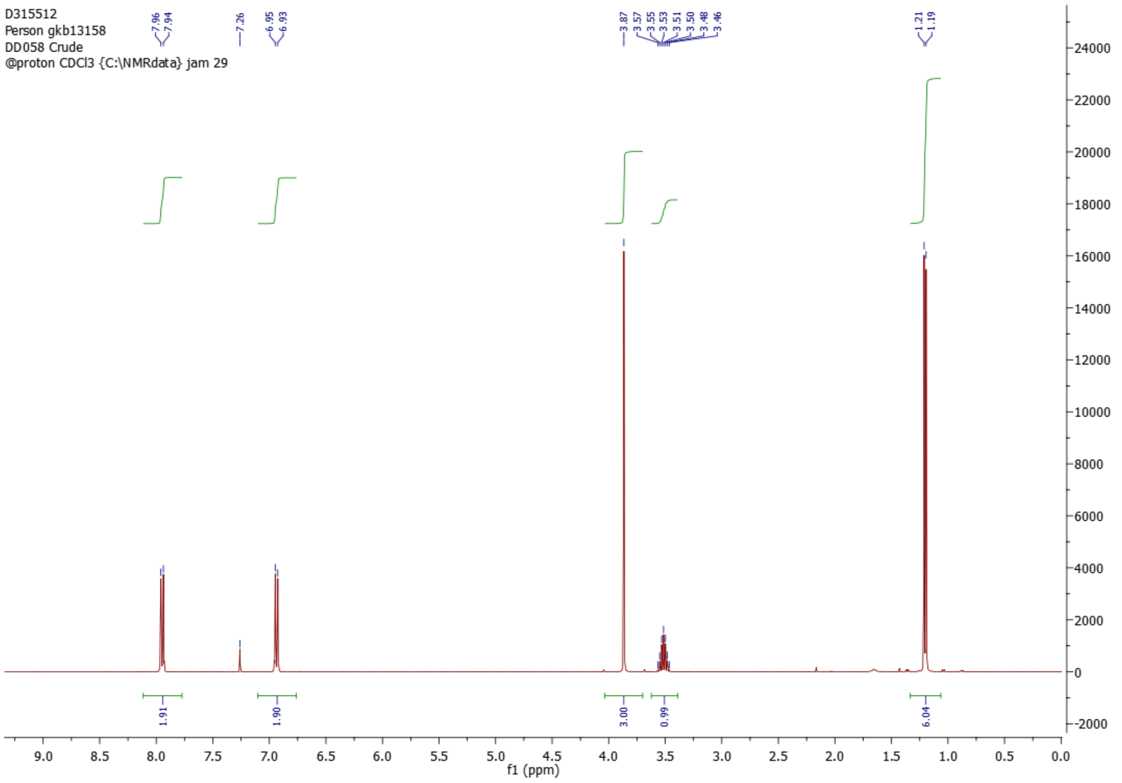
D315512
Person gkb13158
DD058 Crude
@proton CDCl3 {C:\NMRdata} jam 29
f1 (ppm)

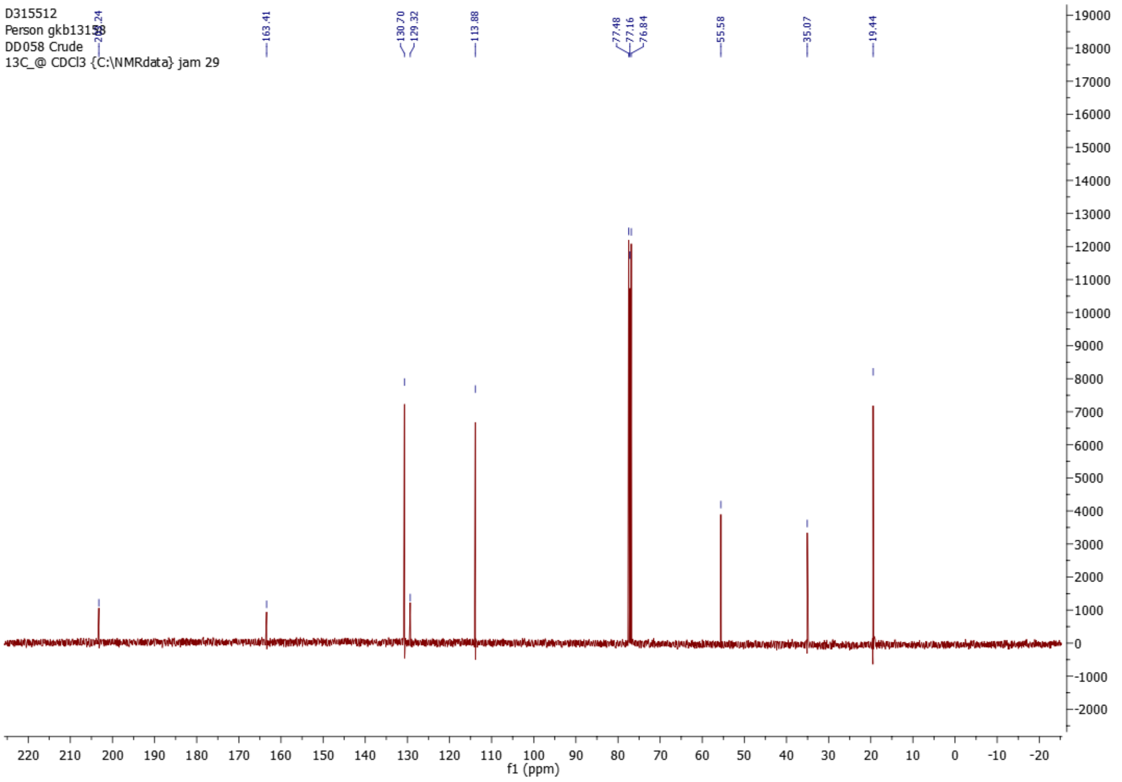
D315512
Person gkb13158
DD058 Crude
13C_@ CDCl3 {C:\NMRdata} jam 29
f1 (ppm)

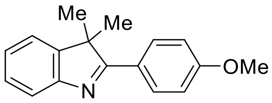
Me
Me
OMe
N

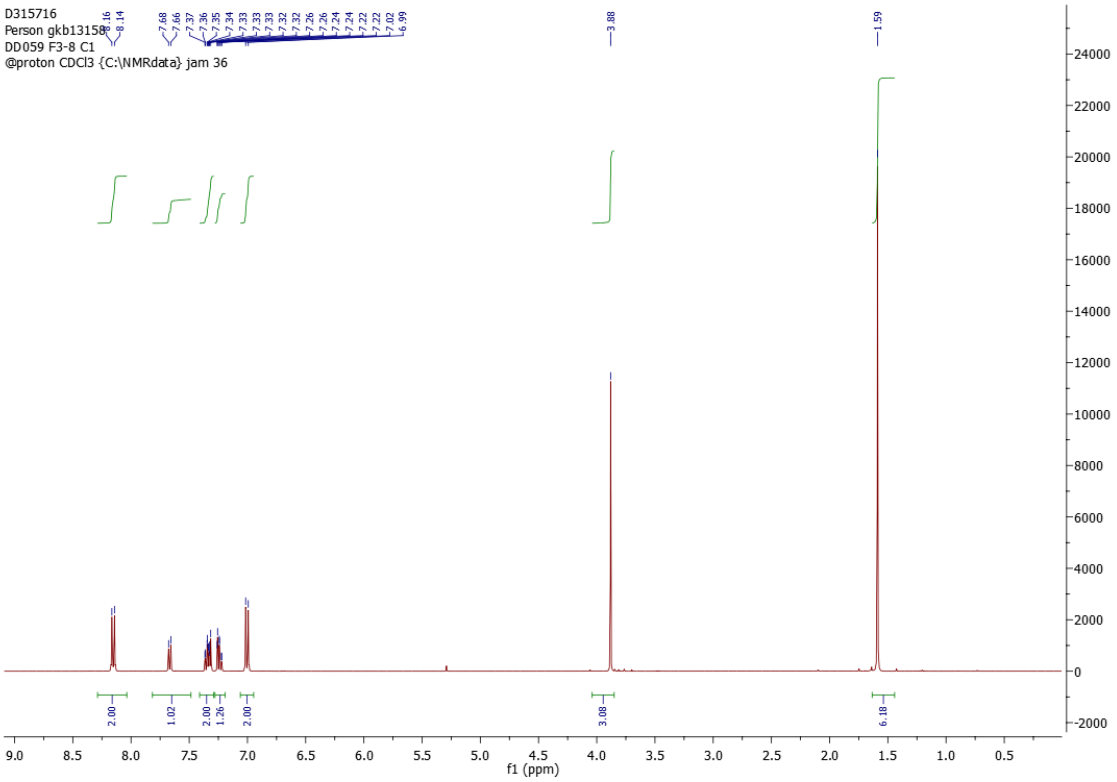
D315716
Person gkb13158
DD059 F3-8 C1
@proton CDCl3 {C:\NMRdata} jam 36
8.16
8.14
7.68
7.66
7.37
7.36
7.35
7.34
7.33
7.33
7.33
7.32
7.32
7.26
7.26
7.24
7.24
7.22
7.22
7.02
6.99
3.88
1.59
2.00
1.02
2.00
1.26
2.00
3.08
6.18
f1 (ppm)

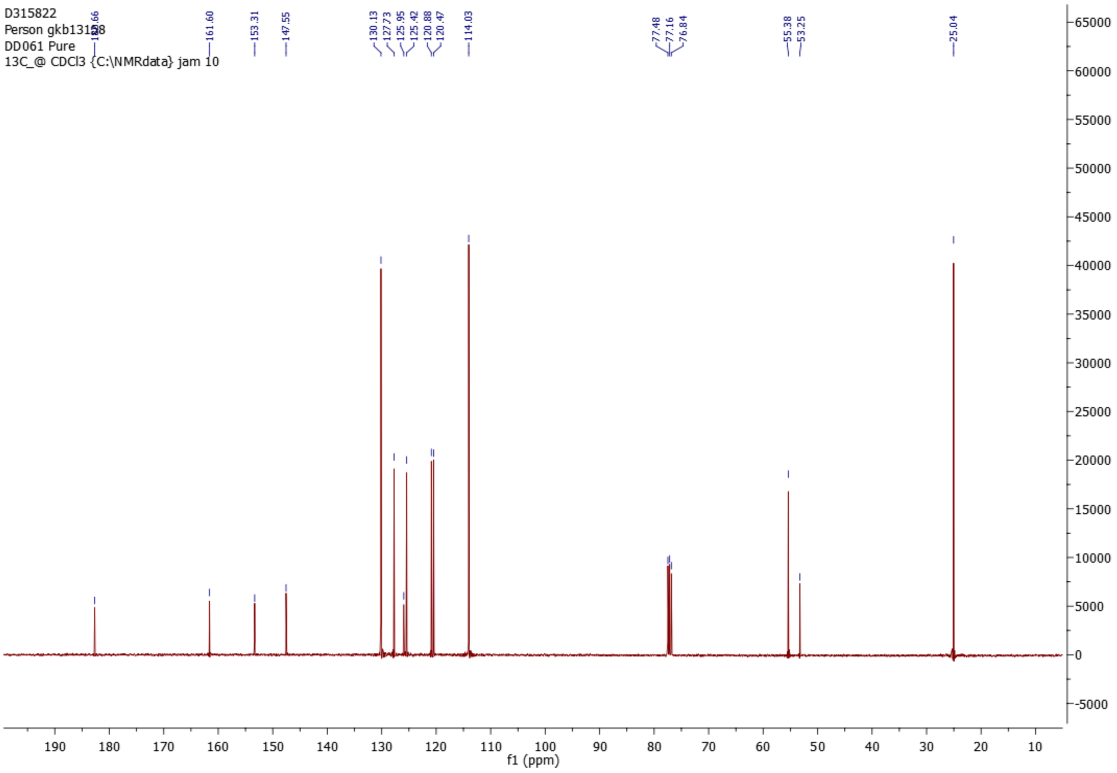
D315822
Person gkb13158
DD061 Pure
13C_@ CDCl3 {C:\NMRdata} jam 10
182.66
161.60
153.31
147.55
130.13
127.73
125.95
125.42
120.88
120.47
114.03
77.48
77.16
76.84
55.38
53.25
25.04
f1 (ppm)

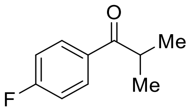
O
Me
Me
F

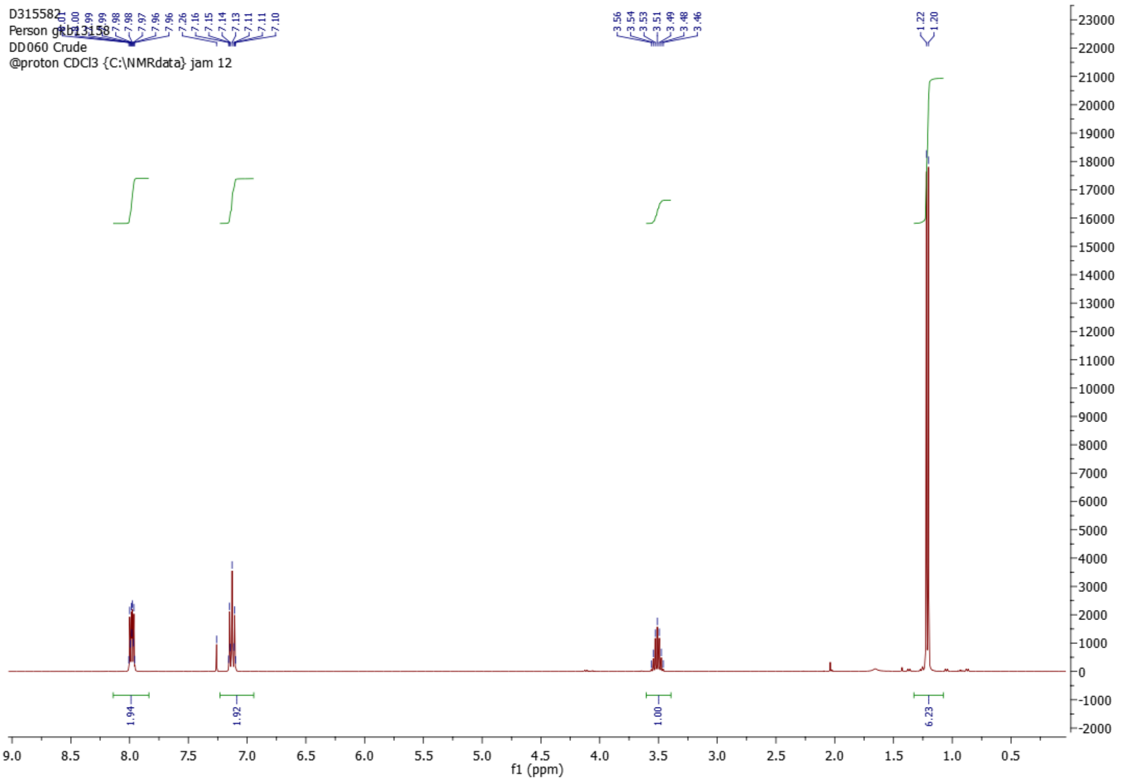
D315582
Person gkb13158
DD060 Crude
@proton CDCl3 {C:\NMRdata} jam 12
8.00 7.99 7.99 7.98 7.98 7.97 7.96 7.96 7.26 7.16 7.15 7.14 7.13 7.11 7.10
3.56 3.54 3.53 3.51 3.49 3.48 3.46
1.22 1.20
1.94
1.92
1.00
6.23
f1 (ppm)

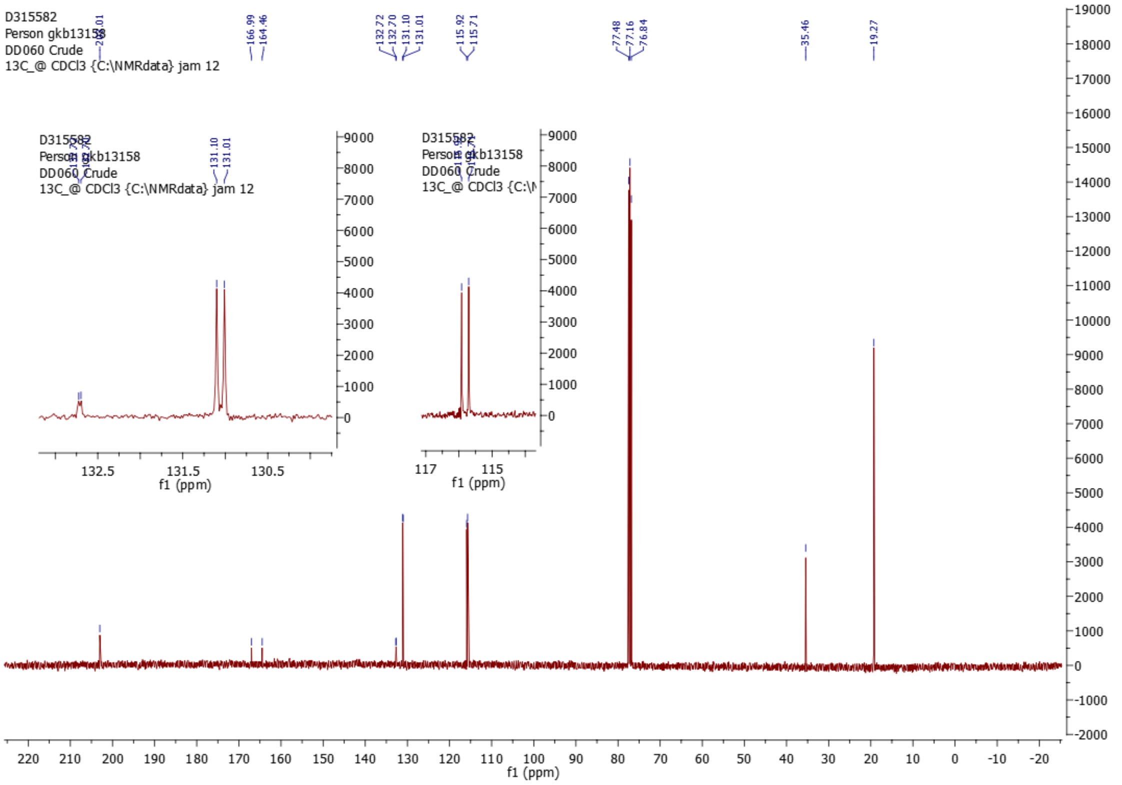
D315582
Person gkb13158
DD060 Crude
13C_@ CDCl3 {C:\NMRdata} jam 12
166.99 164.46
132.72 132.70 131.10 131.01
115.92 115.71
77.48 77.16 76.84
35.46
19.27
f1 (ppm)

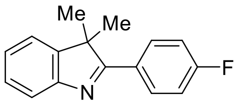
Me
Me
N
F

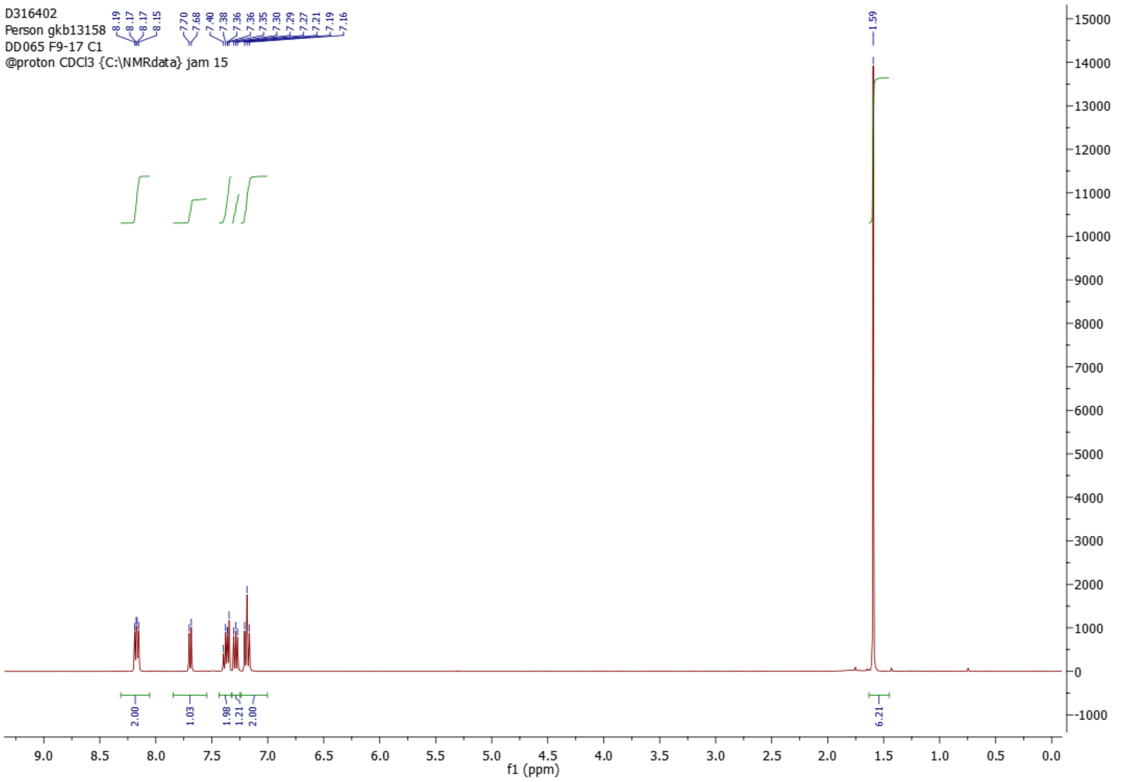
D316402
Person gkb13158
DD065 F9-17 C1
@proton CDCl3 {C:\NMRdata} jam 15
8.19 8.17 8.17 8.15
7.70 7.68
7.40 7.38 7.36 7.36 7.35 7.30 7.29 7.27 7.21 7.19 7.16
1.59
2.00
1.03
1.98
1.21
2.00
6.21
f1 (ppm)

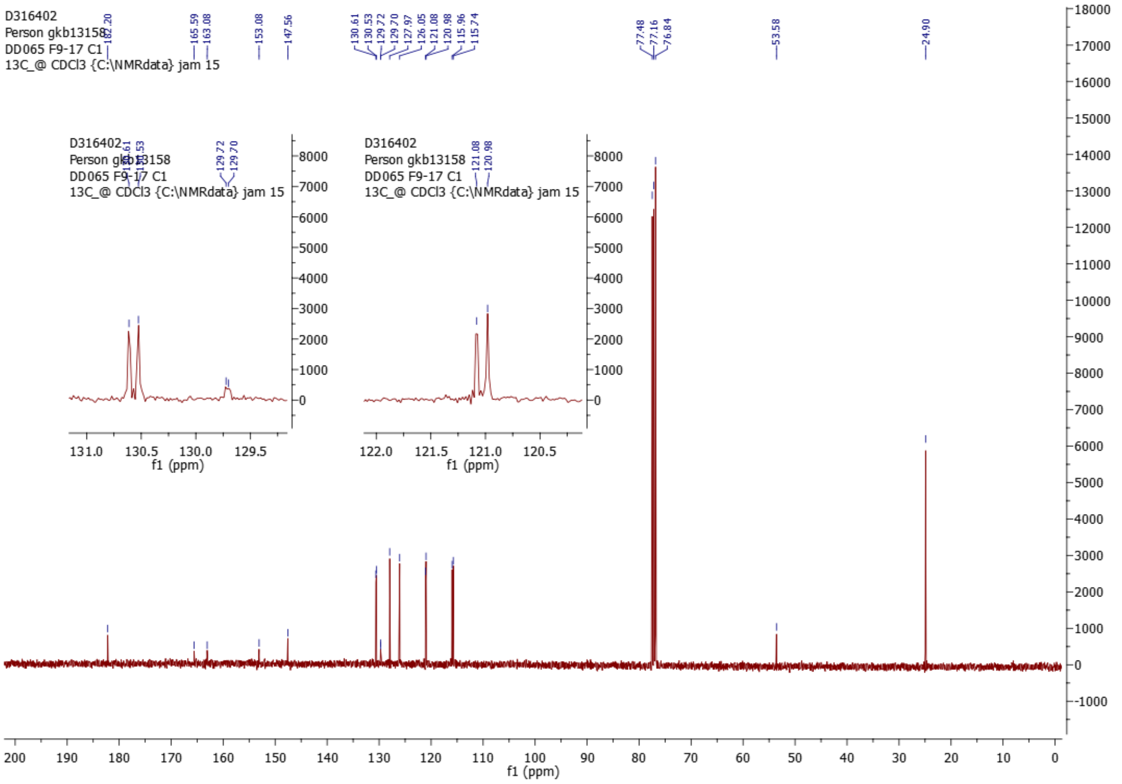
D316402
Person gkb13158
DD065 F9-17 C1
13C_@ CDCl3 {C:\NMRdata} jam 15
182.20
165.59 163.08
153.08
147.56
130.61 130.53 129.72 129.70 127.97 126.05 121.08 120.98 115.96 115.74
77.48 77.16 76.84
53.58
24.90
f1 (ppm)

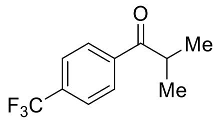

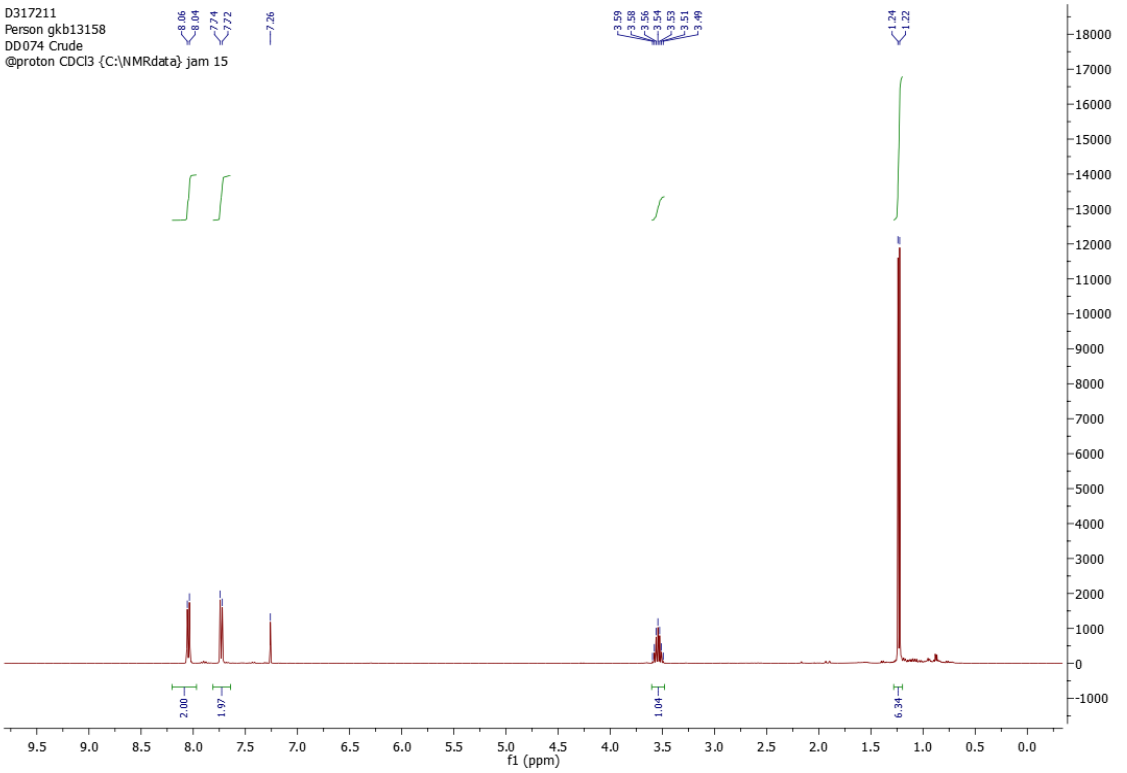
D317211
Person gkb13158
DD074 Crude
@proton CDCl3 {C:\NMRdata} jam 15
8.06
8.04
7.74
7.72
7.26
3.59
3.58
3.56
3.54
3.53
3.51
3.49
1.24
1.22
2.00
1.97
1.04
6.34
f1 (ppm)

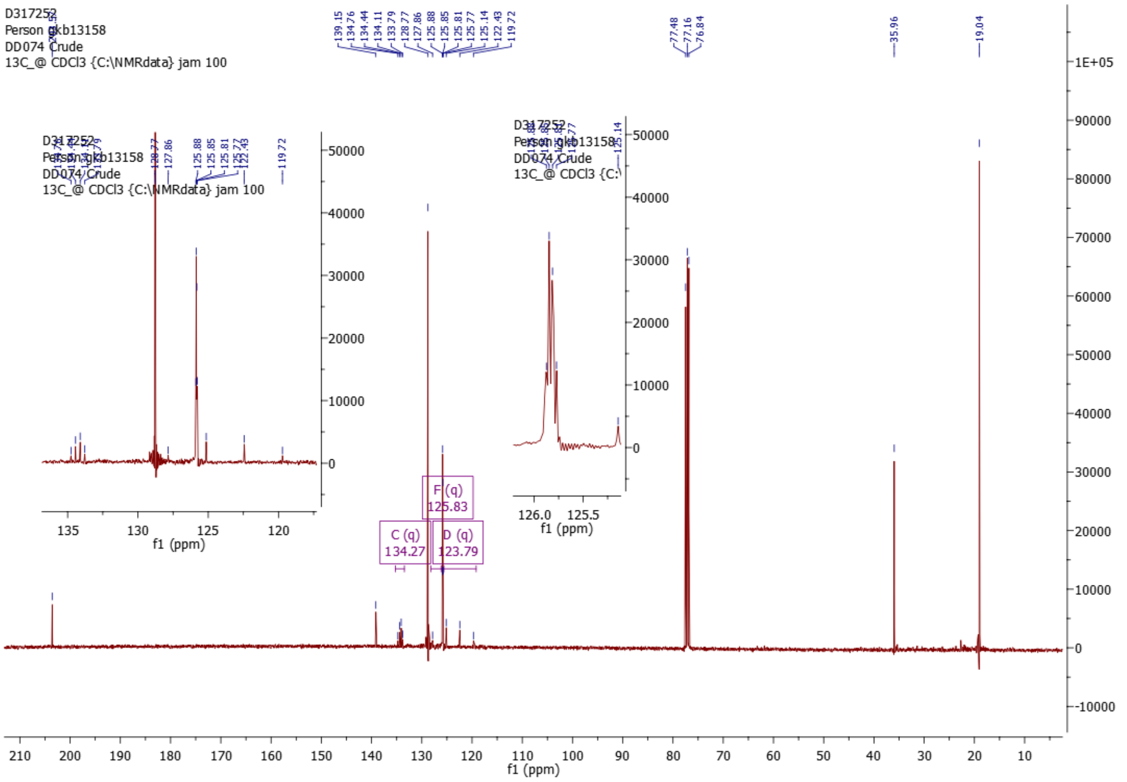
D317252
Person gkb13158
DD074 Crude
13C_@ CDCl3 {C:\NMRdata} jam 100
139.15
134.76
134.44
134.11
133.79
128.77
127.86
125.88
125.85
125.81
125.77
125.14
122.43
119.72
77.48
77.16
76.84
35.96
19.04
F (q)
125.83
C (q)
134.27
D (q)
123.79
f1 (ppm)

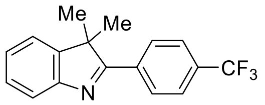
Me
Me
CF3
N

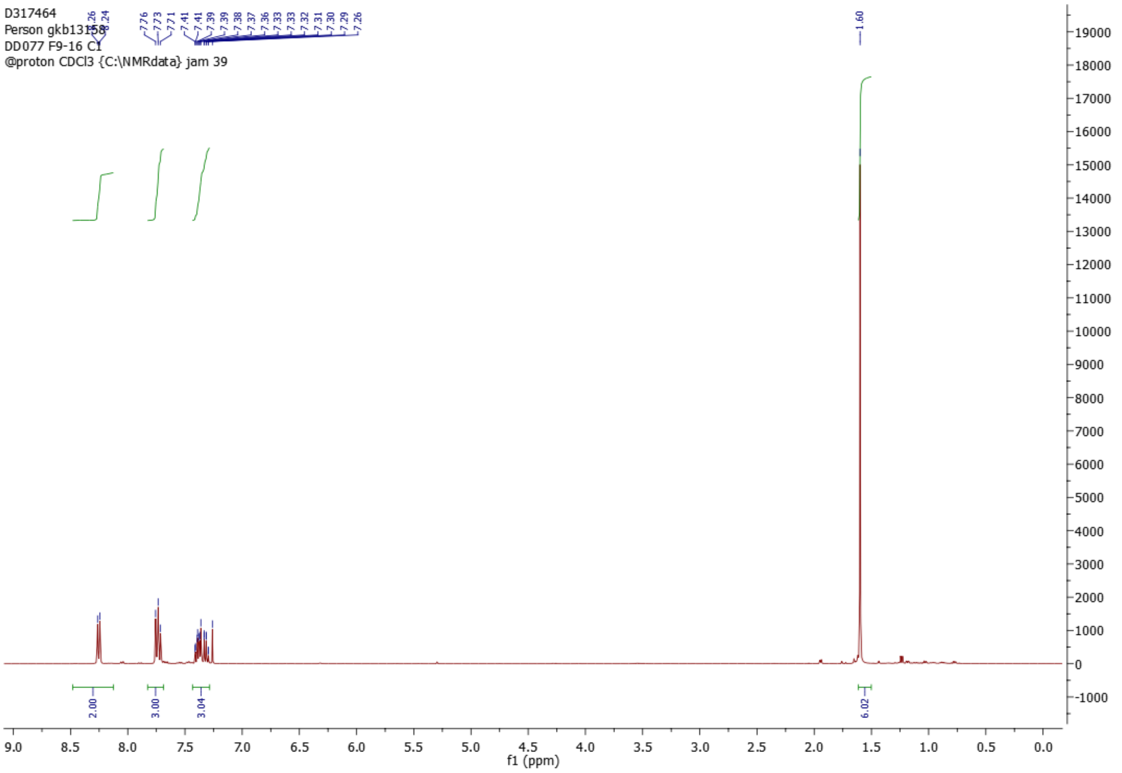
D317464
Person gkb13158
DD077 F9-16 C1
@proton CDCl3 {C:\NMRdata} jam 39
f1 (ppm)

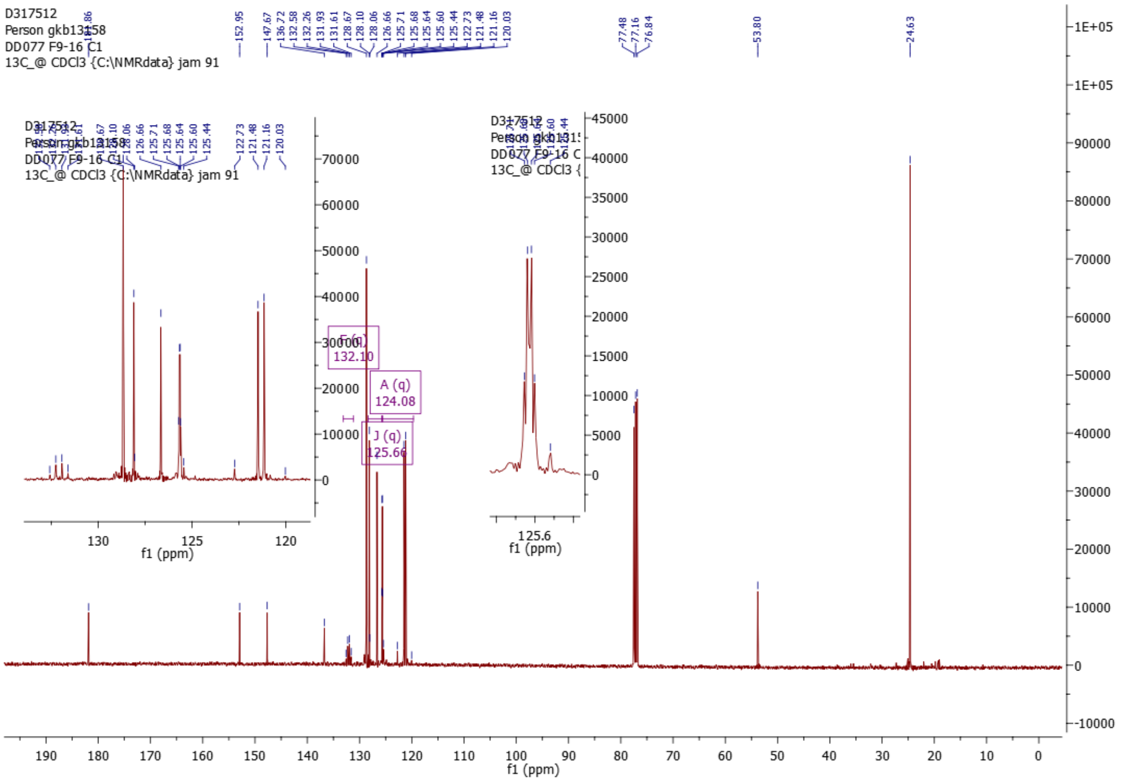
D317512
Person gkb13158
DD077 F9-16 C1
13C_@ CDCl3 {C:\NMRdata} jam 91
F (q)
132.10
A (q)
124.08
J (q)
125.66
f1 (ppm)

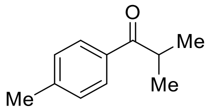
O
Me
Me
Me

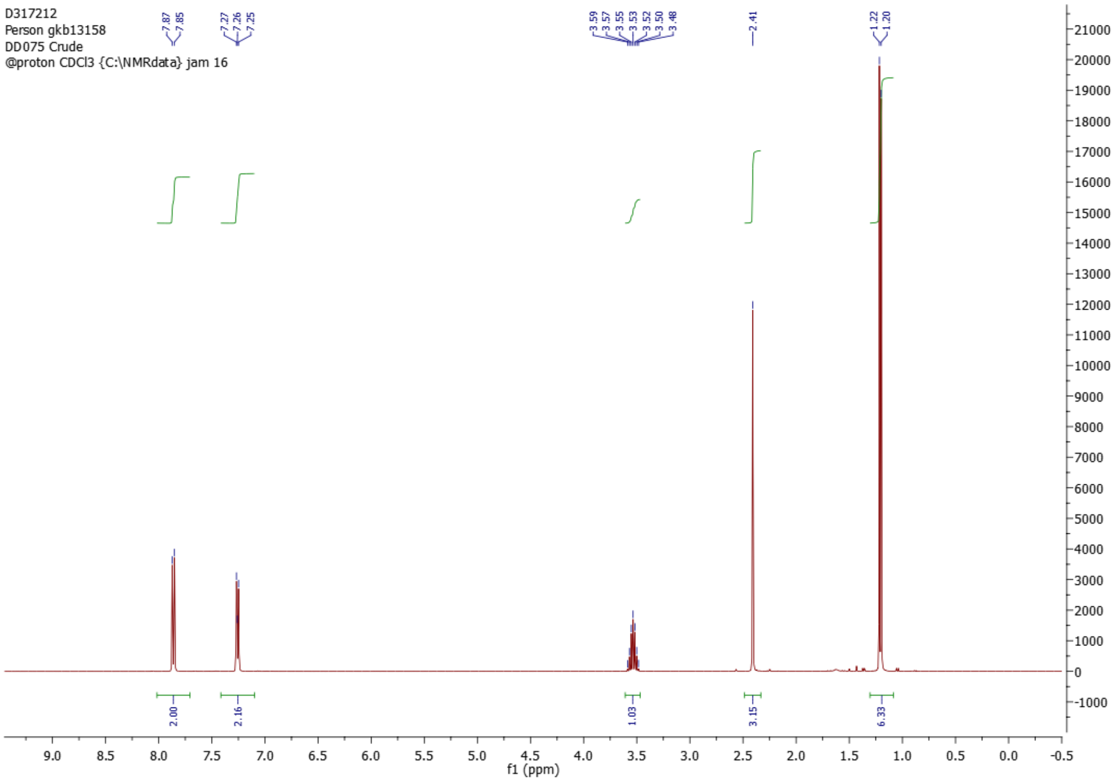
D317212
Person gkb13158
DD075 Crude
@proton CDCl3 {C:\NMRdata} jam 16
7.87
7.85
7.27
7.26
7.25
3.59
3.57
3.55
3.53
3.52
3.50
3.48
2.41
1.22
1.20
2.00
2.16
1.03
3.15
6.33
f1 (ppm)

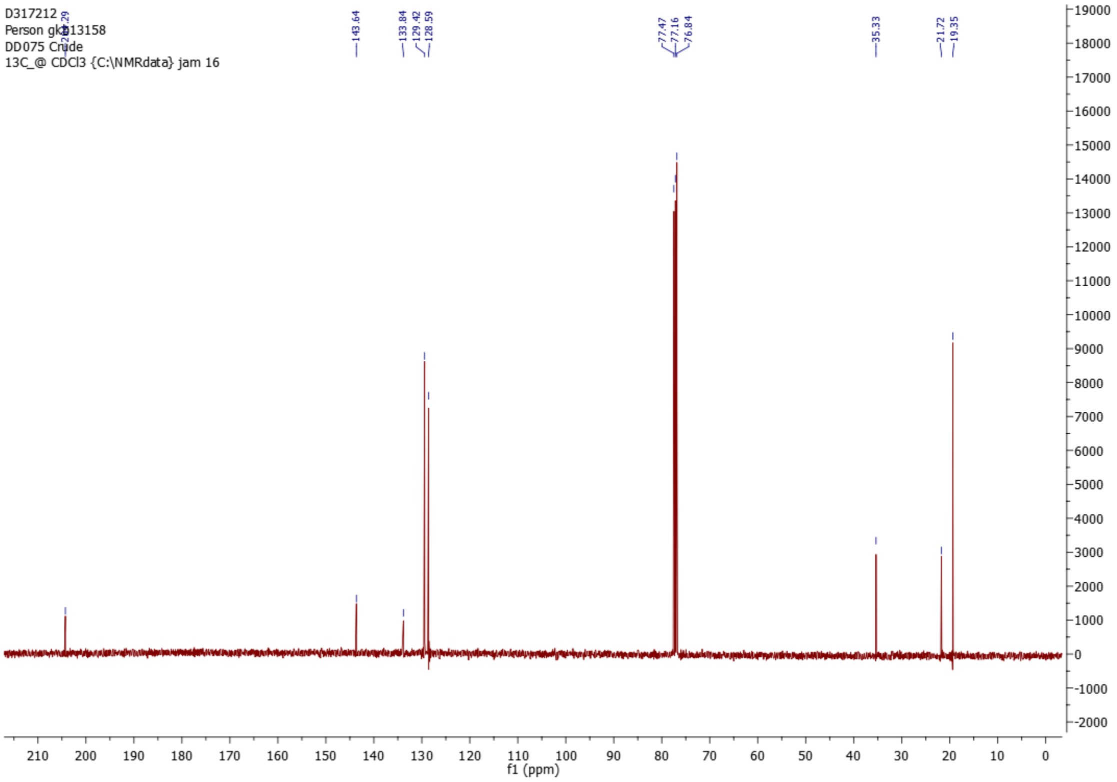
D317212
Person gkb13158
DD075 Crude
13C_@ CDCl3 {C:\NMRdata} jam 16
143.64
133.84
129.42
128.59
77.47
77.16
76.84
35.33
21.72
19.35
f1 (ppm)

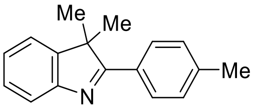

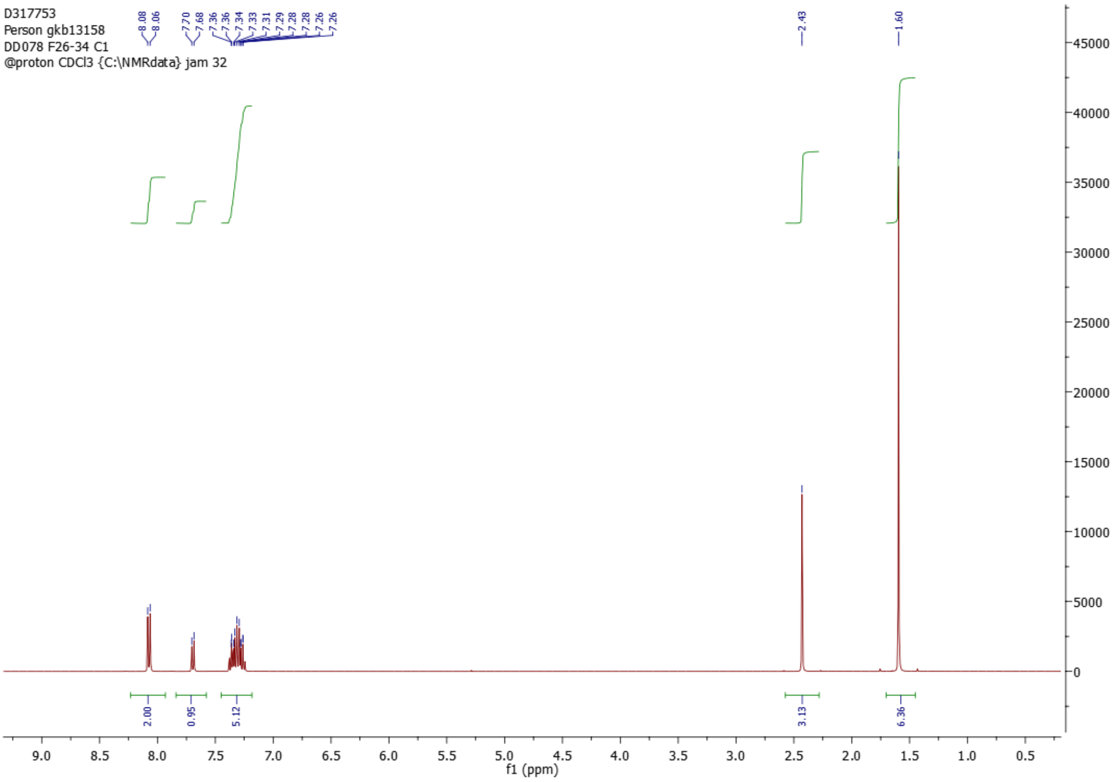

D317753
Person gkb13158
DD078 F26-34 C1
@proton CDCl3 {C:\NMRdata} jam 32
8.08
8.06
7.70
7.68
7.36
7.36
7.34
7.33
7.31
7.29
7.28
7.28
7.26
7.26
2.43
1.60
2.00
0.95
5.12
3.13
6.36
f1 (ppm)

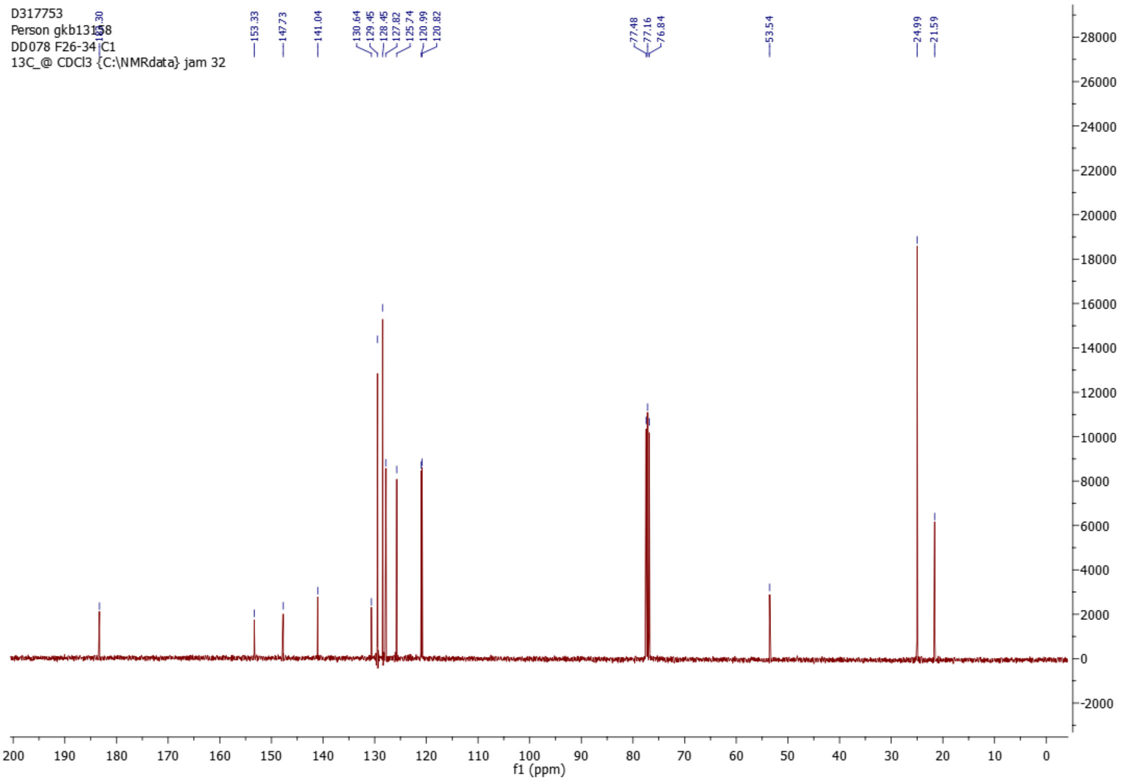

D317753
Person gkb13158
DD078 F26-34 C1
13C_@ CDCl3 {C:\NMRdata} jam 32
183.30
153.33
147.73
141.04
130.64
129.45
128.45
127.82
125.74
120.99
120.82
77.48
77.16
76.84
53.54
24.99
21.59
f1 (ppm)

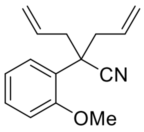
CN
OMe

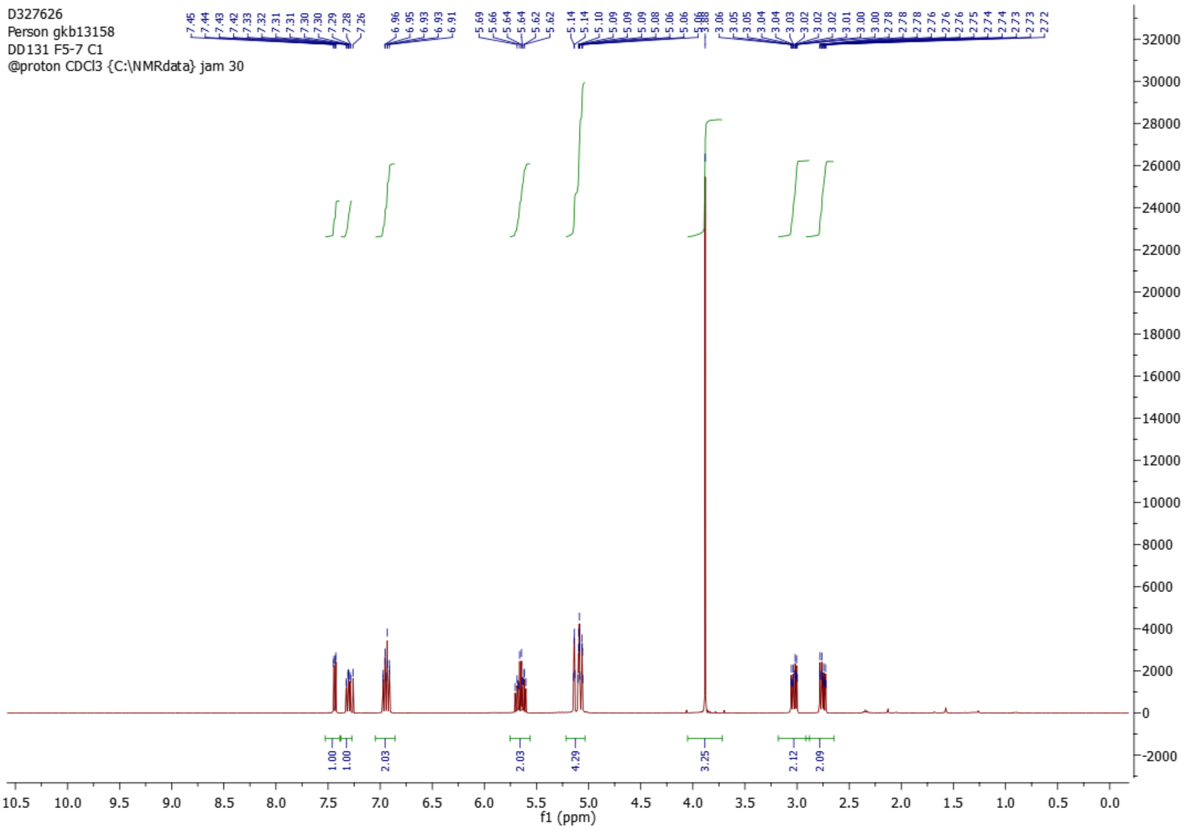
D327626
Person gkb13158
DD131 F5-7 C1
@proton CDCl3 {C:\NMRdata} jam 30
1.00
1.00
2.03
2.03
4.29
3.25
2.12
2.09
f1 (ppm)

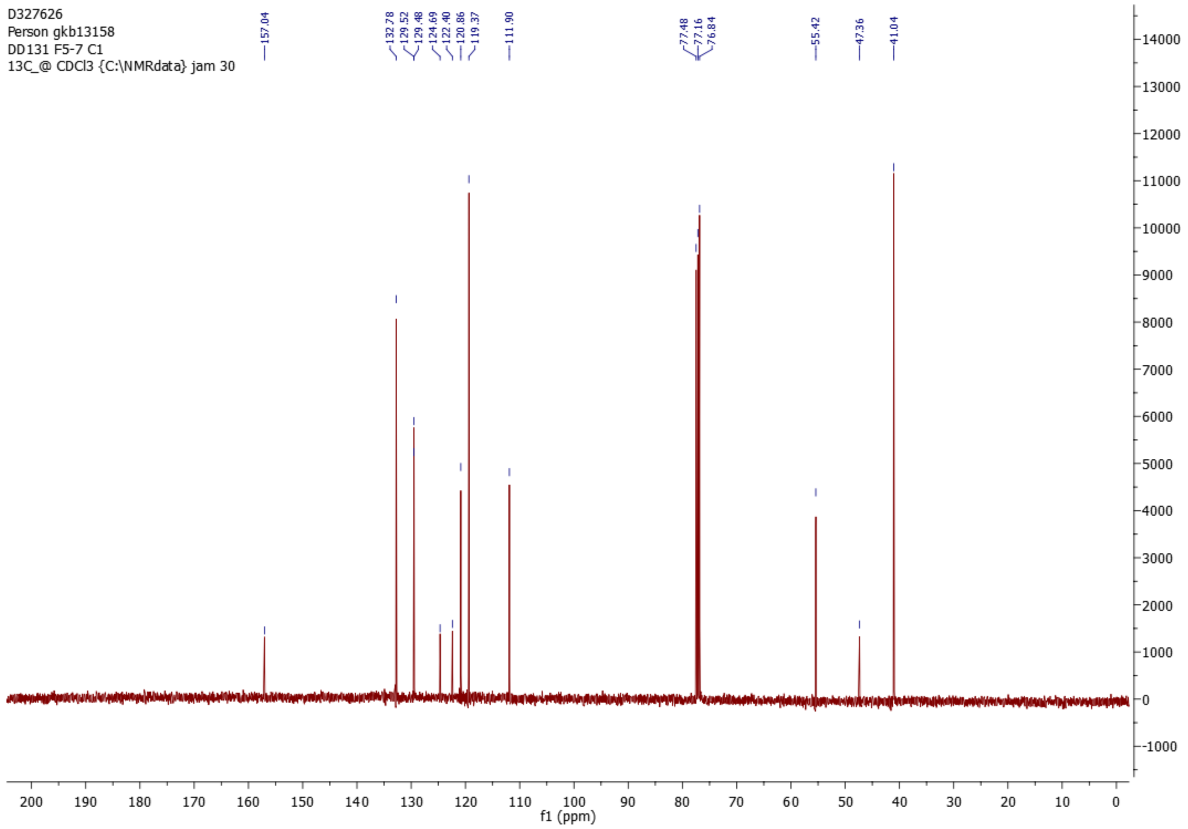
D327626
Person gkb13158
DD131 F5-7 C1
13C_@ CDCl3 {C:\NMRdata} jam 30
157.04
132.78
129.52
129.48
124.69
122.40
120.86
119.37
111.90
77.48
77.16
76.84
55.42
47.36
41.04
f1 (ppm)

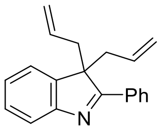
N
Ph

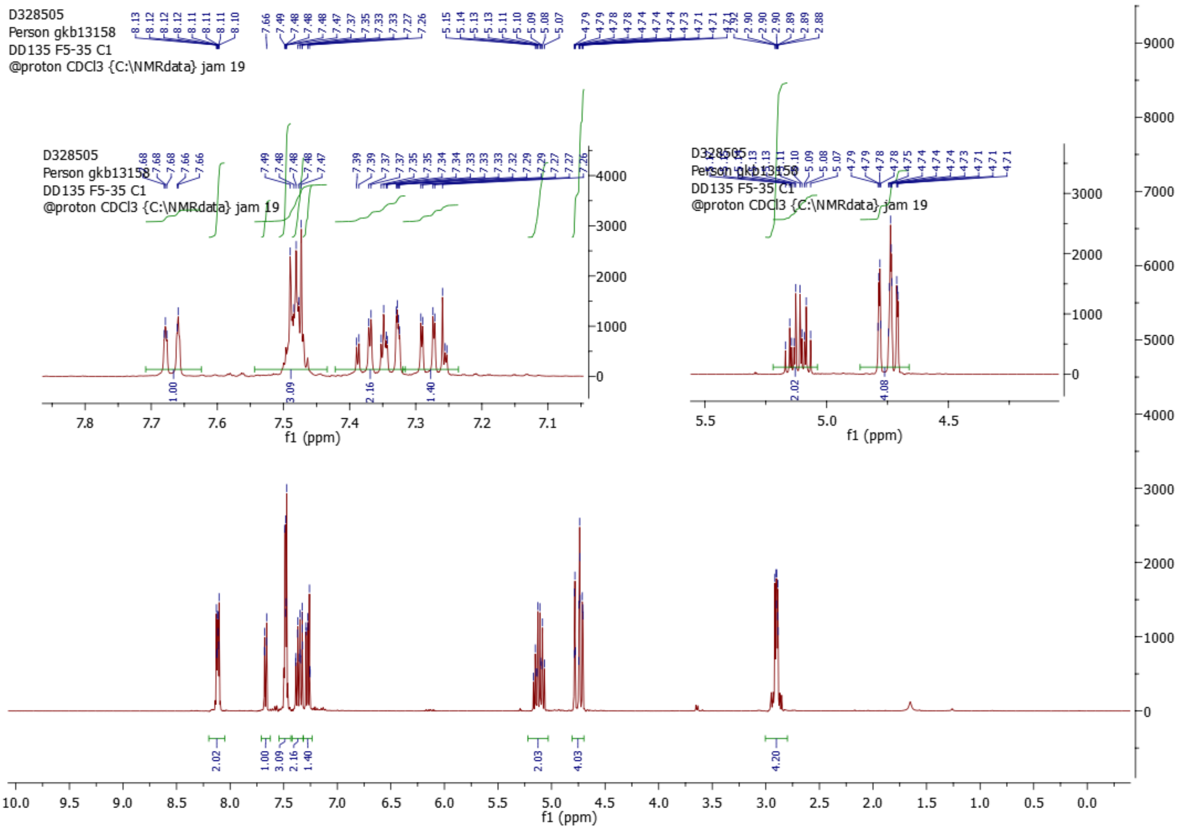
D328505
Person gkb13158
DD135 F5-35 C1
@proton CDCl3 {C:\NMRdata} jam 19
f1 (ppm)

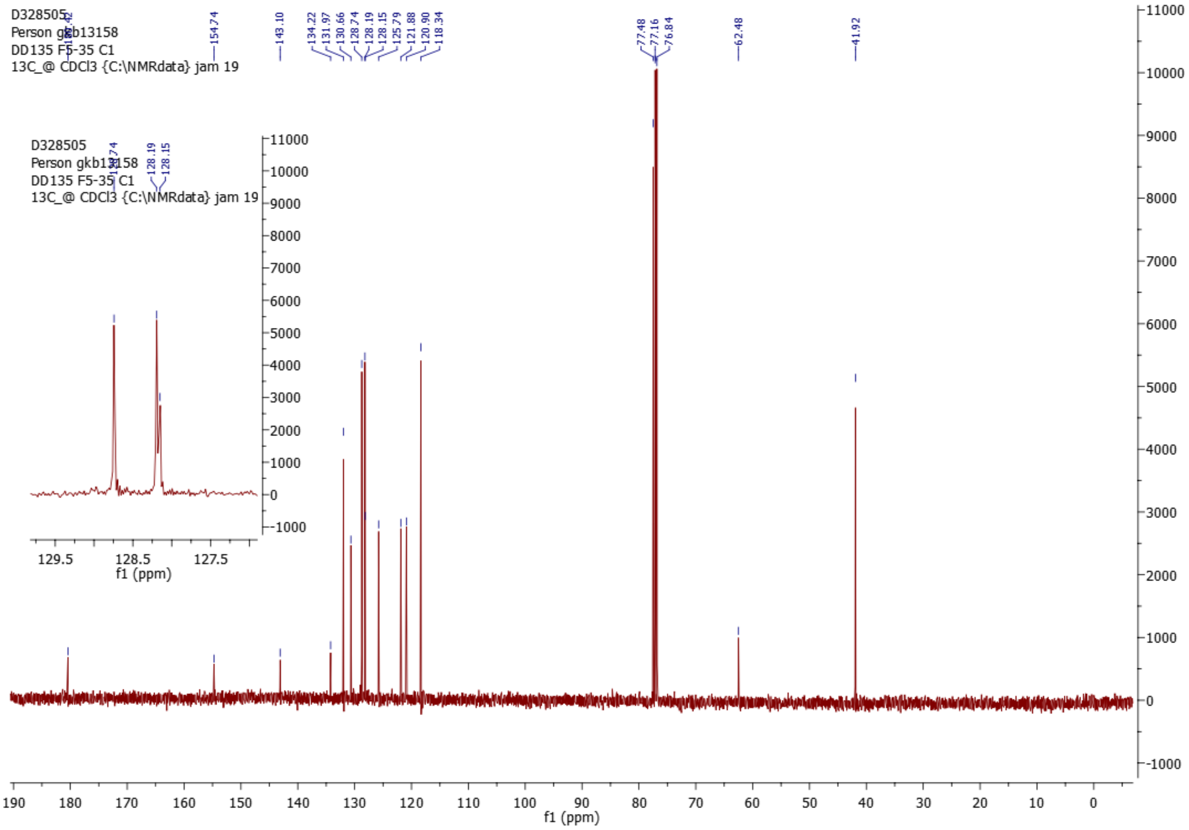
D328505
Person gkb13158
DD135 F5-35 C1
13C_@ CDCl3 {C:\NMRdata} jam 19
154.74
143.10
134.22
131.97
130.66
128.74
128.19
128.15
125.79
121.88
120.90
118.34
77.48
77.16
76.84
62.48
41.92
f1 (ppm)

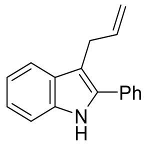
Ph
N
H

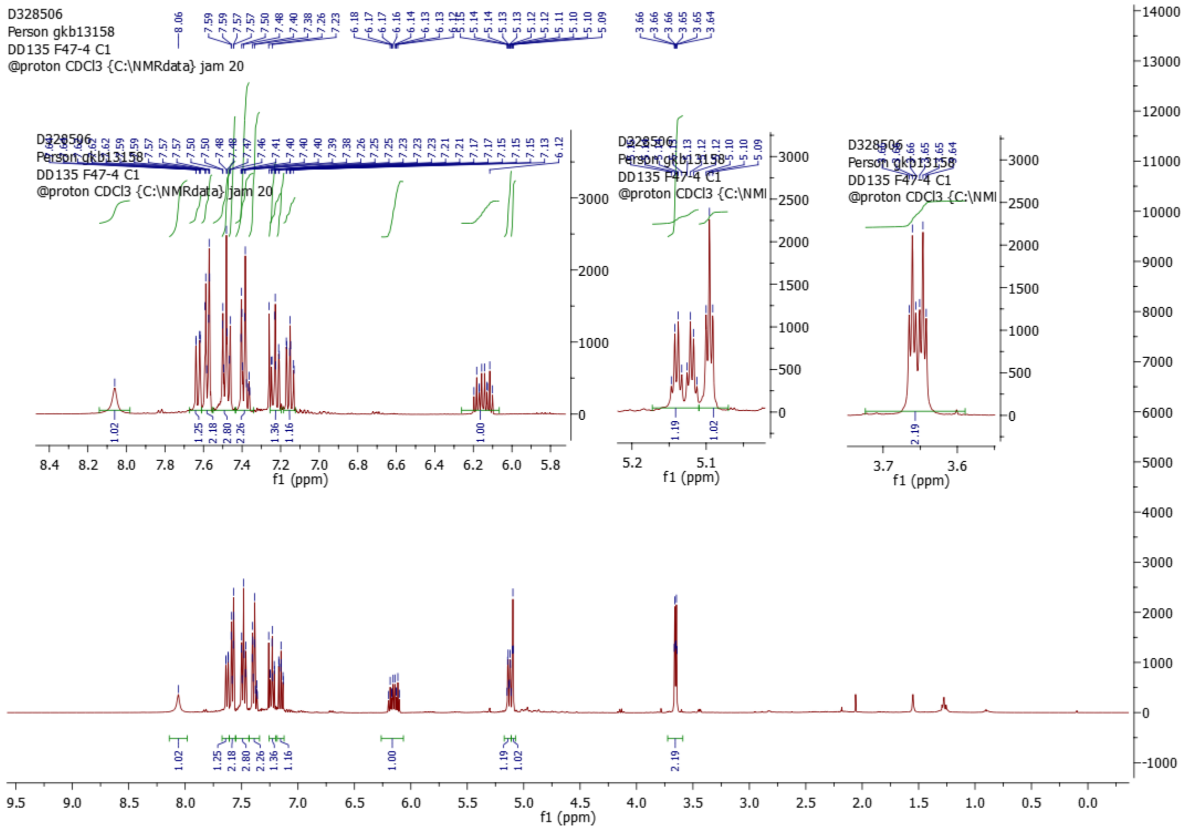
D328506
Person gkb13158
DD135 F47-4 C1
@proton CDCl3 {C:\NMRdata} jam 20
f1 (ppm)

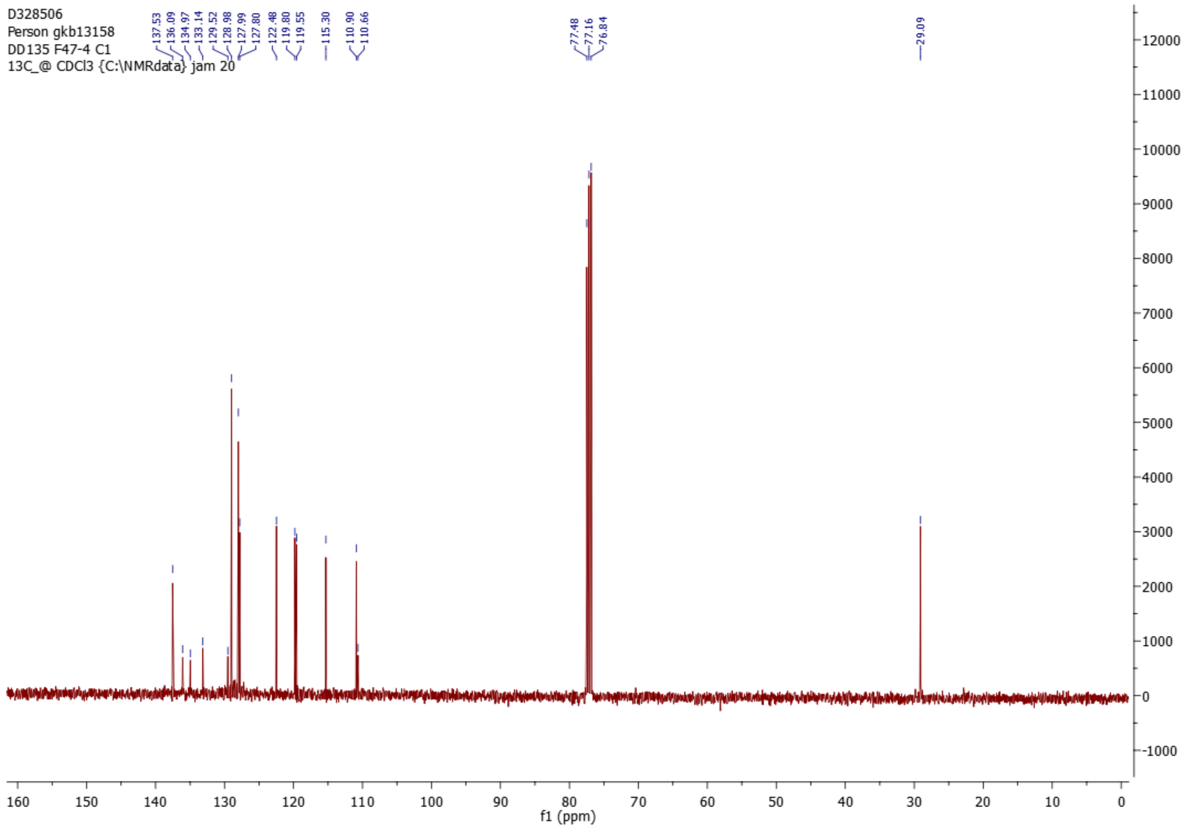
D328506
Person gkb13158
DD135 F47-4 C1
13C_@ CDCl3 {C:\NMRdata} jam 20
137.53
136.09
134.97
133.14
129.52
128.98
127.99
127.80
122.48
119.80
119.55
115.30
110.90
110.66
77.48
77.16
76.84
29.09
f1 (ppm)

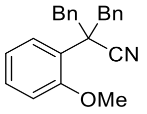
Bn
Bn
CN
OMe

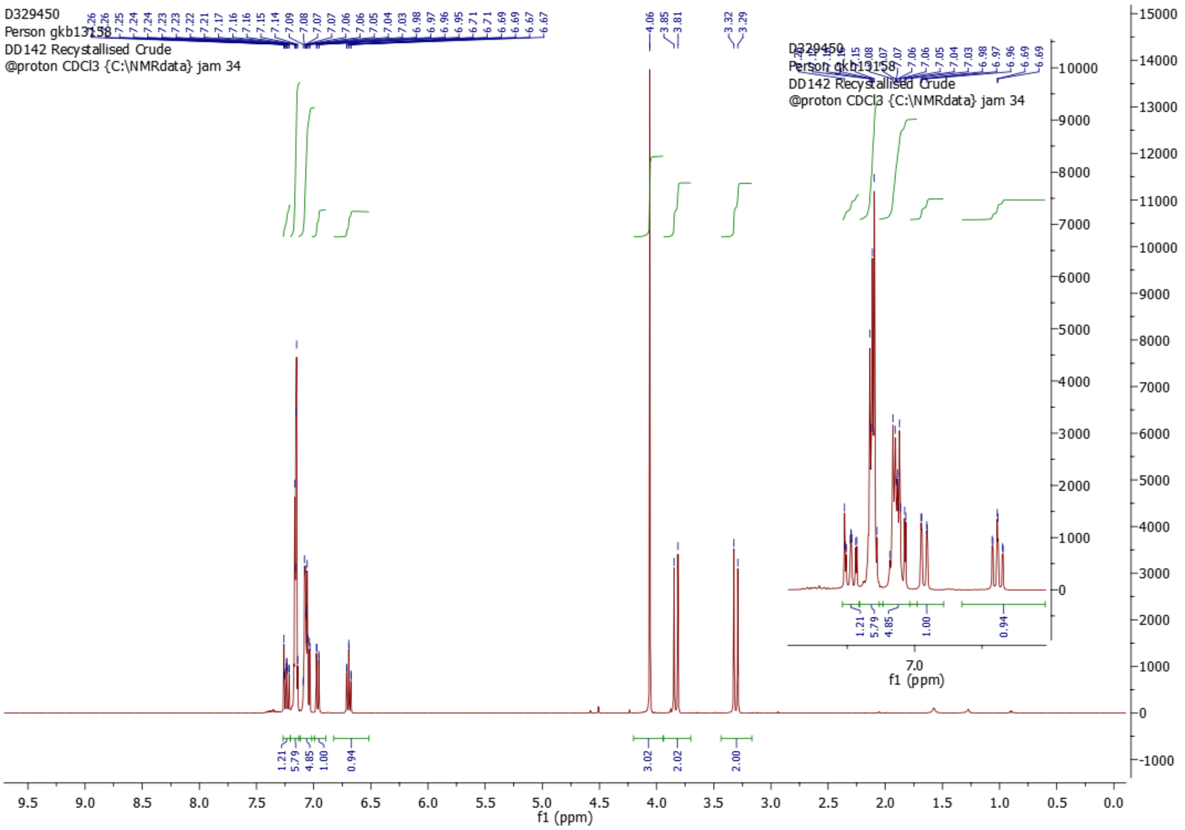
D329450
Person gkb13158
DD142 Recystallised Crude
@proton CDCl3 {C:\NMRdata} jam 34
4.06
3.85
3.81
3.32
3.29
1.21
5.79
4.85
1.00
0.94
3.02
2.02
2.00
f1 (ppm)

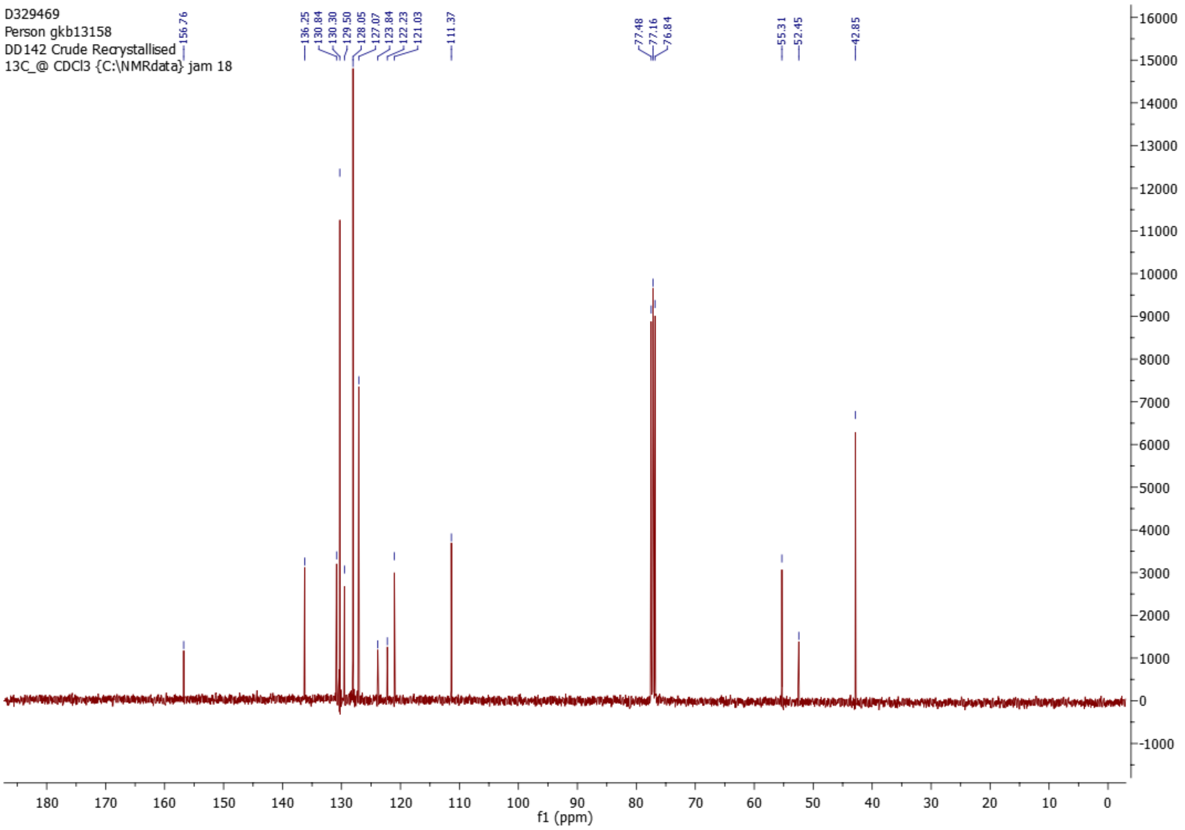
D329469
Person gkb13158
DD142 Crude Recrystallised
13C_@ CDCl3 {C:\NMRdata} jam 18
156.76
136.25
130.84
130.30
129.50
128.05
127.07
123.84
122.23
121.03
111.37
77.48
77.16
76.84
55.31
52.45
42.85
f1 (ppm)

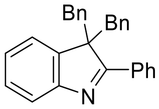
Bn
Bn
Ph
N

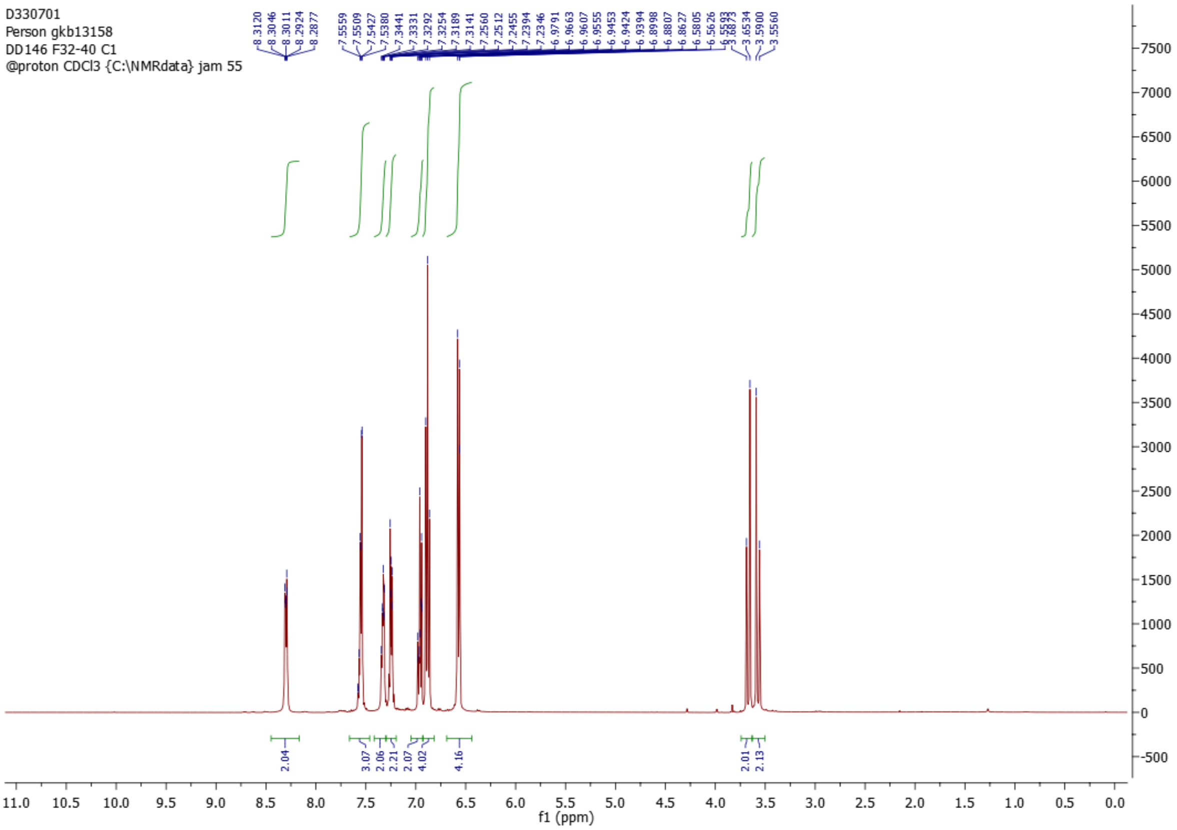
D330701
Person gkb13158
DD146 F32-40 C1
@proton CDCl3 {C:\NMRdata} jam 55
f1 (ppm)

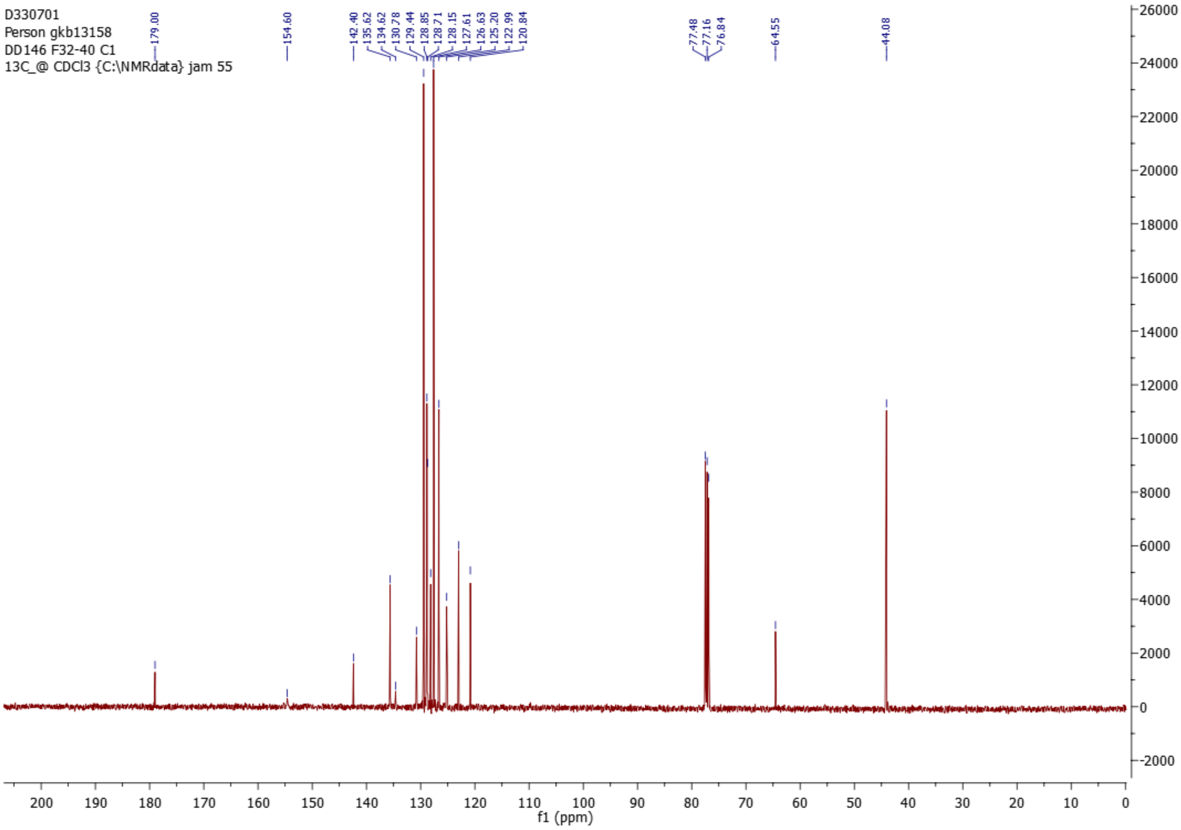
D330701
Person gkb13158
DD146 F32-40 C1
13C_@ CDCl3 {C:\NMRdata} jam 55
179.00
154.60
142.40
135.62
134.62
130.78
129.44
128.85
128.71
128.15
127.61
126.63
125.20
122.99
120.84
77.48
77.16
76.84
64.55
44.08
f1 (ppm)

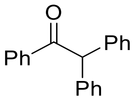
O
Ph
Ph
Ph

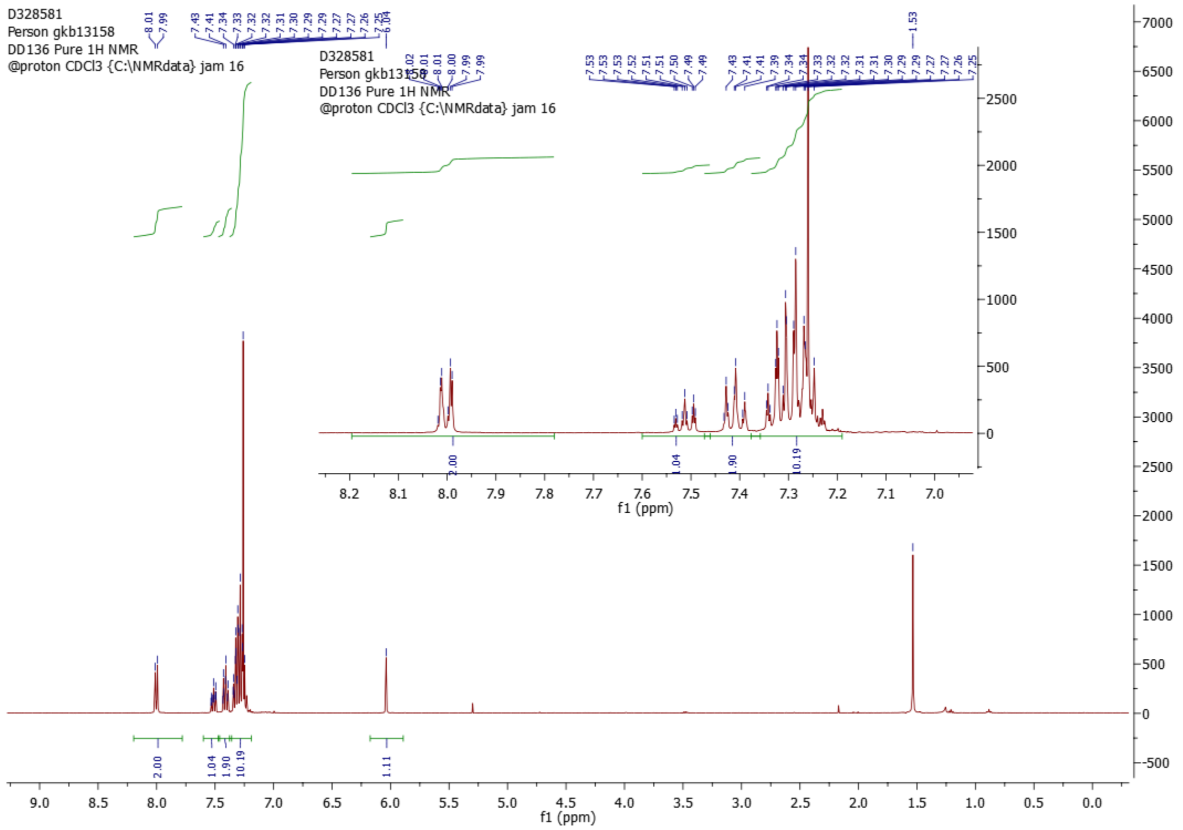
D328581
Person gkb13158
DD136 Pure 1H NMR
@proton CDCl3 {C:\NMRdata} jam 16
f1 (ppm)

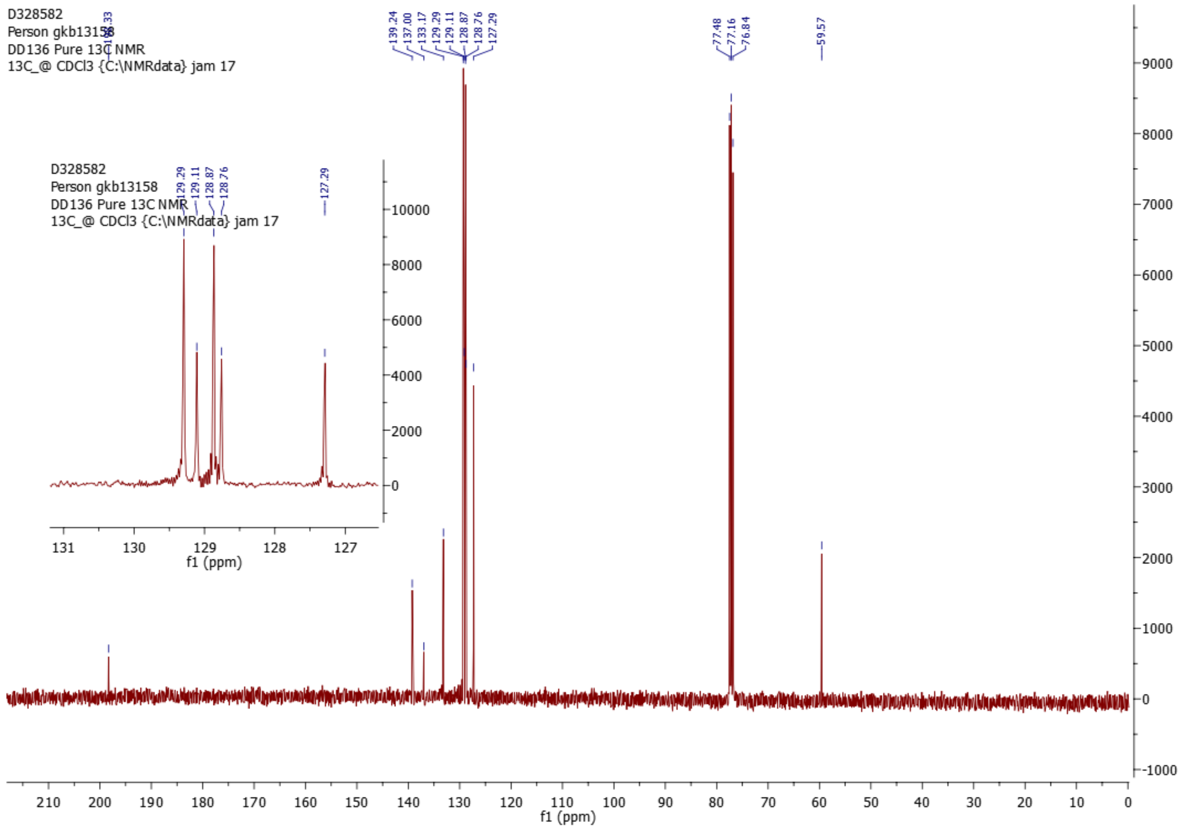
D328582
Person gkb13158
DD136 Pure 13C NMR
13C_@ CDCl3 {C:\NMRdata} jam 17
f1 (ppm)

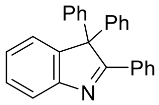
Ph
Ph
Ph
N

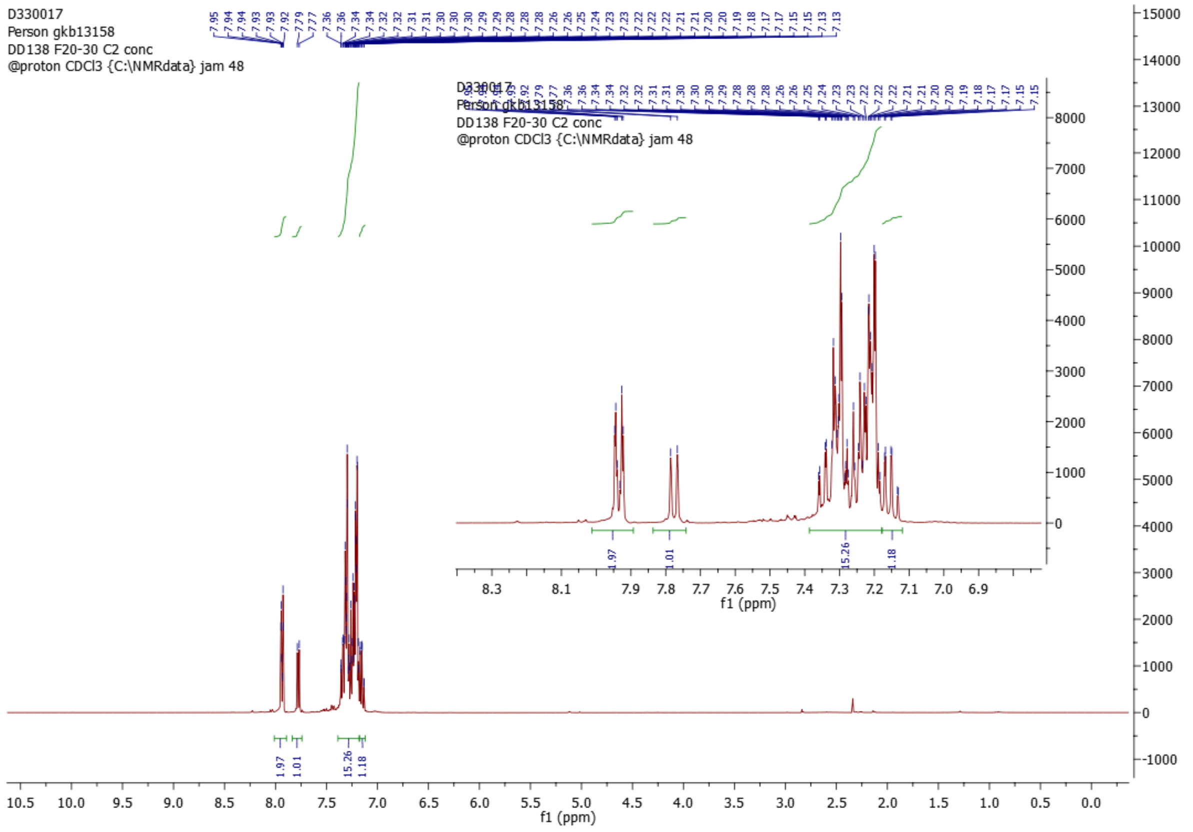
D330017
Person gkb13158
DD138 F20-30 C2 conc
@proton CDCl3 {C:\NMRdata} jam 48
f1 (ppm)

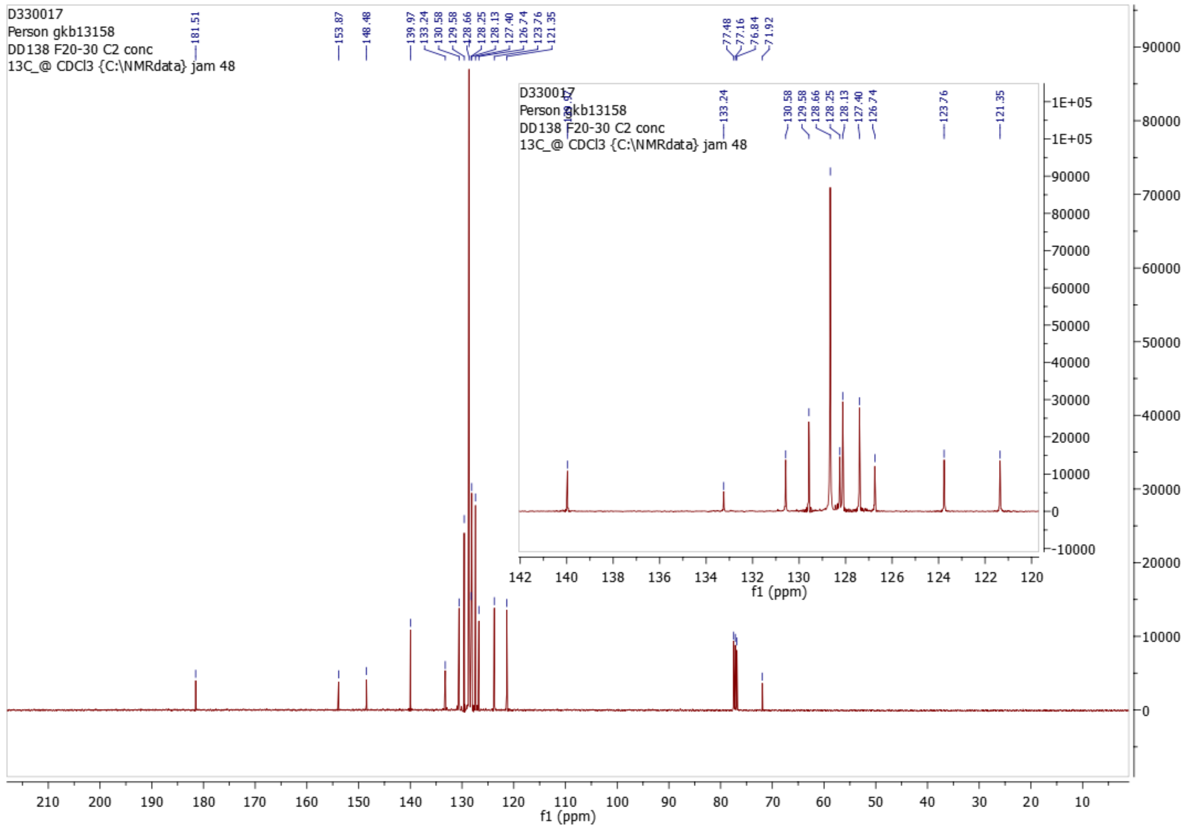
D330017
Person gkb13158
DD138 F20-30 C2 conc
13C_@ CDCl3 {C:\NMRdata} jam 48
181.51
153.87
148.48
139.97
133.24
130.58
129.58
128.66
128.25
128.13
127.40
126.74
123.76
121.35
77.48
77.16
76.84
71.92
f1 (ppm)

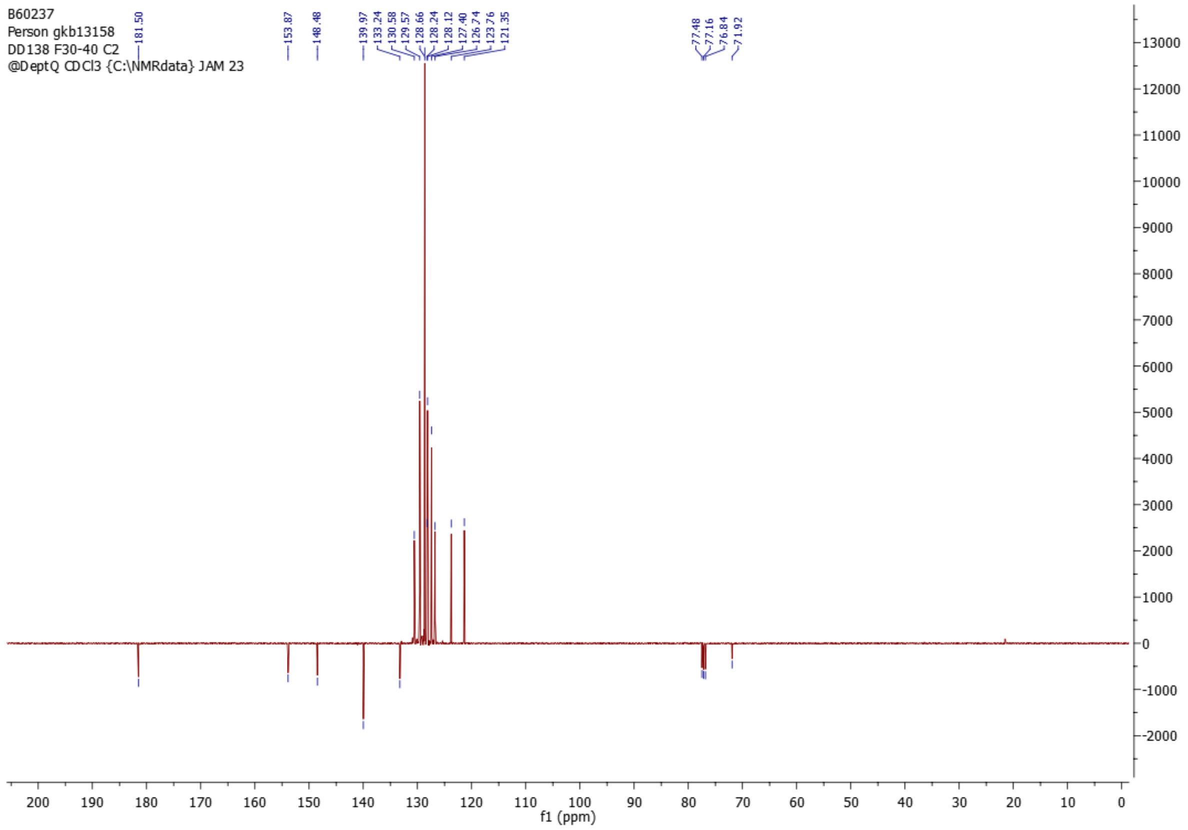

B60237
Person gkb13158
DD138 F30-40 C2
@DeptQ CDCl3 {C:\NMRdata} JAM 23
181.50
153.87
148.48
139.97
133.24
130.58
129.57
128.66
128.24
128.12
127.40
126.74
123.76
121.35
77.48
77.16
76.84
71.92
f1 (ppm)

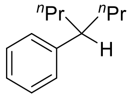

ⁿPr
ⁿPr
H

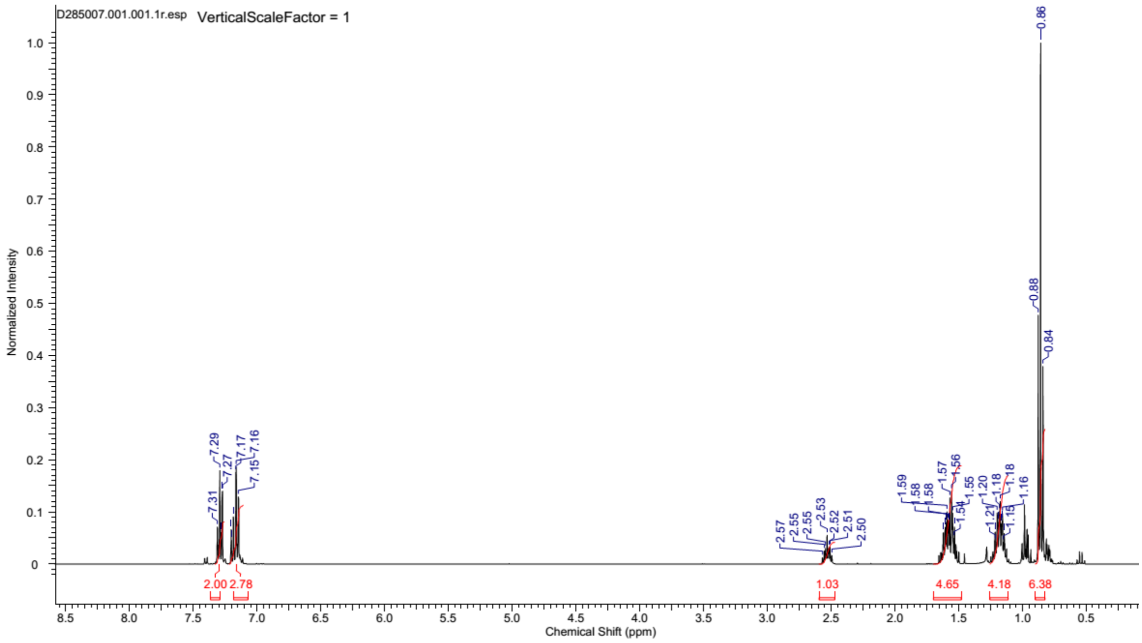

D285007.001.001.1r.esp
VerticalScaleFactor = 1
Normalized Intensity
Chemical Shift (ppm)
7.31
7.29
7.27
7.17
7.16
7.15
2.57
2.55
2.55
2.53
2.52
2.51
2.50
1.59
1.58
1.58
1.57
1.56
1.55
1.54
1.21
1.20
1.18
1.18
1.16
1.15
0.88
0.86
0.84
2.00
2.78
1.03
4.65
4.18
6.38

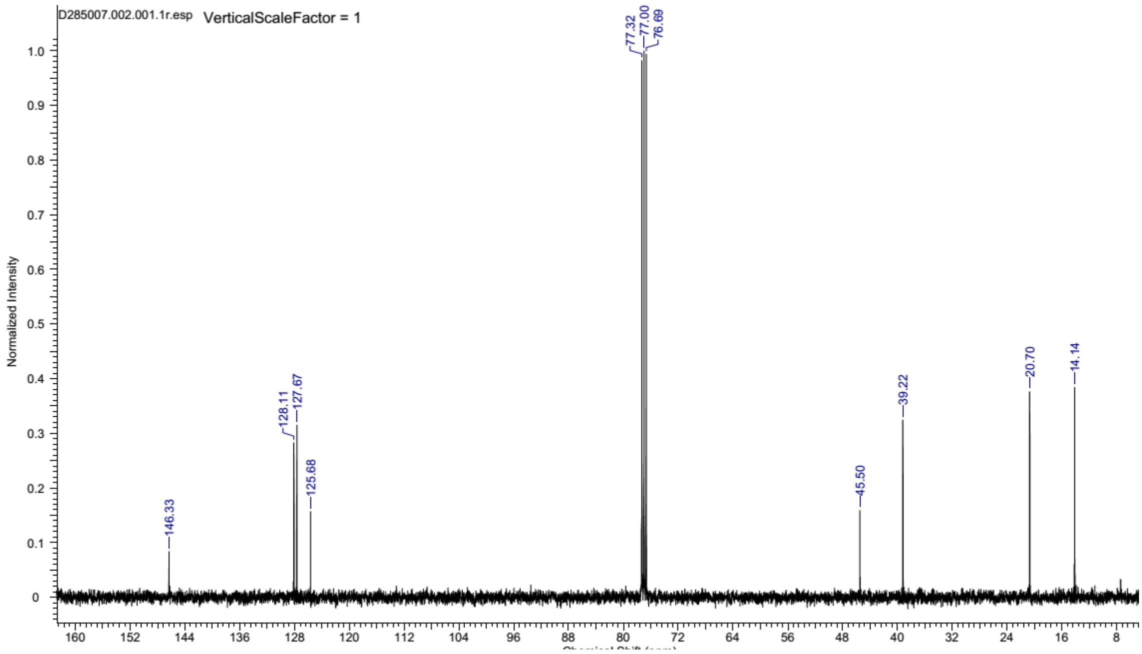

D285007.002.001.1r.esp
VerticalScaleFactor = 1
Normalized Intensity
146.33
128.11
127.67
125.68
77.32
77.00
76.69
45.50
39.22
20.70
14.14

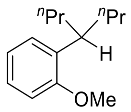
ⁿPr
ⁿPr
H
OMe

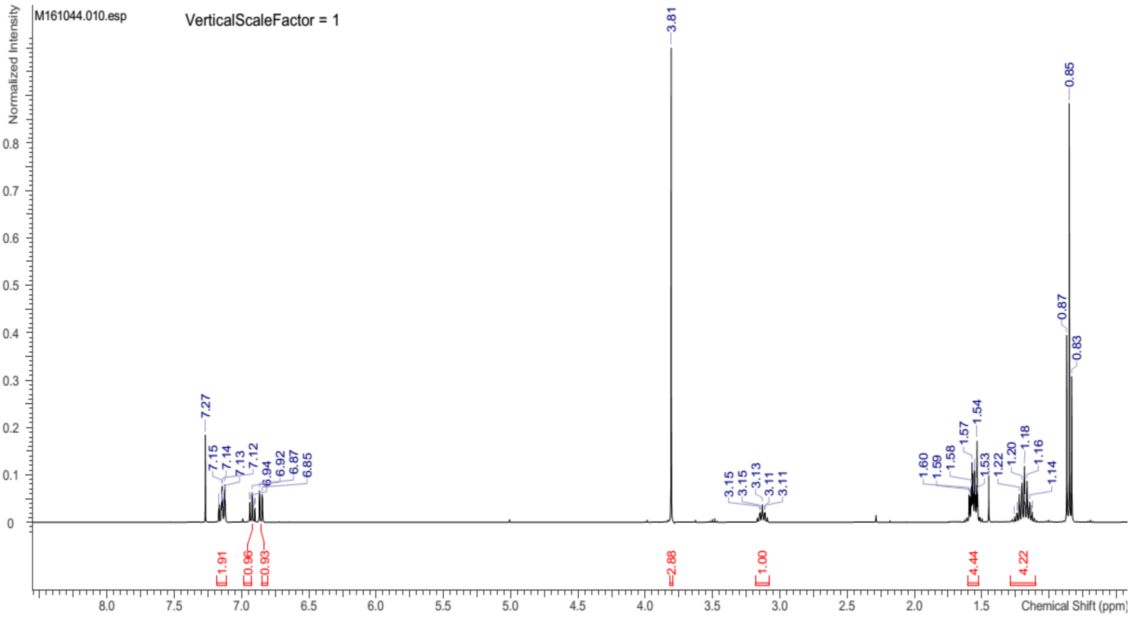
M161044.010.esp
VerticalScaleFactor = 1
Normalized Intensity
3.81
0.85
0.87
0.83
7.27
7.15
7.14
7.13
7.12
6.94
6.92
6.87
6.85
3.15
3.15
3.13
3.11
3.11
1.60
1.59
1.58
1.57
1.54
1.53
1.22
1.20
1.18
1.16
1.14
1.91
0.96
0.93
2.88
1.00
4.44
4.22
Chemical Shift (ppm)

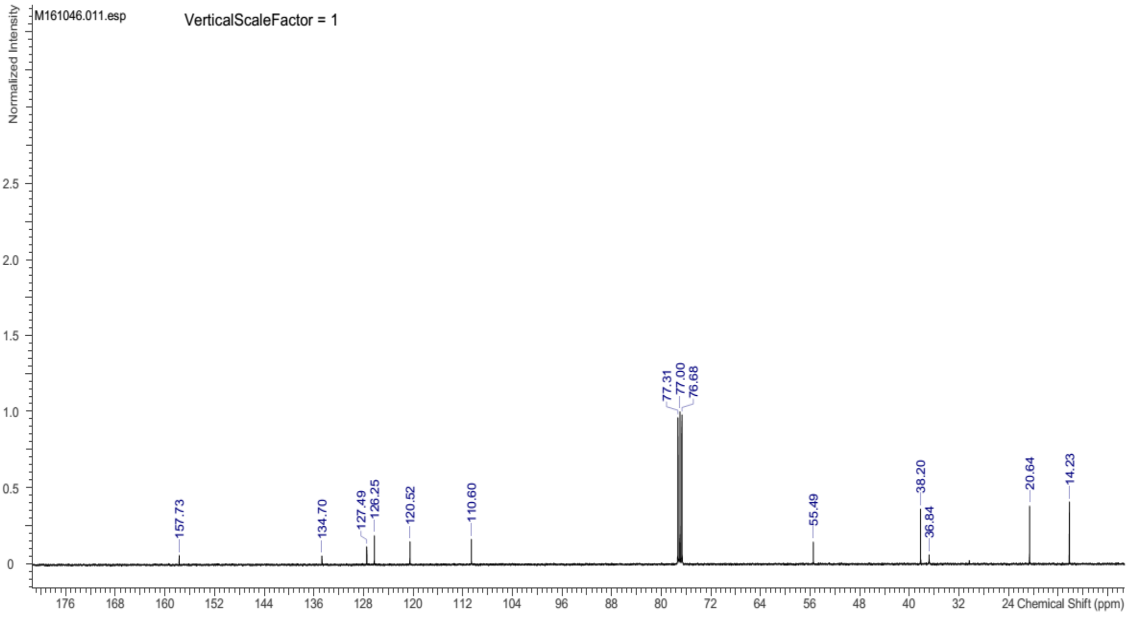
M161046.011.esp
VerticalScaleFactor = 1
Normalized Intensity
157.73
134.70
127.49
126.25
120.52
110.60
77.31
77.00
76.68
55.49
38.20
36.84
20.64
14.23
Chemical Shift (ppm)

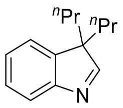

nPr nPr
N

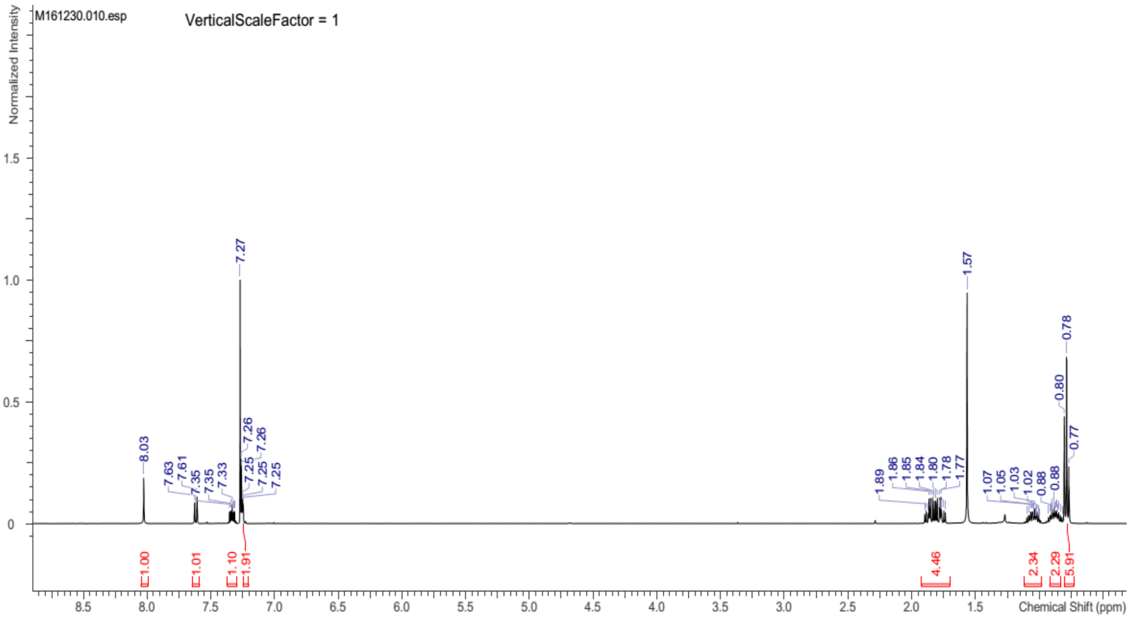

M161230.010.esp
VerticalScaleFactor = 1
Normalized Intensity
8.03
7.63 7.61 7.35 7.35 7.33
7.27 7.26 7.26 7.25 7.25 7.25
1.89 1.86 1.85 1.84 1.80 1.78 1.77
1.57
1.07 1.05 1.03 1.02 0.88 0.88
0.80 0.78 0.77
1.00 1.01 1.10 1.91
4.46 2.34 2.29 5.91
Chemical Shift (ppm)

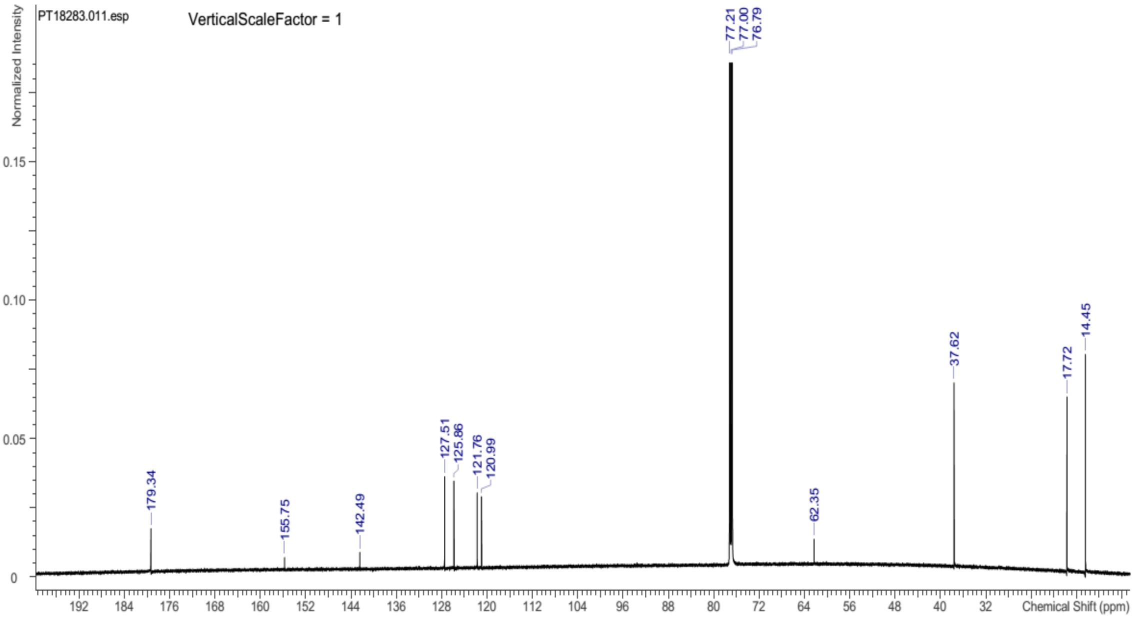

PT18283.011.esp
VerticalScaleFactor = 1
Normalized Intensity
179.34
155.75
142.49
127.51 125.86 121.76 120.99
77.21 77.00 76.79
62.35
37.62
17.72
14.45
Chemical Shift (ppm)

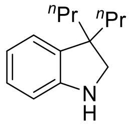

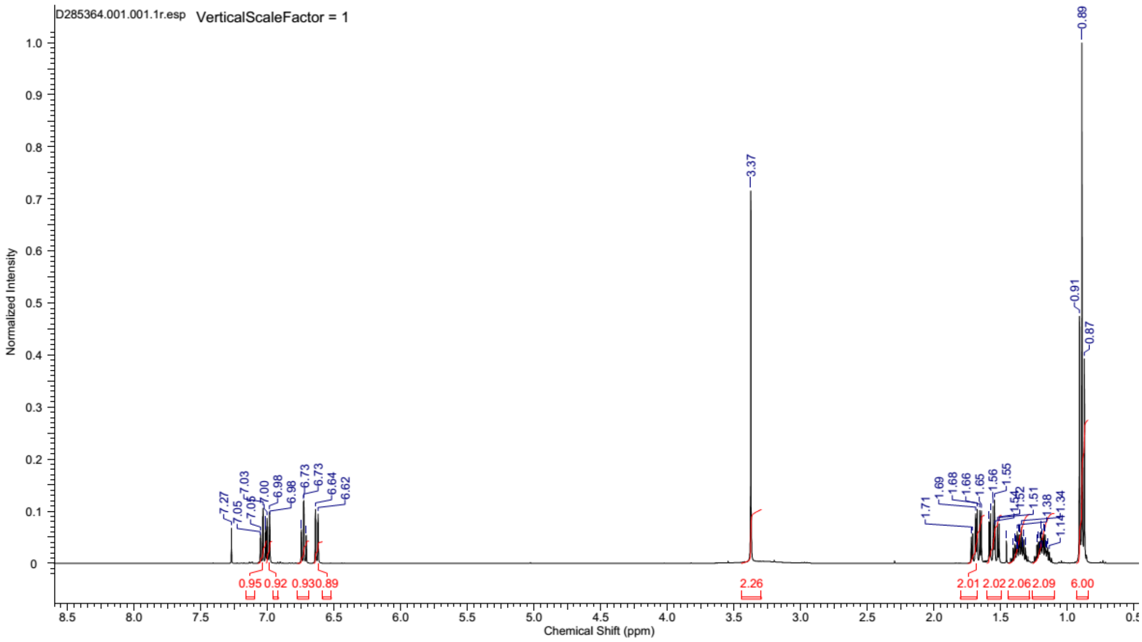
D285364.001.001.1r.esp VerticalScaleFactor = 1
Normalized Intensity
Chemical Shift (ppm)
0.89
3.37
0.91
0.87
0.95 0.92 0.93 0.89
2.26
2.01 2.02 2.06 2.09 6.00

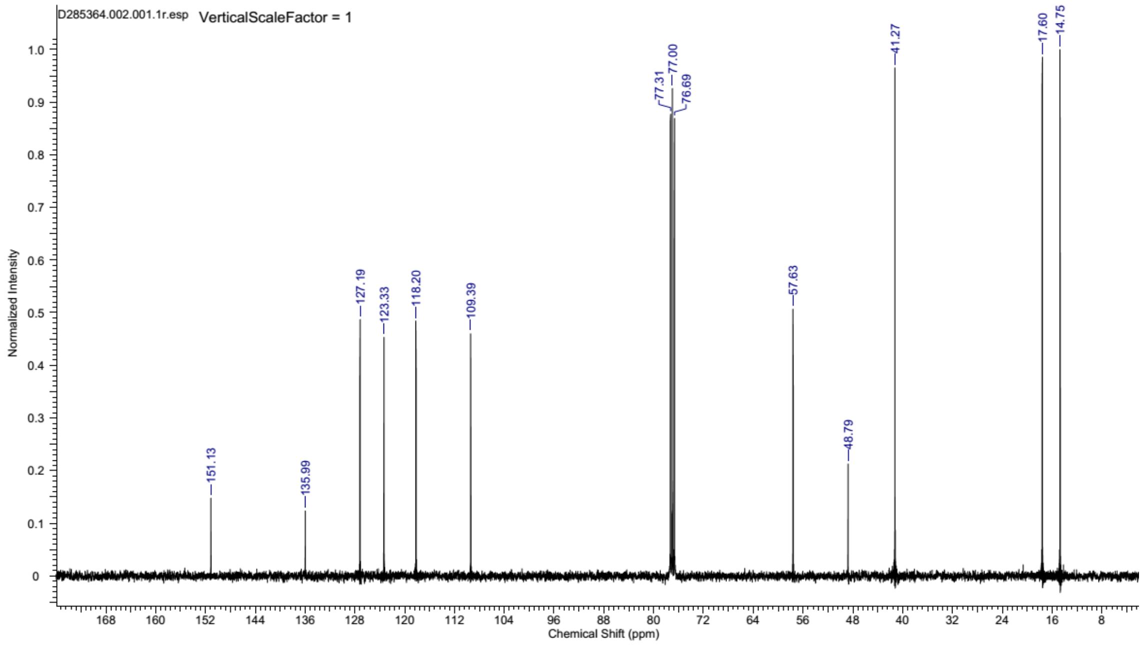
D285364.002.001.1r.esp VerticalScaleFactor = 1
Normalized Intensity
Chemical Shift (ppm)
151.13
135.99
127.19
123.33
118.20
109.39
77.31
77.00
76.69
57.63
48.79
41.27
17.60
14.75

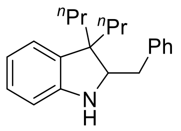
nPr
nPr
Ph
N
H

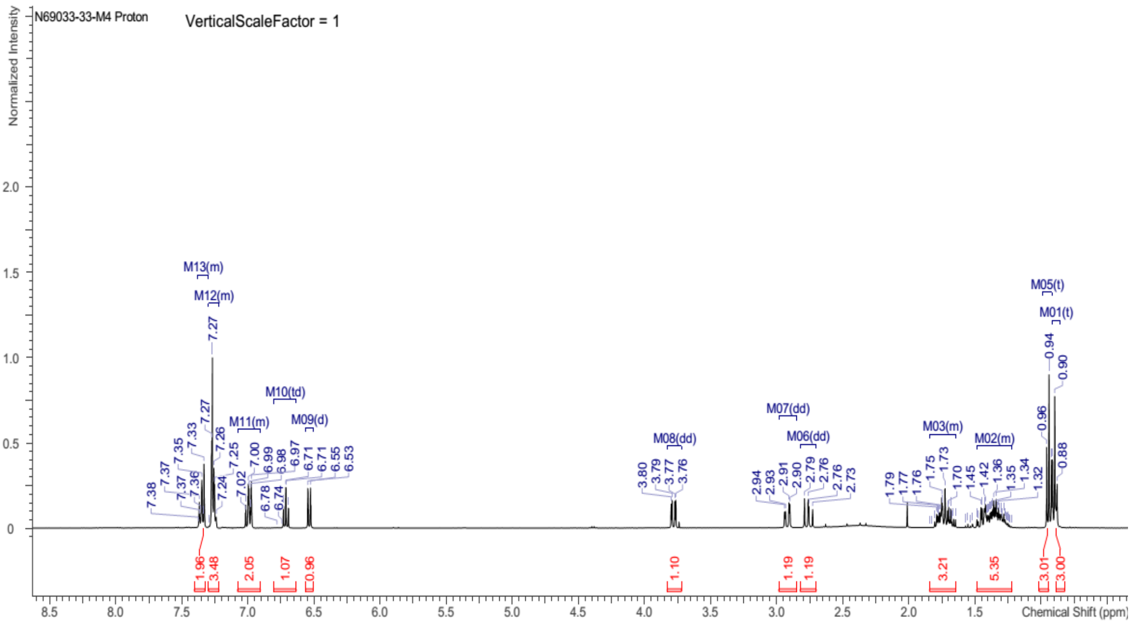
N69033-33-M4 Proton
VerticalScaleFactor = 1
Normalized Intensity
Chemical Shift (ppm)
M13(m)
M12(m)
M11(m)
M10(td)
M09(d)
M08(dd)
M07(dd)
M06(dd)
M03(m)
M02(m)
M05(t)
M01(t)
7.38 7.37 7.37 7.36 7.35 7.33 7.27 7.27 7.26 7.25 7.24 7.02 7.00 6.99 6.98 6.97 6.78 6.74 6.71 6.71 6.55 6.53
3.80 3.79 3.77 3.76
2.94 2.93 2.91 2.90 2.79 2.76 2.76 2.73
1.79 1.77 1.76 1.75 1.73 1.70 1.45 1.42 1.36 1.35 1.34 1.32
0.96 0.94 0.90 0.88
1.96 3.48 2.05 1.07 0.96 1.10 1.19 1.19 3.21 5.35 3.01 3.00

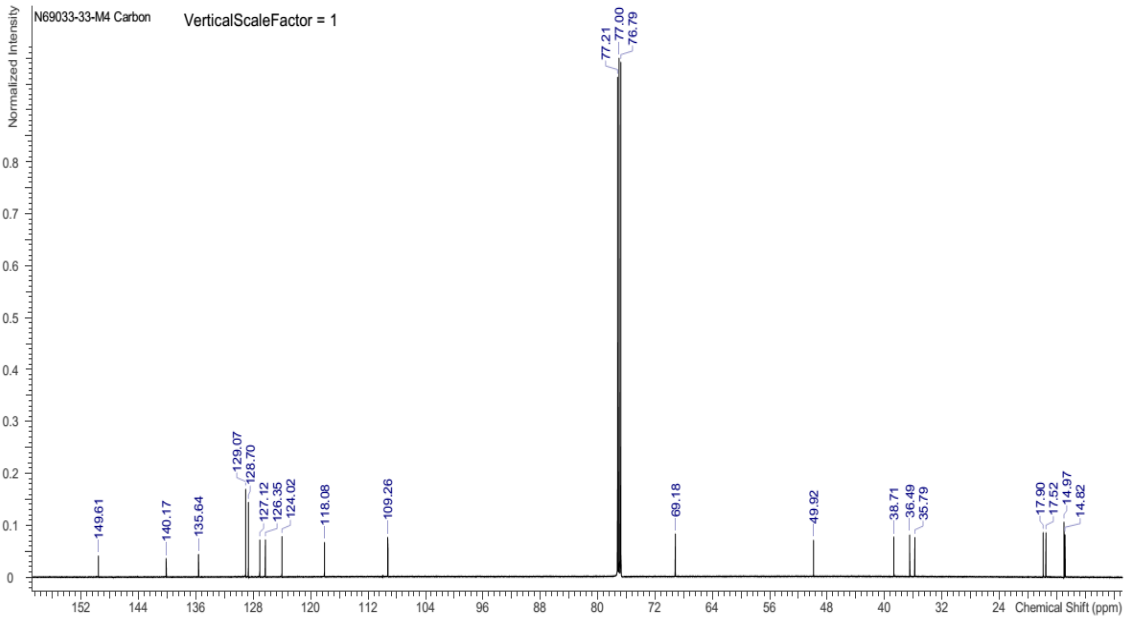
N69033-33-M4 Carbon
VerticalScaleFactor = 1
Normalized Intensity
Chemical Shift (ppm)
149.61
140.17
135.64
129.07
128.70
127.12
126.35
124.02
118.08
109.26
77.21
77.00
76.79
69.18
49.92
38.71
36.49
35.79
17.90
17.52
14.97
14.82

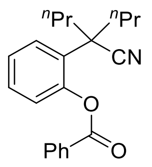

nPr
nPr
CN
O
Ph
O

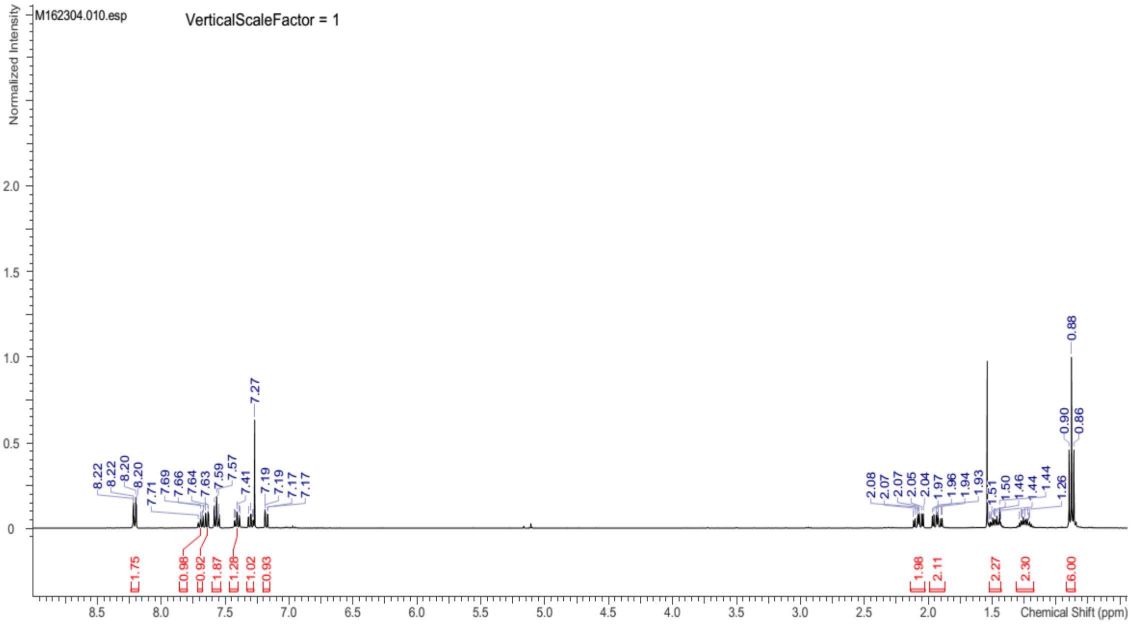

M162304.010.esp
VerticalScaleFactor = 1
Normalized Intensity
Chemical Shift (ppm)

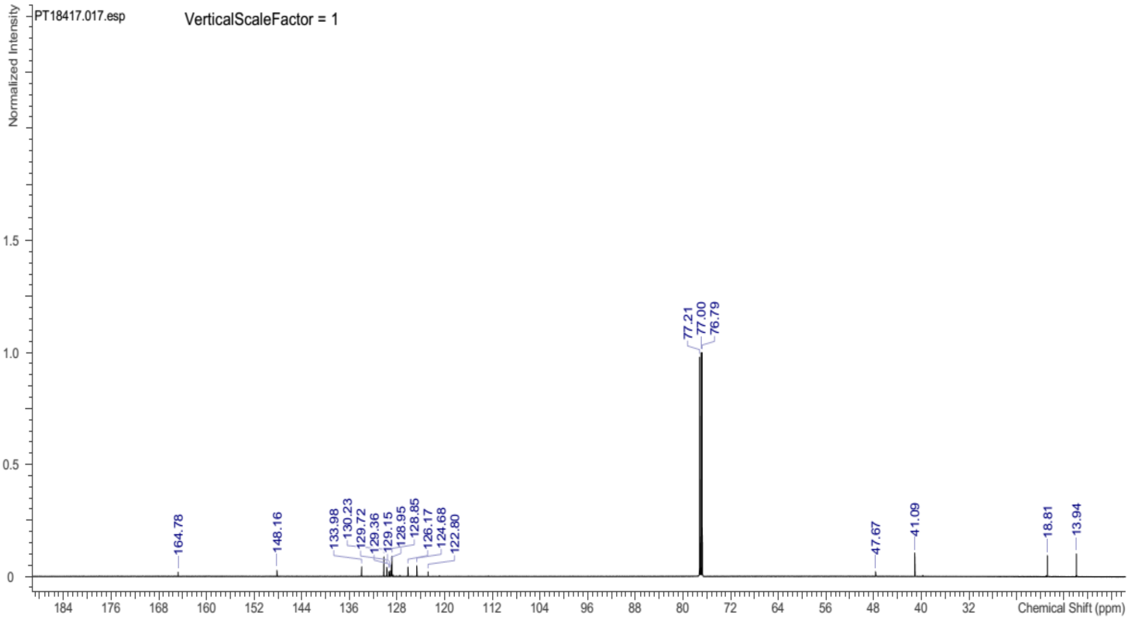

PT18417.017.esp
VerticalScaleFactor = 1
Normalized Intensity
Chemical Shift (ppm)

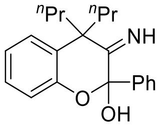

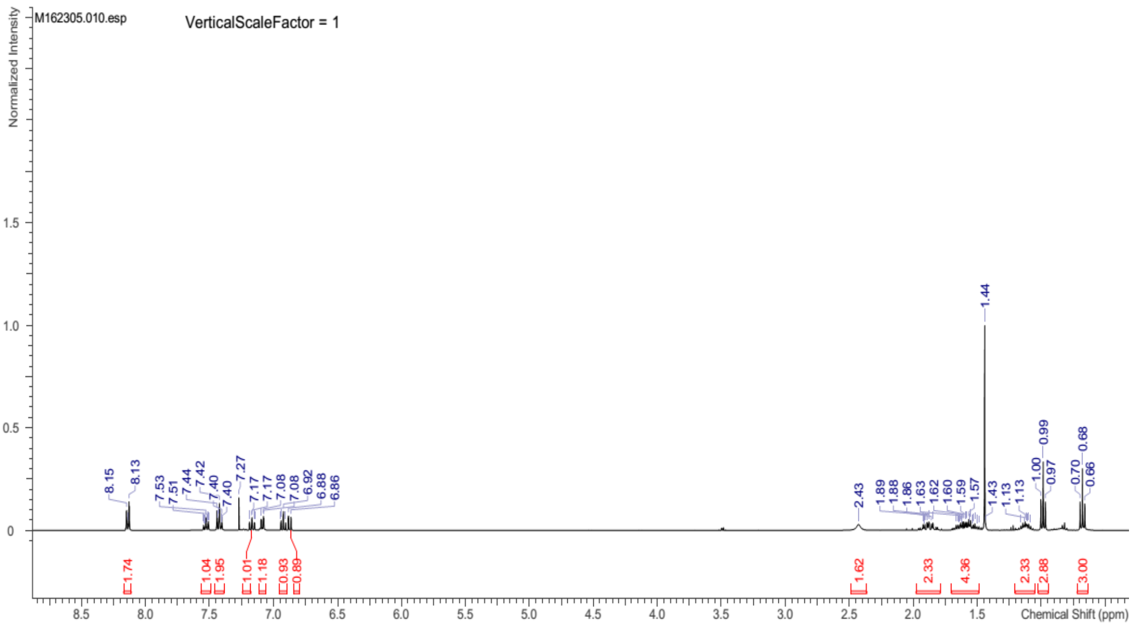
M162305.010.esp
VerticalScaleFactor = 1
Normalized Intensity
Chemical Shift (ppm)

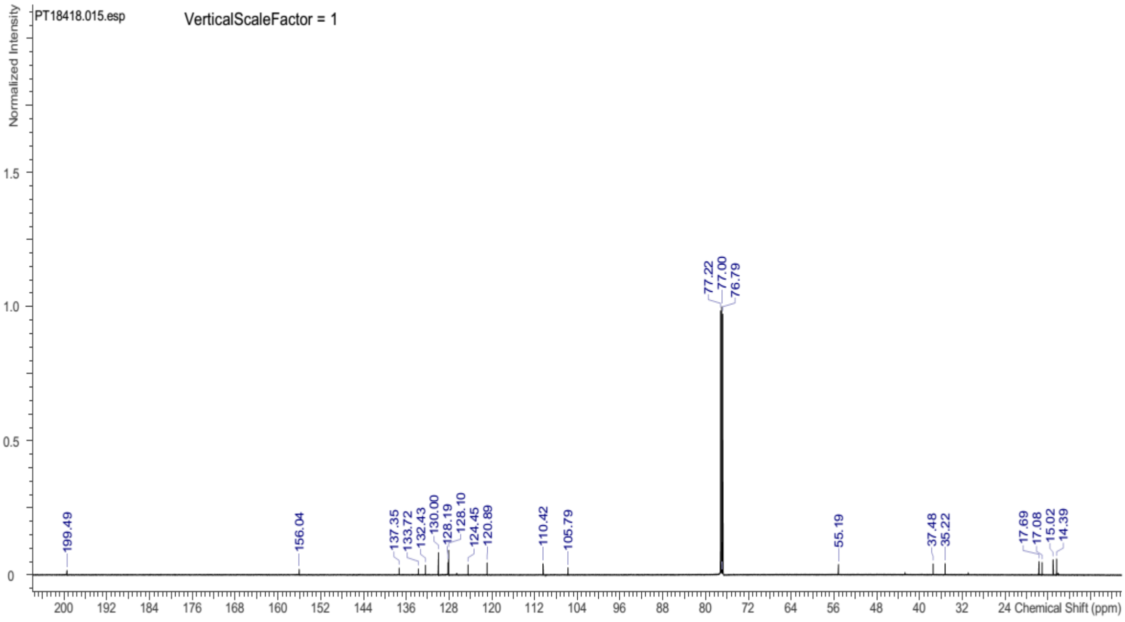
PT18418.015.esp
VerticalScaleFactor = 1
Normalized Intensity
Chemical Shift (ppm)

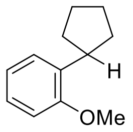

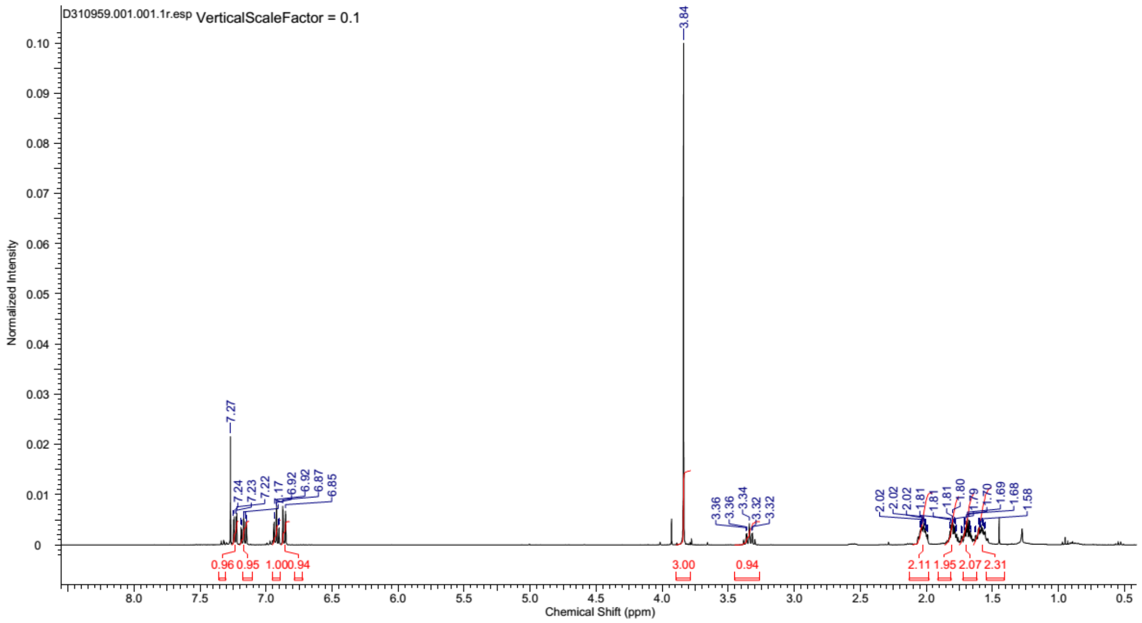

D310959.001.001.1r.esp VerticalScaleFactor = 0.1
Normalized Intensity
Chemical Shift (ppm)

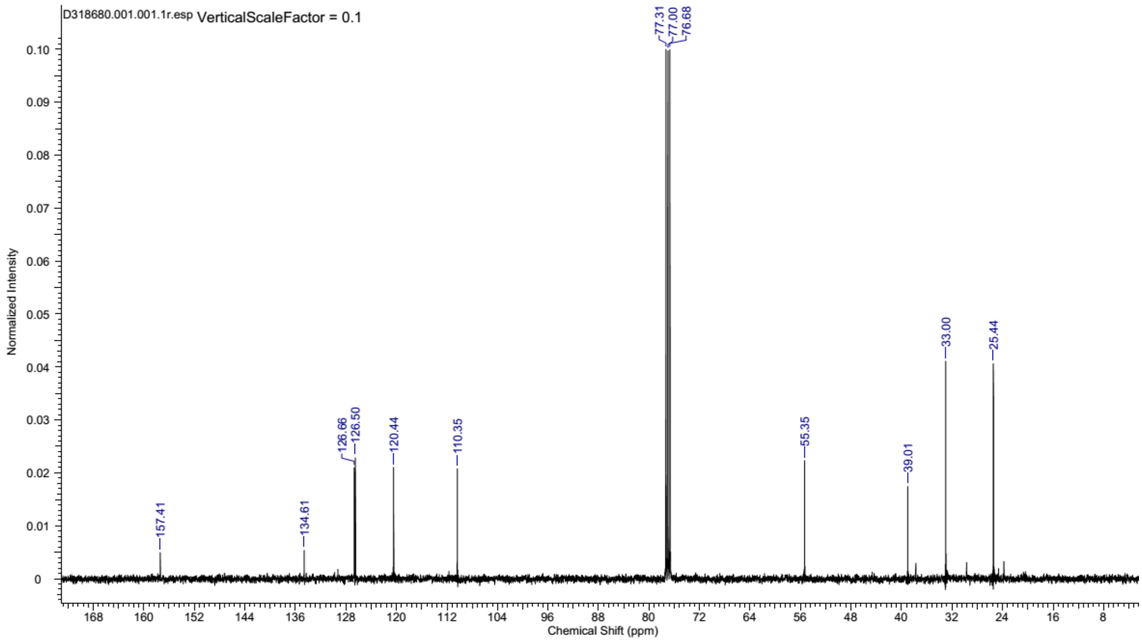

D318680.001.001.1r.esp VerticalScaleFactor = 0.1
Normalized Intensity
Chemical Shift (ppm)

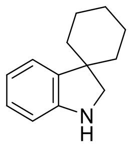

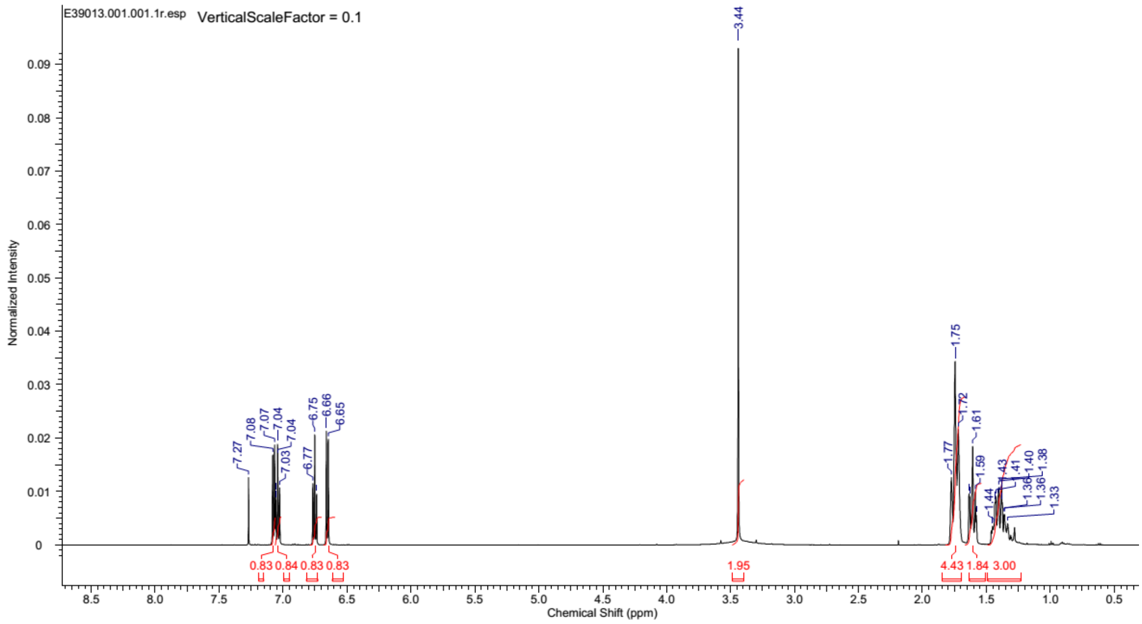

E39013.001.001.1r.esp VerticalScaleFactor = 0.1
Normalized Intensity
Chemical Shift (ppm)
3.44
1.75
7.27
7.08
7.07
7.04
7.04
7.03
6.77
6.75
6.66
6.65
1.77
1.72
1.61
1.59
1.44
1.43
1.41
1.40
1.38
1.36
1.36
1.33
0.83
0.84
0.83
0.83
1.95
4.43
1.84
3.00

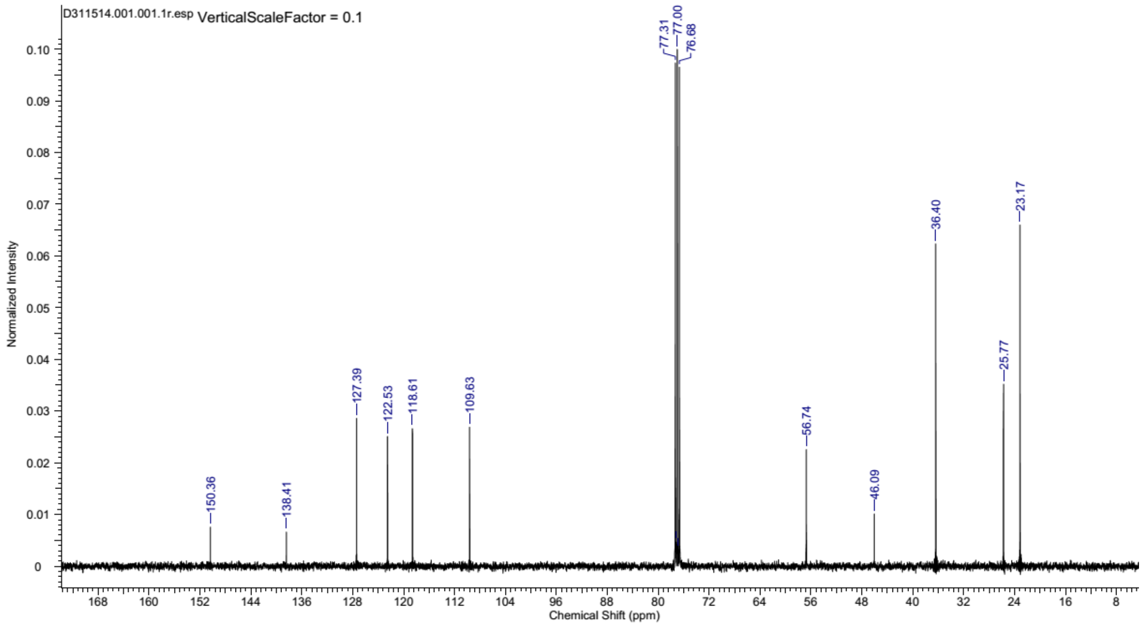

D311514.001.001.1r.esp VerticalScaleFactor = 0.1
Normalized Intensity
Chemical Shift (ppm)
77.31
77.00
76.68
150.36
138.41
127.39
122.53
118.61
109.63
56.74
46.09
36.40
25.77
23.17

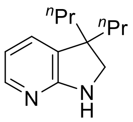

^{n}Pr
^{n}Pr
N
N
H

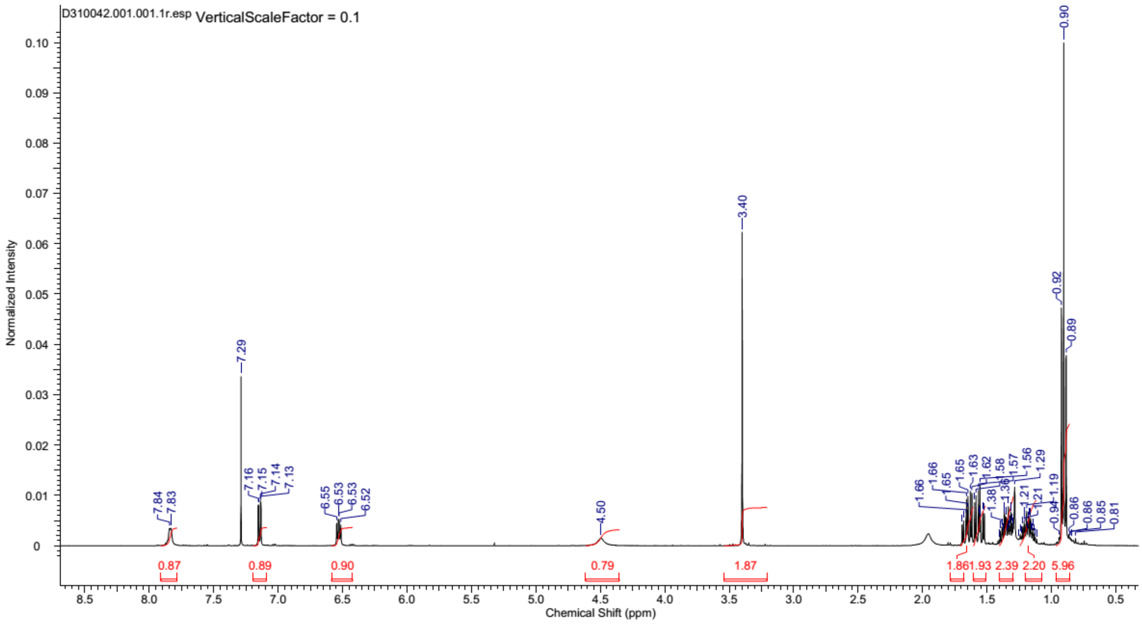

D310042.001.001.1r.esp VerticalScaleFactor = 0.1
Normalized Intensity
Chemical Shift (ppm)
7.84
7.83
7.29
7.16
7.15
7.14
7.13
6.55
6.53
6.53
6.52
4.50
3.40
0.92
0.90
0.89
0.87
0.89
0.90
0.79
1.87
5.96

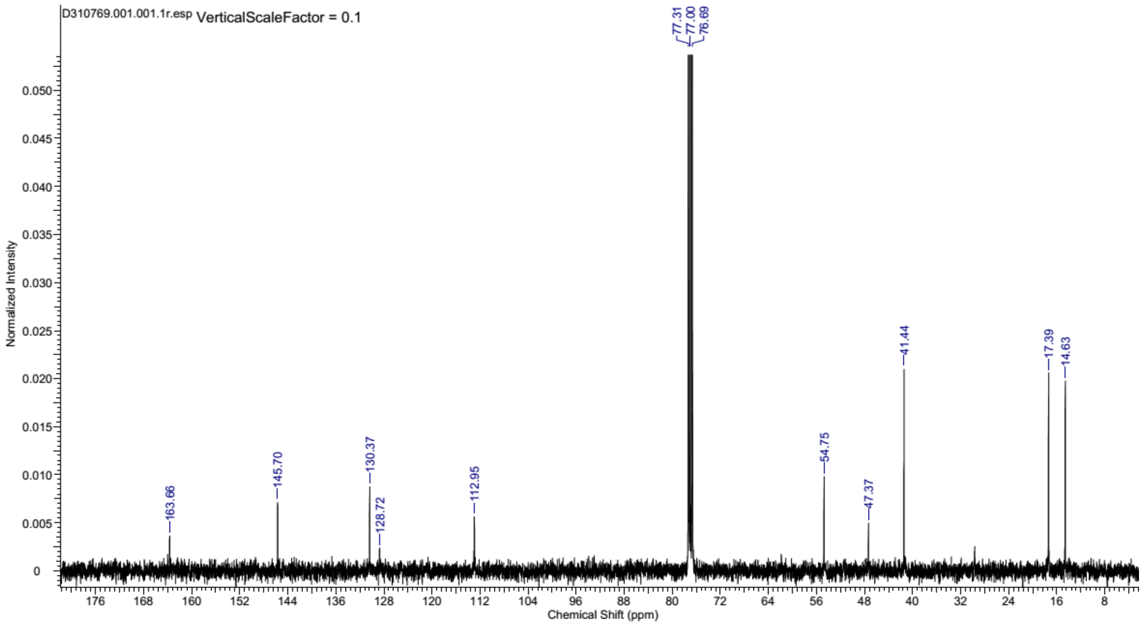

D310769.001.001.1r.esp VerticalScaleFactor = 0.1
Normalized Intensity
Chemical Shift (ppm)
163.66
145.70
130.37
128.72
112.95
77.31
77.00
76.69
54.75
47.37
41.44
17.39
14.63

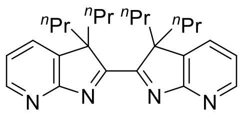
nPr
nPr
nPr
nPr
N
N
N
N

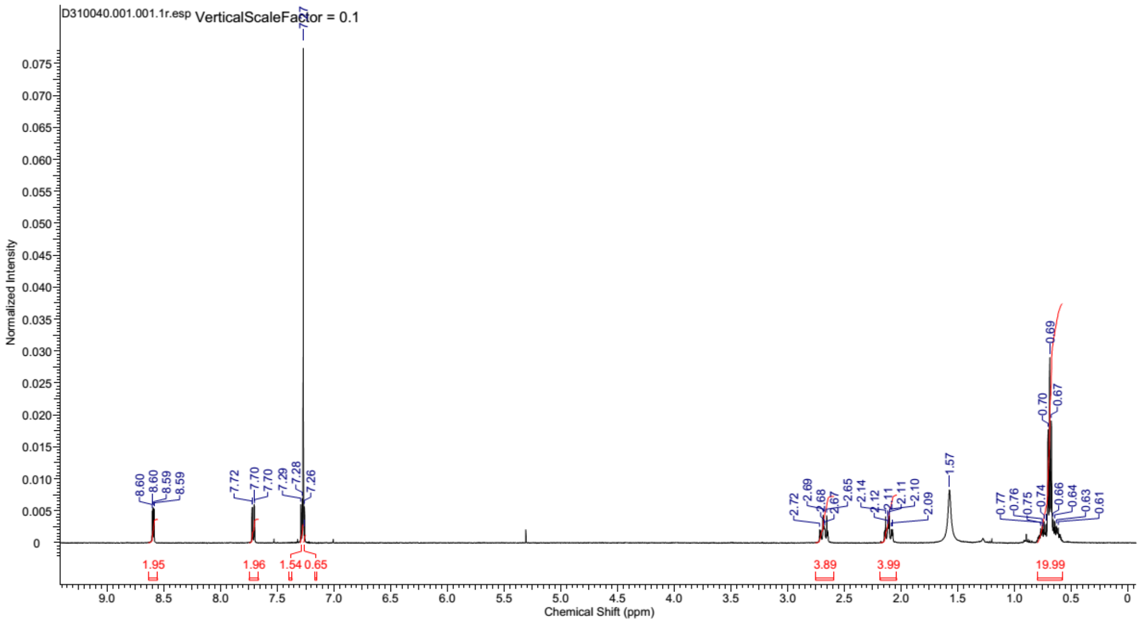
D310040.001.001.1r.esp VerticalScaleFactor = 0.1
Normalized Intensity
Chemical Shift (ppm)
8.60 8.60 8.59 8.59
7.72 7.70 7.70 7.29 7.28 7.27 7.26
2.72 2.69 2.68 2.67 2.65
2.14 2.12 2.11 2.11 2.10 2.09
1.57
0.77 0.76 0.75 0.74 0.70 0.69 0.67 0.66 0.64 0.63 0.61
1.95
1.96
1.54
0.65
3.89
3.99
19.99

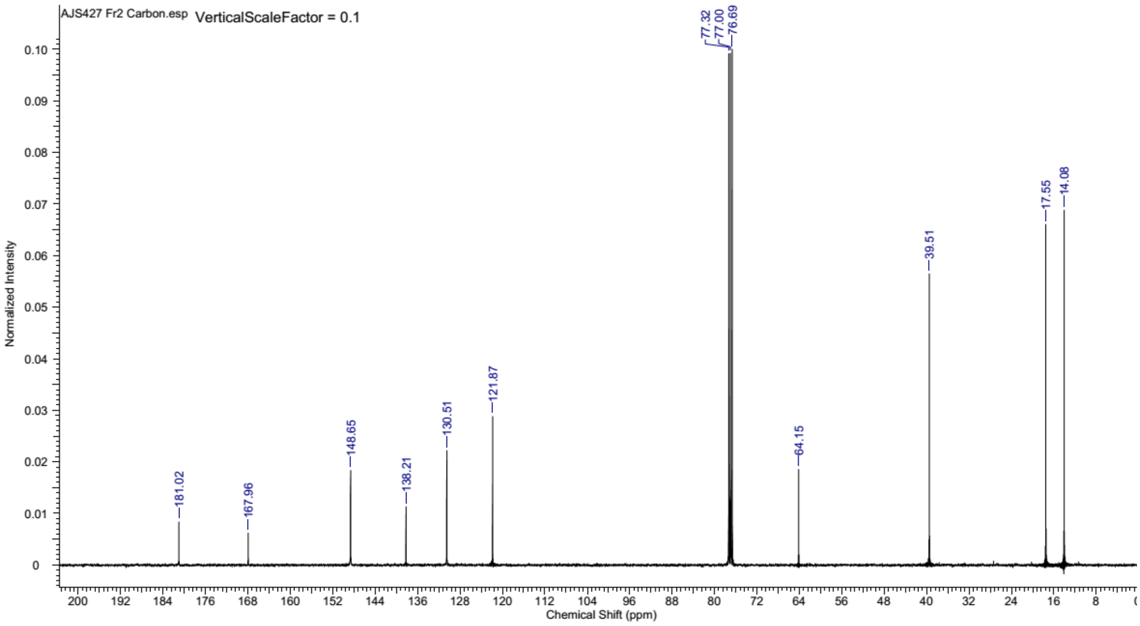
AJS427 Fr2 Carbon.esp VerticalScaleFactor = 0.1
Normalized Intensity
Chemical Shift (ppm)
181.02
167.96
148.65
138.21
130.51
121.87
77.32
77.00
76.69
64.15
39.51
17.55
14.08

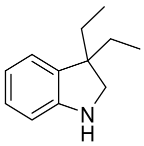

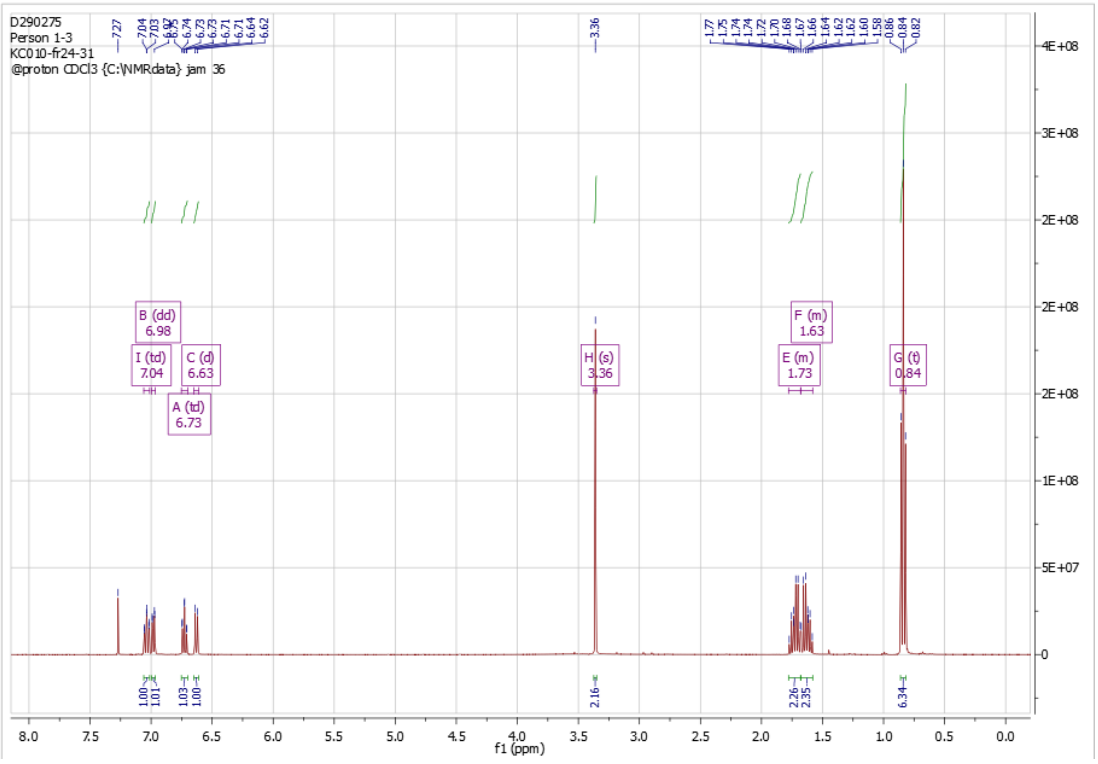
D290275
Person 1-3
KC010-fr24-31
@proton CDCl3 {C:\NMRdata} jam 36
B (dd) 6.98
I (td) 7.04
C (d) 6.63
A (td) 6.73
H (s) 3.36
F (m) 1.63
E (m) 1.73
G (t) 0.84
f1 (ppm)

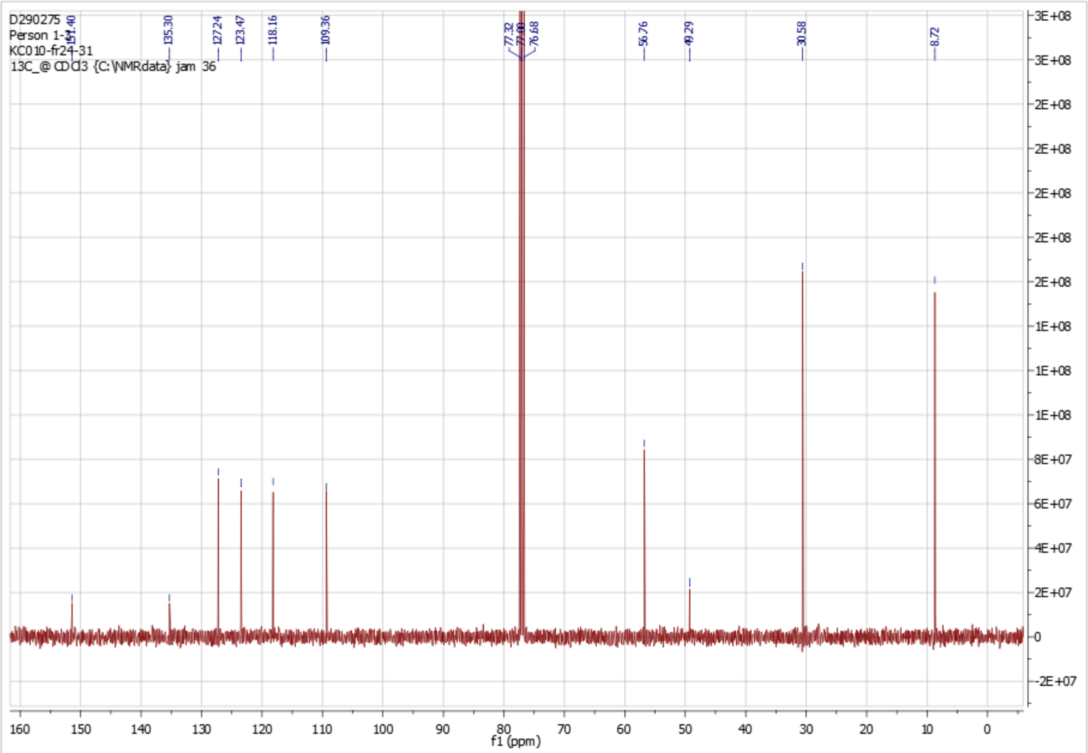
D290275
Person 1-3
KC010-fr24-31
13C_@ CDCl3 {C:\NMRdata} jam 36
f1 (ppm)

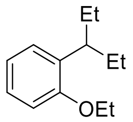
Et
Et
OEt

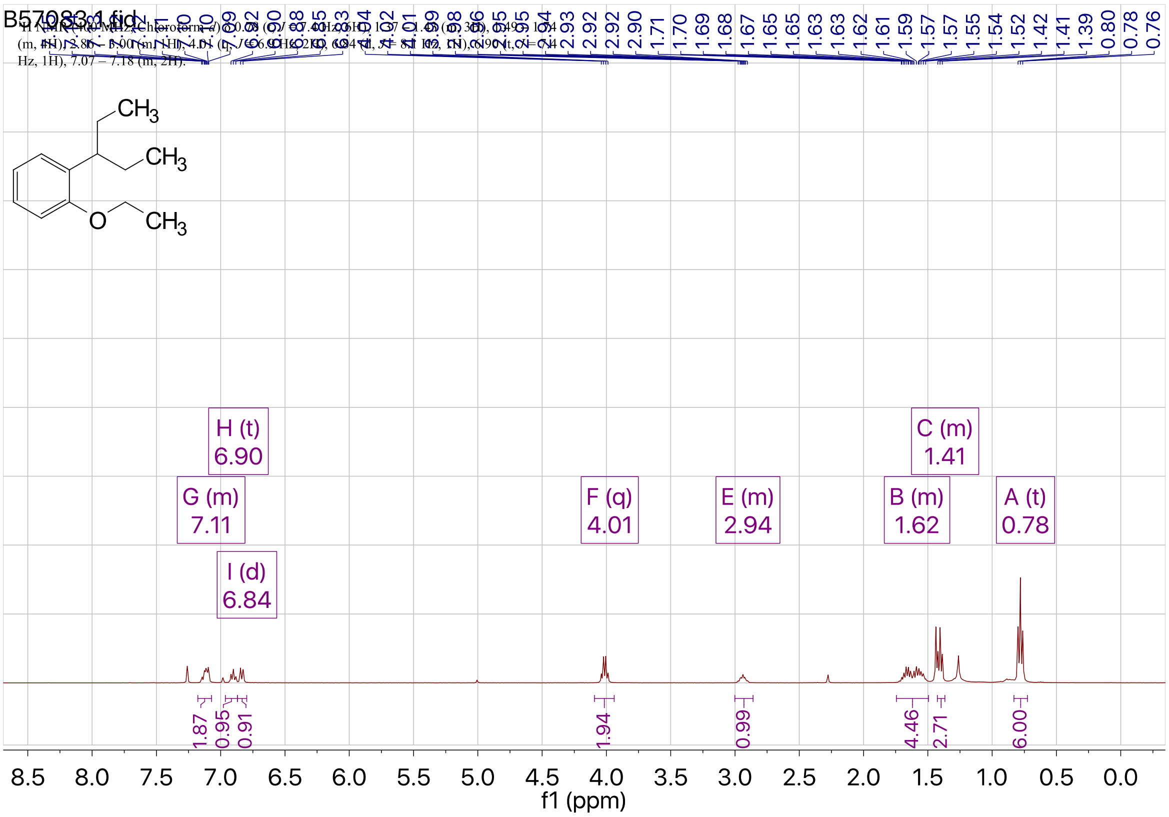
B57083.1.fid
CH3
CH3
O
CH3
H (t)
6.90
G (m)
7.11
I (d)
6.84
F (q)
4.01
E (m)
2.94
C (m)
1.41
B (m)
1.62
A (t)
0.78
1.87
0.95
0.91
1.94
0.99
4.46
2.71
6.00
f1 (ppm)

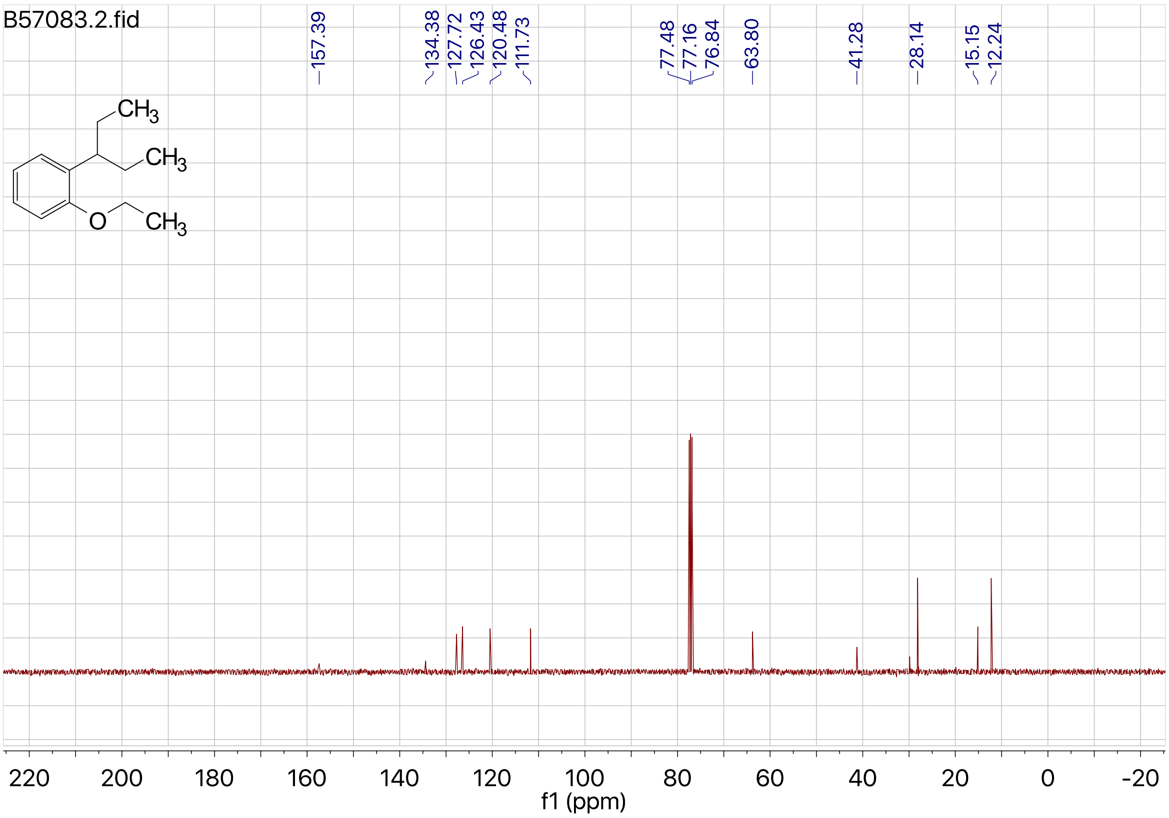
B57083.2.fid
157.39
134.38
127.72
126.43
120.48
111.73
77.48
77.16
76.84
63.80
41.28
28.14
15.15
12.24
CH3
CH3
O
CH3
f1 (ppm)

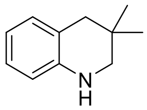
N
H

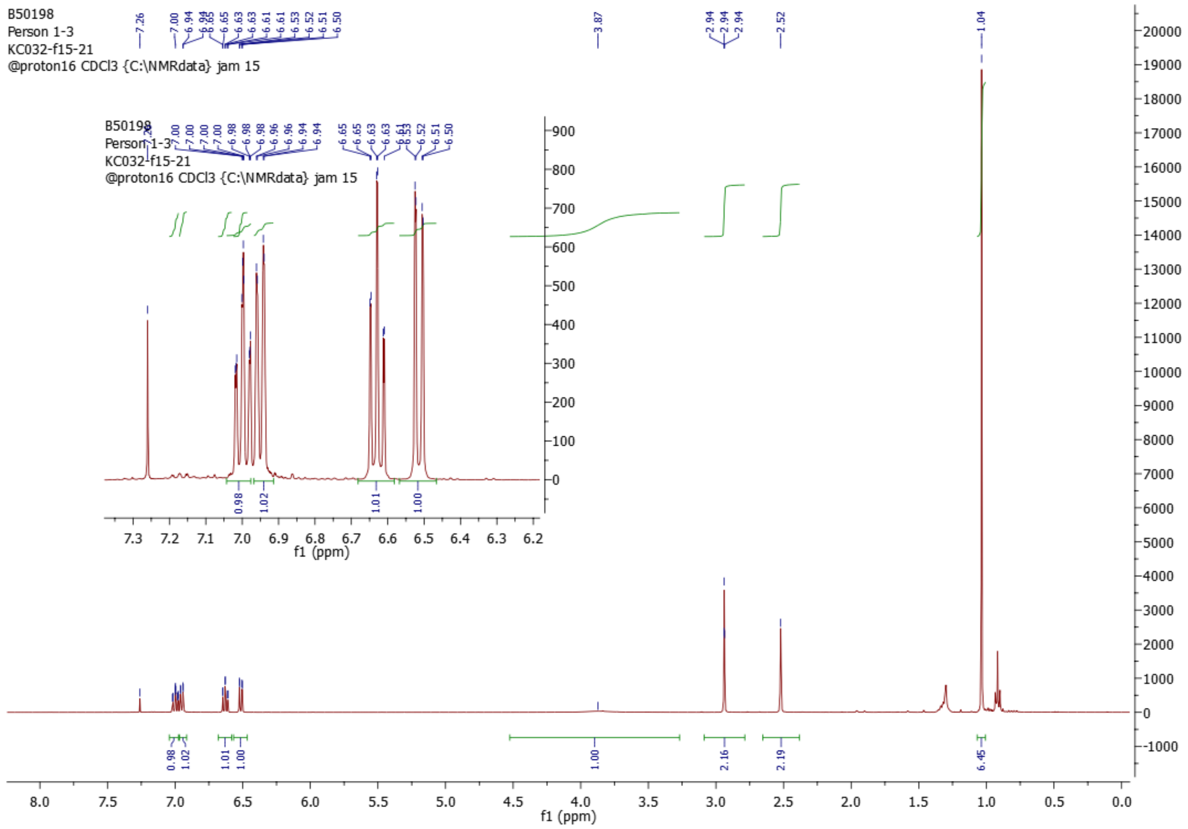
B50198
Person 1-3
KC032-f15-21
@proton16 CDCl3 {C:\NMRdata} jam 15
7.26
7.00
6.94
6.65
6.63
6.61
6.53
6.52
6.51
6.50
3.87
2.94
2.94
2.94
2.52
1.04
0.98
1.02
1.01
1.00
1.00
2.16
2.19
6.45
f1 (ppm)

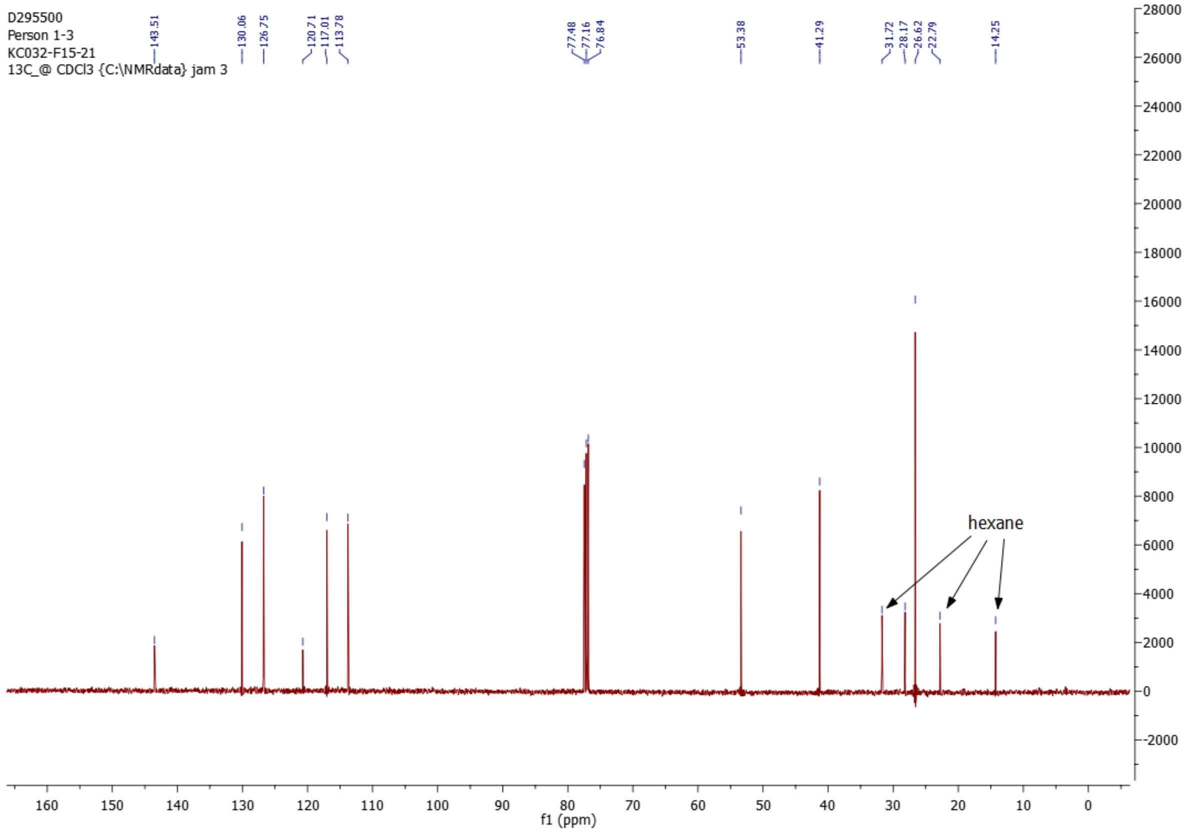
D295500
Person 1-3
KC032-F15-21
13C_@ CDCl3 {C:\NMRdata} jam 3
143.51
130.06
126.75
120.71
117.01
113.78
77.48
77.16
76.84
53.38
41.29
31.72
28.17
26.62
22.79
14.25
hexane
f1 (ppm)

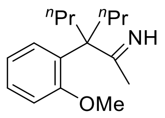

nPr
nPr
NH
OMe

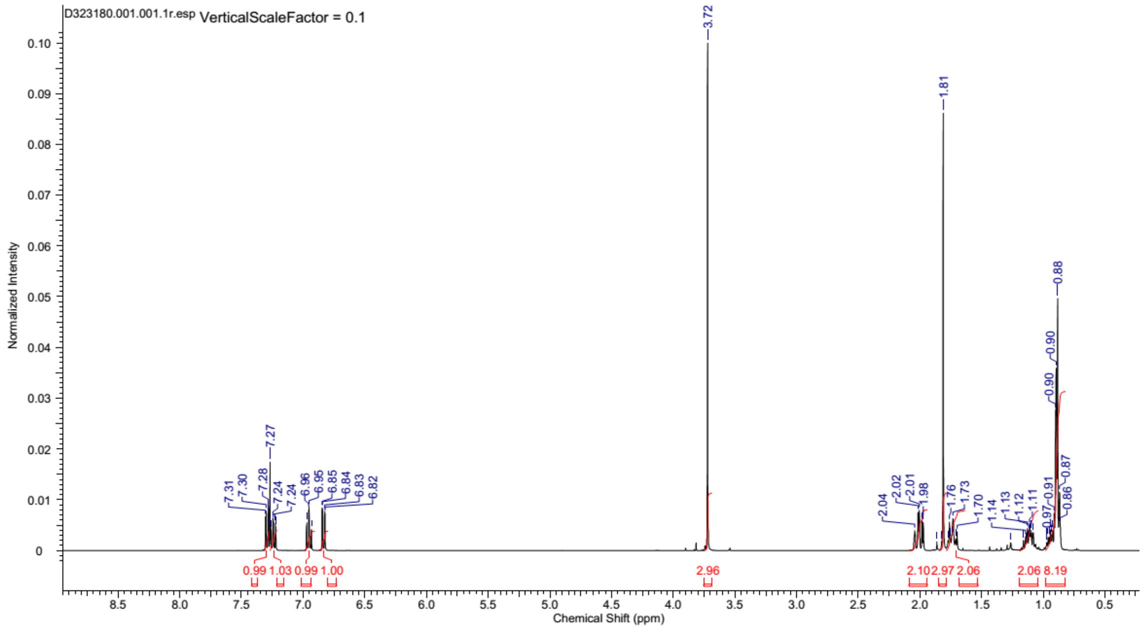

D323180.001.001.1r.esp VerticalScaleFactor = 0.1
Normalized Intensity
Chemical Shift (ppm)
7.31
7.30
7.28
7.27
7.24
7.24
6.96
6.95
6.85
6.84
6.83
6.82
3.72
2.04
2.02
2.01
1.98
1.81
1.76
1.73
1.70
1.14
1.13
1.12
1.11
0.97
0.91
0.90
0.90
0.88
0.87
0.86
0.99
1.03
0.99
1.00
2.96
2.10
2.97
2.06
2.06
8.19

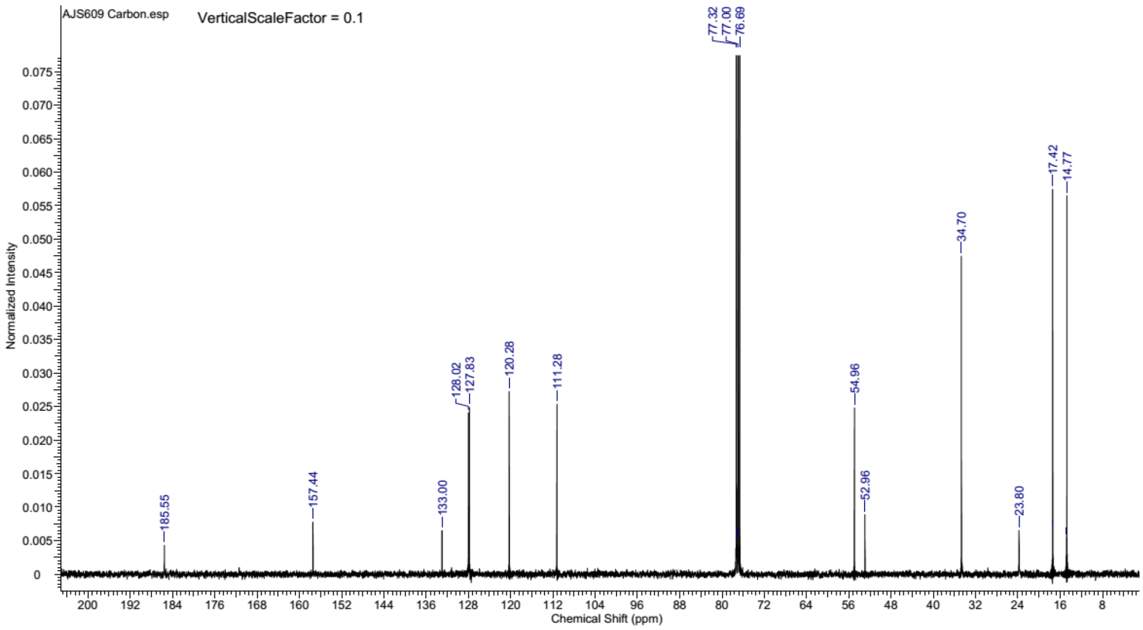

AJS609 Carbon.esp
VerticalScaleFactor = 0.1
Normalized Intensity
Chemical Shift (ppm)
185.55
157.44
133.00
128.02
127.83
120.28
111.28
77.32
77.00
76.69
54.96
52.96
34.70
23.80
17.42
14.77

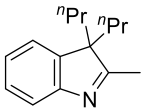

nPr nPr
N

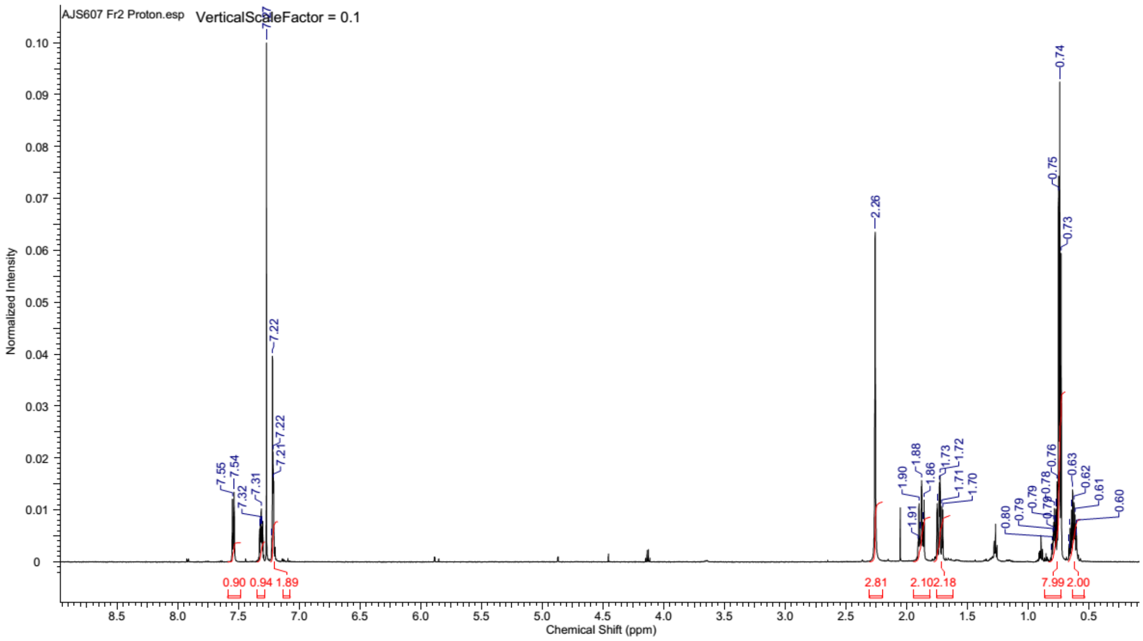

AJS607 Fr2 Proton.esp
VerticalScaleFactor = 0.1
Normalized Intensity
Chemical Shift (ppm)
7.27
7.22
7.22
7.21
7.55
7.54
7.32
7.31
2.26
1.91
1.90
1.88
1.86
1.73
1.72
1.71
1.70
0.80
0.79
0.79
0.78
0.76
0.75
0.74
0.73
0.63
0.62
0.61
0.60
0.90
0.94
1.89
2.81
2.10
2.18
7.99
2.00

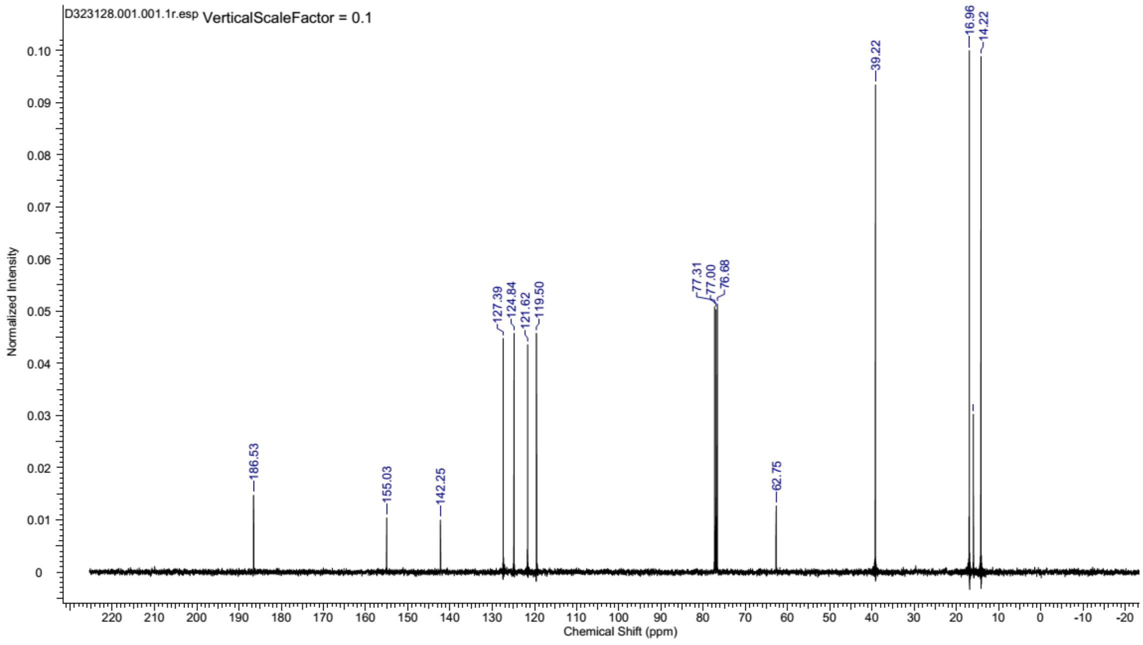

D323128.001.001.1r.esp
VerticalScaleFactor = 0.1
Normalized Intensity
Chemical Shift (ppm)
186.53
155.03
142.25
127.39
124.84
121.62
119.50
77.31
77.00
76.68
62.75
39.22
16.96
14.22

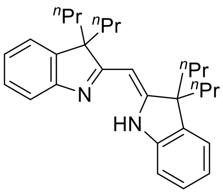
^{n}Pr ^{n}Pr
^{n}Pr
^{n}Pr
N
HN

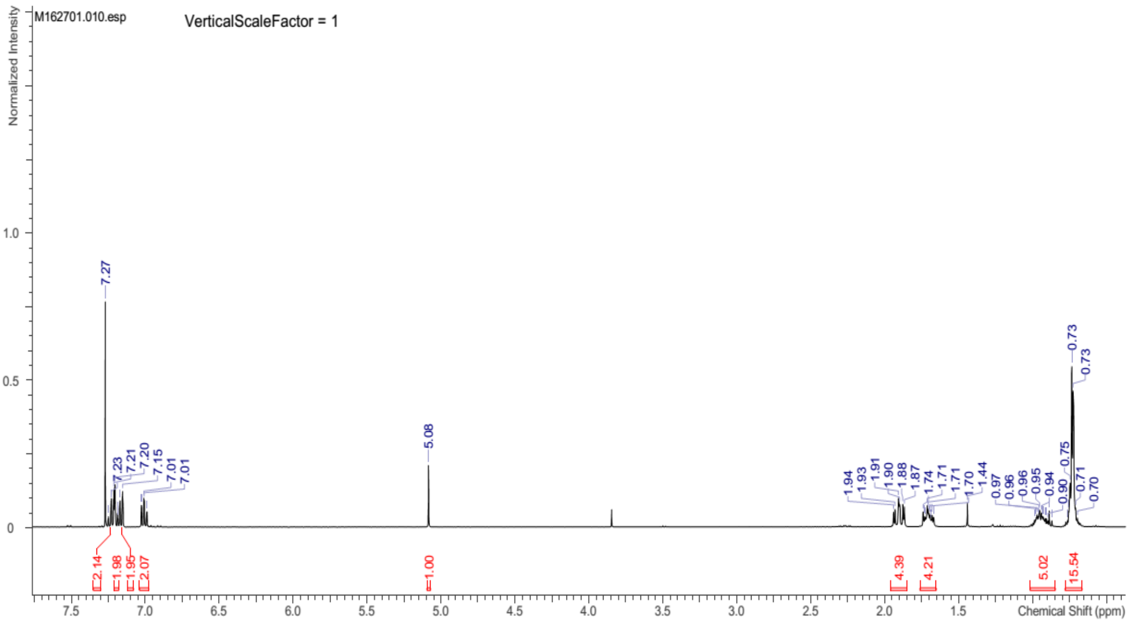
M162701.010.esp
VerticalScaleFactor = 1
Normalized Intensity
7.27
7.23
7.21
7.20
7.15
7.01
7.01
5.08
1.94
1.93
1.91
1.90
1.88
1.87
1.74
1.71
1.71
1.70
1.44
0.97
0.96
0.96
0.95
0.94
0.90
0.75
0.73
0.73
0.71
0.70
2.14
1.98
1.95
2.07
1.00
4.39
4.21
5.02
15.54
Chemical Shift (ppm)

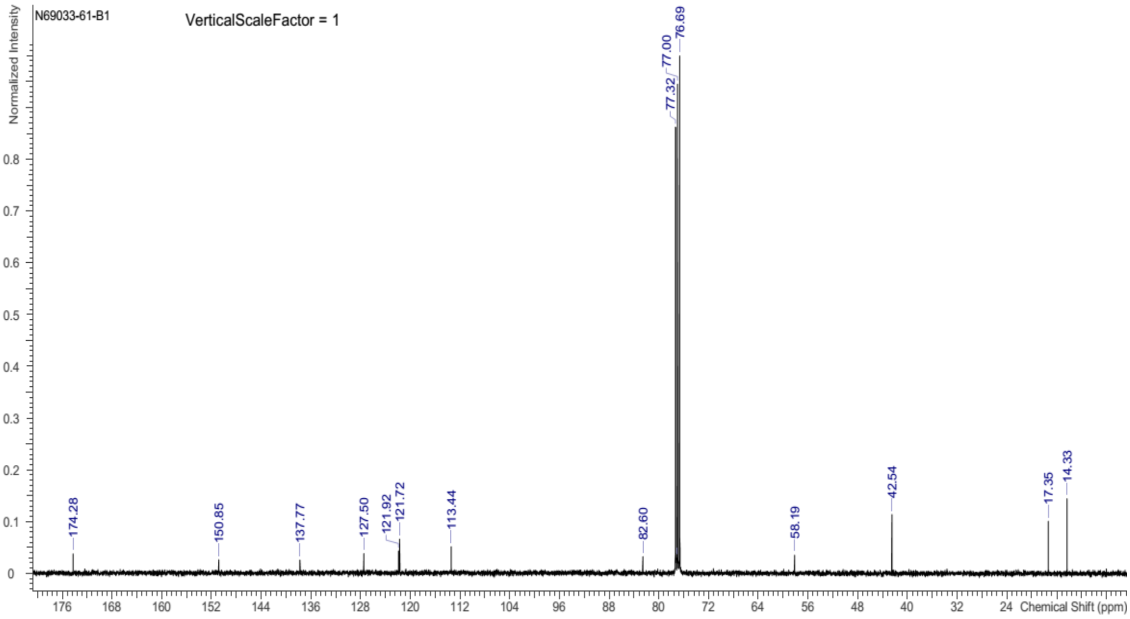
N69033-61-B1
VerticalScaleFactor = 1
Normalized Intensity
174.28
150.85
137.77
127.50
121.92
121.72
113.44
82.60
77.32
77.00
76.69
58.19
42.54
17.35
14.33
Chemical Shift (ppm)

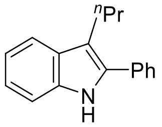

^{n}Pr
Ph
N
H

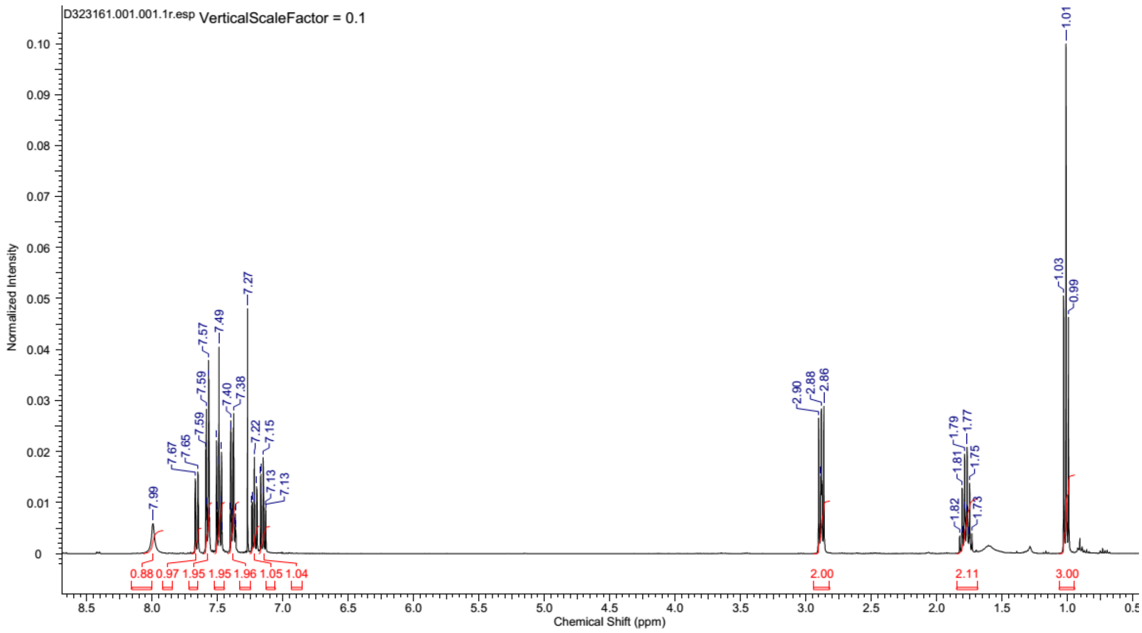

D323161.001.001.1r.esp VerticalScaleFactor = 0.1
Normalized Intensity
Chemical Shift (ppm)
7.99
7.67
7.65
7.59
7.59
7.57
7.49
7.40
7.38
7.27
7.22
7.15
7.13
7.13
2.90
2.88
2.86
1.82
1.81
1.79
1.77
1.75
1.73
1.03
1.01
0.99
0.88
0.97
1.95
1.95
1.96
1.05
1.04
2.00
2.11
3.00

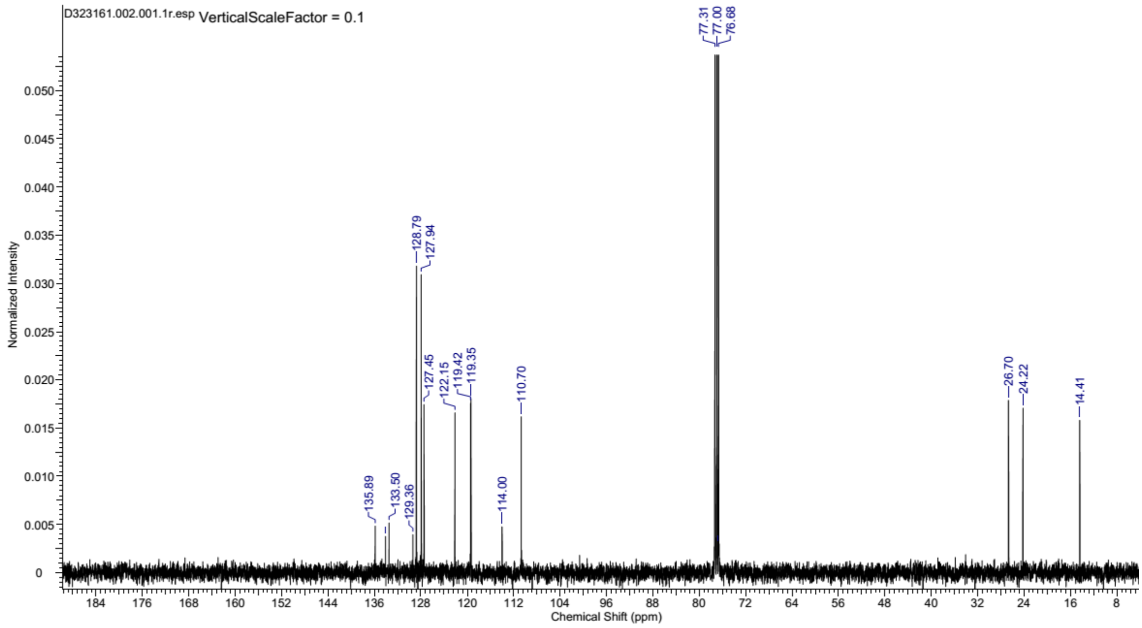

D323161.002.001.1r.esp VerticalScaleFactor = 0.1
Normalized Intensity
Chemical Shift (ppm)
135.89
133.50
129.36
128.79
127.94
127.45
122.15
119.42
119.35
114.00
110.70
77.31
77.00
76.68
26.70
24.22
14.41

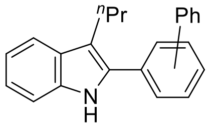

nPr
Ph
N
H

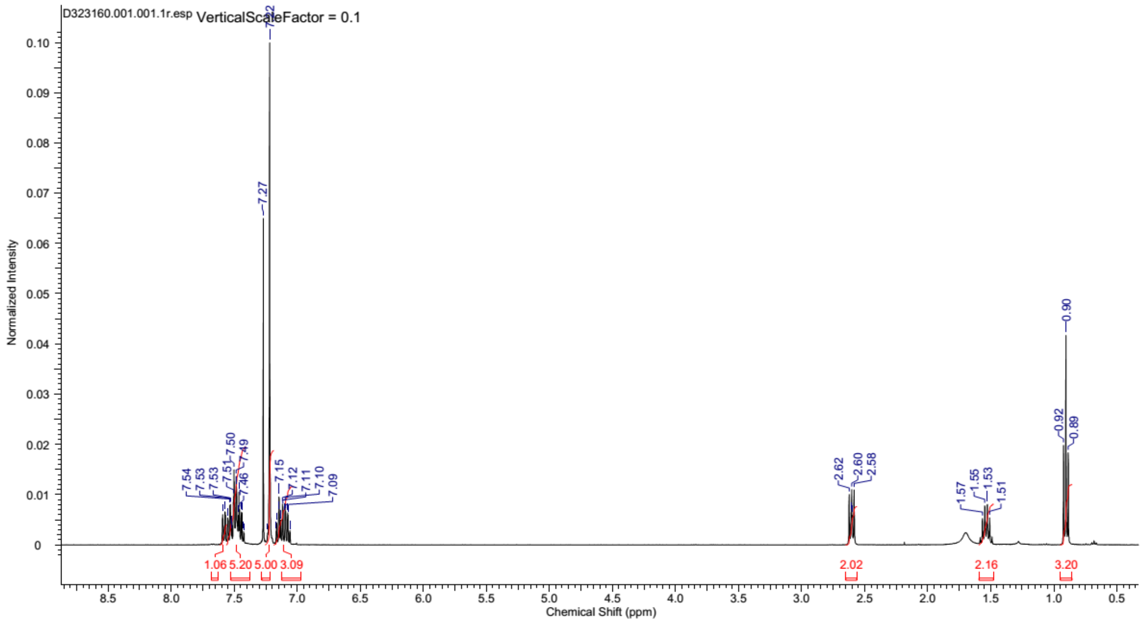

D323160.001.001.1r.esp VerticalScaleFactor = 0.1
Normalized Intensity
Chemical Shift (ppm)

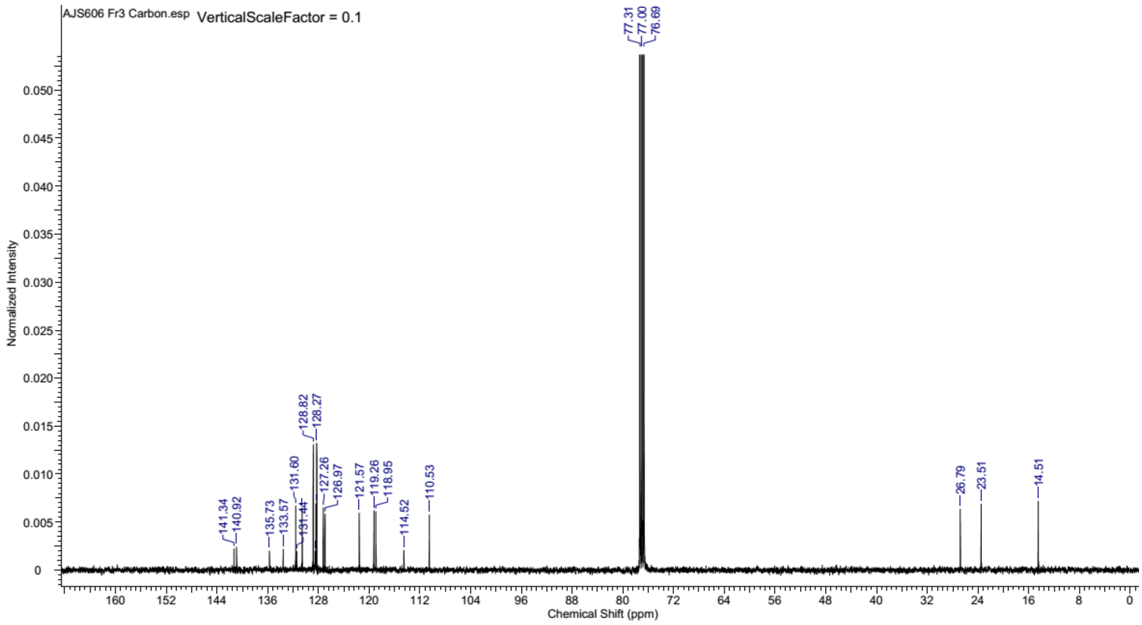

AJS606 Fr3 Carbon.esp VerticalScaleFactor = 0.1
Normalized Intensity
Chemical Shift (ppm)

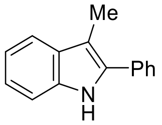
Me
Ph
N
H

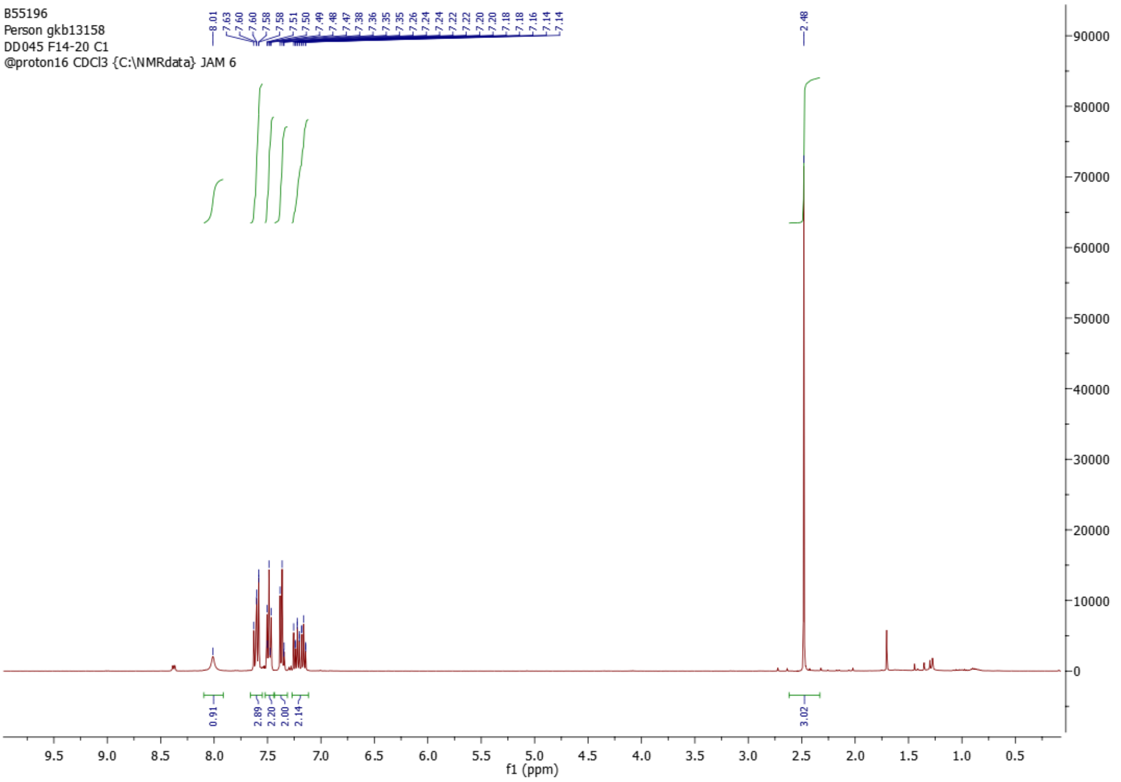
B55196
Person gkb13158
DD045 F14-20 C1
@proton16 CDCl3 {C:\NMRdata} JAM 6
8.01
7.63
7.60
7.60
7.58
7.58
7.51
7.50
7.49
7.48
7.47
7.38
7.36
7.35
7.35
7.26
7.24
7.24
7.22
7.22
7.20
7.20
7.18
7.18
7.16
7.14
7.14
2.48
0.91
2.89
2.20
2.00
2.14
3.02
f1 (ppm)

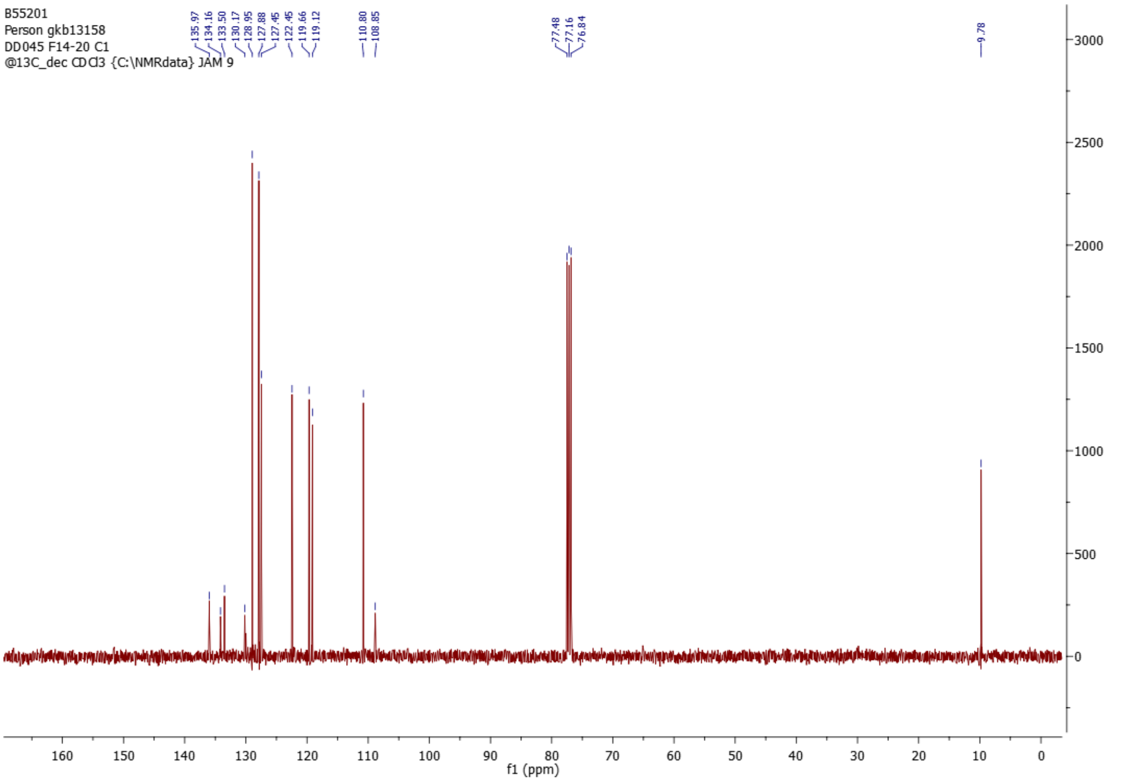
B55201
Person gkb13158
DD045 F14-20 C1
@13C_dec CDCl3 {C:\NMRdata} JAM 9
135.97
134.16
133.50
130.17
128.95
127.88
127.45
122.45
119.66
119.12
110.80
108.85
77.48
77.16
76.84
9.78
f1 (ppm)

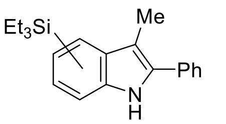

mixture of 2 isomers

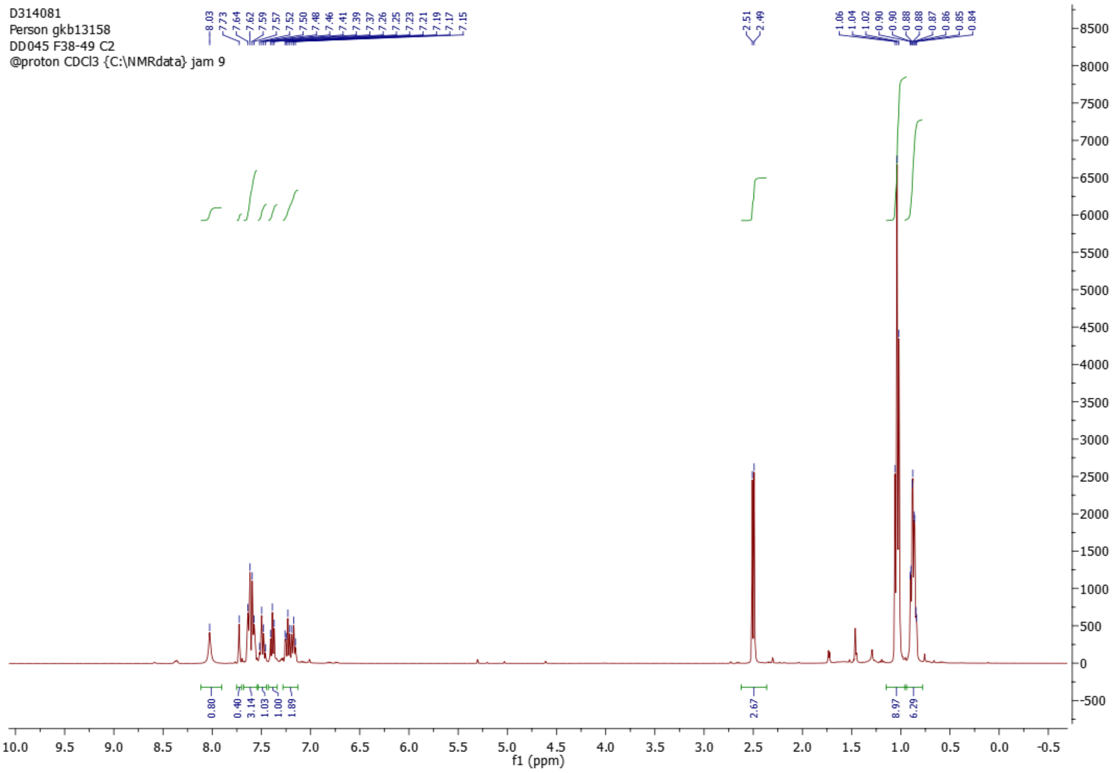

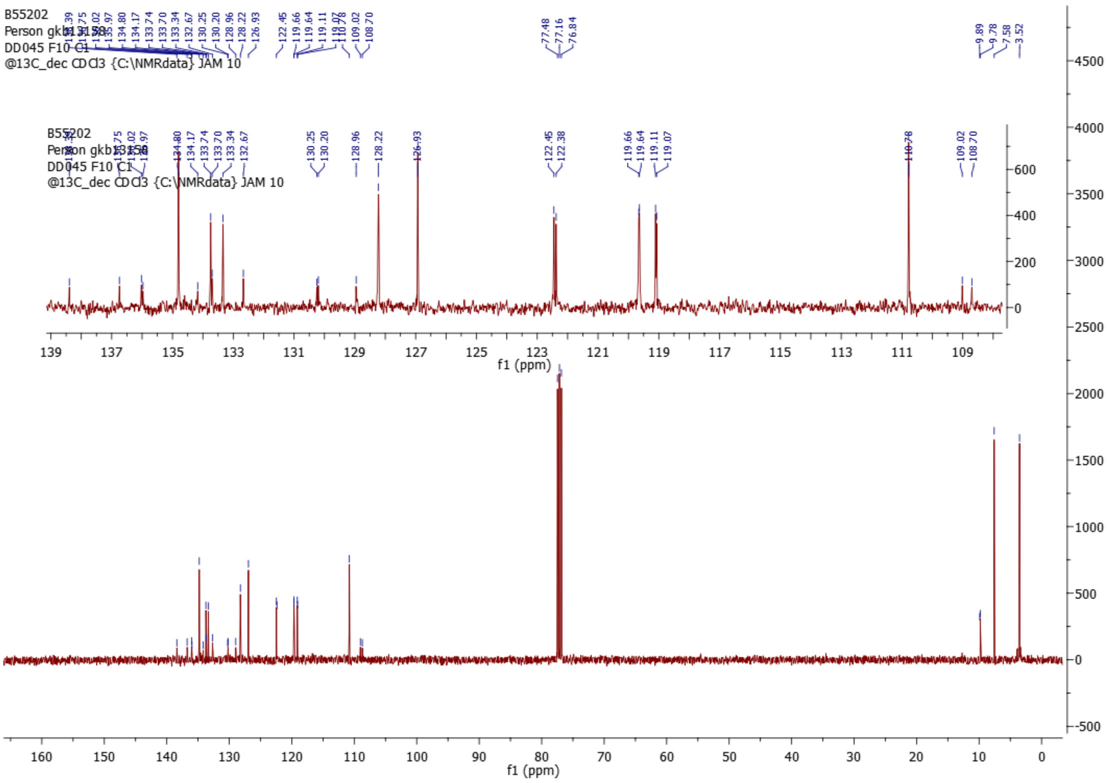

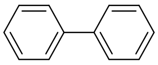

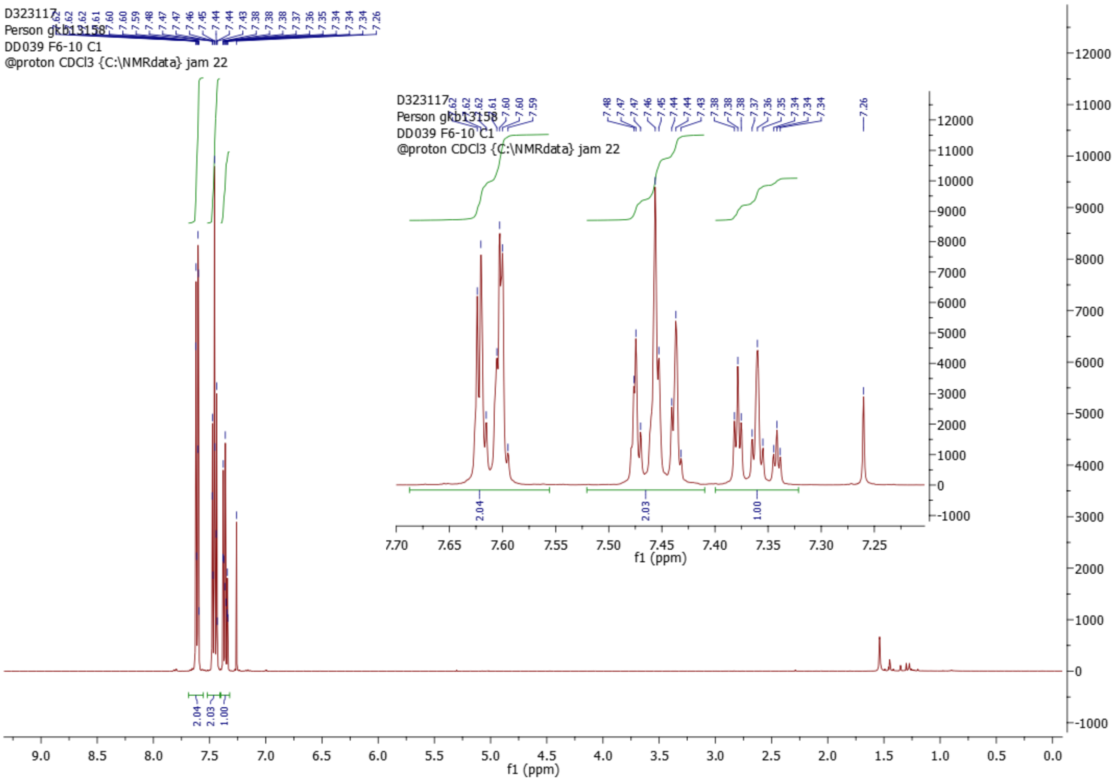
D323117
Person gkb13158
DD039 F6-10 C1
@proton CDCl3 {C:\NMRdata} jam 22
7.62
7.62
7.62
7.61
7.60
7.60
7.59
7.48
7.47
7.47
7.46
7.45
7.44
7.44
7.43
7.38
7.38
7.38
7.37
7.36
7.35
7.34
7.34
7.34
7.34
7.26
2.04
2.03
1.00
f1 (ppm)

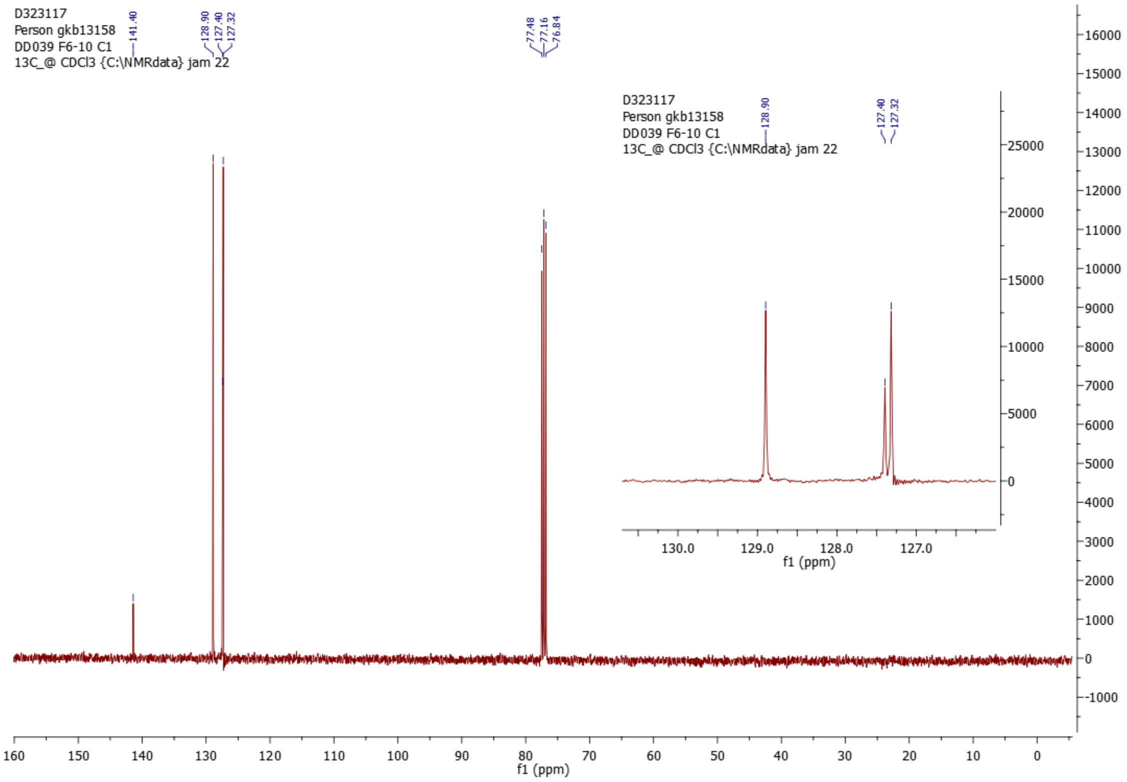
D323117
Person gkb13158
DD039 F6-10 C1
13C_@ CDCl3 {C:\NMRdata} jam 22
141.40
128.90
127.40
127.32
77.48
77.16
76.84
f1 (ppm)

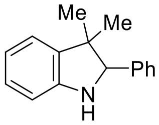

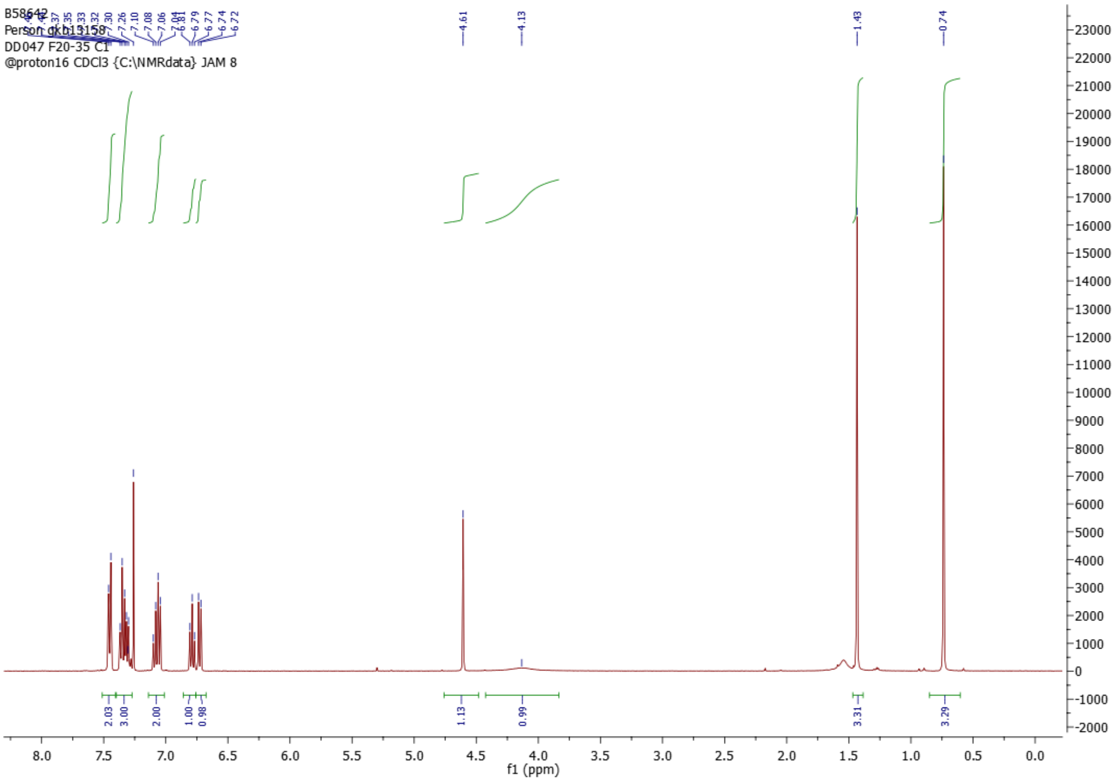

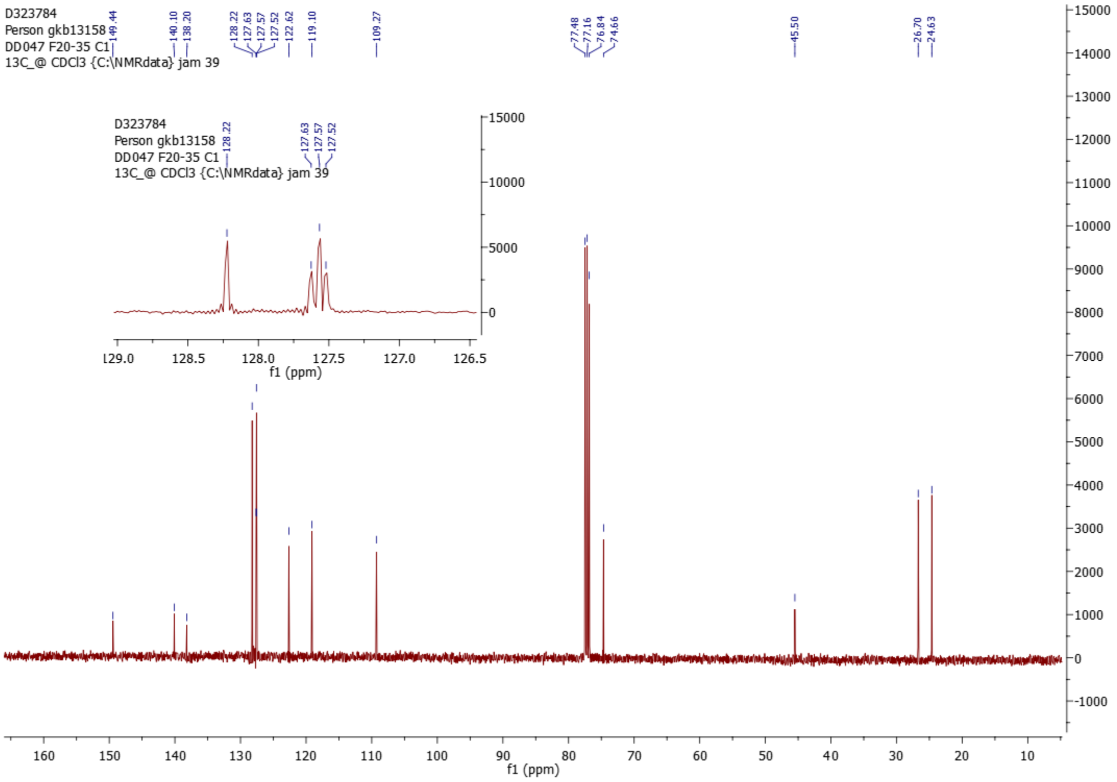

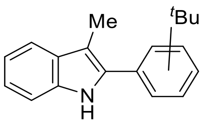
Me
tBu
N
H

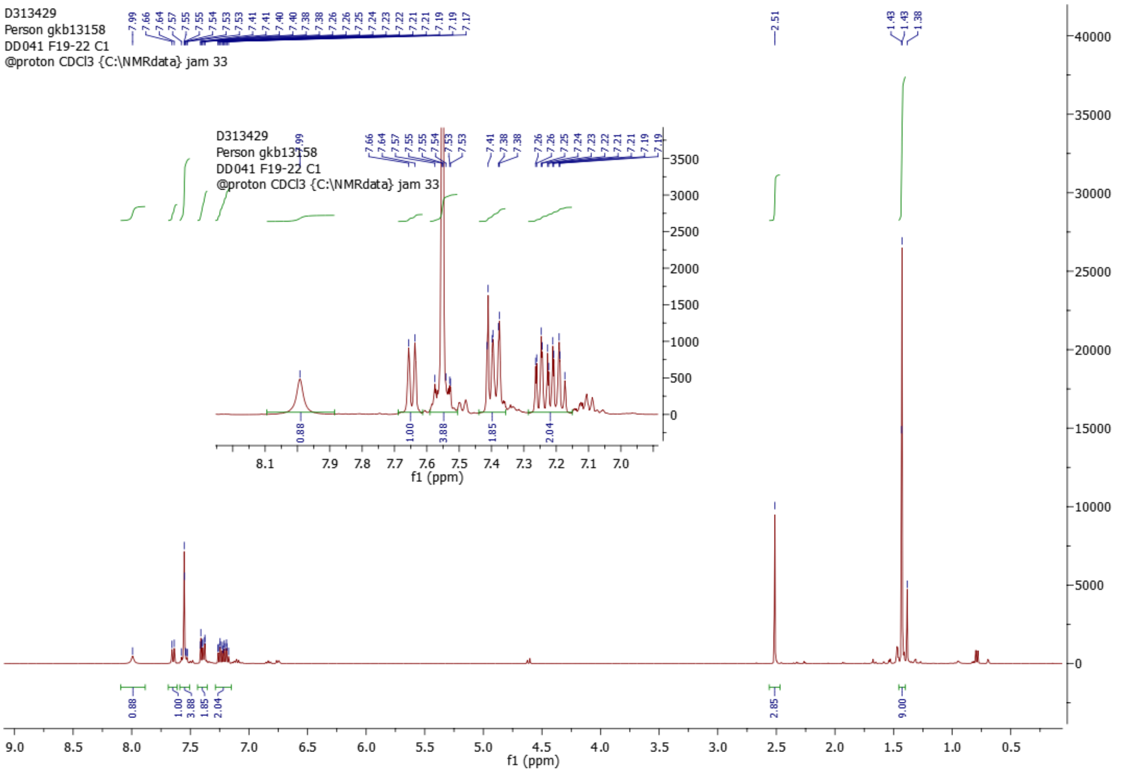
D313429
Person gkb13158
DD041 F19-22 C1
@proton CDCl3 {C:\NMRdata} jam 33
f1 (ppm)

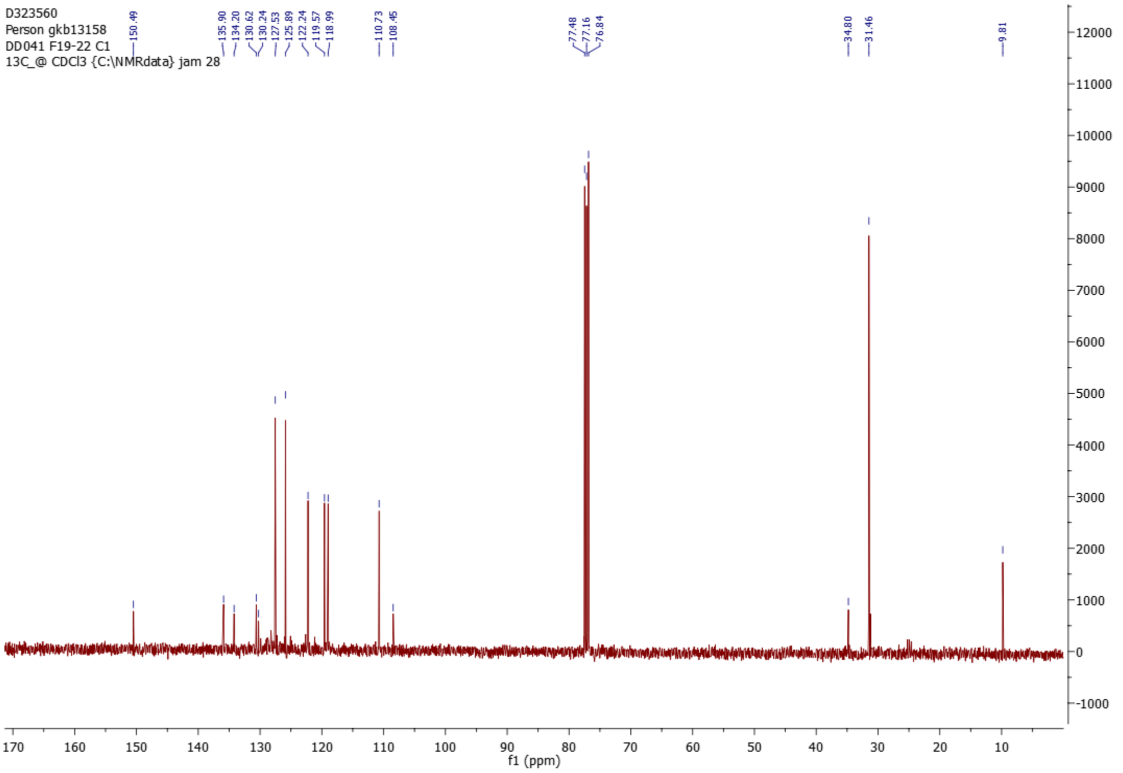
D323560
Person gkb13158
DD041 F19-22 C1
13C_@ CDCl3 {C:\NMRdata} jam 28
150.49
135.90
134.20
130.62
130.24
127.53
125.89
122.24
119.57
118.99
110.73
108.45
77.48
77.16
76.84
34.80
31.46
9.81
f1 (ppm)

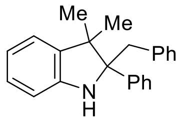

Me
Me
Ph
Ph
N
H

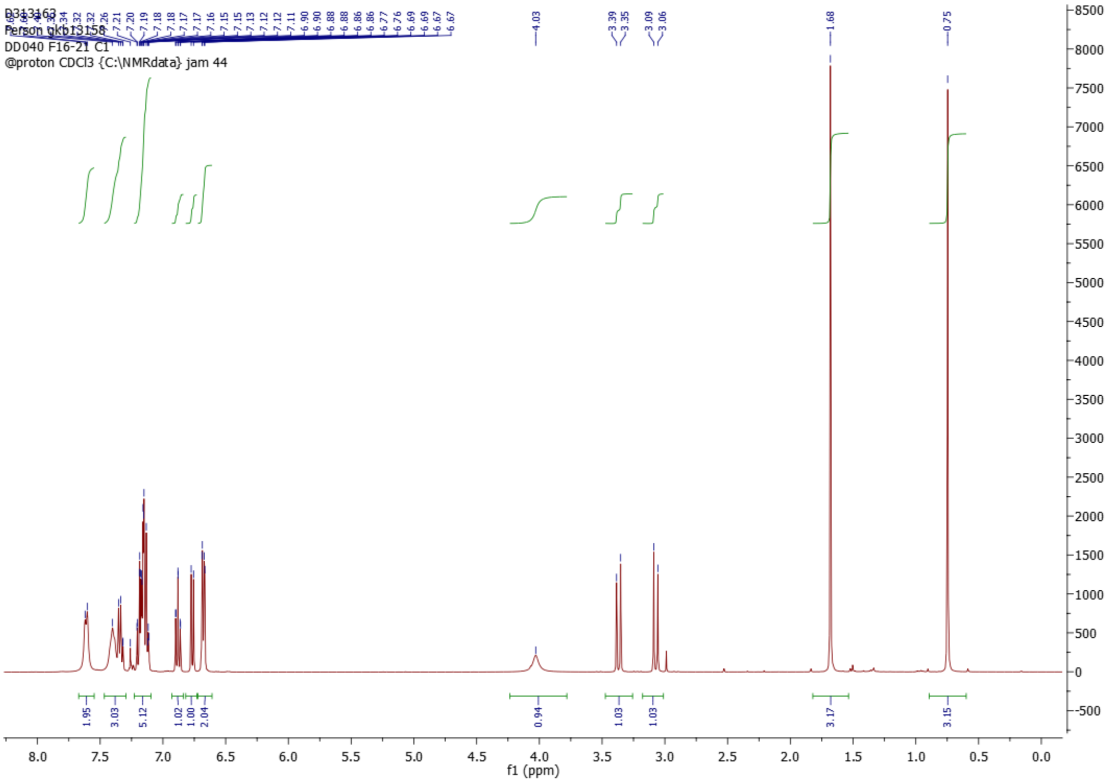

D313163
Person gkb13158
DD040 F16-21 C1
@proton CDCl3 {C:\NMRdata} jam 44
4.03
3.39
3.35
3.09
3.06
1.68
0.75
1.95
3.03
5.12
1.02
1.00
2.04
0.94
1.03
1.03
3.17
3.15
f1 (ppm)

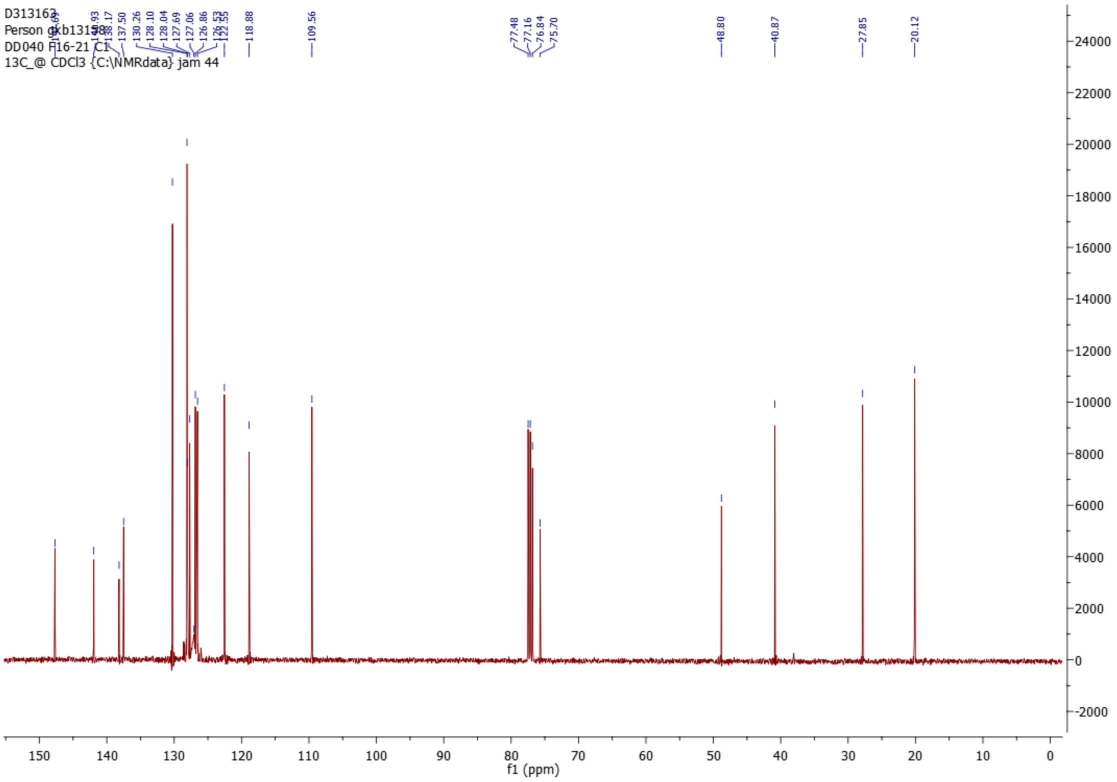

D313163
Person gkb13158
DD040 F16-21 C1
13C_@ CDCl3 {C:\NMRdata} jam 44
118.88
109.56
77.48
77.16
76.84
75.70
48.80
40.87
27.85
20.12
f1 (ppm)

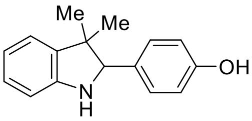
Me
Me
OH
N
H

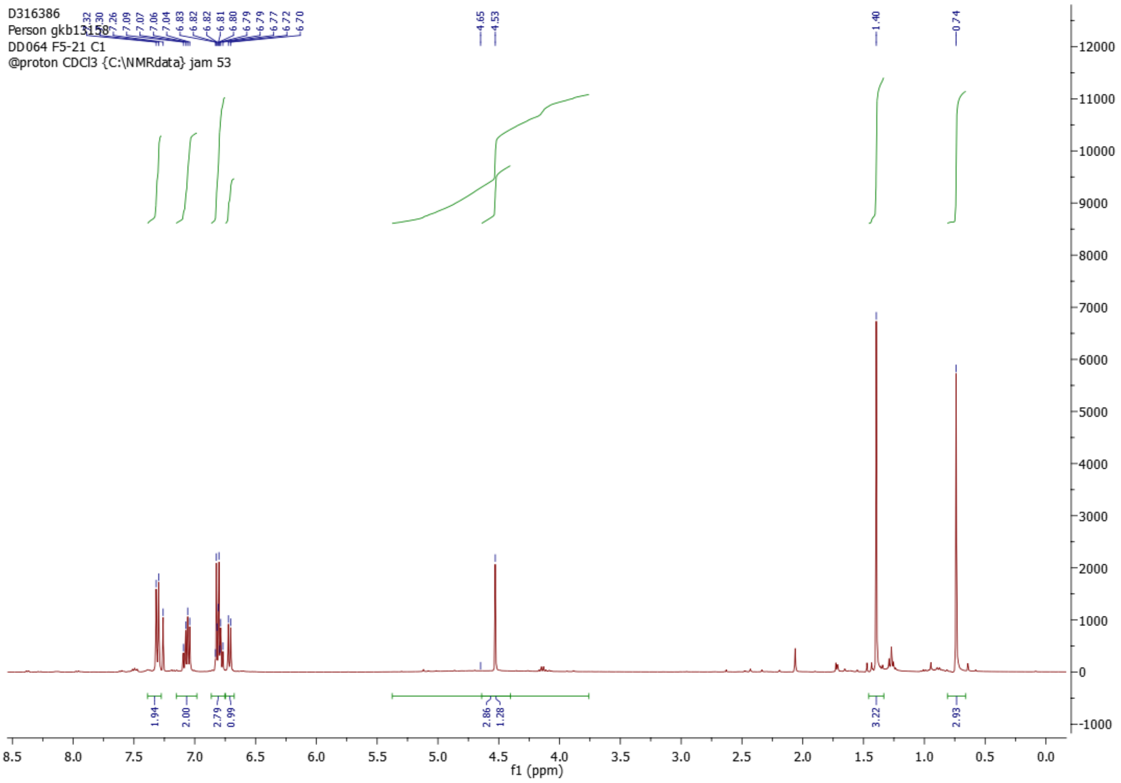
D316386
Person gkb13158
DD064 F5-21 C1
@proton CDCl3 {C:\NMRdata} jam 53
7.32
7.30
7.26
7.09
7.07
7.06
7.04
6.83
6.82
6.82
6.81
6.80
6.79
6.79
6.77
6.72
6.70
4.65
4.53
1.40
0.74
1.94
2.00
2.79
0.99
2.86
1.28
3.22
2.93
f1 (ppm)

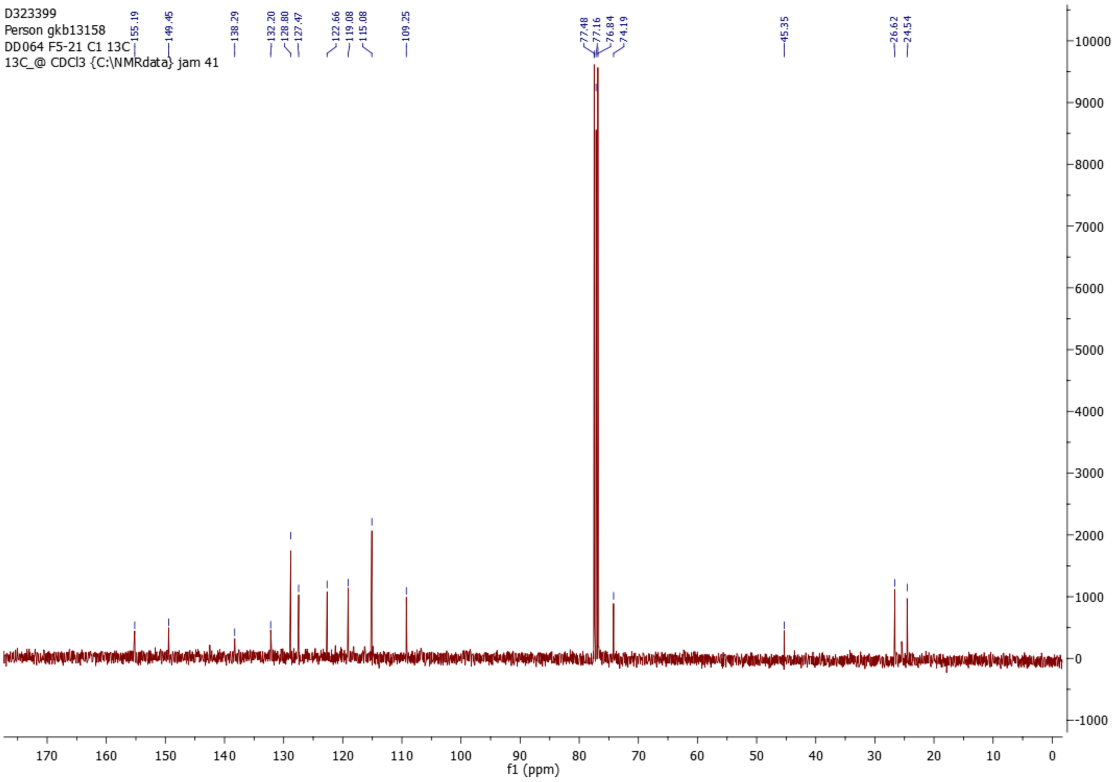
D323399
Person gkb13158
DD064 F5-21 C1 13C
13C_@ CDCl3 {C:\NMRdata} jam 41
155.19
149.45
138.29
132.20
128.80
127.47
122.66
119.08
115.08
109.25
77.48
77.16
76.84
74.19
45.35
26.62
24.54
f1 (ppm)

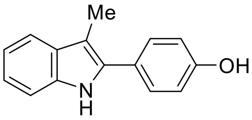

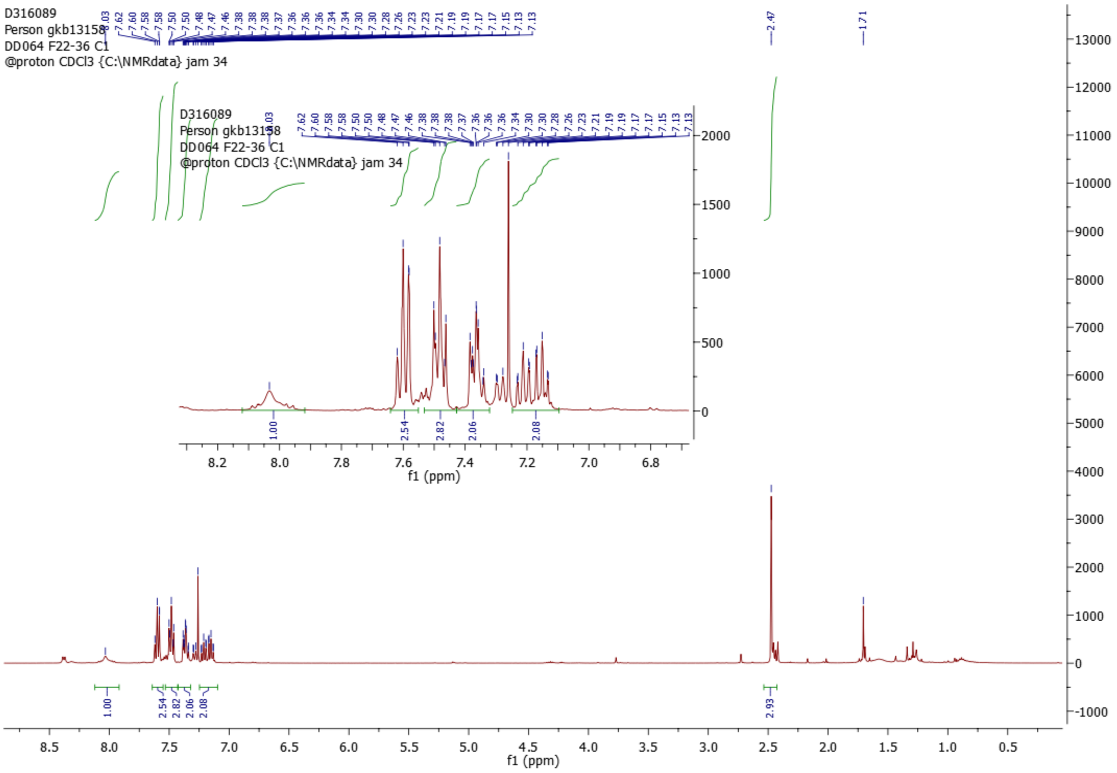
D316089
Person gkb13158
DD064 F22-36 C1
@proton CDCl3 {C:\NMRdata} jam 34
f1 (ppm)

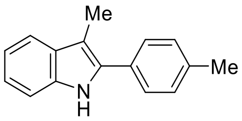
Me
N
H
Me

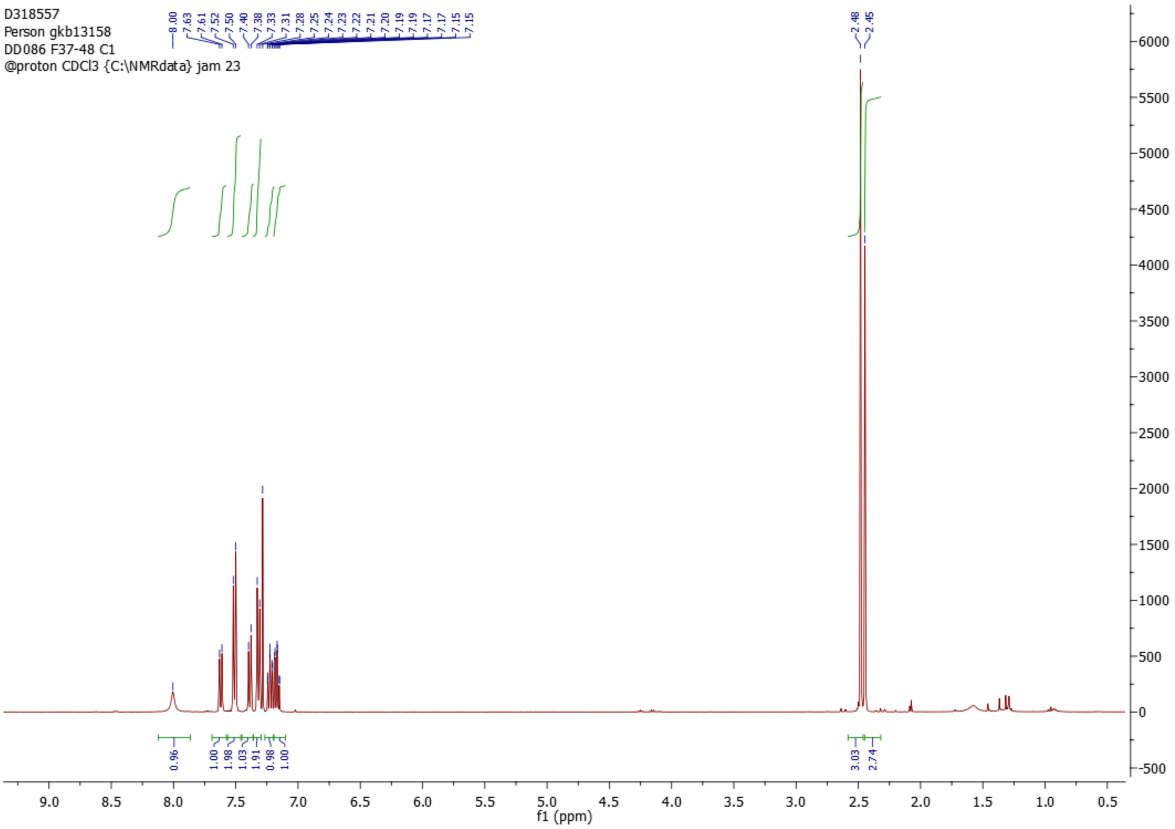
D318557
Person gkb13158
DD086 F37-48 C1
@proton CDCl3 {C:\NMRdata} jam 23
8.00
7.63
7.61
7.52
7.50
7.40
7.38
7.33
7.31
7.28
7.25
7.24
7.23
7.22
7.21
7.20
7.19
7.19
7.17
7.17
7.15
7.15
2.48
2.45
0.96
1.00
1.98
1.03
1.91
0.98
1.00
3.03
2.74
f1 (ppm)

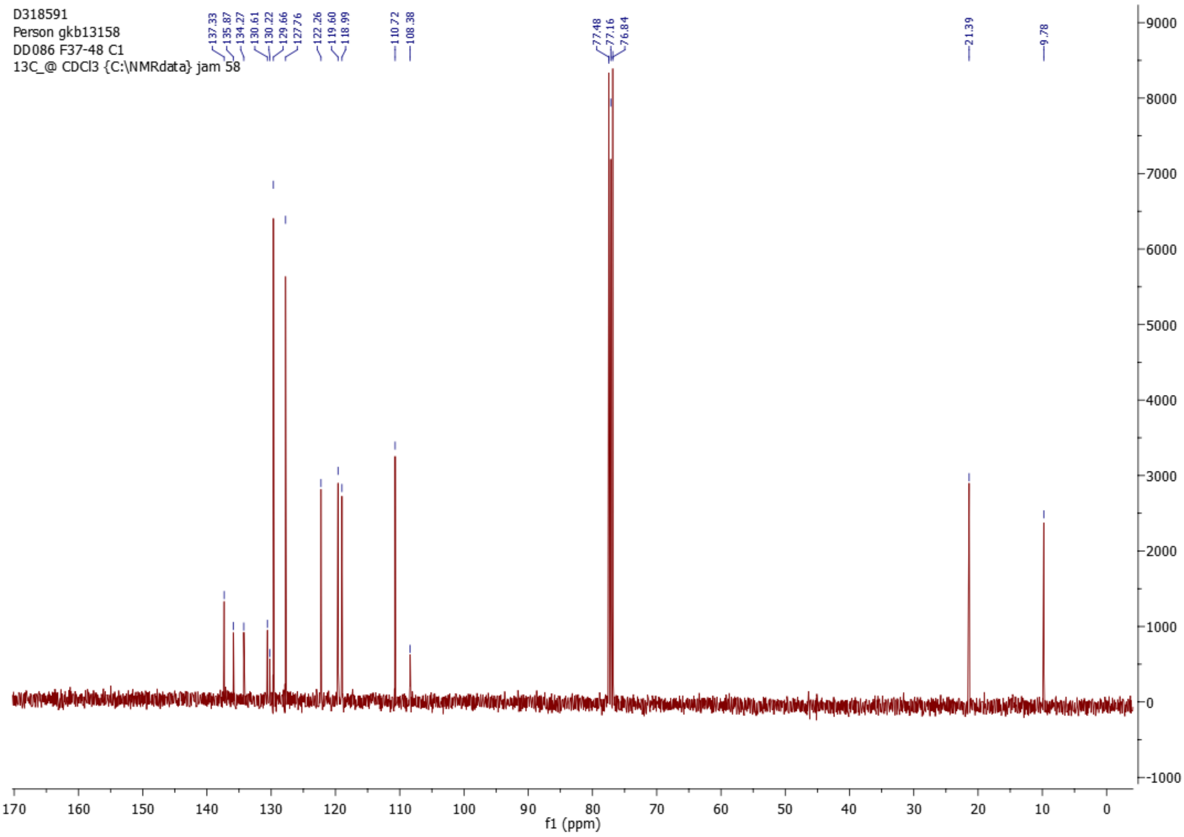
D318591
Person gkb13158
DD086 F37-48 C1
13C_@ CDCl3 {C:\NMRdata} jam 58
137.33
135.87
134.27
130.61
130.22
129.66
127.76
122.26
119.60
118.99
110.72
108.38
77.48
77.16
76.84
21.39
9.78
f1 (ppm)

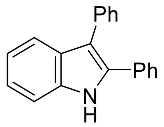
Ph
Ph
N
H

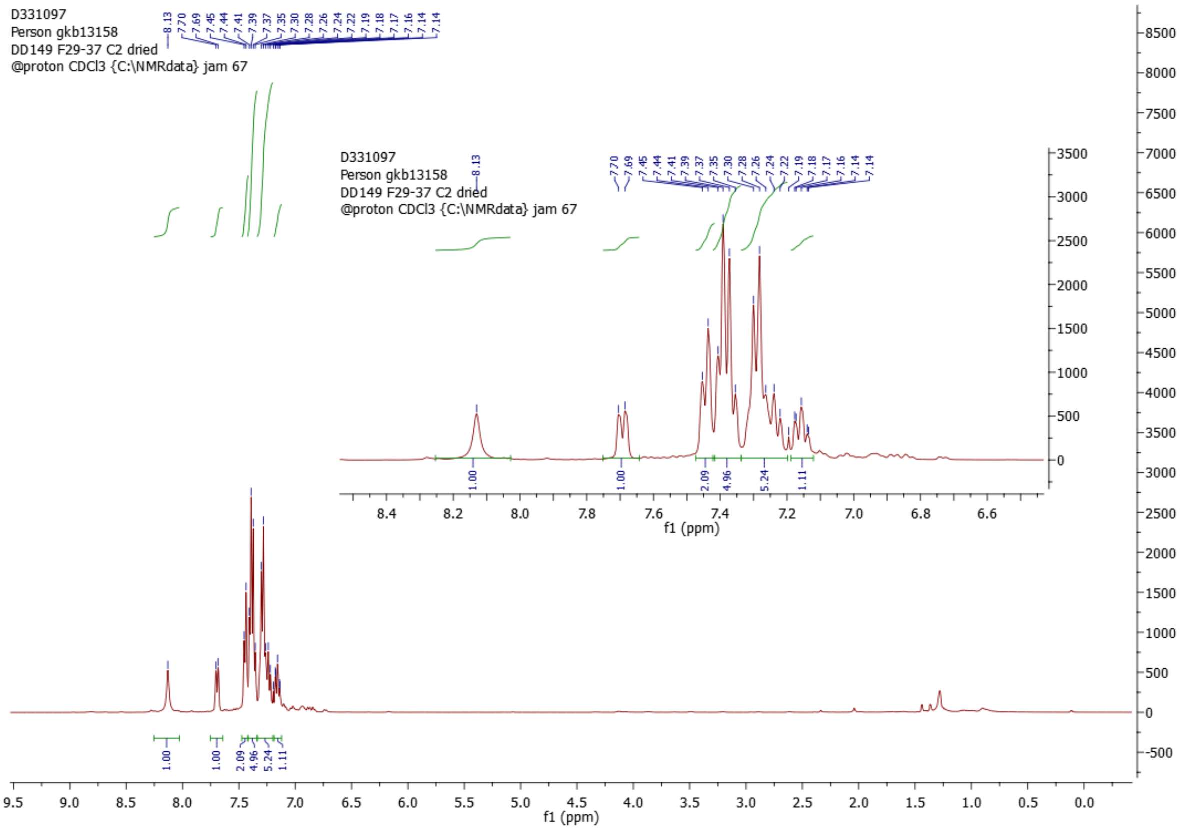
D331097
Person gkb13158
DD149 F29-37 C2 dried
@proton CDCl3 {C:\NMRdata} jam 67
f1 (ppm)

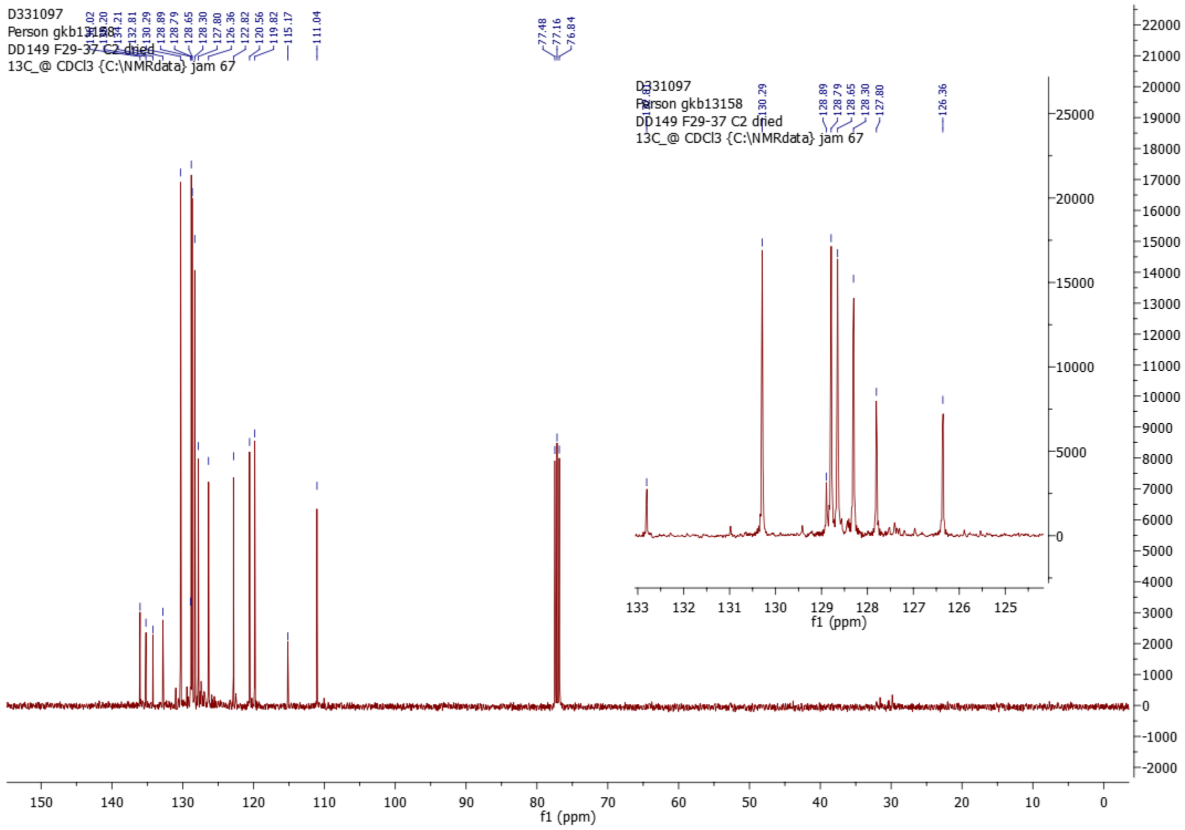
D331097
Person gkb13158
DD149 F29-37 C2 dried
13C_@ CDCl3 {C:\NMRdata} jam 67
f1 (ppm)

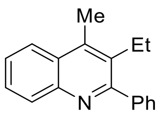
Me
Et
N
Ph

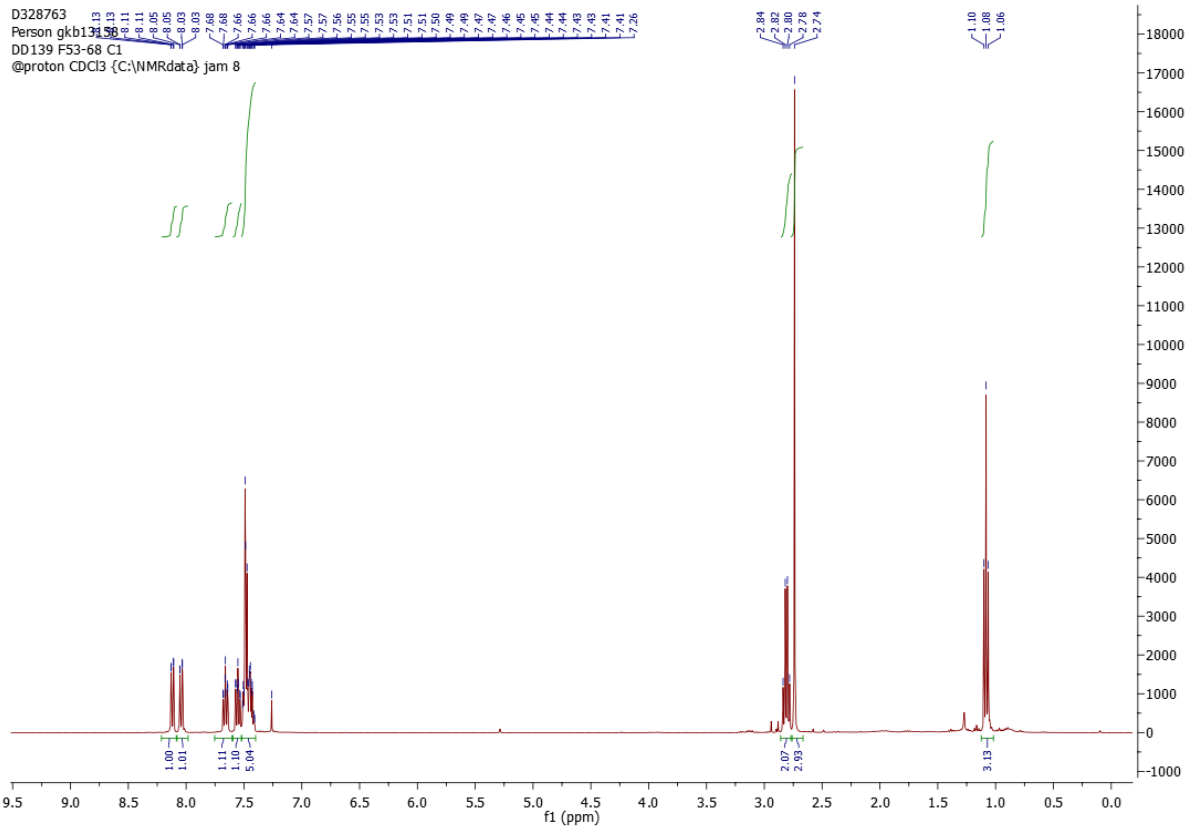
D328763
Person gkb13158
DD139 F53-68 C1
@proton CDCl3 {C:\NMRdata} jam 8
8.13 8.13 8.11 8.11 8.05 8.05 8.03 8.03 7.68 7.68 7.66 7.66 7.64 7.64 7.57 7.57 7.56 7.55 7.55 7.53 7.53 7.51 7.51 7.50 7.49 7.49 7.47 7.47 7.46 7.45 7.45 7.44 7.44 7.43 7.43 7.41 7.41 7.26
2.84 2.82 2.80 2.78 2.74
1.10 1.08 1.06
1.00 1.01 1.11 1.10 5.04 2.07 2.93 3.13
f1 (ppm)

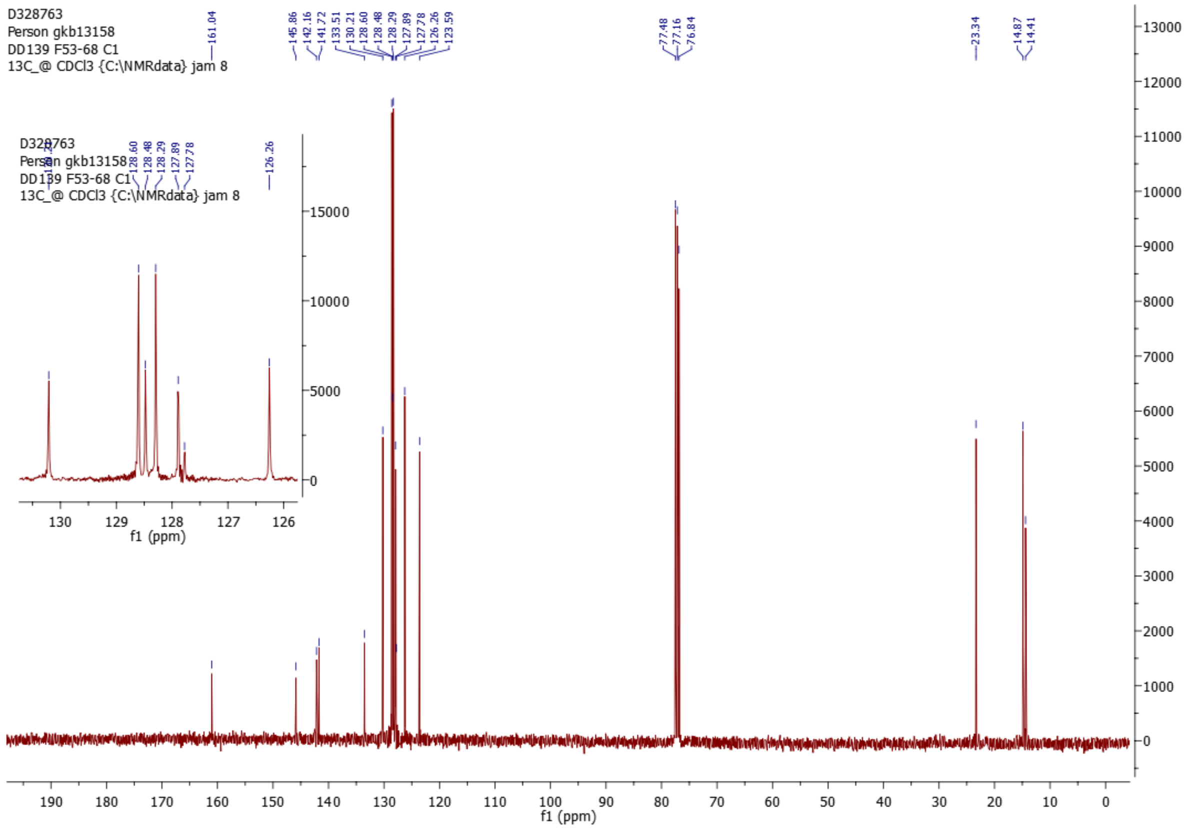
D328763
Person gkb13158
DD139 F53-68 C1
13C_@ CDCl3 {C:\NMRdata} jam 8
161.04
145.86 142.16 141.72 133.51 130.21 128.60 128.48 128.29 127.89 127.78 126.26 123.59
77.48 77.16 76.84
23.34
14.87 14.41
D328763
Person gkb13158
DD139 F53-68 C1
13C_@ CDCl3 {C:\NMRdata} jam 8
128.60 128.48 128.29 127.89 127.78
126.26
f1 (ppm)
f1 (ppm)

NOESY

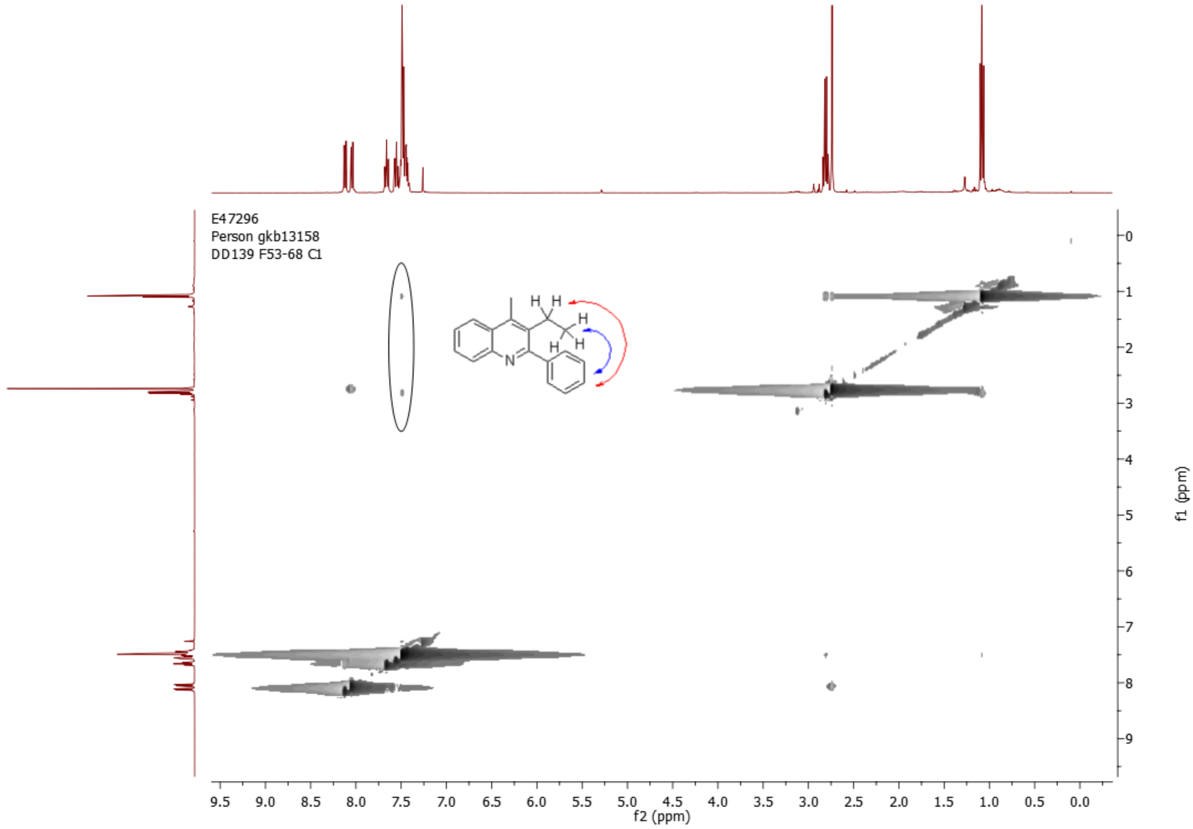
E47296
Person gkb13158
DD139 F53-68 C1
f2 (ppm)
f1 (ppm)

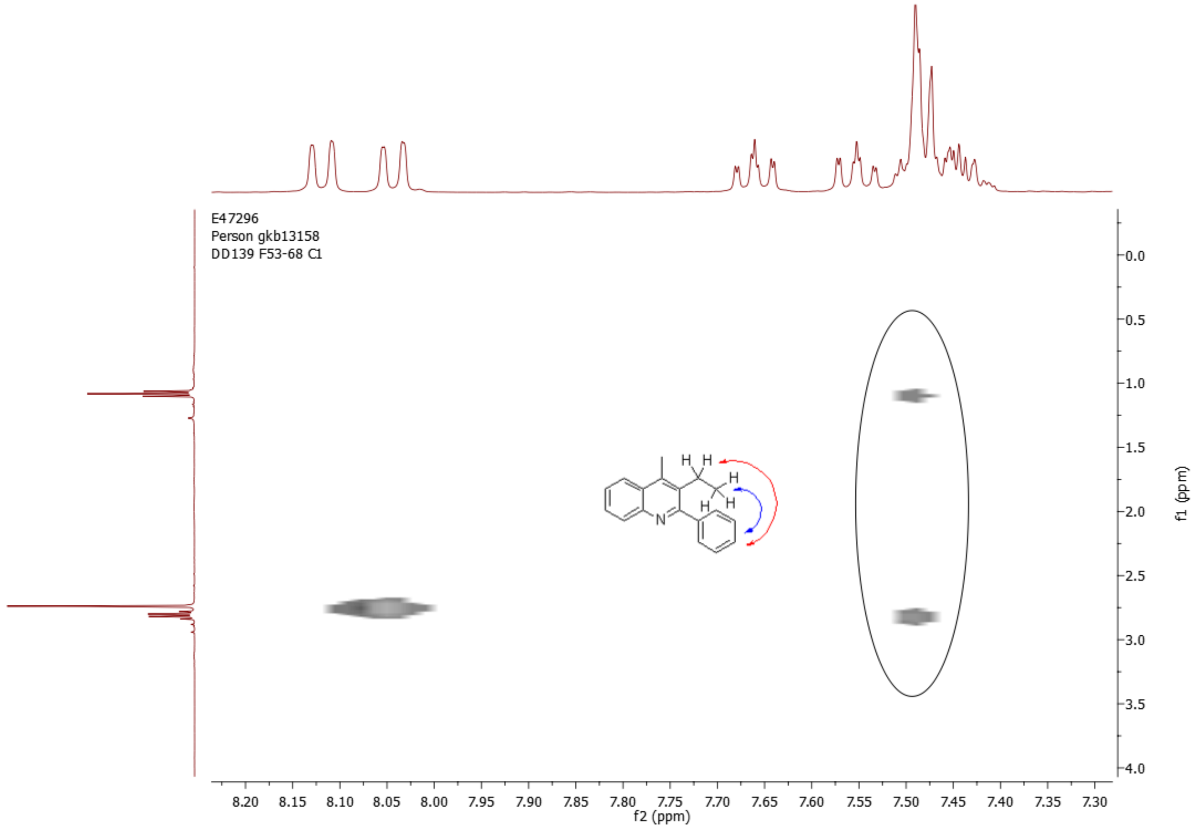
E47296
Person gkb13158
DD139 F53-68 C1
f2 (ppm)
f1 (ppm)

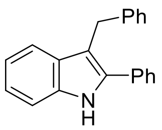

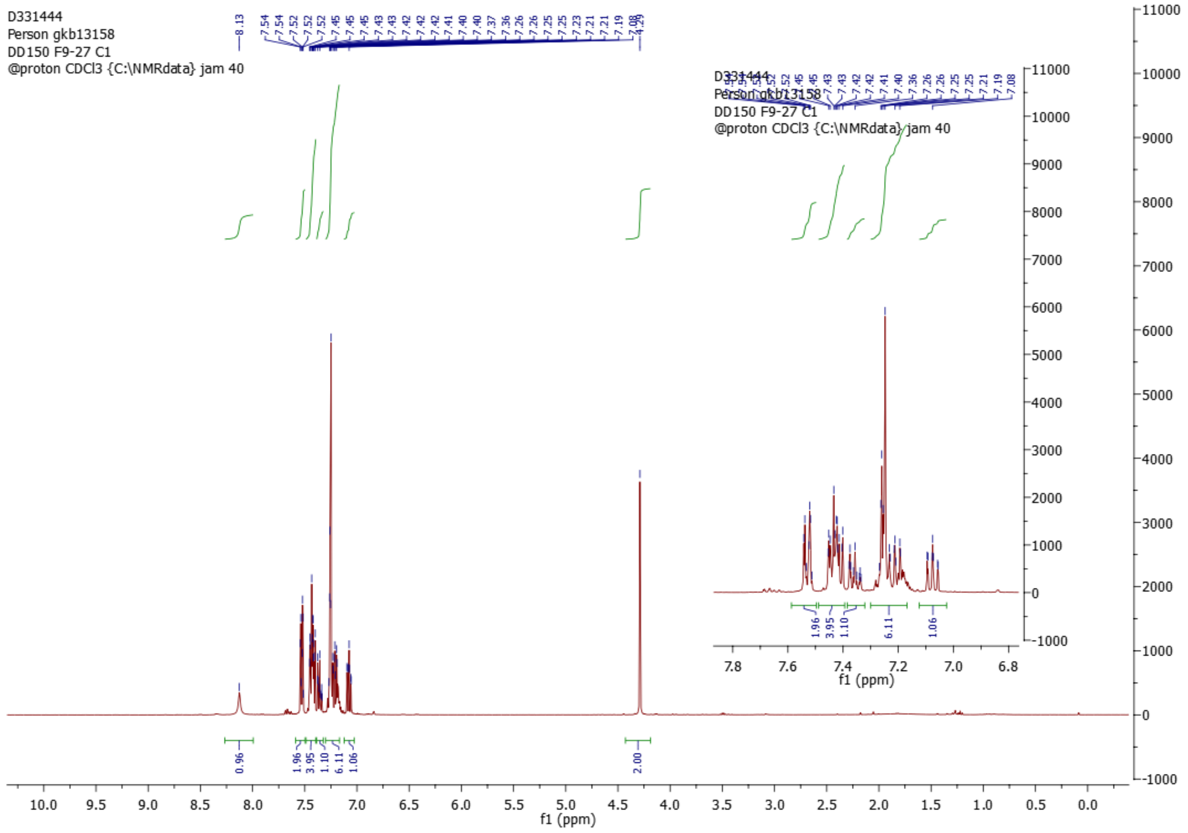
D331444
Person gkb13158
DD150 F9-27 C1
@proton CDCl3 {C:\NMRdata} jam 40
f1 (ppm)

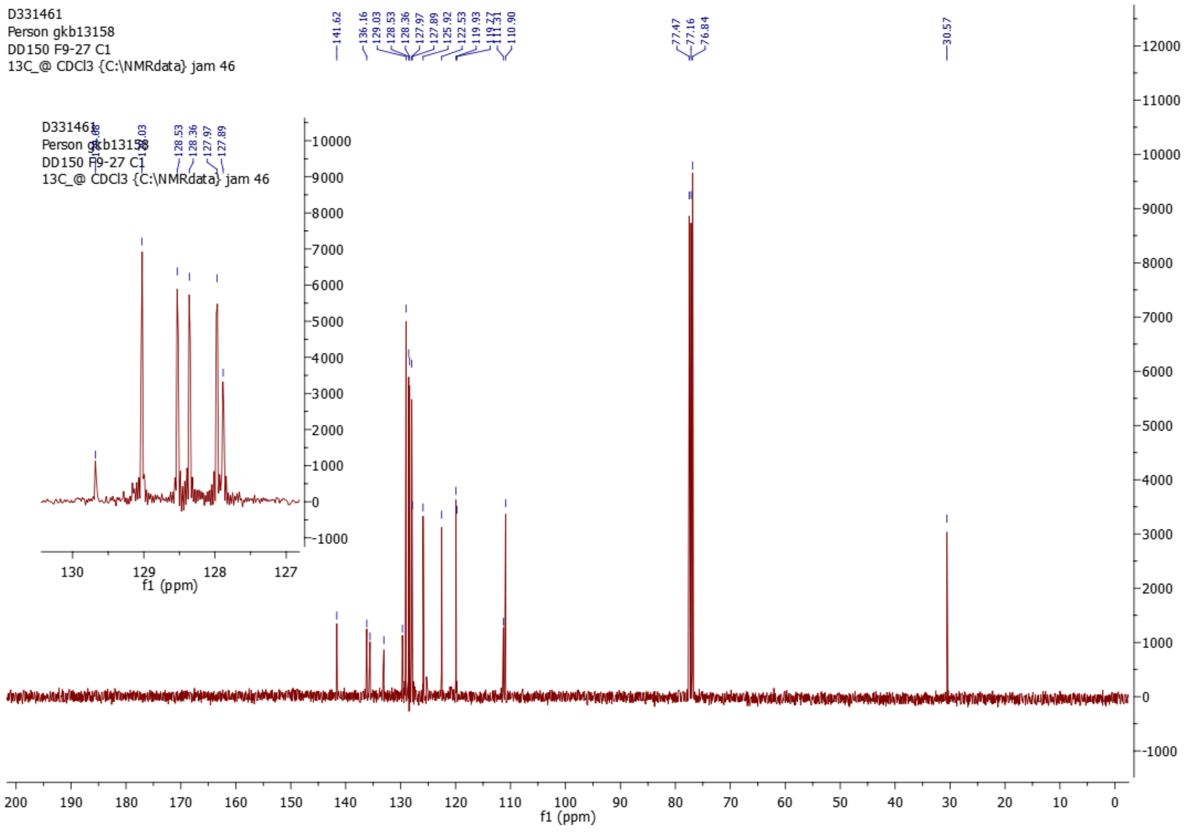
D331461
Person gkb13158
DD150 F9-27 C1
13C_@ CDCl3 {C:\NMRdata} jam 46
f1 (ppm)

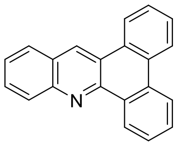

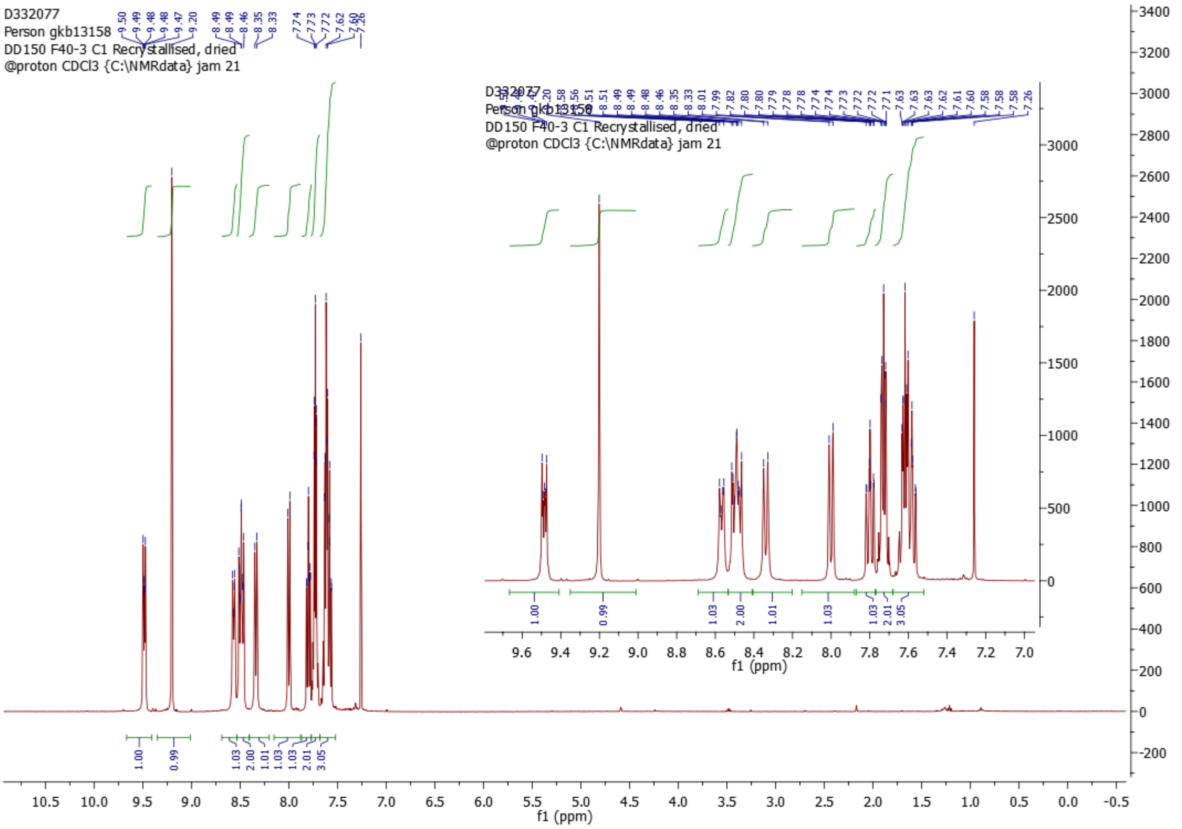

D332077
Person gkb13158
DD150 F40-3 C1 Recrystallised, dried
@proton CDCl3 {C:\NMRdata} jam 21
f1 (ppm)

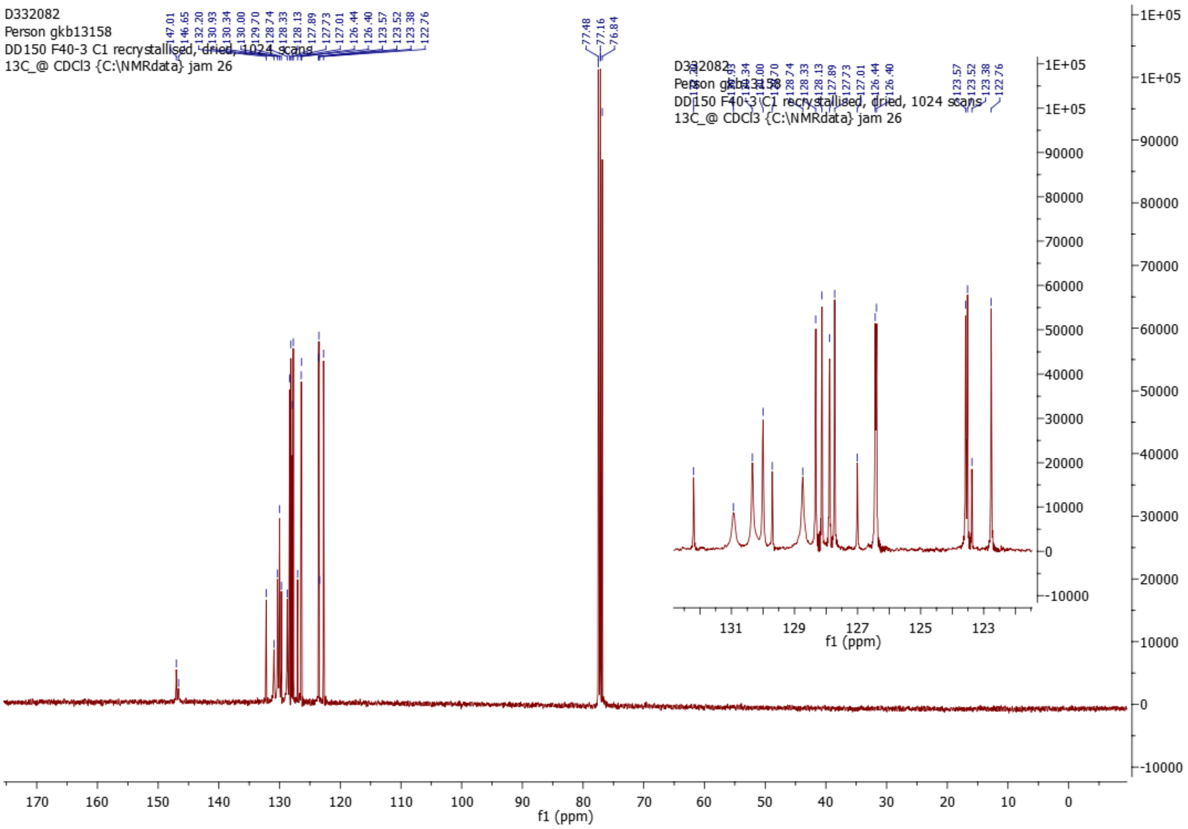

D332082
Person gkb13158
DD150 F40-3 C1 recrystallised, dried, 1024 scans
13C_@ CDCl3 {C:\NMRdata} jam 26
f1 (ppm)

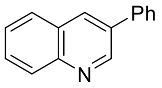
Ph
N

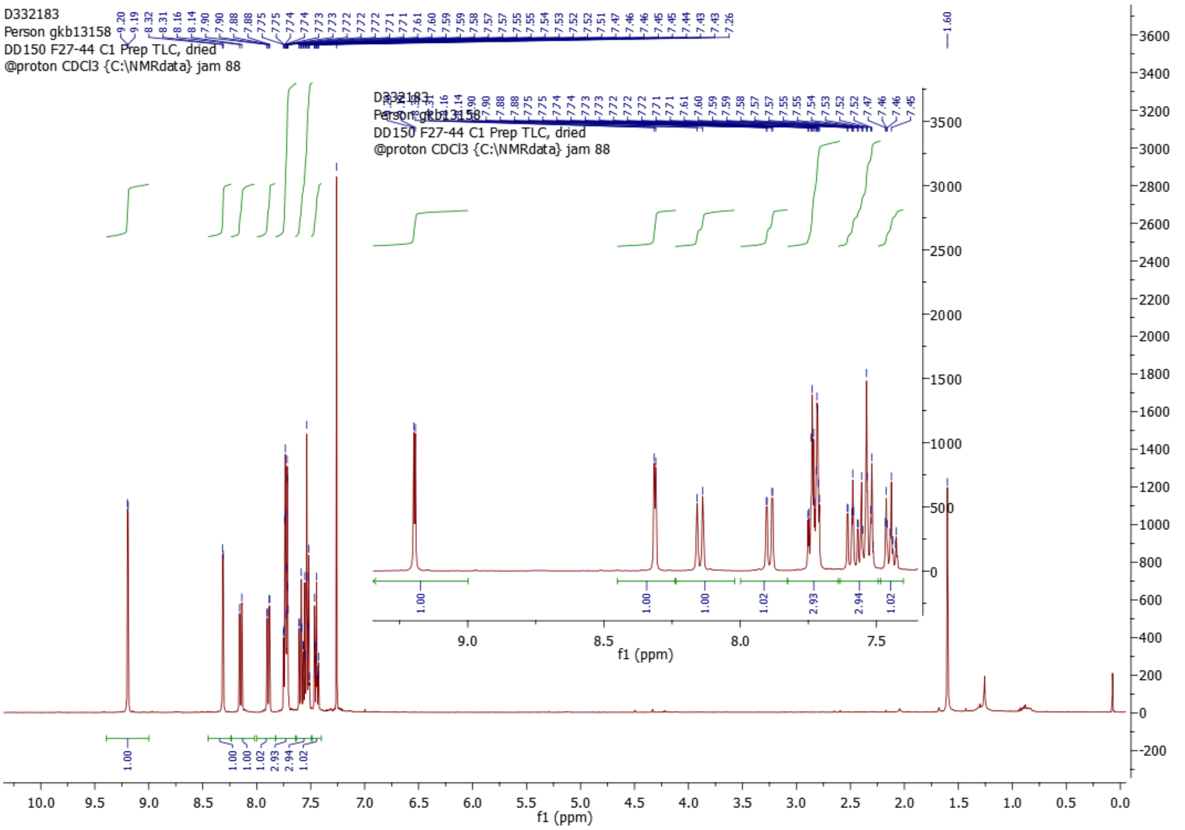
D332183
Person gkb13158
DD150 F27-44 C1 Prep TLC, dried
@proton CDCl3 {C:\NMRdata} jam 88
9.20 9.19 8.32 8.31 8.16 8.14 7.90 7.90 7.88 7.88 7.75 7.75 7.74 7.74 7.73 7.73 7.72 7.72 7.72 7.71 7.71 7.61 7.60 7.59 7.59 7.58 7.57 7.57 7.55 7.55 7.54 7.53 7.52 7.52 7.51 7.47 7.46 7.46 7.45 7.45 7.44 7.43 7.43 7.26
1.60
1.00 1.00 1.00 1.02 2.93 2.94 1.02
f1 (ppm)

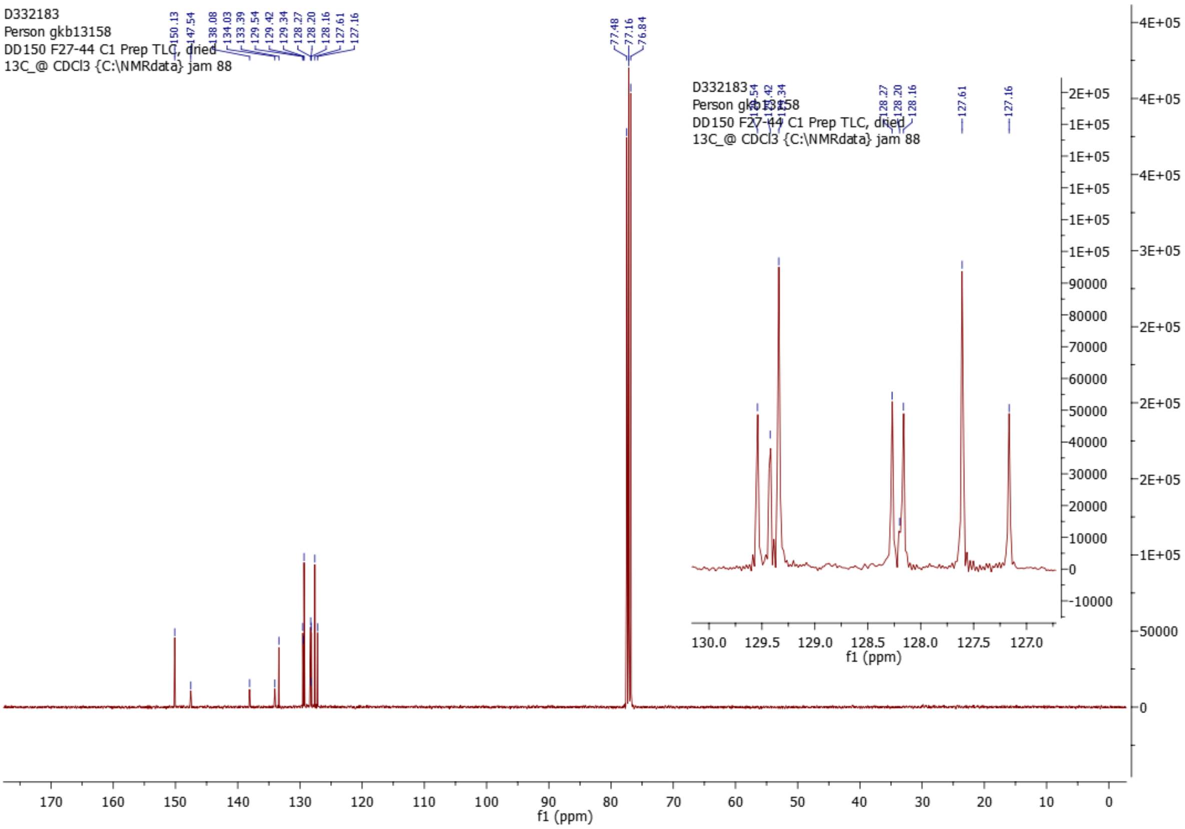
D332183
Person gkb13158
DD150 F27-44 C1 Prep TLC, dried
13C_@ CDCl3 {C:\NMRdata} jam 88
150.13 147.54 138.08 134.03 133.39 129.54 129.42 129.34 128.27 128.20 128.16 127.61 127.16
77.48 77.16 76.84
f1 (ppm)

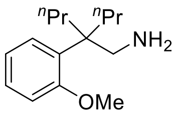

^{n}Pr
^{n}Pr
NH_2
OMe

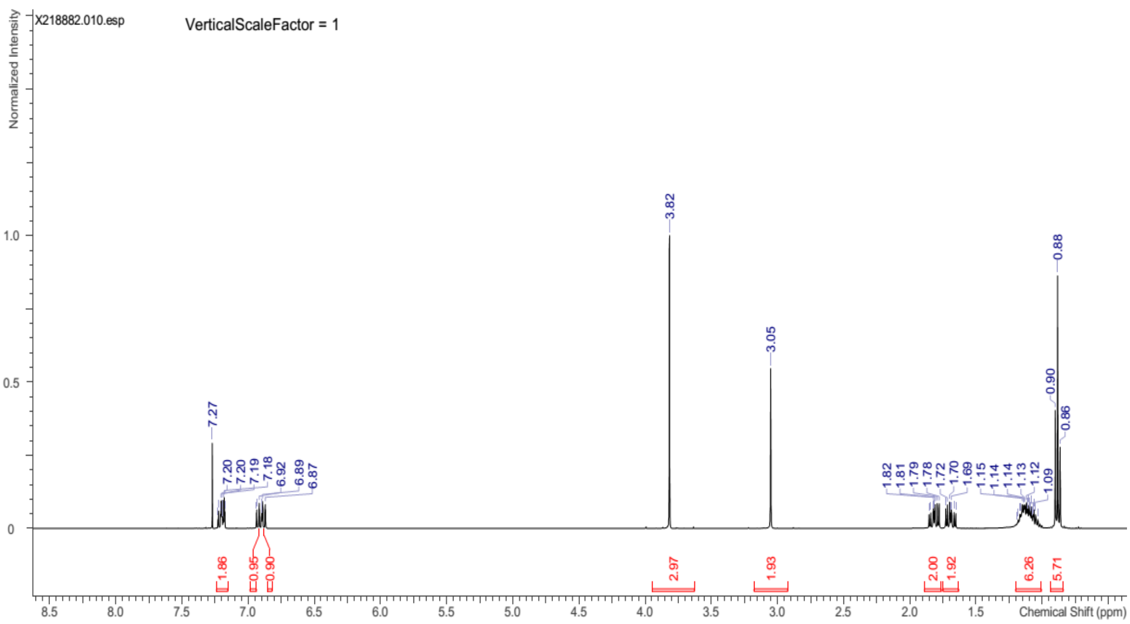

X218882.010.esp
VerticalScaleFactor = 1
Normalized Intensity
7.27
7.20
7.20
7.19
7.18
6.92
6.89
6.87
3.82
3.05
1.82
1.81
1.79
1.78
1.72
1.70
1.69
1.15
1.14
1.13
1.12
1.09
0.90
0.88
0.86
1.86
0.95
0.90
2.97
1.93
2.00
1.92
6.26
5.71
Chemical Shift (ppm)

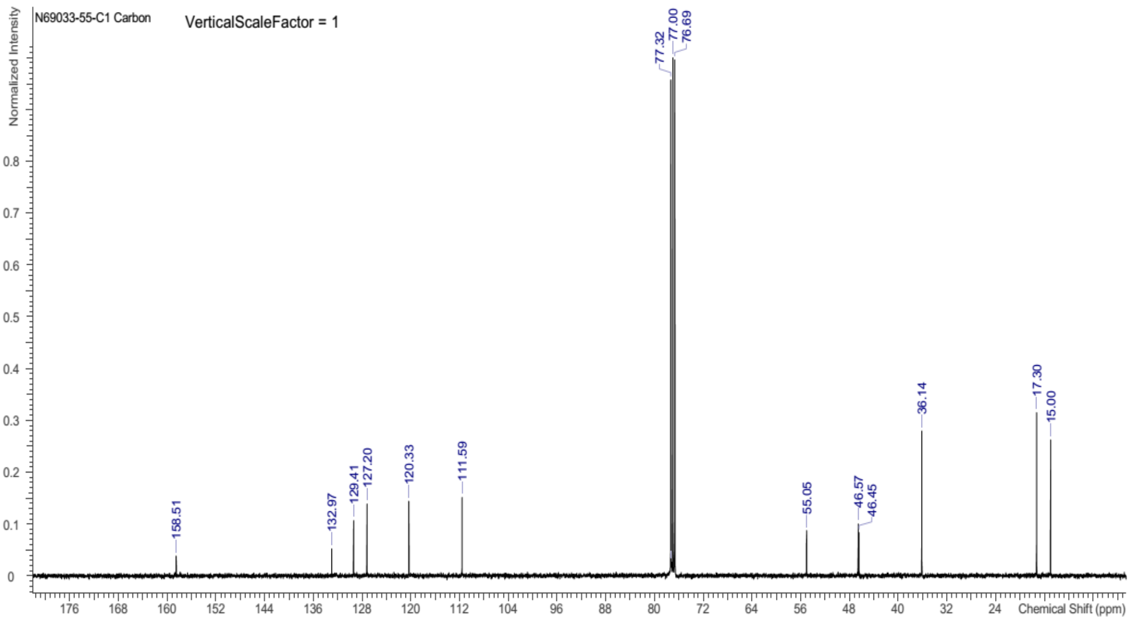

N69033-55-C1 Carbon
VerticalScaleFactor = 1
Normalized Intensity
158.51
132.97
129.41
127.20
120.33
111.59
77.32
77.00
76.69
55.05
46.57
46.45
36.14
17.30
15.00
Chemical Shift (ppm)

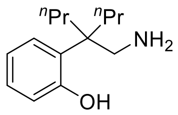

^{n}Pr
^{n}Pr
NH2
OH

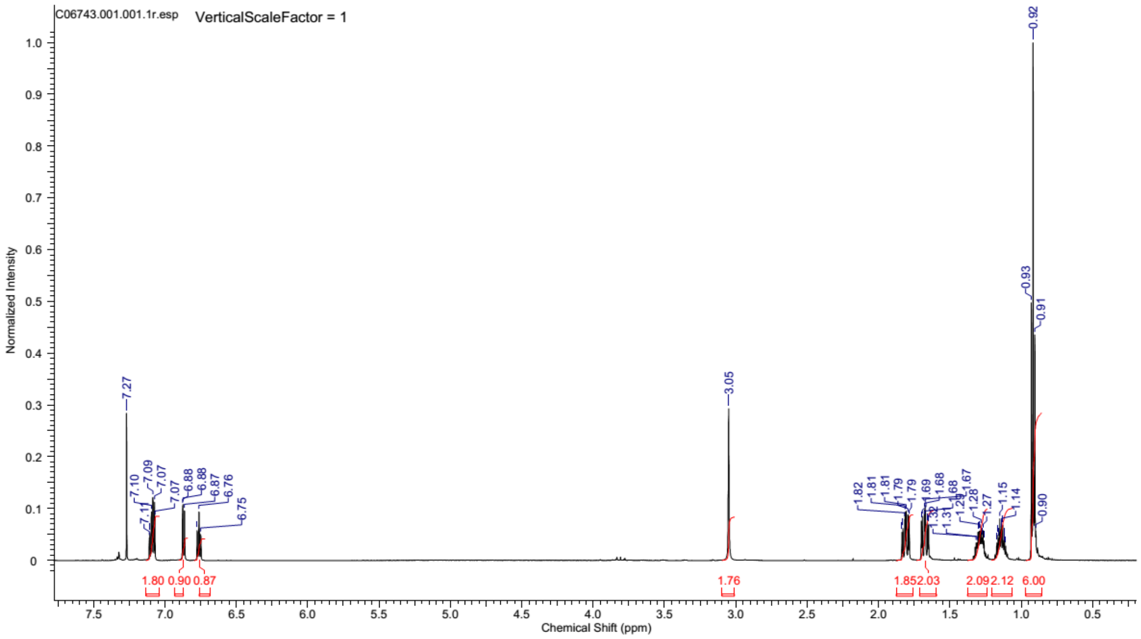

C06743.001.001.1r.esp
VerticalScaleFactor = 1
Normalized Intensity
Chemical Shift (ppm)
7.27
7.11 7.10 7.09 7.07 7.07
6.88 6.88 6.87 6.76 6.75
3.05
1.82 1.81 1.81 1.79 1.79 1.69 1.68 1.68 1.67
1.32 1.31 1.29 1.28 1.27 1.15 1.14
0.93 0.92 0.91 0.90
1.80 0.90 0.87 1.76 1.85 2.03 2.09 2.12 6.00

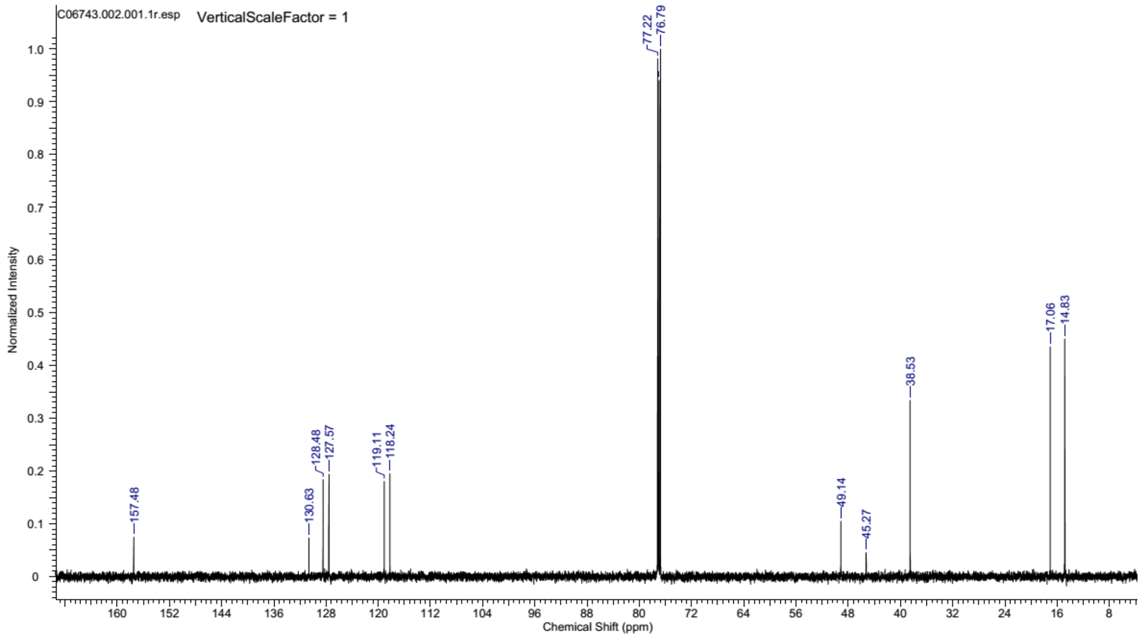

C06743.002.001.1r.esp
VerticalScaleFactor = 1
Normalized Intensity
Chemical Shift (ppm)
157.48
130.63
128.48
127.57
119.11
118.24
77.22
76.79
49.14
45.27
38.53
17.06
14.83

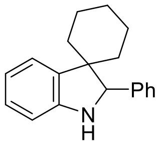
N
H
Ph

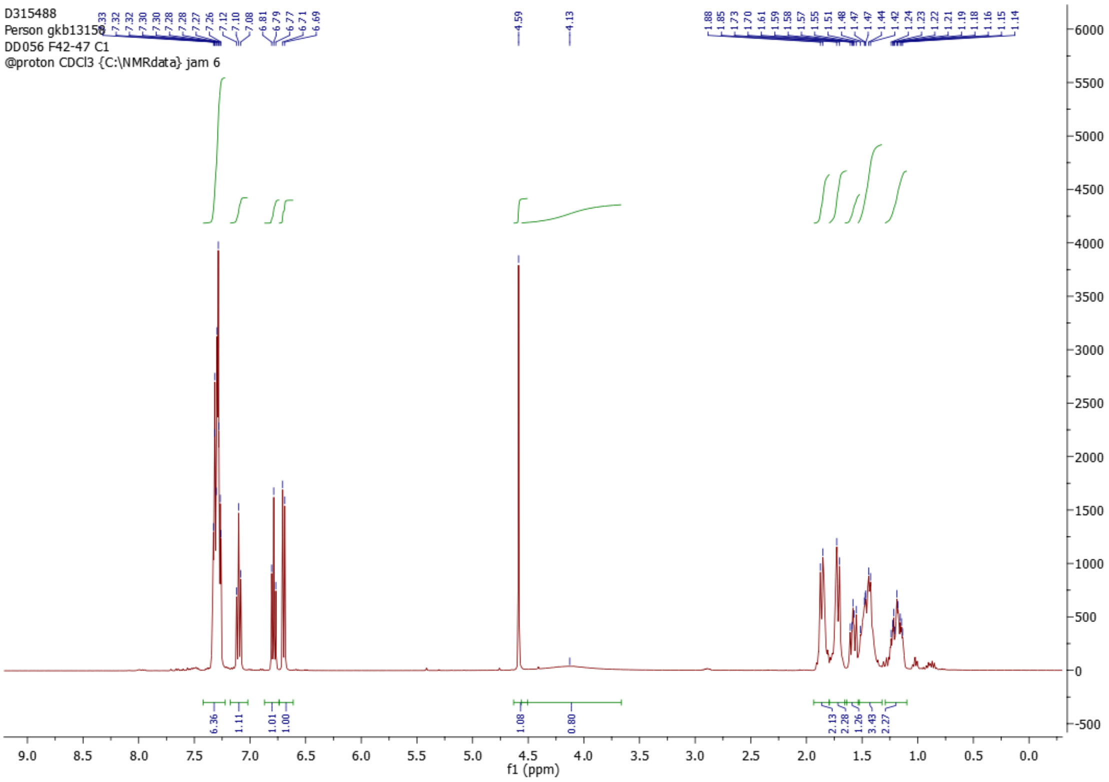
D315488
Person gkb13158
DD056 F42-47 C1
@proton CDCl3 {C:\NMRdata} jam 6
f1 (ppm)

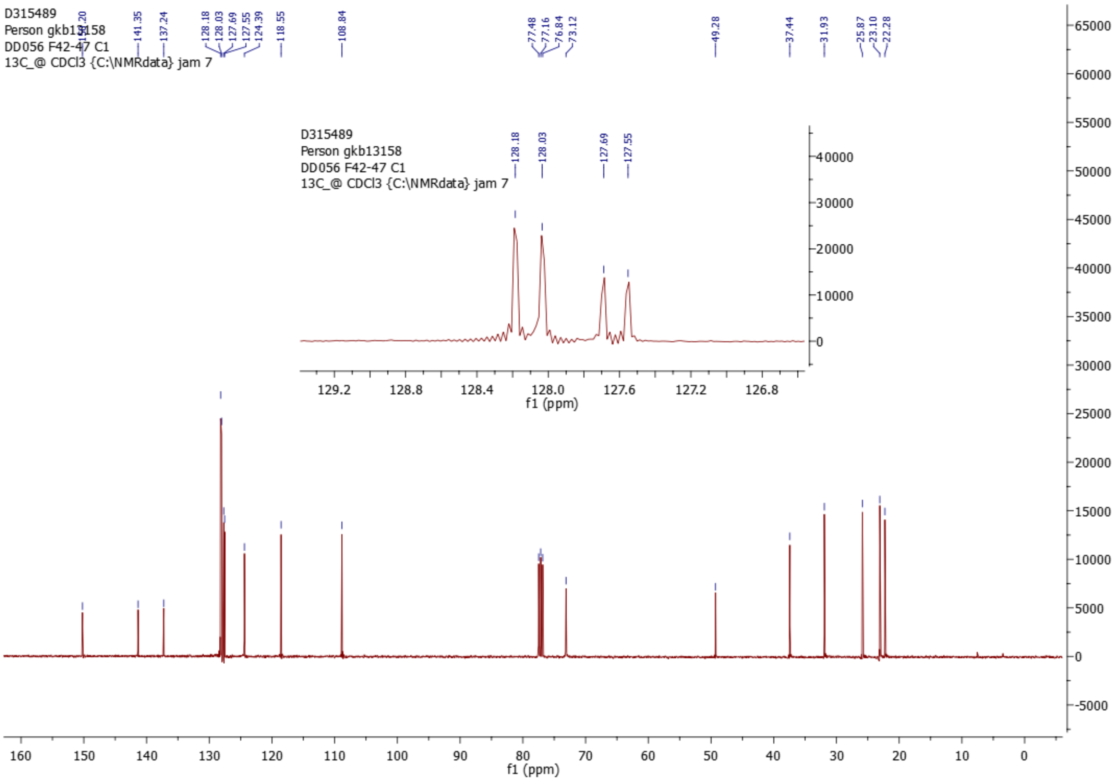
D315489
Person gkb13158
DD056 F42-47 C1
13C_@ CDCl3 {C:\NMRdata} jam 7
f1 (ppm)

References

1 A. D. Lackner, A. V Samant and F. D. Toste, *J. Am. Chem. Soc.*, 2013, **135**, 14090–14093.

2 S. Monfette, Z. R. Turner, S. P. Semproni and P. J. Chirik, *J. Am. Chem. Soc.*, 2012, **134**, 4561–4564.

3 B. K. Trivedi, A. Holmes, T. L. Stoeber, C. J. Blankley, W. H. Roark, J. A. Picard, M. K. Shaw, A. D. Essenburg, R. L. Stanfield and B. R. Krause, *J. Med. Chem.*, 1993, **36**, 3300–3307.

4 T. Kesharwani, A. K. Verma, D. Emrich, J. A. Ward and R. C. Larock, *Org. Lett.*, 2009, **11**, 2591–2593.

5 F. Huber, J. Roesslein and K. Gademann, *Org. Lett.*, 2019, **21**, 2560–2564.

6 M. A. Buil, M. Calbet, M. Castillo, J. Castro, C. Esteve, M. Ferrer, P. Forns, J. González, S. López, R. S. Roberts, S. Sevilla, B. Vidal, L. Vidal and P. Vilaseca, *Eur. J. Med. Chem.*, 2016, **113**, 102–133.

7 T. Yamamoto, T. Furusawa, A. Zhumagazin, T. Yamakawa, Y. Oe and T. Ohta, *Tetrahedron*, 2015, **71**, 19–26.

8 H. J. Kim, J. W. Seo, M. H. Lee, D. S. Shin, H. B. Kim, H. Cho, B. S. Lee and D. Y. Chi, *Tetrahedron*, 2012, **68**, 3942–3947.

9 M. Belletete, F. Brisse, G. Durocher, D. Gravel, A. Heroux and A. Popowycz, *J. Mol. Struct.*, 1993, **297**, 63–80.

10 O. Bakulina, A. Ivanov, V. Suslonov, D. Darin and M. Krasavin, *Beilstein J. Org. Chem.*, 2017, **13**, 1413–1424.

11 C. A. Simoneau and B. Ganem, *Tetrahedron*, 2005, **61**, 11374–11379.

12 C. Gervais, D. Anker and M. Chareire, *Bull. Soc. Chim. Fr.*, 1979, **2**, 241–248.

13 H. Leuchs, *Chem. Ber.*, 1932, **65**, 1586–1589.

14 L.-L. Cao, D.-S. Wang, G.-F. Jiang and Y.-G. Zhou, *Tetrahedron Lett.*, 2011, **52**, 2837–2839.

15 J. C. Ciula and A. Streitwieser, *J. Org. Chem.*, 1991, **56**, 1989–1993.

16 C. Cao, L. Wang, Z. Cai, L. Zhang, J. Guo, G. Pang and Y. Shi, *Eur. J. Org. Chem.*, 2011, **3**, 1570–1574.

17 M. Cariou, R. Carlier and J. Simonet, *Bull. Soc. Chim. Fr.*, 1986, **5**, 781–792.

18 Z. Duan, W. Li and A. Lei, *Org. Lett.*, 2016, **18**, 4012–4015.

19 J. T. R. Liddon, J. A. Rossi-Ashton, R. J. K. Taylor and W. P. Unsworth, *Org. Lett.*, 2018, **20**, 3349–3353.

20 G. Rodriguez, Y. Benito and F. Temprano, *Chem. Lett.*, 1985, **14**, 427–428.

21 A. Kucerovy, J. S. Hathaway, P. G. Manner and O. Repic, *Synth. Commun.*, 1992, **22**, 729–733.

22 O. Ogata, H. Nara, K. Matsumura and Y. Kayaki, *Org. Lett.*, 2019, **21**, 9954–9959.

23 C. Liu, L. Ding, G. Guo, W. Liu and F. L. Yang, *Org. Biomol. Chem.*, 2016, **14**, 2824–2827.

24 M. Peña-Lõpez, H. Neumann and M. Beller, *Chem. Eur. J.*, 2014, **20**, 1818–1824.

25 S. L. Mao, Y. Sun, G. A. Yu, C. Zhao, Z. J. Han, J. Yuan, X. Zhu, Q. Yang and S. H. Liu, *Org. Biomol. Chem.*, 2012, **10**, 9410–9417.

26 Y. D. Wu, J. R. Ma, W. M. Shu, K. L. Zheng and A. X. Wu, *Tetrahedron*, 2016, **72**, 4821–4826.

27 J. Mao, Z. Wang, X. Xu, G. Liu, R. Jiang, H. Guan, Z. Zheng and P. J. Walsh, *Angew. Chem. Int. Ed.*, 2019, **58**, 11033–11038.

28 M. E. Budén, V. A. Vaillard, S. E. Martin and R. A. Rossi, *J. Org. Chem.*, 2009, **74**, 4490–4498.

29 L. L. Cao, D. S. Wang, G. F. Jiang and Y. G. Zhou, *Tetrahedron Lett.*, 2011, **52**, 2837–2839.

30 J. Li, E. Tan, N. Keller, Y. H. Chen, P. M. Zehetmaier, A. C. Jakowetz, T. Bein and P. Knochel, *J. Am. Chem. Soc.*, 2019, **141**, 98–103.

31 P. K. Freeman and N. Ramnath, *J. Org. Chem.*, 1991, **56**, 3646–3651.

32 M. Lucarini, E. Marchesi, G. F. Pedulli and C. Chatgilialoglu, *J. Org. Chem.*, 1998, **63**, 1687–1693.

33 W. B. Liu, D. P. Schuman, Y. F. Yang, A. A. Toutov, Y. Liang, H. F. T. Klare, N. Nesnas, M. Oestreich, D. G. Blackmond, S. C. Virgil, S. Banerjee, R. N. Zare, R. H. Grubbs, K. N. Houk and B. M. Stoltz, *J. Am. Chem. Soc.*, 2017, **139**, 6867–6879.
